# Odronextamab monotherapy in patients with relapsed/refractory diffuse large B cell lymphoma: primary efficacy and safety analysis in phase 2 ELM-2 trial

**DOI:** 10.1038/s43018-025-00921-6

**Published:** 2025-03-17

**Authors:** Won Seog Kim, Tae Min Kim, Seok-Goo Cho, Isidro Jarque, Elżbieta Iskierka-Jażdżewska, Li Mei Poon, H. Miles Prince, Huilai Zhang, Junning Cao, Mingzhi Zhang, Benoît Tessoulin, Sung Yong Oh, Francesca Lim, Cecilia Carpio, Tran-Der Tan, Sabarish Ayyappan, Antonio Gutierrez, Jingxian Cai, Melanie Ufkin, Saleem Shariff, Jurriaan Brouwer-Visser, Aafia Chaudhry, Hesham Mohamed, Srikanth Ambati, Jan Walewski, on behalf of the ELM-2 Investigators, Hannah Rose, Geoffrey Chong, Vinod Ganju, Michael Chu, Mary-Margaret Keating, Yuqin Song, Jun Zhu, Xiaoyan Ke, Shuhua Yi, Huilai Zhang, Qingyuan Zhang, Liqun Zou, Mingzhi Zhang, Dengju Li, Wenbin Qian, Ou Bai, Li Gao, Jie Jin, Caixia Li, Huiqiang Huang, Zheng Wei, Youhua Chen, Pengcheng He, Gandhi Laurent Damaj, Kamal Bouabdballah, Emmanuel Bachy, Corinne Haioun, Franck Morschhauser, Sylvain Choquet, Vincent Delwail, Catherine Thieblemont, Johannes Duell, Thomas Weber, Paul Graf La Rosee, Holger Hebart, Enrico Capochiani, Vittorio Zilioli, Francesca Rossi, Stefano Luminari, Pier Luigi Zinzani, Laura Bagnato, Gianluca Gaidano, Marco Brociner, Cristina Skert, Monica Tani, Roberta Battistini, Leonardo Flenghi, Ryusuke Yamamoto, Kunihiro Tsukasaki, Kenichi Ishizawa, Tomomi Tobai, Toshiki Uchida, Yosuke Minami, Nobuhiko Yamauchi, Junichiro Yuda, Masahiro Takeuchi, Hirokazu Nagai, Youko Suehiro, Yoshiaki Ogawa, Junya Kuroda, Tatsuro Jo, Hirohisa Nakamae, Isao Yoshida, Michal Taszner, Ewa Lech-Maranda, Wanda Knopinska-Posluszny, Tomasz Wrobel, Tadeusz Robak, Wen Son Hsieh, Shin Yeu Ong, Hyeon-Seok Eom, Yeung-Chul Mun, Young Rok Do, Jin Seok Kim, Byung Soo Kim, Jae-Cheol Jo, Ana Jimenez-Ubieto, Rafael Andreu, Alejandro Martin, Agustin Penedo Coello, Raul Cordoba, Aranzazu Alonso, Laura Magnano, Eva Gonzalez-Barca, Sara Miqueleiz, Tsai Yun Chen, Su Peng Yeh, Shang-Ju Wu, Ming-Chung Wang, David Cunningham, Andrea Kuhnl, David Tucker, David Lewis, Nagah Elmusharaf, John Allan, Thomas Jandl, Sami Ibrahimi, Deepa Jagadeesh, Lori Leslie, Parameswaran Venugopal, Jon Arnason, Jose C. Villasboas, Rakhee Vaidya, Don Stevens, Farrukh Awan, Andreas Klein, Umar Farooq

**Affiliations:** 1 Sungkyunkwan University School of Medicine, Samsung Medical Center Division of Hematology–Oncology, Seoul, South Korea; 2 Seoul National University Hospital, Seoul National University Cancer Research Institute, Seoul, South Korea; 3 Seoul St. Mary’s Hospital, The Catholic University of Korea, Seoul, South Korea; 4 Hematology Department, Hospital Universitari i Politècnic La Fe, Centro de Investigación Biomédica en Red de Cáncer—CIBERONC, Valencia, Spain; 5 Copernicus Memorial Hospital, Department of General Hematology, Medical University of Łódź, Łódź, Poland; 6 Department of Haematology-Oncology, National University Cancer Institute, National University Hospital, Singapore, Singapore; 7 Epworth HealthCare and University of Melbourne, Melbourne, Victoria Australia; 8 Department of Lymphoma, Tianjin Medical University Cancer Institute and Hospital, National Clinical Research Center for Cancer, Key Laboratory of Cancer Prevention and Therapy, Tianjin, China; 9 Fudan University Shanghai Cancer Center, Shanghai, China; 10 Department of Oncology, First Affiliated Hospital, Zhengzhou University, Zhengzhou, China; 11 Hematology Department, Nantes University Hospital, Nantes, France; 12 Dong-A University Hospital, Busan, South Korea; 13 Singapore General Hospital, Singapore, Singapore; 14 Department of Hematology, Vall d’Hebron Institute of Oncology (VHIO), University Hospital Vall d’Hebron, Autonomous University of Barcelona (UAB), Barcelona, Spain; 15 Hematology and Medical Oncology, Koo Foundation Sun Yat Sen Cancer Center, Taipei City, Taiwan; 16 University of Iowa Hospital and Clinics, Iowa City, IA USA; 17 Department of Hematology, Hospital Universitario Son Espases, IdISBa, Palma, Spain; 18 Regeneron Pharmaceuticals, Inc., Tarrytown, NY USA; 19 Regeneron UK, Ltd., Uxbridge, UK; 20 Narodowy Instytut Onkologii im. Marii Skłodowskiej-Curie Państwowy Instytut Badawczy, Warszawa, Poland; 21 University Hospital Geelong, Geelong, Victoria Australia; 22 Austin Health, Heidelberg, Victoria Australia; 23 Hudson Institute of Medical Research, Melbourne, Victoria Australia; 24 Cross Cancer Institute, University of Alberta, Edmonton, Alberta Canada; 25 Dalhousie University, Halifax, Nova Scotia Canada; 26 Peking University Cancer Hospital and Institute, Beijing, China; 27 Lymphoma Research Center, Peking University Third Hospital, Beijing, China; 28 National Clinical Research Center for Blood Diseases, Institute of Hematology and Blood Diseases Hospital, Chinese Academy of Medical Sciences and Peking Union Medical College, Tianjin, China; 29 Tianjin Institutes of Health Science, Tianjin, China; 30 Tianjin’s Clinical Research Center for Cancer, National Clinical Research Center for Cancer, Tianjin Medical University Cancer Institute and Hospital, Tianjin Medical University, Tianjin, China; 31 Harbin Medical University Cancer Hospital, Harbin, China; 32 Cancer Center, West China Hospital of Sichuan University, Chengdu, China; 33 The First Affiliated Hospital of Zhengzhou University, Zhengzhou, China; 34 Tongji Hospital, Tongji Medical College, Huazhong University of Science and Technology, Wuhan, China; 35 The Second Affiliated Hospital, College of Medicine, Zhejiang University, Hangzhou, China; 36 The First Hospital of Jilin University, Changchun, China; 37 Children’s Hospital of Soochow University, Suzhou, China; 38 The First Affiliated Hospital of Zhejiang University School of Medicine, Hangzhou, China; 39 Zhejiang Provincial Clinical Research Center for Hematological Disorders, Hangzhou, China; 40 The First Affiliated Hospital of Soochow University, Suzhou, China; 41 Sun Yat-sen University Cancer Center, Guangzhou, China; 42 Zhongshan Hospital, Fudan University, Shanghai, China; 43 Renmin Hospital of Wuhan University, Hubei General Hospital, Wuhan, China; 44 The First Affiliated Hospital of Xi’an Jiaotong University, Xian, China; 45 Caen University Hospital, Caen, France; 46 CHU De Bordeaux, Bordeaux, France; 47 Hôpital Lyon Sud, Hospices Civils de Lyon, Lyon, France; 48 CHU Henri Mondor, Créteil, France; 49 CHU de Lille, Hôpital Claude Huriez, Lille, France; 50 Pitié-Salpêtrière University Hospital, APHP, Sorbonne Université, Paris, France; 51 CHU Poitiers, Poitiers, France; 52 Hôpital Saint-Louis, Paris, France; 53 University Hospital of Würzburg, Würzburg, Germany; 54 Martin Luther University Halle-Wittenberg, Halle, Germany; 55 Schwarzwald-Baar Klinikum, Villingen-Schwenningen, Germany; 56 Stauferklinikum Schwäbisch Gmünd, Mutlangen, Germany; 57 Azienda Unità Sanitaria Locale (USL) Toscana Nord Ovest, Livorno, Italy; 58 ASST Grande Ospedale Metropolitano Niguarda, Milan, Italy; 59 UOC Ematologia-Fondazione IRCCS Cà Granda OM Policlinico, Milan, Italy; 60 Azienda Unità Sanitaria Locale-IRCCS, Reggio Emilia, Italy; 61 University of Modena and Reggio Emilia, Reggio Emilia, Italy; 62 IRCCS Azienda Ospedaliero-Universitaria di Bologna, Istituto di Ematologia ‘Seràgnoli’, Bologna, Italy; 63 Università di Bologna, Bologna, Italy; 64 Università Del Piemonte Orientale, Novara, Italy; 65 Ospedale di Circolo e Fondazione Macchi-ASST Sette Laghi, Varese, Italy; 66 Ospedale Dell’Angelo, Venice, Italy; 67 Santa Maria delle Croci Hospital, Ravenna, Italy; 68 Azienda Ospedaliera San Camillo, Rome, Italy; 69 Azienda Ospedaliera di Perugia, Perugia, Italy; 70 Kobe City Medical Center General Hospital, Kobe, Japan; 71 International Medical Center, Saitama Medical University, Saitama, Japan; 72 Yamagata University, Yamagata, Japan; 73 University of Michigan, Comprehensive Cancer Center, Ann Arbor, MI USA; 74 Japanese Red Cross Aichi Medical Center Nagoya Daini Hospital, Nagoya, Japan; 75 National Cancer Center Hospital East, Kashiwa, Japan; 76 Cancer Institute Hospital, Japanese Foundation for Cancer Research, Tokyo, Japan; 77 Chiba Cancer Center, Chiba, Japan; 78 National Hospital Organization Nagoya Medical Center, Nagoya, Japan; 79 National Organization Kyushu Cancer Center, Fukuoka, Japan; 80 Tokai University School of Medicine, Kanagawa, Japan; 81 Kyoto Prefectural University of Medicine, Kyoto, Japan; 82 Japanese Red Cross Nagasaki Genbaku Hospital, Nagasaki, Japan; 83 Osaka Metropolitan University Hospital, Osaka, Japan; 84 Shikoku Cancer Center, Matsuyama, Japan; 85 Medical University of Gdansk, Gdansk, Poland; 86 Institute of Hematology and Transfusion Medicine, Warsaw, Poland; 87 The Sea Hospital in Gdynia, Gdynia, Poland; 88 Wroclaw Medical University, Wroclaw, Poland; 89 Copernicus Memorial Hospital, Medical University of Lodz, Lodz, Poland; 90 ICON Cancer Centre, Singapore, Singapore; 91 National Cancer Center, Goyang, Republic of Korea; 92 Ewha Womans University School of Medicine, Seoul, Republic of Korea; 93 Dongsan Medical Center, Daegu, Republic of Korea; 94 Yonsei University College of Medicine, Seoul, Republic of Korea; 95 Korea University College of Medicine, Seoul, Republic of Korea; 96 Ulsan University Hospital, University of Ulsan College of Medicine, Ulsan, Republic of Korea; 97 Hospital Universitario 12 de Octubre, Madrid, Spain; 98 Hospital Universitari i Politècnic La Fe, Valencia, Spain; 99 Hospital Clínico Universitario de Salamanca, Salamanca, Spain; 100 START Madrid-CIOCC Phase I Unit, University Hospital HM Sanchinarro, Madrid, Spain; 101 Hospital Universitario Fundacion Jimenez Diaz, Madrid, Spain; 102 Hospital Universitario Quirónsalud Madrid, Madrid, Spain; 103 Hospital Clínic de Barcelona, Barcelona, Spain; 104 Institut Catalá d’Oncologia Hospitalet, IDIBELL, Universitat de Barcelona, Barcelona, Spain; 105 Hospital de la Santa Creu i Sant Pau, IIB-Sant Pau and José Carreras Leukemia Research Institutes, Barcelona, Spain; 106 National Cheng Kung University Hospital, Tainan, Taiwan; 107 China Medical University Hospital, Taichung, Taiwan; 108 National Taiwan University Hospital, Taipei, Taiwan; 109 Chang Gung Memorial Hospital, Kaohsiung, Taiwan; 110 Royal Marsden Hospital, London, UK; 111 King’s College Hospital, London, UK; 112 Royal Cornwall Hospital, Truro, UK; 113 Derriford Hospital, Plymouth, UK; 114 University Hospital of Wales, Cardiff, UK; 115 Weill Cornell Medicine, New York, NY USA; 116 Stony Brook University Hospital, Stony Brook, New York, NY USA; 117 OU Health-Stephenson Cancer Center, Oklahoma City, OK USA; 118 Cleveland Clinic, Cleveland, OH USA; 119 John Theurer Cancer Center, Hackensack Meridian Health, Hackensack, NJ USA; 120 Rush University Medical Center, Chicago, IL USA; 121 Beth Israel Deaconess Medical Center, Boston, MA USA; 122 Mayo Clinic, Rochester, MN USA; 123 Atrium Health Wake Forest Baptist, Wake Forest University, Winston-Salem, NC USA; 124 Norton Cancer Institute, Louisville, KY USA; 125 Harold C. Simmons Comprehensive Cancer Center, University of Texas Southwestern Medical Center, Dallas, TX USA; 126 Tufts Medical Center, Boston, MA USA; 127 University of Iowa, Iowa City, IA USA

**Keywords:** Clinical trial design, Cancer, Antibody therapy, Drug discovery

## Abstract

The phase 2, multicohort, ongoing ELM-2 study evaluates odronextamab, a CD20×CD3 bispecific antibody, in patients with relapsed/refractory (R/R) B cell non-Hodgkin lymphoma after ≥2 lines of therapy. Here primary analysis of the diffuse large B cell lymphoma (DLBCL) cohort is reported. Patients received intravenous odronextamab in 21-day cycles until progression or unacceptable toxicity, with cycle 1 step-up dosing to mitigate cytokine release syndrome (CRS) risk. The primary endpoint was objective response rate (ORR). Secondary endpoints included complete response (CR) rate, duration of response, progression-free survival (PFS) and overall survival. A total of 127 patients were enrolled. At the 29.9-month efficacy follow-up, the ORR was 52.0% and CR rate was 31.5%. Median durations of response and CR were 10.2 and 17.9 months, respectively. Undetectable minimal residual disease at cycle 4 day 15 was associated with PFS benefit. With a step-up of 0.7 to 4 to 20 mg (*n* = 60), CRS was the most common treatment-emergent adverse event (53.3% (grade ≥3, 1.7%)). No immune effector cell-associated neurotoxicity syndrome was reported. Infections were reported in 82/127 (64.6%) patients (grade ≥3, 38.6%; coronavirus disease 2019, 18.1% (grade ≥3, 12.6%)). In conclusion, odronextamab showed encouraging efficacy in heavily pretreated R/R DLBCL and generally manageable safety with supportive care. Clinical trial registration: NCT03888105.

## Abstract (web summary)

Kim and colleagues perform a phase 2 clinical trial (ELM-2) of odronextamab monotherapy in patients with relapsed/refractory diffuse large B cell lymphoma and report on the primary analyses of the efficacy and safety profile.

## Main

Diffuse large B cell lymphoma (DLBCL) is an aggressive form of B cell non-Hodgkin lymphoma (B-NHL)^1^. Approximately 30% of people will relapse after first-line treatment with immunochemotherapy (for example, rituximab, cyclophosphamide, doxorubicin, vincristine and prednisolone) and poor outcomes are observed in this relapsed/refractory (R/R) setting; the median overall survival (OS) is approximately 6–7 months in people with primary refractory disease^2–4^.

T cell-engaging therapies, including chimeric antigen receptor (CAR) T cell therapies and bispecific antibodies, are important modalities in the management of R/R DLBCL. CAR T cell treatments were initially approved in people with two or more prior therapy lines^5–10^ and have since received approval in DLBCL refractory to or relapsed after first-line immunochemotherapy^8,10–12^. The shift to earlier CAR T cell therapy necessitates the development of effective options in the third-line setting.

Bispecific antibodies, which bind T cells to a tumor antigen on cancer cells, have shown encouraging activity in solid and hematologic malignancies and are manufactured to allow off-the-shelf administration^13–15^. Recently, glofitamab and epcoritamab received accelerated US approval for DLBCL treatment after at least two prior lines of systemic therapy^16,17^.

Odronextamab is an Fc-silenced, human CD20×CD3 bispecific antibody that simultaneously engages CD20 on malignant B cells and CD3 on cytotoxic T cells to induce T cell-mediated cytotoxicity of the former^18^. The phase 1 dose-escalation and expansion ELM-1 study demonstrated encouraging activity and generally manageable safety of odronextamab monotherapy in patients with heavily pretreated B-NHL, including those with R/R DLBCL after CAR T cell therapy (at the active dose for aggressive lymphoma (≥80 mg); the objective response rate (ORR) was 33% in those who had previous CAR T cell therapy)^19^. Following further safety, pharmacokinetic and pharmacodynamic evaluation, the recommended dose for DLBCL was established as 160 mg (ref. ^19^). Here, we report the long-term efficacy and safety results of odronextamab in patients with R/R DLBCL from the phase 2 ELM-2 study (NCT03888105).

## Results

### Patient disposition and characteristics

**Extended Data Fig. 1:**
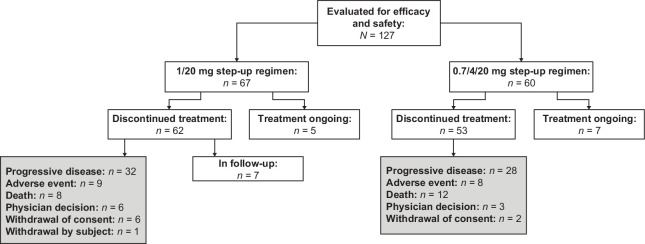
Patient disposition for the DLBCL cohort. *N* = 127 patients enrolled into the DLBCL cohort and evaluated for efficacy and safety. *n* numbers represent the number of patients who received the 1/20 mg or 0.7/4/20 mg cycle 1 step-up dosing regimens, those who discontinued treatment (and reasoning), and those with ongoing treatment at the time of data cut-off (August 18, 2023). DLBCL, diffuse large B-cell lymphoma. Source data

Between March 24, 2020 and May 18, 2022, 127 patients with DLBCL were enrolled, treated with odronextamab and evaluated for efficacy and safety. At data cutoff (August 18, 2023), the median duration of exposure was 18.0 weeks (range: 0.9–168.1) and 19 (15.0%) patients remained in the study. In total, 91.3% of patients completed cycle 1 (C1); of these, 67 (52.8%) received the C1 regimen with a step up of 1 to 20 mg, and 60 (47.2%) received the regimen with a step up of 0.7 to 4 to 20 mg. In total, 63.0% of patients completed four or more cycles of odronextamab treatment. The most common reasons for treatment discontinuation were progressive disease (PD; 47.2%), death (15.7%) and adverse events (AEs; 13.4%), occurring in a similar proportion of patients by step-up regimen (Extended Data Fig. 1).

Anti-infection prophylaxis was recommended as part of a protocol amendment; eight patients (6.3%) received prophylaxis for cytomegalovirus (CMV) infection, 84 patients (66.1%) received prophylaxis for *Pneumocystis jirovecii* pneumonia and nine patients (7.1%) received intravenous (IV) immunoglobulin prophylaxis.

At baseline, the median age of patients was 67 years (range: 24–88), with 23.6% of patients aged ≥75 years (Table 1). Most patients (81.1%) had advanced disease (Ann Arbor stages III–IV) and 55.9% had high–intermediate or high International Prognostic Index scores. A total of 31 (24.4%) patients had transformed DLBCL (24, non-Richter’s; 7, Richter’s) and 11 (8.7%) had double-hit or triple-hit cytogenetic rearrangements by local assessment. The median number of prior therapy lines was two (range: 2–8), with 20.5% of patients having received four or more prior lines, 55.1% of patients being primary refractory, 64.6% of patients being double refractory to an alkylator and anti-CD20 antibody and 17.3% of patients with prior autologous stem cell transplantation (ASCT). Baseline demographics were generally similar irrespective of step-up dosing regimen used (1 to 20 mg versus 0.7 to 4 to 20 mg) (Supplementary Table 1).

**Table 1:** Patient demographics and baseline characteristics

Characteristic	Total (*N* = 127)
Median age, years (range)	67 (24–88)
Age ≥75 years, *n* (%)	30 (23.6)
Male, *n* (%)	76 (59.8)
Race, *n* (%)	
White	61 (48.0)
Asian	53 (41.7)
Not reported	13 (10.2)
Geographic region, *n* (%)	
Asia–Pacific	64 (50.4)
Europe	52 (40.9)
North America	11 (8.7)
ECOG performance status, *n* (%)	
0	41 (32.3)
1	86 (67.7)
Ann Arbor stage, *n* (%)	
I–II	24 (18.9)
III–IV	103 (81.1)
International Prognostic Index, *n* (%)	
Low (0–1)	19 (15.0)
Low–intermediate (2)	36 (28.3)
High–intermediate (3)	41 (32.3)
High (4–5)	30 (23.6)
Missing	1 (0.8)
Cell of origin, *n* (%)	
GCB	43 (33.9)
ABC or non-GCB	56 (44.1)
Unclassified or missing	28 (22.0)
DLBCL subtype, *n* (%)	
DLBCL, de novo	96 (75.6)
DLBCL, transformed (Richter’s)	7 (5.5)
DLBCL, transformed (non-Richter’s)	24 (18.9)
DLBCL cytogenetic status, *n* (%)	
Double hit	5 (3.9)
Triple hit	6 (4.7)
Bulky disease, *n* (%)	28 (22.0)
Median prior lines of therapy (range), *n*	2 (2–8)
Prior lines of antilymphoma treatment, *n* (%)	
2	67 (52.8)
3	34 (26.8)
≥4	26 (20.5)
Prior ASCT, *n* (%)	22 (17.3)
R/R status, *n* (%)	
Primary refractory	70 (55.1)
Refractory to any prior line of therapy	115 (90.6)
Refractory to last line of therapy	110 (86.6)
Refractory to anti-CD20 antibody in any line	99 (78.0)
Refractory to an alkylator in any line	88 (69.3)
Double refractory to alkylator and anti-CD20 antibody in any line	82 (64.6)

### Efficacy

The primary endpoint of ORR per independent central review (ICR) was 52.0% (66/127 (95% confidence interval (CI): 42.9–60.9); Table 2). The complete response (CR) rate was 31.5% (40/127 (95% CI: 23.5–40.3)). The median time to response was 2.6 months (range: 0.8–6.4) and 87.9% (58/66) of patients who responded did so by their first assessment at week 12. Response rates by ICR were similar in patients who received the step-up regimen of 1 to 20 mg and in those who received the step-up regimen of 0.7 to 4 to 20 mg, with overlapping CIs for ORR, CR and partial response (PR; Supplementary Table 2). Response rates as reported by local investigator assessment were similar to those reported by ICR, with an ORR of 49.6% (63/127 (95% CI: 40.6–58.6)) and a CR rate of 38.6% (49/127 (95% CI: 30.1–47.6)).

**Table 2:** Odronextamab response summary according to ICR

Outcome	ICR (*N* = 127)
Best overall response, *n* (%)	
Objective response	66 (52.0) (95% CI: 42.9–60.9)
CR	40 (31.5) (95% CI: 23.5–40.3)
PR	26 (20.5) (95% CI: 13.8–28.5)
DOR, median, months	10.2 (95% CI: 5.0–17.9)
Probability of maintaining objective response at 12 months, %	48.1 (95% CI: 35.1–59.9)
Probability of maintaining objective response at 24 months, %	36.9 (95% CI: 24.2–49.6)
Duration of CR, median, months	17.9 (95% CI: 10.2–NE)
Probability of maintaining CR at 12 months, %	61.5 (95% CI: 44.4–74.8)
Probability of maintaining CR at 24 months, %	47.2 (95% CI: 29.7–62.9)

**Fig. 1:**
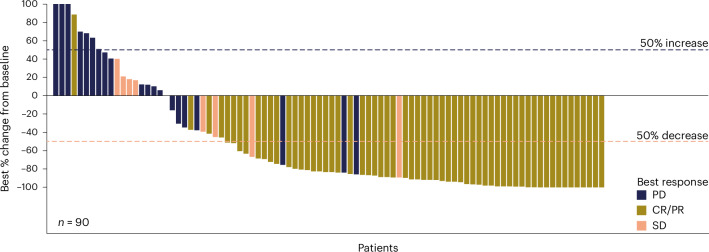
Waterfall plot of best percentage change from baseline in tumor sum of the products of the diameters. Data for each evaluable patient are shown as a separate bar on the figure (*n* = 90 patients). SD, stable disease. Source data

Tumor size was reduced in 78.9% (71/90) of patients with postbaseline imaging (Fig. 1). With a median efficacy follow-up of 29.9 months (95% CI: 20.4–32.6), the median duration of response (DOR) per ICR was 10.2 months (95% CI: 5.0–17.9). Patients with a best response of CR had a median CR duration of 17.9 months (95% CI: 10.2–not evaluable (NE)). Among the 21 patients who sustained a CR for 9 months and were eligible to transition to dosing once every 4 weeks, 18 transitioned and the median DOR from the time of transition was 18.5 months (95% CI: 6.0–NE).

**Fig. 2:**
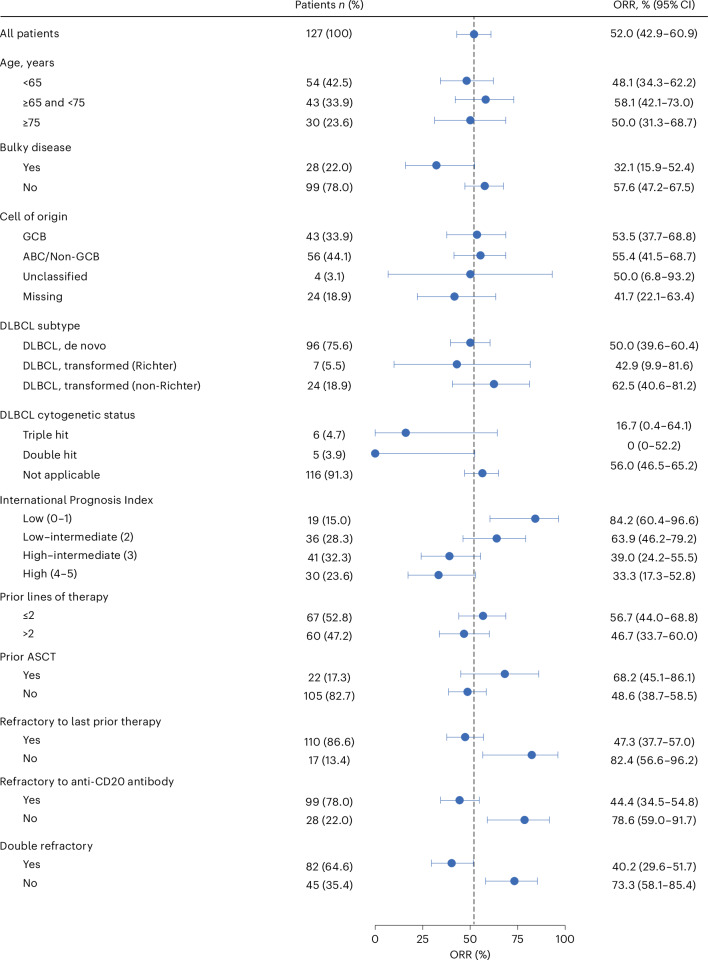
Subgroup analysis of ORR by ICR. ORR data are presented as the mean values ± 95% CIs. The vertical dashed line indicates the ORR for all patients (*N* = 127). Source data

Odronextamab demonstrated antitumor activity in patients across a range of key subgroups, including in patients aged 75 years and older (ORR = 50.0%), in those with more than two lines of prior therapy (ORR = 46.7%) and in those who were double refractory to an alkylator and anti-CD20 antibody (ORR = 40.2%) (Fig. 2).

**Fig. 3:**
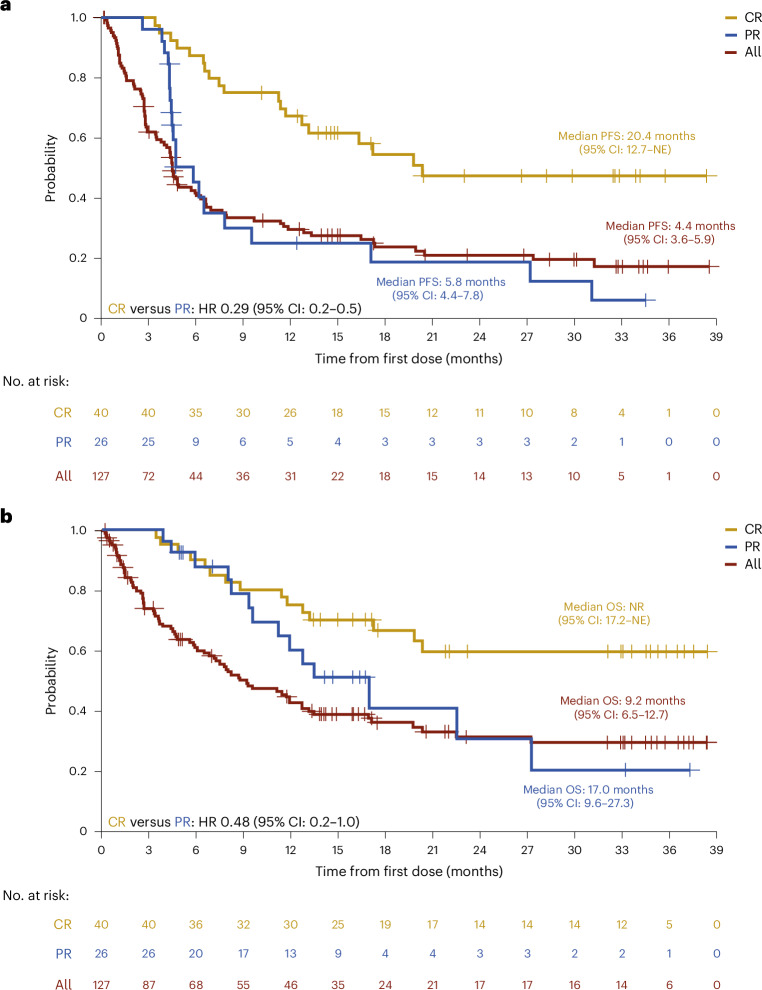
PFS and OS in patients treated with odronextamab, for all patients and by best overall response. **a**, PFS. **b**, OS. Data are presented as Kaplan–Meier curves, with tick marks indicating patients with censored data (*N* = 127 patients; *n* = 40 with CR, *n* = 26 with PR). Median values with 95% CIs are presented alongside the respective curves. Source data

The median progression-free survival (PFS) was 4.4 months (95% CI: 3.6–5.9) and median OS was 9.2 months (95% CI: 6.5–12.7). The median PFS in patients with CR (20.4 months) was longer than that in those with PR (5.8 months; hazard ratio (HR) = 0.29 (95% CI: 0.2–0.5)), as was the OS (not reached (NR) versus 17.0 months, respectively; HR = 0.48 (95% CI: 0.2–1.0)) (Fig. 3a,b).

### Biomarker assessment

**Fig. 4:**
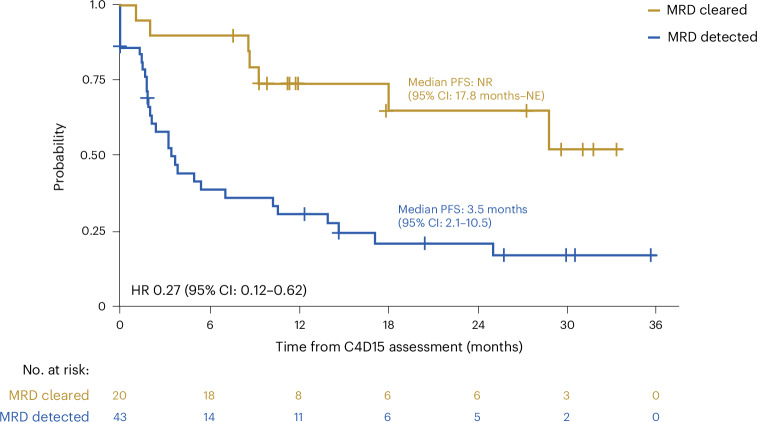
PFS by C4D15 ctDNA MRD status. Data are presented as Kaplan–Meier curves, with tick marks indicating patients with censored data (*n* = 63 ctDNA-evaluable patients; *n* = 20 with cleared MRD at C4D15, *n* = 43 with detected MRD at C4D15). Median values with 95% CIs are presented alongside the respective curves. The HR for PFS in patients with MRD cleared versus MRD detected was calculated by univariate Cox regression. Source data

**Extended Data Fig. 2:**
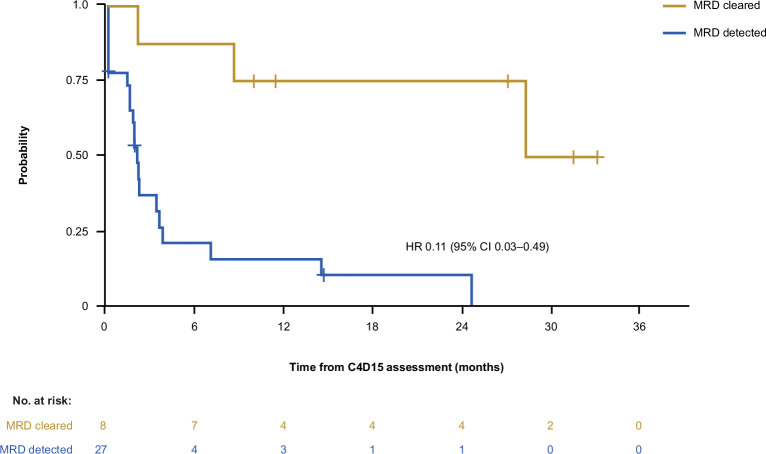
PFS by C4D15 ctDNA MRD status in patients with DLBCL and no CR at C4D15. *N* = 35 ctDNA-evaluable patients (*n* = 8 with cleared MRD at C4D15; *n* = 27 with detected MRD at C4D15). Data are presented as Kaplan–Meier curves, with tick marks indicating patients with censored data. The HR for PFS in patients with MRD cleared versus MRD detected was calculated by univariate Cox regression. C4D15, cycle 4 day 15; CI, confidence interval; CR, complete response; ctDNA, circulating tumor DNA; DLBCL, diffuse large B-cell lymphoma; HR, hazard ratio; MRD, minimal residual disease; PFS, progression-free survival. Source data

Among 63 patients evaluable for circulating tumor DNA (ctDNA) assessment who had a response assessment at C4 day 15 (C4D15), all were positive for minimal residual disease (MRD^+^) at baseline. At C4D15, 43 remained MRD^+^ and 20 were MRD^−^. PFS was longer in patients who were MRD^−^ by C4D15 versus those who were MRD^+^ (HR = 0.27; 95% CI: 0.12–0.62) (Fig. 4). MRD negativity also predicted PFS benefit in patients who did not achieve CR by positron emission tomography–computed tomography at C4D15 (MRD^−^ versus MRD^+^: HR = 0.11; 95% CI: 0.03–0.49) (Extended Data Fig. 2).

### Safety

Treatment-emergent AEs (TEAEs) were reported in 126 (99.2%) patients, with 111 (87.4%) patients experiencing at least one treatment-related (per investigator assessment) TEAE (Table 3). Overall, the most common TEAEs were cytokine release syndrome (CRS; 55.1%), pyrexia (43.3%), anemia (38.6%) and neutropenia (30.7%). Grade ≥3 TEAEs occurred in 107 (84.3%) patients, the most common being neutropenia (26.0%), anemia (22.8%), thrombocytopenia (15.0%), and coronavirus disease 2019 (COVID-19; 10.2%). Serious TEAEs occurred in 82 (64.6%) patients and were considered treatment related in 62 (48.8%) patients.

**Table 3:** Summary of AEs with odronextamab treatment

*n* (%)	Patients (*N* = 127)	
	All grades	Grade ≥3
Patients with any TEAE	126 (99.2)	107 (84.3)
Patients with any treatment-related TEAE	111 (87.4)	68 (53.5)
TEAEs occurring in ≥15% of patients (composite, preferred term)		
CRS^a^	70 (55.1)	6 (4.7)
Pyrexia	55 (43.3)	5 (3.9)
Anemia	49 (38.6)	29 (22.8)
Neutropenia	39 (30.7)	33 (26.0)
Diarrhea	28 (22.0)	2 (1.6)
Cough	26 (20.5)	0 (0)
Thrombocytopenia	24 (18.9)	19 (15.0)
Hypokalemia	24 (18.9)	11 (8.7)
Infusion-related reaction^b^	23 (18.1)	0 (0)
Fatigue	21 (16.5)	3 (2.4)
COVID-19	21 (16.5)	13 (10.2)
Nausea	19 (15.0)	0 (0)
Infections (system organ class)	82 (64.6)	49 (38.6)
Neurologic AEs^c^	54 (42.5)	5 (3.9)
Serious TEAE	82 (64.6)	
Treatment-related serious TEAE	62 (48.8)	
TEAE leading to treatment discontinuation	17 (13.4)	
Treatment-related TEAE leading to treatment discontinuation	12 (9.4)	
TEAE leading to dose interruption/delay	92 (72.4)	
Treatment-related TEAE leading to dose interruption/delay	67 (52.8)	
TEAE leading to death	20 (15.7)	
Treatment-related TEAE leading to death	5 (3.9)	

^a^CRS data are reported for both C1 step-up dosing regimens combined.

^b^Two cases of infusion-related reaction were related to IV immunoglobulin infusions rather than to treatment.

^c^Events included psychiatric disorders and nervous system disorders.

A total of 17 (13.4%) patients had TEAEs that led to treatment discontinuation. Treatment-related TEAEs leading to discontinuation were encephalopathy (*n* = 2), CRS, COVID-19, CMV reactivation, pulmonary tuberculosis, aphasia, supraventricular tachycardia, and cholangitis sclerosing (*n* = 1 each); CRS, tachycardia, pancreatitis, septic shock, pneumonia plus cough in one patient; and *P.* *jirovecii* plus neutrophil count decrease in one patient. TEAEs leading to death were reported in 20 (15.7%) patients (Supplementary Table 3); these were considered treatment related in five (3.9%) patients (COVID-19, pneumonia, *P.* *jirovecii* pneumonia, pseudomonal sepsis (*n* = 1 each), and CMV pneumonia plus CMV reactivation in one patient).

**Extended Data Fig. 3:**
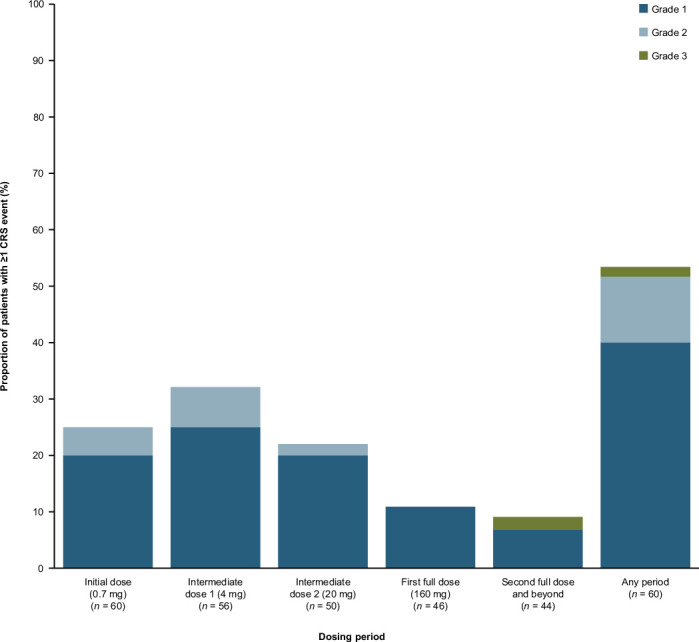
Treatment-emergent CRS by dose and severity grade with the 0.7/4/20 mg regimen. The percentage of patients in the 0.7/4/20 mg regimen cohort who experienced at least one CRS event are shown according to CRS grade (1, 2, or 3). *n* numbers represent the number of patients treated at each of the dose points shown. CRS, cytokine release syndrome. Source data

With the step-up regimen of 0.7 to 4 to 20 mg, CRS was reported in 32/60 (53.3%) patients and mostly occurred during C1 (Extended Data Fig. 3). With this regimen, the majority of events were of low grade (grade 1, 40%; grade 2, 11.7%), with one grade 3 case occurring in the setting of acute pancreatitis (pancreatic lymphoma mass causing obstruction of biliary drainage) at week 6; this was numerically lower than the rate of grade 3 CRS with the original step-up regimen of 1 to 20 mg (*n* = 5 (7.5%)) (Supplementary Table 4). There were no cases of grade 4 or grade 5 CRS. CRS was managed with tocilizumab in 15 (25.0%) patients and systemic steroids in 13 (21.7%) patients (Supplementary Table 5); no patients required mechanical ventilation or intensive care unit admission for CRS. The median time to onset of CRS was 18.0 h (range: −3.4 to 221.0) and CRS events resolved in a median of 7.7 h (range: 0.1–143.9). Infusion-related reactions occurred in five (8.3%) patients (all grade 1 or 2). Two cases of encephalopathy that led to discontinuation of odronextamab treatment occurred in the setting of CRS during step-up dosing: one grade 3 event with the step-up regimen of 1 to 20 mg in a 79-year-old patient, and one grade 2 event with the step-up regimen of 0.7 to 4 to 20 mg in an 85-year-old patient; both events resolved with steroids.

Neurologic AEs of any grade occurred in 54 (42.5%) patients (grade ≥3, five (3.9%) patients), including in 22 (36.7%) patients with the step-up regimen of 0.7 to 4 to 20 mg (all grade 1 or 2). Neurologic AEs reported in >5% of patients were insomnia (*n* = 20 (15.7%)), dizziness (*n* = 12 (9.4%)) and headache (*n* = 8 (6.3%)). No cases of immune effector cell-associated neurotoxicity syndrome (ICANS; preferred term) were reported with either regimen. Tumor lysis syndrome occurred in one patient (grade 3–4) with the step-up regimen of 1 to 20 mg (Supplementary Table 4). There was one case of low-grade tumor flare.

Infections occurred in 82/127 (64.6%) patients (grade ≥3, 38.6%). The most frequent type of infection was COVID-19, which was reported in 18.1% (grade ≥3, 12.6%) of patients. Febrile neutropenia was also observed in three (2.4%) patients. Overall, six (4.7%) patients discontinued treatment because of treatment-related infections. Grade 5 infection occurred in 15 patients, with five cases because of COVID-19. Other grade 5 infections included pneumonia and sepsis (*n* = 3 each), *P.* *jirovecii* pneumonia, CMV infection, and pseudomonal sepsis (*n* = 1 each), and CMV infection reactivation plus CMV pneumonia in one patient.

## Discussion

In this phase 2 study, odronextamab monotherapy demonstrated substantial efficacy in heavily pretreated patients with R/R DLBCL. These results are consistent with those from the ELM-1 study in patients with R/R DLBCL who had received prior CAR T cell therapy and no new safety signals were observed^19,20^, indicating that odronextamab may have an important role in maintaining effective disease control in this aggressive lymphoma.

Overall, the baseline characteristics of enrolled patients were representative of a heavily pretreated, highly refractory population. High-risk factors included double-hit and triple-hit cytogenetic rearrangements (9% of patients), transformed disease (24%), age ≥ 75 years (24%), Ann Arbor stage III–IV (81%) and prior ASCT (17%). Despite the difficult-to-treat nature of this population, odronextamab demonstrated consistent ORRs across high-risk subgroups.

T cell-engaging therapies including CAR T cell therapies are now established as an important treatment option for people with R/R DLBCL^2^. Despite encouraging ORRs (52–82%), eligibility for CAR T cell therapy is low (6–22%) and uptake among eligible people is variable because of access barriers (for example, administration complexities, manufacturing timelines and costs), associated toxicities (including CRS, ICANS and prolonged cytopenias) and potential risk of secondary malignancy^5–7,21–25^. These challenges may underpin the less frequent use of CAR T cell therapies reported in elderly people^26^. Potential earlier use of CAR T cell therapy also highlights the need for therapies that are active after these agents, where outcomes are typically dismal^27^. Bispecific antibodies have, thus, assumed greater importance as an alternative treatment option, particularly in the third line following the recent approvals of axicabtagene ciloleucel and lisocabtagene maraleucel for people with DLBCL who are R/R within 12 months of first-line immunochemotherapy^8,10^. Although cross-study comparisons of different single-arm studies are challenging, in the third-line setting, odronextamab demonstrated ORR and CR rates of 52% (95% CI: 42.9–60.9) and 31% (95% CI: 23.5–40.3), respectively, similar to those across the field of bispecific antibodies in R/R DLBCL (glofitamab, 52% (95% CI: 43–60) and 39% (95% CI: 32–48); epcoritamab, 63% (95% CI: 55.0–70.6) and 39% (95% CI: 31.2–46.9), respectively)^28,29^. Although this study did not include people treated with prior CAR T cell therapy, the efficacy of odronextamab in this population is supported by data from a prospective cohort of patients with disease progression after CAR T cell therapy in ELM-1 (*n* = 44), where the ORR was 48% (CR rate, 30%) and median DOR was NR after a median efficacy follow-up of 4.9 months. These data are consistent with those reported in CAR T cell therapy-naive patients in the current study and there were no major differences in safety profile^20^.

Biomarker assessment revealed MRD clearance in 20/63 patients at C4D15 of odronextamab treatment, with improved PFS in patients with cleared versus detectable MRD. The observed association between MRD clearance and PFS benefit, even in patients without positron emission tomography–computed tomography CR, indicates the prognostic utility of MRD measurement at this early time point and supports further investigation at later stages of treatment and potentially even after treatment.

The odronextamab administration schedule involved step-up dosing during C1, which was optimized during the study to help mitigate the risk of CRS. The step-up regimens used differed by just 1 week before reaching full dose and pharmacokinetic data indicated that exposure levels were similar for both regimens after the first full dose was received^30^. Following step-up, patients received weekly odronextamab in C2–C4, before dosing once every 2 weeks (once every 4 weeks with durable CR) as maintenance treatment until disease progression or unacceptable toxicity. This treatment regimen provided compelling antitumor control while maintaining or improving patient-reported outcomes over 42 weeks^31^. Alternative treatment paradigms, including fixed duration, have been explored in this setting^28,29^ but the optimal treatment approach for R/R DLBCL is yet to be determined. The potential for growth of subclonal cell populations supports treatment to progression^32–34^ in highly refractory people with aggressive lymphoma and appeared feasible. In addition, administration frequency could be reduced to once every 4 weeks in patients with a durable CR, enabling continued antitumor control in the context of reduced treatment burden.

Among the key AEs associated with bispecific antibodies, severe CRS risk was generally mitigated with the optimized C1 step-up regimen, with a numerically lower rate of grade 3 CRS compared with the original step-up regimen of 1 to 20 mg. One case of grade 3 CRS occurred with the revised regimen, although this was confounded by concurrent acute pancreatitis. Grade ≥3 CRS was reported in 4% (6/154) and 2.5% (4/157) of patients treated with glofitamab and epcoritamab, respectively^28,29^. Tocilizumab and corticosteroids for CRS were given to 25% and 22% of patients, respectively, in the current study according to evolving institutional guidelines^35–38^, although no patients required ventilatory or intensive care unit support. Given the low incidence of severe CRS, ongoing studies are evaluating odronextamab dosing in the outpatient setting, an important consideration for promoting equitable access to effective treatment options in underserved communities. No ICANS was reported with odronextamab in contrast to glofitamab (8%) and epcoritamab (6%)^28,29^. Neurologic AEs occurred in 43% of patients treated with odronextamab, similar to rates reported with glofitamab in R/R B-NHL (40%) and epcoritamab in R/R DLBCL (35%)^39,40^. Neurologic AEs were mostly grade ≤2 with odronextamab and the events observed were generally consistent with those reported with other bispecific antibodies^16,40^. Two cases of grade 2–3 encephalopathy that led to treatment discontinuation were reported in elderly patients; however, both events occurred in the setting of CRS during step-up dosing and resolved with steroids.

Infections were observed in 65% of patients, which may be common in a population with impaired B cell functionality because of underlying malignancy, prior exposure to immunosuppressive agents and chemotherapy, and anticipated B cell depletion and hypogammaglobulinemia induced by odronextamab^41,42^. Anti-infection prophylaxis was added to the protocol during the study, although local practices for infection management and IV immunoglobulin supplementation may have differed between global sites. COVID-19 was the most frequent infection reported and the most frequent grade 5 infection, reflecting the course of ELM-2 enrollment. Enrollment began early in the pandemic when viral severity and mortality were high and no vaccines or anti-COVID-19 treatments were available. Later enrollment occurred when more transmissible variants were prevalent, vaccine availability had improved and social-distancing measures were relaxing. Randomized controlled trials are required to further investigate the risk of infections with odronextamab and better characterize the kinetics of B cell depletion following fixed durations of treatment.

TEAEs resulting in death were reported in 15.7% of patients treated with odronextamab, of which five (3.9%) were because of COVID-19 infection. With glofitamab and epcoritamab, TEAEs leading to death were reported in approximately 5% of patients, which were related to COVID-19 in five (3.2%) and two (1.3%) patients, respectively^28,29^. Most fatal TEAEs were caused by infections. The differences in fatal infection rates may be attributed to variations in populations, regional infection rates and local supportive care practices. In addition, the timing of enrollment during different phases of the COVID-19 pandemic, including the availability of treatments and vaccines, may have influenced outcomes.

In conclusion, odronextamab demonstrated highly encouraging clinical activity, including durable CRs, in heavily pretreated, highly refractory patients with R/R DLBCL. AEs were experienced by nearly all patients treated with odronextamab. However, these events were generally manageable with supportive care measures. Odronextamab is a potential treatment option for people with highly refractory R/R DLBCL. Phase 3 trials are currently enrolling in earlier lines of therapy and will inform the future management paradigm for aggressive lymphomas.

## Methods

### Study design and patients

ELM-2 is an ongoing phase 2, open-label, multicohort, multicenter, single-arm study of odronextamab monotherapy in R/R B-NHL (ClinicalTrials.gov identifier: NCT03888105). Here, we report long-term follow-up results of the primary analysis in the cohort of patients with R/R DLBCL. Patients were recruited from various centers across multiple countries, including the USA, Australia, Canada, China, France, Germany, Italy, Japan, the Republic of Korea, Poland, Singapore, Spain, Taiwan and the United Kingdom. Methods for the follicular lymphoma cohort of ELM-2, which used the same endpoints as the DLBCL cohort, have been published^43^. Eligible patients for the DLBCL cohort were aged ≥18 years with DLBCL (de novo or transformed) refractory to or relapsed after two or more prior lines of systemic therapy, including an anti-CD20 antibody and an alkylator. Other inclusion criteria were measurable disease on cross-sectional imaging, Eastern Cooperative Oncology Group (ECOG) performance status 0–1, and adequate bone marrow and hepatic functions. Patients with high-grade lymphoma (double-hit and triple-hit cytogenetic rearrangements) were accepted. People with primary central nervous system lymphoma or prior ASCT, CAR T cell therapy or CD20×CD3 bispecific antibody treatment were excluded.

Measures to ensure diverse and inclusive enrollment included diverse trial sites, translated consent forms for under-represented populations, extended screening windows for patients with access constraints, broad eligibility criteria to include patients with controlled human immunodeficiency virus, hepatitis B and hepatitis C infection, and lower thresholds for those with compromised organ function because of lymphoma.

Prophylaxis for *P.* *jirovecii* pneumonia was recommended for all patients. Other anti-infection prophylaxis measures included IV immunoglobulin supplementation and antivirals, in accordance with the protocol and local institutional standard, as well as the National Comprehensive Cancer Network^44^, American Society of Clinical Oncology^45^ or European Society for Medical Oncology guidelines^46^. In patients with severe hypogammaglobulinemia (<400 mg dl^−1^) or in patients with recurrent episodes of infection with immunoglobulin levels between 400 and 600 mg dl^−1^, supplementation with IV immunoglobulin was recommended. For patients with positive hepatitis B surface antigens, hepatitis B core antibodies and/or measurable viral load, an appropriate antiviral agent for hepatitis B virus was recommended. Appropriate antiviral prophylaxis was recommended for patients with prior herpes simplex virus or CMV infection.

Patients received IV odronextamab in 21-day cycles. The original step-up regimen (1 to 20 mg) during C1 comprised a dose of 1 mg split over day 1 (0.5 mg) and day 2 (0.5 mg) and 20 mg split over day 8 (10 mg) and day 9 (10 mg), followed by the full dose of 160 mg on day 15. The step-up dosing regimen was optimized during the study to further mitigate the risk of CRS by reducing the initial dose and adding an intermediary dose. The revised step-up regimen of 0.7 to 4 to 20 mg regimen consisted of 0.7 mg split over day 1 (0.2 mg) and day 2 (0.5 mg), 4 mg split over day 8 and day 9 and 20 mg split over day 15 and day 16 of C1. Following C1 step-up dosing, patients received odronextamab 160 mg on days 1, 8 and 15 of C2–C4 and then 320 mg once every 2 weeks as maintenance until disease progression or another protocol-defined reason for treatment discontinuation. Patients were admitted for inpatient monitoring for 24 h following each infusion up to and including C2D1.

In patients who had a CR that lasted for 9 months or longer by investigator evaluation, the frequency of dosing was reduced to 320 mg once every 4 weeks.

Premedication with dexamethasone, diphenhydramine and acetaminophen was given during C1 step-up dosing to help mitigate the risk of CRS. All patients received 20 mg of IV dexamethasone 1–3 h before each split or single infusion dose for both regimens. Patients on the step-up regimen of 0.7 to 4 to 20 mg also received 10 mg of oral dexamethasone 12–24 h before the first split infusion and 25 mg of IV diphenhydramine and 650 mg of oral acetaminophen 30–60 min before each split or single infusion. Patients then received 10 mg of oral dexamethasone 24 h after the second split infusion or first single infusion. Premedication was continued until the patient received the full weekly dose without experiencing infusion-related reactions or CRS. Patients who developed symptoms consistent with severe CRS were considered for treatment with tocilizumab, corticosteroid and other interventions according to the clinical judgment of the investigator.

The protocol and amendments were approved by the relevant institutional review boards and ethics committees (Supplementary Table 6). The study protocol is included in the Supplementary Information. The study was conducted according to applicable regulatory requirements, guidelines of Good Clinical Practice as specified by the International Conference on Harmonization and principles originating from the Declaration of Helsinki. All patients provided written informed consent before enrollment. Where possible, the present report was developed in accordance with CONSORT reporting guidelines^47^. Further information on research design is available in the Nature Research Reporting Summary linked to this article.

### Endpoints and assessments

The primary endpoint was ORR, assessed by ICR and in accordance with Lugano criteria^48^. Secondary endpoints included ORR assessed by local investigator, CR rate, DOR, PFS, OS and patient-reported quality-of-life outcomes.

Disease assessments using computed tomography/magnetic resonance imaging and positron emission tomography were performed during screening, at week 12 and then every 8 weeks in year 1, every 12 weeks in year 2 and during follow-up as described in the protocol.

Exploratory endpoints included changes in select cytokine levels and MRD status using ctDNA, with samples taken at baseline, at week 12 and at every radiologic response assessment in patients with CR. A modified AVENIO ctDNA analysis workflow (Roche; research only) was used for next-generation sequencing according to the cancer personalized profiling obtained by deep sequencing^49^. Whole-blood-cell pellets were used to filter out germline allele variants and MRD negativity was reported when the *P* value for allele frequency was greater than 0.005 (ref. ^50^). The study was not powered for statistical testing of MRD analyses given their exploratory nature.

Safety and tolerability were assessed until 90 days after the last dose of study drug or initiation of another antilymphoma therapy, with AEs graded according to the National Cancer Institute Common Terminology Criteria for AEs (version 5). CRS grading was adapted from American Society for Transplantation and Cellular Therapy guidelines^51^. TEAE and treatment-related TEAE data are presented. TEAEs were defined as AEs that newly occurred or worsened during the on-treatment period and any treatment-related serious AEs that occurred during the post-treatment period. TEAEs were deemed treatment related by the investigator.

### Statistics and reproducibility

In the ELM-2 study of B-NHL, approximately 512 patients were planned for enrollment into five disease-specific cohorts (DLBCL, follicular lymphoma, marginal zone lymphoma, mantle cell lymphoma and ‘other B-NHL’). This report included all patients in the DLBCL global cohort (160 mg once weekly or 320 mg once every 2 weeks), with no data excluded. Data distribution was assumed to be normal, but not formally tested.

An exact binomial design was adopted for the primary endpoint of ORR. The two-sided 95% CIs for the observed ORR were calculated on the basis of a sample size of 112. Assuming a clinically meaningful ORR as being greater than 35%, with 112 patients, an ORR of at least 45% would have a lower CI bound that excludes 35%. In addition, if the observed ORR was at least 50%, 55% or 60%, the lower bound of the 95% CI would exclude an ORR of 40%, 45% and 50%, respectively. With a sample size of 112 patients, if the true treatment effect of odronextamab was 50%, the probability of the observed lower bound of 95% CI excluding 35% was 89%. Enrollment was increased to include at least 60 patients treated with a step-up regimen of 0.7 to 4 to 20 mg, and up to 127 patients with 160 mg of weekly dosing. The step-up dosing regimen allocation for patients was nonrandomized and unblinded.

Patients NE for best overall response were considered nonresponders. The primary analysis for the primary endpoint was performed after all patients had completed 36 weeks of tumor assessment or withdrawn from study. Efficacy and safety analyses were performed in all patients who received odronextamab. DOR, PFS and OS were analyzed using Kaplan–Meier estimation. Data collection and analysis were not performed blind to the conditions in the experiments.

All analyses were performed using SAS (SAS Institute) version 9.4 or above. The statistical analysis plan is included in the Supplementary Information.

### Subgroup analysis

Patient demographics were summarized for the DLBCL cohort, including age (<65 years, 65–75 years, and ≥65 years), sex (male or female; self-reported by patients), race (White, Black or African American, American Indian or Alaska native, native Hawaiian or other Pacific Islander, not reported, unknown, or other) and ethnicity (Hispanic or Latino, or not Hispanic or Latino).

ORR per ICR was analyzed in subgroups of patients with DLBCL defined by baseline characteristics, including age, cell of origin (germinal center B cell-like (GCB) DLBCL, activated B cell-like (ABC) DLBCL/non-GCB, or unclassified DLBCL) and cytogenetic status (triple hit or double hit). If a subgroup included fewer than ten patients, the analysis for the given subgroup was not performed or combined with another subgroup.

### Reporting summary

Further information on research design is available in the Nature Portfolio Reporting Summary linked to this article.

## Supplementary information

Supplementary Information Redacted Study Protocol and redacted Statistical Analysis Plan.

Reporting Summary

CONSORT checklist Completed CONSORT 2010 checklist.

CONSORT abstract checklist Completed CONSORT 2010 abstract checklist.

REMARK checklist Completed REMARK checklist.

Supplementary Tables 1–6 Supplementary Table 1: Patient demographics and baseline characteristics of patients who received the step-up dosing regimens of 1 to 20 mg or 0.7 to 4 to 20 mg (*n* = number of patients). Supplementary Table 2: Best overall responses of patients who received the step-up dosing regimens of 1 to 20 mg or 0.7 to 4 to 20 mg, according to ICR (*n* = number of patients). Supplementary Table 3: TEAEs resulting in death among 127 patients treated with odronextamab (*n* = number of events). *CMV reactivation and CMV pneumonia were reported as the cause of death in one patient. Supplementary Table 4: AEs of special interest of any grade or grade ≥3 in patients treated with the step-up dosing regimens of 1 to 20 mg or 0.7 to 4 to 20 mg (*n* = number of events). *Events included psychiatric disorders and nervous system disorders; ^†t^reatment related. Supplementary Table 5: Rates of CRS and CRS management approaches among 60 patients treated with the C1 step-up regimen of 0.7 to 4 to 20 mg (*n* = number of patients). Supplementary Table 6: Countries, names and site numbers of institutional review boards and ethics committees who approved the ELM-2 study protocol and amendments. HREC, Health Research Ethics Committee; IRB, Institutional Review Board; MEC, Medical Ethics Committee; WCG, Western Institutional Review Board—Copernicus Group.

## Source data

Source Data Fig. 1 Statistical source data.

Source Data Fig. 2 Statistical source data.

Source Data Fig. 3 Statistical source data.

Source Data Fig. 4 Statistical source data.

Source Data Extended Data Fig. 1 Statistical source data.

Source Data Extended Data Fig. 2 Statistical source data.

Source Data Extended Data Fig. 3 Statistical source data.

## Data Availability

Patient personal data will be treated in compliance with all applicable laws and regulations. The sponsor shall take all appropriate measures to safeguard and prevent access to these data by any unauthorized third party. Qualified researchers can request access to study documents (including the clinical study report, study protocol with any amendments, blank case report form and statistical analysis plan) that support the methods and findings in this paper. Individual anonymized patient data will be considered for sharing (1) once the product and indication have been approved by major health authorities (the U.S. Food and Drug Administration, European Medicines Agency, Pharmaceuticals and Medical Devices Agency, etc.) or development of the product has been discontinued globally for all indications on or after April 2020 and there are no plans for future development; (2) if there is legal authority to share the data; and (3) if there is not a reasonable likelihood of patient reidentification. Requests should be submitted to https://vivli.org/. Once the criteria for data availability have been fulfilled, the time frame from data request to access of data will be approximately 1–6 months. Source data are provided with this paper.
