# Author Correction: Odronextamab monotherapy in patients with relapsed/refractory diffuse large B cell lymphoma: primary efficacy and safety analysis in phase 2 ELM-2 trial

**DOI:** 10.1038/s43018-025-00967-6

**Published:** 2025-04-16

**Authors:** Won Seog Kim, Tae Min Kim, Seok-Goo Cho, Isidro Jarque, Elżbieta Iskierka-Jażdżewska, Li Mei Poon, H. Miles Prince, Huilai Zhang, Junning Cao, Mingzhi Zhang, Benoît Tessoulin, Sung Yong Oh, Francesca Lim, Cecilia Carpio, Tran-Der Tan, Sabarish Ayyappan, Antonio Gutierrez, Jingxian Cai, Melanie Ufkin, Saleem Shariff, Jurriaan Brouwer-Visser, Aafia Chaudhry, Hesham Mohamed, Srikanth Ambati, Jan Walewski, on behalf of the ELM-2 Investigators, Hannah Rose, Geoffrey Chong, Vinod Ganju, Michael Chu, Mary-Margaret Keating, Yuqin Song, Jun Zhu, Xiaoyan Ke, Shuhua Yi, Huilai Zhang, Qingyuan Zhang, Liqun Zou, Mingzhi Zhang, Dengju Li, Wenbin Qian, Ou Bai, Li Gao, Jie Jin, Caixia Li, Huiqiang Huang, Zheng Wei, Youhua Chen, Pengcheng He, Gandhi Laurent Damaj, Kamal Bouabdballah, Emmanuel Bachy, Corinne Haioun, Franck Morschhauser, Sylvain Choquet, Vincent Delwail, Catherine Thieblemont, Johannes Duell, Thomas Weber, Paul Graf La Rosee, Holger Hebart, Enrico Capochiani, Vittorio Zilioli, Francesca Rossi, Stefano Luminari, Pier Luigi Zinzani, Laura Bagnato, Gianluca Gaidano, Marco Brociner, Cristina Skert, Monica Tani, Roberta Battistini, Leonardo Flenghi, Ryusuke Yamamoto, Kunihiro Tsukasaki, Kenichi Ishizawa, Tomomi Tobai, Toshiki Uchida, Yosuke Minami, Nobuhiko Yamauchi, Junichiro Yuda, Masahiro Takeuchi, Hirokazu Nagai, Youko Suehiro, Yoshiaki Ogawa, Junya Kuroda, Tatsuro Jo, Hirohisa Nakamae, Isao Yoshida, Michal Taszner, Ewa Lech-Maranda, Wanda Knopinska-Posluszny, Tomasz Wrobel, Tadeusz Robak, Wen Son Hsieh, Shin Yeu Ong, Hyeon-Seok Eom, Yeung-Chul Mun, Young Rok Do, Jin Seok Kim, Byung Soo Kim, Jae-Cheol Jo, Ana Jimenez-Ubieto, Rafael Andreu, Alejandro Martin, Agustin Penedo Coello, Raul Cordoba, Aranzazu Alonso, Laura Magnano, Eva Gonzalez-Barca, Sara Miqueleiz, Tsai Yun Chen, Su Peng Yeh, Shang-Ju Wu, Ming-Chung Wang, David Cunningham, Andrea Kuhnl, David Tucker, David Lewis, Nagah Elmusharaf, John Allan, Thomas Jandl, Sami Ibrahimi, Deepa Jagadeesh, Lori Leslie, Parameswaran Venugopal, Jon Arnason, Jose C. Villasboas, Rakhee Vaidya, Don Stevens, Farrukh Awan, Andreas Klein, Umar Farooq

**Affiliations:** 1 Sungkyunkwan University School of Medicine, Samsung Medical Center Division of Hematology–Oncology, Seoul, South Korea; 2 Seoul National University Hospital, Seoul National University Cancer Research Institute, Seoul, South Korea; 3 Seoul St. Mary’s Hospital, The Catholic University of Korea, Seoul, South Korea; 4 Hematology Department, Hospital Universitari i Politècnic La Fe, Centro de Investigación Biomédica en Red de Cáncer—CIBERONC, Valencia, Spain; 5 Copernicus Memorial Hospital, Department of General Hematology, Medical University of Łódź, Łódź, Poland; 6 Department of Haematology-Oncology, National University Cancer Institute, National University Hospital, Singapore, Singapore; 7 Epworth HealthCare and University of Melbourne, Melbourne, Victoria Australia; 8 Department of Lymphoma, Tianjin Medical University Cancer Institute and Hospital, National Clinical Research Center for Cancer, Key Laboratory of Cancer Prevention and Therapy, Tianjin, China; 9 Fudan University Shanghai Cancer Center, Shanghai, China; 10 Department of Oncology, First Affiliated Hospital, Zhengzhou University, Zhengzhou, China; 11 Hematology Department, Nantes University Hospital, Nantes, France; 12 Dong-A University Hospital, Busan, South Korea; 13 Singapore General Hospital, Singapore, Singapore; 14 Department of Hematology, Vall d’Hebron Institute of Oncology (VHIO), University Hospital Vall d’Hebron, Autonomous University of Barcelona (UAB), Barcelona, Spain; 15 Hematology and Medical Oncology, Koo Foundation Sun Yat Sen Cancer Center, Taipei City, Taiwan; 16 University of Iowa Hospital and Clinics, Iowa City, IA USA; 17 Department of Hematology, Hospital Universitario Son Espases, IdISBa, Palma, Spain; 18 Regeneron Pharmaceuticals, Inc., Tarrytown, NY USA; 19 Regeneron UK, Ltd., Uxbridge, UK; 20 Narodowy Instytut Onkologii im. Marii Skłodowskiej-Curie Państwowy Instytut Badawczy, Warszawa, Poland; 21 University Hospital Geelong, Geelong, Victoria Australia; 22 Austin Health, Heidelberg, Victoria Australia; 23 Hudson Institute of Medical Research, Melbourne, Victoria Australia; 24 Cross Cancer Institute, University of Alberta, Edmonton, Alberta Canada; 25 Dalhousie University, Halifax, Nova Scotia Canada; 26 Peking University Cancer Hospital and Institute, Beijing, China; 27 Lymphoma Research Center, Peking University Third Hospital, Beijing, China; 28 National Clinical Research Center for Blood Diseases, Institute of Hematology and Blood Diseases Hospital, Chinese Academy of Medical Sciences and Peking Union Medical College, Tianjin, China; 29 Tianjin Institutes of Health Science, Tianjin, China; 30 Tianjin’s Clinical Research Center for Cancer, National Clinical Research Center for Cancer, Tianjin Medical University Cancer Institute and Hospital, Tianjin Medical University, Tianjin, China; 31 Harbin Medical University Cancer Hospital, Harbin, China; 32 Cancer Center, West China Hospital of Sichuan University, Chengdu, China; 33 The First Affiliated Hospital of Zhengzhou University, Zhengzhou, China; 34 Tongji Hospital, Tongji Medical College, Huazhong University of Science and Technology, Wuhan, China; 35 The Second Affiliated Hospital, College of Medicine, Zhejiang University, Hangzhou, China; 36 The First Hospital of Jilin University, Changchun, China; 37 Children’s Hospital of Soochow University, Suzhou, China; 38 The First Affiliated Hospital of Zhejiang University School of Medicine, Hangzhou, China; 39 Zhejiang Provincial Clinical Research Center for Hematological Disorders, Hangzhou, China; 40 The First Affiliated Hospital of Soochow University, Suzhou, China; 41 Sun Yat-sen University Cancer Center, Guangzhou, China; 42 Zhongshan Hospital, Fudan University, Shanghai, China; 43 Renmin Hospital of Wuhan University, Hubei General Hospital, Wuhan, China; 44 The First Affiliated Hospital of Xi’an Jiaotong University, Xian, China; 45 Caen University Hospital, Caen, France; 46 CHU De Bordeaux, Bordeaux, France; 47 Hôpital Lyon Sud, Hospices Civils de Lyon, Lyon, France; 48 CHU Henri Mondor, Créteil, France; 49 CHU de Lille, Hôpital Claude Huriez, Lille, France; 50 Pitié-Salpêtrière University Hospital, APHP, Sorbonne Université, Paris, France; 51 CHU Poitiers, Poitiers, France; 52 Hôpital Saint-Louis, Paris, France; 53 University Hospital of Würzburg, Würzburg, Germany; 54 Martin Luther University Halle-Wittenberg, Halle, Germany; 55 Schwarzwald-Baar Klinikum, Villingen-Schwenningen, Germany; 56 Stauferklinikum Schwäbisch Gmünd, Mutlangen, Germany; 57 Azienda Unità Sanitaria Locale (USL) Toscana Nord Ovest, Livorno, Italy; 58 ASST Grande Ospedale Metropolitano Niguarda, Milan, Italy; 59 UOC Ematologia-Fondazione IRCCS Cà Granda OM Policlinico, Milan, Italy; 60 Azienda Unità Sanitaria Locale-IRCCS, Reggio Emilia, Italy; 61 University of Modena and Reggio Emilia, Reggio Emilia, Italy; 62 IRCCS Azienda Ospedaliero-Universitaria di Bologna, Istituto di Ematologia ‘Seràgnoli’, Bologna, Italy; 63 Università di Bologna, Bologna, Italy; 64 Università Del Piemonte Orientale, Novara, Italy; 65 Ospedale di Circolo e Fondazione Macchi-ASST Sette Laghi, Varese, Italy; 66 Ospedale Dell’Angelo, Venice, Italy; 67 Santa Maria delle Croci Hospital, Ravenna, Italy; 68 Azienda Ospedaliera San Camillo, Rome, Italy; 69 Azienda Ospedaliera di Perugia, Perugia, Italy; 70 Kobe City Medical Center General Hospital, Kobe, Japan; 71 International Medical Center, Saitama Medical University, Saitama, Japan; 72 Yamagata University, Yamagata, Japan; 73 University of Michigan, Comprehensive Cancer Center, Ann Arbor, MI USA; 74 Japanese Red Cross Aichi Medical Center Nagoya Daini Hospital, Nagoya, Japan; 75 National Cancer Center Hospital East, Kashiwa, Japan; 76 Cancer Institute Hospital, Japanese Foundation for Cancer Research, Tokyo, Japan; 77 Chiba Cancer Center, Chiba, Japan; 78 National Hospital Organization Nagoya Medical Center, Nagoya, Japan; 79 National Organization Kyushu Cancer Center, Fukuoka, Japan; 80 Tokai University School of Medicine, Kanagawa, Japan; 81 Kyoto Prefectural University of Medicine, Kyoto, Japan; 82 Japanese Red Cross Nagasaki Genbaku Hospital, Nagasaki, Japan; 83 Osaka Metropolitan University Hospital, Osaka, Japan; 84 Shikoku Cancer Center, Matsuyama, Japan; 85 Medical University of Gdansk, Gdansk, Poland; 86 Institute of Hematology and Transfusion Medicine, Warsaw, Poland; 87 The Sea Hospital in Gdynia, Gdynia, Poland; 88 Wroclaw Medical University, Wroclaw, Poland; 89 Copernicus Memorial Hospital, Medical University of Lodz, Lodz, Poland; 90 ICON Cancer Centre, Singapore, Singapore; 91 National Cancer Center, Goyang, Republic of Korea; 92 Ewha Womans University School of Medicine, Seoul, Republic of Korea; 93 Dongsan Medical Center, Daegu, Republic of Korea; 94 Yonsei University College of Medicine, Seoul, Republic of Korea; 95 Korea University College of Medicine, Seoul, Republic of Korea; 96 Ulsan University Hospital, University of Ulsan College of Medicine, Ulsan, Republic of Korea; 97 Hospital Universitario 12 de Octubre, Madrid, Spain; 98 Hospital Universitari i Politècnic La Fe, Valencia, Spain; 99 Hospital Clínico Universitario de Salamanca, Salamanca, Spain; 100 START Madrid-CIOCC Phase I Unit, University Hospital HM Sanchinarro, Madrid, Spain; 101 Hospital Universitario Fundacion Jimenez Diaz, Madrid, Spain; 102 Hospital Universitario Quirónsalud Madrid, Madrid, Spain; 103 Hospital Clínic de Barcelona, Barcelona, Spain; 104 Institut Catalá d’Oncologia Hospitalet, IDIBELL, Universitat de Barcelona, Barcelona, Spain; 105 Hospital de la Santa Creu i Sant Pau, IIB-Sant Pau and José Carreras Leukemia Research Institutes, Barcelona, Spain; 106 National Cheng Kung University Hospital, Tainan, Taiwan; 107 China Medical University Hospital, Taichung, Taiwan; 108 National Taiwan University Hospital, Taipei, Taiwan; 109 Chang Gung Memorial Hospital, Kaohsiung, Taiwan; 110 Royal Marsden Hospital, London, UK; 111 King’s College Hospital, London, UK; 112 Royal Cornwall Hospital, Truro, UK; 113 Derriford Hospital, Plymouth, UK; 114 University Hospital of Wales, Cardiff, UK; 115 Weill Cornell Medicine, New York, NY USA; 116 Stony Brook University Hospital, Stony Brook, New York, NY USA; 117 OU Health-Stephenson Cancer Center, Oklahoma City, OK USA; 118 Cleveland Clinic, Cleveland, OH USA; 119 John Theurer Cancer Center, Hackensack Meridian Health, Hackensack, NJ USA; 120 Rush University Medical Center, Chicago, IL USA; 121 Beth Israel Deaconess Medical Center, Boston, MA USA; 122 Mayo Clinic, Rochester, MN USA; 123 Atrium Health Wake Forest Baptist, Wake Forest University, Winston-Salem, NC USA; 124 Norton Cancer Institute, Louisville, KY USA; 125 Harold C. Simmons Comprehensive Cancer Center, University of Texas Southwestern Medical Center, Dallas, TX USA; 126 Tufts Medical Center, Boston, MA USA; 127 University of Iowa, Iowa City, IA USA

**Keywords:** Clinical trial design, Cancer, Antibody therapy, Drug discovery

Correction to: *Nature Cancer* 10.1038/s43018-025-00921-6, published online 17 March 2025.

This article was originally published under standard Springer Nature license (© The Author(s), under exclusive licence to Springer Nature America, Inc.). It is now available as an open-access paper under a Creative Commons Attribution 4.0 International license, © The Author(s). The error has been corrected in the HTML and PDF versions of the article.

