# Supplementary material for: Odronextamab monotherapy in patients with relapsed/refractory diffuse large B cell lymphoma: primary efficacy and safety analysis in phase 2 ELM-2 trial
Source: Nat Cancer. 2025 Mar 17;6(3):528–39. doi: 10.1038/s43018-025-00921-6 (PMC12003196; doi:10.1038/s43018-025-00921-6)
Supplement: Supplementary file 1 — Redacted Study Protocol and redacted Statistical Analysis Plan. [file 43018_2025_921_MOESM1_ESM.pdf]

nature cancer

Supplementary information https://doi.org/10.1038/s43018-025-00921-6

# Odronextamab monotherapy in patients with relapsed/refractory diffuse large B cell lymphoma: primary efficacy and safety analysis in phase 2 ELM-2 trial

In the format provided by the authors and unedited

**Table of Contents**

Study Protocol ........................................................................................................ 2

Statistical Analysis Plan ........................................................................................ 177

**EudraCT Number: 2017-002139-41**
IND Number: 120417

Regeneron Pharmaceuticals, Inc.

**Clinical Study Protocol**

# AN OPEN-LABEL STUDY TO ASSESS THE ANTI-TUMOR ACTIVITY AND SAFETY OF REGN1979, AN ANTI-CD20 X ANTI-CD3 BISPECIFIC ANTIBODY, IN PATIENTS WITH RELAPSED OR REFRACTORY B-CELL NON-HODGKIN LYMPHOMA

| | |
|---|---|
| **Compound:** | REGN1979 (odronextamab) |
| **Study Name:** | ELM-2 |
| **Clinical Phase:** | 2 |
| **Protocol Number:** | R1979-ONC-1625 |
| **Protocol Version:** | R1979-ONC-1625 Amendment 5 Global |
| **Amendment 5 Global Date of Issue:** | *See appended electronic signature page* |
| **Amendment 4 Global Date of Issue:** | 14 May 2021 |
| **Amendment 4 EU Date of Issue:** | 21 Dec 2020 |
| **Amendment 3 Global Date of Issue:** | 11 Nov 2020 |
| **Amendment 2 Global Date of Issue:** | 19 Jun 2020 |
| **Amendment 3 FR Date of Issue:** | 31 Mar 2020 |
| **Amendment 3 EU Date of Issue:** | 8 Jan 2020 |
| **Amendment 3 US Date of Issue:** | 20 Dec 2019 |
| **Amendment 2 FR Date of Issue:** | 13 Dec 2019 |
| **Amendment 2 US Date of Issue:** | 3 Dec 2019 |
| **Amendment 1 US Date of Issue:** | 11 Nov 2019 |
| **Amendment 1 Global Date of Issue:** | 4 Nov 2019 |
| **Amendment 2 EU Date of Issue:** | 16 Oct 2019 |
| **Amendment 1 EU Date of Issue:** | 15 Apr 2019 |
| **Original Date of Issue:** | 19 Dec 2018 |
| **Medical/Study Director:** | PPD<br>PPD<br>Regeneron Pharmaceuticals, Inc.<br>777 Old Saw Mill River Road<br>Tarrytown, NY 10591 |

**Confidential:** This submission contains confidential and proprietary commercial information and/or trade secrets which are subject to protection under laws and regulations worldwide including, but not limited to TRIPS Article 39 and regional, national or state laws whether by

legislation or in equity. For the purposes of compliance with all applicable freedom of information laws, and regulations, and their equivalents anywhere in the world including but not limited to the US Freedom of Information Act 5 U.S.C. §552, Regeneron expects that, prior to disclosure to third parties of any information and/or data related to, or contained within, this submission, it will be given every opportunity under applicable laws to protect its commercial interests and/or its trade secrets including the opportunity to object to such disclosure and/or provide redaction proposals.

# AMENDMENT HISTORY

Amendment 5 Global

The purpose of this amendment is to add monitoring for cytomegalovirus (CMV), add an exploratory endpoint measuring hospitalizations, update the inclusion criteria for the Other B-NHL cohort, clarify the total sample size for the study, and to add an interim administrative review of efficacy and safety. The principal changes to the protocol and the affected sections are highlighted in the table below.

| Description of Change | Brief Rationale | Section # and Name |
|---|---|---|
| **Change in Endpoints/Objectives** | | |
| Added an additional exploratory endpoint and objective measuring hospitalizations | To assess the burden of hospitalization for patients including but not limited to study drug administration and management of adverse events while on study | Section 2.3 Exploratory Objectives<br>Section 4.1.3 Exploratory Endpoints<br>Table 10 Schedule of Events |
| Updated the language within the immunogenicity endpoint | To clarify that immunogenicity will be determined by assessing the incidence and titer of anti-drug antibodies (ADA) and incidence of neutralizing antibodies (NAb) to odronextamab over time | Clinical Study Protocol Synopsis (Endpoints)<br>Section 4.1.2 Secondary Endpoints |
| **Administrative Efficacy Analysis** | | |
| Added an administrative review after at least 70 patients have been treated with the modified step-up regimen and have cleared a 4-week safety review | To inform further clinical development of the odronextamab program. | Clinical Study Protocol Synopsis (Statistical Plan)<br>Section 5.2 Planned Interim Analysis<br>Section 10.5 Interim Analysis |
| **Inclusion/Exclusion Criteria** | | |
| Update the inclusion criteria to allow patients with mixed B-NHL histology into the study | To allow patients with mixed histology B-NHL and patients with transformed DLBCL who already received treatments that are considered standard regimens for aggressive lymphoma in their prior lines given for lower grade neoplasms. These patients will be included in the 'Other B-NHL' cohort as they do not fulfill the criteria for the DLBCL cohort. | Clinical Study Protocol Synopsis (Target Population)<br>Section 1.5 Other B-cell non-Hodgkin Lymphoma<br>Section 6.2 Study Population<br>Section 6.2.1 Inclusion Criteria #3 |
| Removed women with tubal ligation from the list of women that are NOT required to undergo pregnancy testing at study entry. | Revised to be consistent with the IB and per MHRA feedback | Section 6.2.2 Exclusion Criteria, #19 |
| **Sample Size** | | |
| Clarified the total sample size | To include additional enrollment in China and Japan extension cohorts | Clinical Study Protocol Synopsis (Sample Size)<br>Section 6.1 Number of Patients Planned |



VV-RIM-00192456-1.0 Approved - 11 Apr 2022 GMT-5:00

| Description of Change | Brief Rationale | Section # and Name |
|---|---|---|
| **Infection Prophylaxis** | | |
| Added monitoring of CMV infection | To standardize CMV monitoring guidance during study | Section 6.2.2 Exclusion Criteria #12 (d) new<br>Section 7.3.4 B-Cell Depletion and Infection Prophylaxis<br>Section 7.3.4.1 Recommendations for Prophylaxis to Decrease the Rise of Infections<br>Table 10 Schedule of Events<br>Section 8.1.1 Footnotes for the Schedule of Events Table, #34<br>Section 8.2.3.7 Laboratory Testing<br>Section 9.4.3 Other Events that Require Accelerated Reported to Sponsor |
| Added discontinuation criteria for uncontrolled opportunistic infection | To permanently discontinue study drug due to uncontrolled opportunistic infection | Section 7.3.1.1 Reasons for Permanent Discontinuation of Study Drug |
| **COVID-19 Vaccination** | | |
| Guidance for COVID-19 vaccination was revised to include the booster dose. | Guidance has been updated regarding when patients may be vaccinated and receive a booster dose while enrolled in this study to support global vaccination activities during the COVID-19 pandemic. | Section 5.1.1.1 COVID-19 Vaccination During the Study<br>Section 7.6.2 Permitted Medications and Procedures |
| **Other Updates** | | |
| Updated footnote for PK sample collection | To clarify PK sample collection for Q4W | Section 8.1.3 Footnotes for the Schedule of Events Detailed Sample Collection Time Points for Cytokines, Pharmacokinetics, Lymphocyte Immunophenotying (Whole Blood), PBMC Immunophenotyping, C-Reactive Protein, and Ferritin, Footnote #6 |
| Added a note for patients switching from Q2W to Q4W dosing after 9 months of maintained CR, after .discussion and approval from the sponsor's Medical Monitor. | To clarify that patients receiving a lower dose than the protocol-specified Q2W dose may be able to switch to Q4W dosing. | Section 5.1 Study Description<br>Figure 3 Patient Study Flow Diagram footnotes<br>Section 5.1.3.1 Study Treatment<br>Section 8.1.1 Footnotes for the Schedule of Events Table, #30 |

| Description of Change | Brief Rationale | Section # and Name |
|---|---|---|
| Edited the observation periods for analysis of safety variables | To be consistent with the statistical analysis plan | Section 10.4.4.1 Adverse Events |
| Minor edits | For clarity | Throughout the protocol |



VV-RIM-00192456-1.0 Approved - 11 Apr 2022 GMT-5:00

Amendment 4 Global

The purpose of this amendment is to address Heath Authority (HA) feedback by implementing cytokine release syndrome (CRS) risk mitigation measures including a modified step-up dosing regimen, updated recommendations for tocilizumab and corticosteroid use in CRS management, premedications during step-up dosing, increased medical monitoring in the event of CRS and stopping rules based on the rate of grade ≥3 CRS events. In addition, enrollment will be opened only for FL grade 1-3a, DLBCL, and Other B-NHL cohorts with this amendment until an initial evaluation of safety is performed. The principal changes to the protocol and the affected sections are highlighted in the table below.

| Description of Change | Brief Rationale | Section Number and Name |
|---|---|---|
| **Changes to Study based on revised step-up dosing** | | |
| Decreased the total new dose at week 1 to 0.7 mg (split as 0.2 mg/ 0.5 mg to be given over 2 days, preferably consecutive).<br><br>Implemented an additional intermediate split dose of odronextamab (4 mg) at week 2 (intermediate dose 1) | To mitigate CRS risk and optimize the step-up regimen as per HA request. | Clinical Study Protocol Synopsis (Target Population, Study Design and Study Treatment)<br>Section 5.1.3.1 Study Treatment,<br>Figure 4 Odronextamab Treatment for FL Grade 1-3a Cohort and MZL Cohort<br>Figure 5 Odronextamab Treatment for DLBCL Cohort Arm 1, MCL after BTK Inhibitor Cohort, and Other B-NHL Cohort<br>Section 7.1 Investigational Treatment |
| Added dose rationale for decreasing the dose at week 1 and the addition of the split dose | To explain the rationale behind decreasing the initial dose at week 1 and the addition of split dose at week 2 | Section 3.2.2 Rationale for Dose Selection |
| Updated the duration of inpatient hospital admission based on the modified step-up regimen | Updated inpatient monitoring based on the modified step-up dosing regimen | Section 8.2.3.1 Safety Monitoring |
| Updated sample size | Updated sample size based on feedback from HA in order to provide sufficient data from patients dosed with the modified step-up dosing regimen | Clinical Study Protocol Synopsis (Population and Statistical Plan)<br>Figure 1 Study Schema |

| Description of Change | Brief Rationale | Section Number and Name |
|---|---|---|
| | | Section 6.1 Number of Patients Planned<br>Section 10.2.1 Follicular Lymphoma Grade 1-3a Cohort<br>Section 10.2.2 Diffuse Large B-Cell Lymphoma<br>Table 16 Sample size at Original and Modified Step-up Regimens in Each Cohort (new table)<br>Section 10.5 Interim Analysis<br>Table 18 Interim Futility Analysis |
| **Updates related to enrollment cohorts** | | |
| Paused enrollment for patients with MZL | The Sponsor would like to evaluate the effectiveness of additional CRS risk minimization measures in other sub-types prior to treating patients with MZL | Clinical Study Protocol Synopsis (Target Population),<br>Section 6.2 Study Population<br>Section 6.2.1 Inclusion Criteria #3 |
| Stopped enrollment for patients with Burkitt lymphoma and Burkitt-like lymphoma with 11q aberration | The Sponsor would like to ensure the revised modified step-up dosing regimen reduces the rates of CRS in other sub-types before extending to patients with Burkitt and Burkitt-like lymphoma, who may be more susceptible to CRS | Clinical Study Protocol Synopsis (Target Population),<br>Section 3.1 Hypothesis<br>Section 6.2 Study Population<br>Section 6.2.1 Inclusion Criteria #3 |
| **Updates related to patient safety (CRS and premedications)** | | |
| Added earlier intervention with tocilizumab and steroids for grade 1 and 2 CRS and updated the guidelines for administration of tocilizumab for the management of CRS following CARTOX working group and SITC recommendations and | To further mitigate the risk of severe CRS | Section 3.2.3 Rationale for Treatment Duration,<br>Section 7.3.3.1 Standard Intervention of Tozilizumab and Corticosteroids (new section),<br>Table 4 Management Guidance and Odronextamab Dosing in Setting of Cytokine |



VV-RIM-00192456-1.0 Approved - 11 Apr 2022 GMT-5:00

| Description of Change | Brief Rationale | Section Number and Name |
|---|---|---|
| added rationale for these recommendations | | Mediated Toxicities: Cytokine Release Syndrome (CRS) and Other Adverse Events<br>Table 5 Guidelines for Administration of Tocilizumab and Corticosteroids in the Setting of Cytokine Release Syndrome |
| Introduction of mandatory premedication with antihistamine and acetaminophen for the initial, intermediate, and first full dose of odronextamab. Further instruction on premedication with dexamethasone for all doses throughout the study are provided | To standardize premedications used during the step-up dosing as an additional measure to minimize the risk of CRS | Section 7.2 Premedications |
| Added earlier intervention with tocilizumab and enhanced corticosteroid pre- and post-medication if 2 patients experience grade ≥3 CRS | To mitigate further the risk of severe CRS and will be triggered only if 2 patients experience grade ≥3 CRS in any lymphoma subtype | Section 7.3.3.2.1 Premedication<br>Section 7.3.3.2 Earlier Intervention of Tocilizumab and Enhanced Corticosteroid Pre-and Post Medication ONLY Following Sponsor Notification (new section)<br>Table 6 Guidelines for EARLIER Administration of Tocilizumab in the Setting of Cytokine Release Syndrome. |
| Modified stopping rules for grade ≥3 CRS | To minimize the number of patients being exposed during optimization of step-up regimen for CRS events. | Figure 2 Schema for CRS Safety Assessments (new figure)<br>Section 10.4.4.2 Stopping Rules for Grade ≥3 CRS |

| Description of Change | Brief Rationale | Section Number and Name |
|---|---|---|
| **Other safety updates** | | |
| Updated dose modification for patients who experience tumor lysis syndrome | Updated the table based on the new dosing regimen | Table 9 Management Guidance and Study Drug Dosing in Setting of Tumor Lysis Syndrome |
| Added recommendations for gastrointestinal (GI) prophylaxis with proton pump inhibitors (PPI) and/or histamine 2 receptor antagonists (H2RA) during step-up dosing | To mitigate the risk of peptic ulcer disease due to corticosteroid use during step-up dosing | Section 7.2 Premedications |
| Added recommendations for anti-infective prophylaxis including IVIG supplementation, Pneumocystis jirovecii pneumonia (PJP) prophylaxis and antivirals | To mitigate the risk of infections due to additional immunosuppression from corticosteroid use and B-cell depletion | Section 7.3.4 B-Cell Depletion and Infection Prophylaxis<br>Section 7.3.4.1 Recommendations for Prophylaxis to Decrease the Risk of Infections (new section) |
| The permitted concomitant medication section has been updated to provide guidance about when patients may receive a COVID-19 vaccine prior to and while enrolled in this study. | Guidance has been added about when patients may be vaccinated prior to and while enrolled in this study to support global vaccination activities during the COVID-19 pandemic. | Section 5.1.1.1 COVID-19 Vaccination During the Study (new section)<br>Section 7.6.2 Permitted Medications and Procedures |
| **Statistical updates** | | |
| Clarified response assessment evaluation criteria for patients with MZL | Based on feedback from HA | Section 8.2.2.2 Radiographic Disease Assessment<br>Section 10.4.3.1 Primary Efficacy Analysis |
| Updated the timing of primary analysis until all patients have completed 52-week tumor assessment in the FL grade 1-3a/MZL cohorts and 36 week tumor assessment in the DLBCL/Other B-NHL/MCL cohorts or have withdrawn from the study. | Updated the timing of primary analysis to collect additional durability of response data based on HA feedback | Clinical Study Protocol Synopsis (Endpoint and Statistical Plan)<br>Section 4.1.1 Primary Endpoint<br>Section 4.1.2 Secondary Endpoints |

| Description of Change | Brief Rationale | Section Number and Name |
|---|---|---|
| | | Section 10.4.3.1 Primary Efficacy Analysis |
| Added an interim efficacy analysis when 80 patients with FL grade 1-3a have completed 52-week assessment and all patients in the DLBCL cohort have completed 36-week assessment or have withdrawn from the study earlier. | To inform further clinical development of odronextamab program | Clinical Study Protocol Synopsis (Statistical Plan)<br>Section 10.4.3.1 Primary Efficacy Analysis<br>Section 10.5 Interim Analysis |
| **Other updates related to schedule of events and timing of assessments** | | |
| Updated Schedule of Events table to clarify the assessments (weight, lymphatic exam as well as limited PE, B-symptom and ECOG performance status) done every 4 weeks. | Added more clarity to the schedule of events for the 4 weekly assessments | Table 10 Schedule of Events<br>Section 8.1.1 Footnotes for the Schedule of Events Table, footnotes 9, 10, 11 and 14 |
| Updated Schedule of Events table to require testing for cytokine profiling (serum) on Week 4 Day 2.<br>Added increased timepoints for sampling for cytokines, PK, PBMC, CRP, and ferritin. | To allow for continued safety monitoring of patients based on the modified step-up dosing schedule | Section 8.1 Schedule of Events<br>Section 8.1.1 Footnotes for the Schedule of Events Table, footnotes (25) and (26)<br>Table 11 Schedule of Events: Detailed Sample Collection Time Points for Cytokines, Pharmacokinetics, Lymphocyte Immunophenotyping, Ferritin, and C-Reactive Protein<br>Section 8.1.3 Footnotes for the Schedule of Events: Detailed Sample Collection Time Points for Cytokines , |

| Description of Change | Brief Rationale | Section Number and Name |
|---|---|---|
| | | Pharmacokinetics, Lymphocyte Immunophenotyping, Ferritin, and C-Reactive Protein Footnote 4<br>Section 8.2.8 Pharmacodynamic and Exploratory Biomarker Procedures |
| Added a new section with details on changes to the schedule of events if the dosing schedule is modified | Clarified the changes to the sample collection/study procedures if there is dose modification | Section 8.1.2 Changes to the Schedule of Events/Study Procedures in case of Dose Modifications |
| Removed TruCulture Whole blood sampling | Sufficient samples have been collected to perform analysis and validate the assay. | Section 8.1 Schedule of Events<br>Section 8.1.1 Footnotes for the Schedule of Events Table, footnotes (29; deleted footnote)<br>Table 11 Schedule of Events: Detailed Sample Collection Time Points for Cytokines, Pharmacokinetics, Lymphocyte Immunophenotyping, Ferritin, and C-Reactive Protein (Updated table title)<br>Section 8.1.3 Footnotes for the Schedule of Events: Detailed Sample Collection Time Points for Cytokines , Pharmacokinetics, Lymphocyte Immunophenotyping, Ferritin, and C-Reactive Protein Footnote 5 (Deleted footnote) |

| Description of Change | Brief Rationale | Section Number and Name |
|---|---|---|
| | | Section 8.2.8.1 Biomarker Assessments |
| Updated Schedule of Events table to increase timepoints sampling for lymphocyte and PBMC immunophenotyping, cytokines, CRP, and ferritin, and added footnote 34 to allow additional lab samples to be collected at any time | To allow for continued safety monitoring of patients and biomarker samples based on the modified step-up dosing regimen | Table 10 Schedule of Events<br>Section 8.1.1 Footnotes for the Schedule of Events Table, footnotes (25) and (26) and (33)<br>Table 11 Schedule of Events: Schedule of Events Detailed Sample Collection Time Points for Cytokines, Pharmacokinetics, Lymphocyte Immunophenotyping, PBMC Immunophenotyping. C-Reactive Protein, and Ferritin<br>Section 8.1.3 Footnotes for the Schedule of Events: Detailed Sample Collection Time Points for Cytokines , Pharmacokinetics, Lymphocyte Immunophenotyping, PBMC Immunophenotyping, TruCulture Whole Blood, C-Reactive Protein and Ferritin Footnote 2, 4 and 8 |
| **Administrative and other updates** | | |
| Updated Appendix 2 | To replace the table with verbatim excerpt from the publication on Lugano classification | Appendix 2 Malignant Lymphoma Response Definitions Per Lugano Criteria |

| Description of Change | Brief Rationale | Section Number and Name |
|---|---|---|
| Added the name of the study | Administrative changes | Protocol title page |
| Updated the number of sites | Administrative changes | Protocol Synopsis |
| Updated medical study director titles and added the name of an additional study director and updated the confidentiality statement | Administrative changes | Protocol title page |
| Editorial changes | Improved clarity | Throughout the protocol |

Amendment 4 EU

The purpose of this amendment is to pause enrollment for all patients with B-cell malignancies.

Amendment 3 Global

The purpose of this amendment is to address Heath Authority (HA) feedback, pause enrollment for patients with mantle cell lymphoma (MCL), and update the overdose language. The principal changes to the protocol and the affected sections are highlighted in the table below.

| Description of Change | Brief Rationale | Section Number and Name |
|---|---|---|
| Modified eligibility criteria to pause enrollment of patients with Mantle Cell Lymphoma (MCL) | Based on the disproportionate number of grade 3 or higher infusion-related reactions (IRR)/cytokine release syndrome (CRS) events and deaths observed in patients with MCL following either an initial or intermediate dose of odronextamab, current risk mitigation measures are inadequate to support continued investigation in this population. Enrollment of patients with MCL is halted until further risk mitigation measures are in place. | Clinical Study Protocol Synopsis (Target Population)<br>Section 5.1 Study Description<br>Section 6.2.1 Inclusion Criteria, #3<br>Figure 5 Odronextamab Treatment for DLBCL cohort Arm 1, MCL after BTK Inhibitor Cohort, and Other B-NHL Cohort |
| Clarified criteria for overdose and removed association with adverse events. | Updated per HA request to minimize risk of dosing errors. | Section 9.4.3 Other Events that Require Accelerated Reporting to Sponsor |
| Made editorial changes for clarity regarding split infusions | Updated for clarity. | Clinical Study Protocol Synopsis (Study Drug: Dose/Route/Schedule) |

| Description of Change | Brief Rationale | Section Number and Name |
|---|---|---|
| during initial and intermediate doses | | Section 5.1.3.1 Study Treatment<br>Figure 4 Odronextamab Treatment for FL grade 1-3a Cohort and MZL Cohort<br>Figure 5 Odronextamab Treatment for DLBCL cohort Arm 1, MCL after BTK Inhibitor Cohort, and Other B-NHL Cohort<br>Figure 6 Odronextamab Treatment for DLBCL Cohort Arm 2 |

Amendment 2 Global

The purpose of this amendment is to address Heath Authority (HA) feedback. The principal changes in this protocol amendment are as follows:

- In order to characterize better the treatment effect, the Sponsor standardized the dosing schedule for Q2W to Q4W transition. All patients with durable complete responses (CRs) as assessed by local investigator lasting for 9 months will transition from every 2 weeks (Q2W) to every 4 weeks (Q4W) dosing frequency at the assigned dose. Patients must have received the assigned nominal dose at the Q2W dosing schedule for at least 3 preceding doses before switching from Q2W to Q4W dosing in order to maintain adequate serum concentrations of odronextamab to minimize the risk of (CRS).
- Added safety pausing rule to pause the transition of patients from Q2W to Q4W dosing if 5 or more of the first 16 patients treated with at least two Q4W doses experience grade ≥2 CRS.
- Standardized the week 3 dose of 80 mg or 160 mg to be administered as a single infusion if a patient does not experience grade 3 CRS prior to the week 3 dose.
- Based on the cumulative safety, pharmacokinetic (PK) and efficacy data, in the diffuse large B-cell lymphoma (DLBCL) cohort, the Sponsor clarified that after the initial step, enrollment will only continue with the odronextamab 160 mg QW dosing arm followed by the 320 mg Q2W dosing arm until a total of 112 patients have received this dose level. Due to this change, the DLBCL cohort size changed from 162 to 146 patients. The data from the 2 arms will be used for descriptive analyses that support analysis of PK parameters and pharmacodynamic effects to advance the understanding of the exposure-response relationship, therefore, the interim analysis is removed for the DLBCL cohort.
- Updated the inclusion criterion for follicular lymphoma (FL) grade 1-3a cohort to require patients to have failed combination lenalidomide and rituximab treatment, where approved, or were deemed not appropriate to receive this treatment according to the investigator.

- To base eligibility for patients on the number of prior lines of systemic therapies, the inclusion criterion was updated to require failure of ≥2 rather than ≥1 prior line of systemic therapy for the Other B-cell non-Hodgkin lymphoma (B-NHL) cohort and the marginal zone lymphoma (MZL) cohort.
- Defined the specific types of aggressive lymphoma eligible for this study based on the World Health Organization (WHO) 2016 classification.
- In light of the COVID-19 pandemic, adapted language regarding site enrollment and initiation, as well as study procedures and monitoring.
- The Sponsor increased the stringency of the futility stopping rules by increasing the boundary for interim futility analysis with rationale for the Other B-NHL cohort, mantle cell lymphoma (MCL), and MZL cohorts.

The changes to the protocol and the affected sections are highlighted in the table below.

| Description of Change | Brief Rationale | Section Number and Name |
|---|---|---|
| Specified that if a patient has demonstrated a CR and has shown a durable response for at least 9 months after the initial determination of CR, the frequency of study drug administration will be reduced at the assigned dose from Q2W to Q4W intervals, based on local investigator evaluation. Patients must have received the assigned nominal dose at the Q2W dosing schedule for at least 3 preceding doses before switching from Q2W to Q4W dosing. | Standardized the dosing schedule by reducing the dosing frequency in patients demonstrating sustained CR to better characterize the treatment effect over time based on HA guidance. | Clinical Study Protocol Synopsis (Study Design, Study Duration)<br><br>Section 5.1 Study Description<br>Section 5.1.3.1 Study Treatment<br>Figure 4 Odronextamab Treatment for FL grade 1-3a Cohort and MZL Cohort<br>Figure 5 Odronextamab Treatment for DLBCL Cohort Arm 1, MCL after BTK Inhibitor Cohort, and Other B-NHL Cohort<br>Figure 6 Odronextamab Treatment for DLBCL Cohort Arm 2<br>Section 8.1.1 Footnotes for the Schedule of Events Table, footnote #30<br>Section 8.1.3 Footnotes for the Schedule of Events Detailed Sample Collection Time Points for Cytokines, Pharmacokinetics, Lymphocyte Immunophenotyping (Whole Blood), PBMC Immunophenotyping, TruCulture Whole Blood, C-Reactive Protein, and Ferritin, footnote #5 |

| Description of Change | Brief Rationale | Section Number and Name |
|---|---|---|
| Standardized the week 3 dose of 80 mg or 160 mg to be administered as a single infusion instead of a split infusion if a patient does not experience grade 3 CRS prior to the week 3 dose. | Updated based on HA guidance to standardize criteria for administering the week 3 dose as a single infusion versus split infusions over 2 days. | Clinical Study Protocol Synopsis (Treatment)<br>Section 3.2.2 Rationale for Dose Selection<br>Section 5.1.3.1 Study Treatment<br>Figure 4 Odronextamab Treatment for FL grade 1-3a Cohort and MZL Cohort<br>Figure 5 Odronextamab Treatment for DLBCL Cohort Arm 1, MCL after BTK Inhibitor Cohort, and Other B-NHL Cohort<br>Figure 6 Odronextamab Treatment for DLBCL Cohort Arm 2<br>Section 7.1 Investigational Treatment<br>Table 10 Schedule of Events, footnote #29 |
| In the DLBCL cohort, clarified that after the initial step, enrollment will only continue with the odronextamab 160 mg QW followed by 320 mg Q2W dosing arm until a total of 112 patients are reached for that dose level. The number of patients randomized in Arm 1 and Arm 2 decreased from 100 patients to 68 patients. Due to this change, the DLBCL cohort size changed from 162 patients to 146 patients. The number of patients to be enrolled in the study decreased from 497 patients to 481 patients.<br>Interim analysis is removed for the DLBCL cohort. | Based on the cumulative safety, PK and efficacy data, the dose for DLBCL cohort was selected to be 160 mg QW followed by 320 mg Q2W. The data from the 2 arms will be used for descriptive analyses that support analysis of PK parameters and pharmacodynamic effects to advance the understanding of the exposure-response relationship, therefore the interim analysis for this cohort has been removed. | Clinical Study Protocol Synopsis (Study Design, Population, Statistical Plan, Interim Analysis)<br>Section 5.1 Study Description<br>Section 5.2 Planned Interim Analysis<br>Section 6.1 Number of Patients Planned<br>Section 7.4 Method of Treatment Assignment<br>Section 10.2.2 Diffuse Large B-Cell Lymphoma<br>Figure 1 Study Schema<br>Section 10.4.3.1 Primary Efficacy Analysis |
| Changed re-treatment to resumption of treatment | Re-treatment is no longer applicable. | Section 5.1.4 Resumption of Treatment<br>Section 8.1.1 Footnotes for the Schedule of Events Table, #4 |
| Specified for inclusion in the FL grade 1-3a expansion cohort patients must have failed combination lenalidomide and rituximab | Updated based on HA guidance. | Section 6.2.1 Inclusion Criteria #3 |

| Description of Change | Brief Rationale | Section Number and Name |
|---|---|---|
| treatment where approved or where deemed not appropriate to receive this treatment according to the investigator. | | |
| Updated the inclusion criterion for the DLBCL cohort to include number of prior therapies directed only at the DLBCL and excluded therapies directed at the antecedent low-grade lymphoma. Additionally, patients with DLBCL transformation from prior CLL can only be enrolled in the absence of CLL component. | Updated based on HA guidance. | Section 6.2.1 Inclusion Criteria #3 |
| Updated the inclusion criterion for the other B-NHL cohort to require at least 2 prior lines of systemic therapy rather than at least 1. | Updated based on HA guidance to require at least 2 prior lines as this cohort includes lymphoma subtypes that will be considered aggressive in nature. | Clinical Study Protocol Synopsis (Primary Objective, Target Population)<br>Section 2.1 Primary Objective<br>Section 6.2.1 Inclusion Criteria #3<br>Section 6.2 Study Population |
| Updated the inclusion criterion for the MZL cohort to require at least 2 prior lines of systemic therapy rather than at least 1. | Updated based on HA guidance to require at least 2 prior lines given the availability of other approved therapies in this indication. | Clinical Study Protocol Synopsis (Primary Objective, Target Population)<br>Section 2.1 Primary Objective<br>Section 3.2.1 Rationale for Patient Population and Study Design<br>Section 6.2 Study Population<br>Section 6.2.1 Inclusion Criteria #3 |
| Removed duration of disease control from secondary objectives and endpoints | Lymphoma patients are considered to have refractory disease if there is no response within 6 months of the last dose, therefore, duration of disease control is not applicable after 6 months. | Clinical Study Protocol Synopsis, Secondary Objectives<br>Section 2.2 Secondary Objectives<br>Section 4.1.2 Secondary Endpoints<br>Section 10.2.5 Other B-Cell non-Hodgkin Lymphoma Cohort<br>Section 10.4.3.2 Secondary Efficacy Analysis |
| Moved pharmacokinetic parameters out of Section 4.2.2 and into Section 10.4.5.1 | To clarify the difference between PK variables and PK parameters. | Section 4.2.2 Pharmacokinetic Variables<br>Section 10.4.5.1 Analysis of Drug Concentration Data |

| Description of Change | Brief Rationale | Section Number and Name |
|---|---|---|
| Specified lymphoma subtypes that can be included for the DLBCL, MZL and Other B-NHL cohorts based on the World Health Organization (WHO) classification (Beham-Schmid, 2017) | Specified subtypes of MZL and other B-NHL according to HA feedback. | Section 6.2.1 Inclusion Criteria #3<br>Section 22 References |
| Modified the wording in the inclusion criterion 3, in the event that there are other approved lines of therapies in a specific indication. | Updated based on the HA feedback to consider the appropriateness of available therapies prior to enrollment. | Section 6.2.1 Inclusion Criteria #3 |
| Specified minimum hematologic parameters for patients with bone marrow involvement or splenic sequestration. | Updated based on HA guidance. | Section 6.2.1 Inclusion Criteria #6 |
| Corrected exclusion criterion 8 to specify the correct lymphoma subtype. | Corrected previous error where incorrect subtype was listed. | Section 6.2.2 Exclusion Criteria #8 |
| Corrected the error in the synopsis that stated that anti-CD20 antibody is a required prior line of systemic therapy in patients in the Other B-NHL cohort. | Corrected for consistency within the protocol. | Clinical Study Protocol Synopsis, Primary Objective<br>Section 2.1 Primary Objective |
| Added a statement that rescreening is allowed for one time only. | Updated to rescreen patients who failed screening at an earlier time point. | Section 5.1.2 Screening Period |
| Clarified that quality of life assessments will be administered electronically. | Updated based on HA guidance. | Clinical Study Protocol Synopsis, Procedures and Assessments<br>Section 5.1.3.3 Study Assessments<br>Section 8.2.7 Quality of Life Procedures |
| For the day 1 Week 1 dose, added a minimum time between the start of the first split infusion and the start of the second split infusion. | This allows adequate time to manifest if there is any potential TLS or CRS or other safety issues following the day 1 Week 1 dose. | Clinical Study Protocol Synopsis, Treatment<br>Section 7.1 Investigational Treatment |
| Added grade ≥3 neurotoxicity as a criteria for permanent discontinuation of study drug. | Updated based on HA guidance. | Section 7.3.1.1 Reasons for Permanent Discontinuation of Study Drug |
| Edited and added language to discuss with Sponsor potential provisions for monitoring or dosing upon restarting study | To enhance safety of restarting study drugs after prolonged interruption. | Section 7.3 Dose Modification, Study Drug Discontinuation, and Management of Specific Toxicities |

| Description of Change | Brief Rationale | Section Number and Name |
|---|---|---|
| drugs in the event of prolonged interruption. | | Section 7.3.1.2 Reasons for Restarting Study Drug Administration Following Temporary Treatment Interruption (new) |
| Added language regarding site enrollment and initiation in light of COVID-19. | To address a requirement under the guidance that has come out in the EU to incorporate some language in the benefit/risk to address considerations related to conduct of a study during the pandemic. | Section 5.1.1 Study Conduct in Response to COVID-19 (new) |
| Adaptations in study procedures and monitoring during the on-going COVID-19 pandemic. | To address guidance from the US and EU regarding the conduct of clinical trials during the COVID-19 pandemic. | Section 8.1 Schedule of Events |
| Updated the concomitant medications section to include guidance for patients who are receiving concomitant cytochrome P450 (CYP450) substrates. | To address the potential elevation of cytokines in patients receiving T-cell engaging therapeutics which may temporally suppress cytochrome P450 (CYP450) enzymes. | Section 7.6 Concomitant Medications and Procedures |
| Added instructions that additional ADA testing may be conducted on previously collected samples during first 12 weeks of treatment.<br><br>Clarified sampling timepoints for whole blood (tumor DNA), plasma ctDNA samples, PBMC immunophenotyping, and lymphocyte immunophenotyping (whole blood).<br><br>Minimal residual disease (MRD) samples will be collected for patients that achieve CR at each time point that Pet-CT scans are performed. | Updated based on HA feedback and change in study design. | Section 8.1.1 Footnotes for the Schedule of Events Table, footnote #23<br>Section 8.1.3 Footnotes for the Schedule of Events Detailed Sample Collection Time Points for Cytokines, Pharmacokinetics, Lymphocyte Immunophenotyping (Whole Blood), PBMC Immunophenotyping, TruCulture Whole Blood, C-Reactive Protein, and Ferritin, footnote #7<br>Section 8.2.8.1 Biomarker Assessments |
| Updated schedule of events tables as follows:<br>• Vital signs measured at week 12<br>• Removed collections for C-reactive protein and ferritin at week 4, days 2 and 3, as well | For consistency.<br><br>Data so far have shown little value for collecting these laboratory tests at later timepoints. | Table 10 Schedule of Events<br>Section 8.1.1 Footnotes for the Schedule of Events Table, #7, #25, #26, #27, #29<br>Table 11 Schedule of Events Detailed Sample Collection Time Points for Cytokines, Pharmacokinetics, |

| Description of Change | Brief Rationale | Section Number and Name |
|---|---|---|
| as weeks 16, 18, 20, 36 safety follow-up visit 1. Removed collections for cytokine profiling at weeks 18<br>• Added a ± 30 minute window for each TruCulture collection<br>• Added annual MRD assessments<br>• CT/MRI are collected Q12W during extended follow-up | To further clarify the window of timing when sample collection is acceptable.<br>For consistency with other protocols in the odronextamab program.<br>For clarity. | Lymphocyte Immunophenotyping (Whole Blood), PBMC Immunophenotyping, TruCulture Whole Blood, C-Reactive Protein, and Ferritin<br>Section 8.1.3 Footnotes for the Schedule of Events Detailed Sample Collection Time Points for Cytokines, Pharmacokinetics, Lymphocyte Immunophenotyping (Whole Blood), PBMC Immunophenotyping, TruCulture Whole Blood, C-Reactive Protein, and Ferritin, Footnotes #4 and #5 |
| Added grade 3 or greater IRR to list of adverse events of special interest (AESI) | Updated to collect reports of grade 3 or greater IRR. | Section 9.4.3 Other Events that Require Accelerated Reporting to Sponsor |
| Increased the boundary for interim futility analysis with rationale for the Other B-NHL cohort, MCL, and MZL cohorts. | Updated based on HA guidance to increase the stringency of the futility stopping rules. | Clinical Study Protocol Synopsis (Statistical Plan)<br>Section 10.2.5 Other B-Cell non-Hodgkin Lymphoma Cohort<br>Table 15 The 2-sided 95% Exact Confidence Intervals for Observed ORR Given a Sample Size of 67 Patients<br>Section 10.5 Interim Analysis<br>Table 18 Interim Futility Analysis |
| Added safety pausing rule to pause the transition of patients from Q2W to Q4W dosing if 5 or more of the first 16 patients treated with at least two Q4W doses experience grade ≥2 CRS. | Updated based on HA guidance to delay transition to lower dosing frequency if deemed unsafe. | Section 10.4.4.2 Safety Pausing Rule after Transition from Q2W to Q4W Dosing (new)<br><br>Table 17 Percent of Patients with Grade ≥2 CRS with 1-Sided 80% Confidence Interval |
| Changed reference to the Pharmacovigilance and Risk Management department/lead to the Global Patient Safety department/lead. | Departmental name and organizational change. | Section 9.6 Safety Monitoring. |
| Changed mentions of Amendment 1 Global to | Corrected for accuracy. | Section 3.2.1 Rationale for Patient Population and Study Design |

| Description of Change | Brief Rationale | Section Number and Name |
|---|---|---|
| reference this amendment instead. | | Section 3.2.3 Rationale for Treatment Duration<br>Section 5.1.4 Re-Treatment<br>Table 10 Schedule of Events, footnote #4 |
| Added a new section regarding clinical study data transparency. | Updated to be consistent across the sponsor's protocols. | Section 14.5 Clinical Study Data Transparency |
| Replaced REGN1979 with odronextamab | Implementation of the international nonproprietary name. | Throughout |
| Edits made for clarity and grammatical errors. | Updated for clarity. | Throughout |

**Amendment 3 US**

The main changes to the protocol are:

- To include 4 additional study cohorts comprising patients with relapsed or refractory:
  - Diffuse large B-cell lymphoma (DLBCL),
  - Mantle cell lymphoma (MCL) following failure of or intolerance to a Bruton's tyrosine kinase (BTK) inhibitor,
  - Marginal zone lymphoma (MZL), or
  - Other B-cell non-Hodgkin lymphomas (NHLs) that are known to have the CD20 protein expression.
- In the follicular lymphoma (FL) grade 1-3a and MZL cohorts, the nominal dose of odronextamab will be 80 mg during weekly (QW) dosing and subsequently 160 mg during Q2W dosing.
- In the MCL cohort and the cohort comprising patients with other B-NHLs, the nominal QW full dose of odronextamab will be 160 mg during QW dosing and subsequently 320 mg during Q2W dosing.
- In the DLBCL cohort, patients will be randomized to 2 treatment arms (1:1) in the initial step of this cohort to select the dose regimen. In Arm 1, the QW full dose of odronextamab will be 160 mg during QW dosing and subsequently 320 mg during Q2W dosing. In Arm 2, the QW full dose of odronextamab will be 320 mg during QW dosing and subsequently 320 mg during Q2W dosing. After an initial comparison of dose regimens to evaluate the respective safety, efficacy, and PK data, one study Arm in this cohort may be closed, and the other Arm with the selected dose regimen will continue enrollment to a total of 112 patients for that Arm.
- In considering the clonal cellular heterogeneity of lymphomas and the potential need to continue treatment in order to achieve durable control of disease, the sponsor is revising the treatment duration in this study to continue until the time of disease progression or any other protocol-defined reason for treatment discontinuation. In addition, if a patient has demonstrated a CR and has shown a durable response for at least 9 months after the initial determination of CR, then the investigator may choose to decrease the frequency of study infusions at the assigned dose from Q2W intervals to Q4W intervals.

The following table summarizes the revisions in this protocol amendment:

| **Change and Rationale for Change** | **Section Changed** |
|---|---|
| Updated the description of the study patient population to include additional B-NHL subtypes. | Title Page<br>Clinical Study Protocol Synopsis<br>Section 1 Introduction<br>Section 1.1 Follicular Lymphoma Grade 1-3a<br>Section 1.2 Diffuse Large B-Cell Lymphoma<br>Section 1.3 Mantle Cell Lymphoma<br>Section 1.4 Marginal Zone Lymphoma<br>Section 1.5 Other B-NHL Subtypes<br>Section 2 Study Objectives<br>Section 3 Hypothesis and Rationale<br>Section 4 Study Endpoints and Variables<br>Section 5.1 Study Description<br>Section 5.2 Planned Interim Analysis<br>Section 6.1 Number of Patients Planned<br>Section 6.2 Study Population<br>Section 10 Statistical Plan |
| In the FL grade 1-3a and MZL cohorts, odronextamab will be administered at a nominal dose of 80 mg during QW dosing followed by 160 mg during Q2W dosing. In the MCL after BTK inhibitor cohort and DLBCL cohort Arm 1, odronextamab will be administered at a nominal dose of 160 mg during QW dosing and subsequently 320 mg during Q2W dosing. In the DLBCL cohort Arm 2, patients will receive 320 mg as a nominal dose during QW dosing and subsequently 320 mg during Q2W dosing. The dose regimen for each disease-specific cohort is based on recent clinical observations of safety and efficacy in the first-in-human study R1979-HM-1333 study. | Section 1 Introduction<br>Section 3.2.2 Rationale for Dose Selection<br>Section 5.1.2.1 Study Treatment<br>Section 7.1 Investigational Treatment |
| The treatment duration in this study is revised to continue until the time of disease progression or other protocol-defined reason for treatment discontinuation. In addition, if a patient has demonstrated a CR and has shown a durable response for at least 9 months after the initial determination of CR, then the investigator may choose to decrease the odronextamab dosing frequency thereafter at the assigned dose from Q2W to Q4W intervals. | Clinical Study Protocol Synopsis<br>Section 3.2.3 Rationale for Treatment Duration<br>Section 5 Study Design<br>Section 5.1.5 End of Study Definition<br>Section 8.1 Schedule of Events |
| Added lymph node tissue biomarker analysis at baseline and on-treatment as an exploratory objective and endpoint (eg, CD20 antigen expression, T cell density and phenotype). | Section 2.3 Exploratory Objectives<br>Section 4.1.3 Exploratory Endpoints |



VV-RIM-00192456-1.0 Approved - 11 Apr 2022 GMT-5:00

| **Change and Rationale for Change** | **Section Changed** |
|---|---|
| The safety and tolerability of 320 mg weekly odronextamab has been demonstrated in study R1979-HM-1333, which includes a first nominal dose at week 3 of 160 mg odronextamab on each of 2 days. Therefore, for cohorts in this study in which the weekly nominal dose does not exceed 160 mg odronextamab (all cohorts except Arm 2 of the DLBCL cohort), the first nominal dose at week 3 may be administered either as a single infusion (previously mandated as a split infusion over 2 days) or as a split infusion at the investigator's discretion.<br>In Arm 2 of the DLBCL cohort, in which the nominal dose for QW dosing is 320 mg, patients will receive the first nominal dose administered at week 3 split as 2 separate infusions of 160 mg over 2 days. | Clinical Study Protocol Synopsis<br>Section 5.1.2.1 Study Treatment<br>Section 3.2.2 Rationale for Dose Selection<br>Section 8.1.1 Footnotes for the Schedule of Events |
| Removed the re-treatment option for patients who are newly enrolled or continue on study treatment at the time of Amendment 1 Global, because as of this amendment patients are treated until the time of disease progression or other protocol-defined reason for study treatment discontinuation. For patients who are off treatment at the time Amendment 1 Global becomes active, re-treatment may be considered. | Section 5.1.4 Re-Treatment<br>Section 8.1.1 Footnotes for the Schedule of Events |
| Specified that the treatment assignment for patients enrolled into the 2 DLBCL arms will be in a randomized manner to investigate 2 different dose regimens. | Section 7.4 Method of Treatment Assignment |
| The FACT-Lym questionnaire is added to the EORTC QLQ-C30 and EQ-5D-3L questionnaires to assess health-related quality-of-life (HRQL) changes for study patients. | Clinical Study Protocol Synopsis<br>Section 2.2 Secondary objectives<br>Section 4.1.2 Secondary endpoints<br>Section 8.1 Schedule of Events<br>Section 8.2.7 Quality of Life Procedures<br>Section 10.4.7 Analysis of Quality of Life Data |
| To enhance the operational feasibility of this study, an additional 7-day interval during the screening period will be allowed for baseline radiographic imaging and central histopathology confirmation. | Section 5.1.1 Screening Period<br>Section 8.1 Schedule of Events |
| Specified that an Independent Data Monitoring Committee (IDMC) is established to review clinical safety data for this study. | Section 6.3.1 Independent Data Monitoring Committee |

| Change and Rationale for Change | Section Changed |
|---|---|
| Revisions to safety monitoring introduced following Health Authority guidance:<br>• Clarified footnote Table 7: Diagnosis of Laboratory and Clinical Tumor Lysis Syndrome to consider other differential diagnosis for acute kidney injury<br>• Added that no other anti-lymphoma therapies are permitted while patients are receiving study treatment.<br>• Added guidance regarding hematopoietic support comprising G-CSF or platelet transfusion therapy during screening.<br>• Clarification regarding testing during screening for HIV, HBV, and HCV.<br>• The safety follow-up period has been revised to record all adverse events until 90 days following the last dose of odronextamab, or until the start of non-protocol anti-lymphoma therapy, whichever is first. | Table 7 Diagnosis of Laboratory and Clinical Tumor Lysis Syndrome*<br>Section 7.6.1 Prohibited Medications<br>Section 7.6.2 Permitted Medications and Procedures<br>Section 8.2.3.7 Laboratory Testing<br>Section 8.1.3 Early Termination Visit<br>Section 8.2.3.1 Safety Monitoring<br>Section 9.4.1 Adverse Events<br>Section 9.4.2 Serious Adverse Events<br>Section 9.4.6 Follow-up |
| Revisions to patient eligibility included:<br>• Added inclusion criteria specifications for disease-specific cohorts.<br>• Specified that the investigator must discuss with the sponsor the eligibility of patients with cell counts below thresholds listed in the eligibility criteria to enroll in the study if the low values may be explained by lymphoma bone marrow infiltration or splenic sequestration of platelets due to lymphoma.<br>Revisions to patient eligibility based on health authority guidance:<br>• Added guidance regarding sperm donation has been included.<br>• Added prior treatment with anti-CD20 bispecific antibodies as an exclusion criterion | Section 6.2.1 Inclusion Criteria #2, #3<br>Section 6.2.1 Inclusion Criteria #6<br>Section 6.2.1 Inclusion Criteria #7<br>Section 6.2.2 Exclusion Criteria #19<br>Section 6.2.2 Exclusion Criteria #20 |
| Clarified blood samples are to be collected for biomarker assessments in the DLBCL cohort only.<br>Clarified that unused biomarkers samples, as well as PK and ADA samples, will be stored for up to 15 years following completion of the study. | Section 8.1.1 Footnotes for the Schedule of Events<br>Section 8.2.8.1 Biomarker Assessments<br>Section 8.2.8.2 Whole Blood Immune Monitoring Sample<br>Section 8.2.8.3 PBMC Immunophenotyping Sample |

| Change and Rationale for Change | Section Changed |
|---|---|
| Added new section describing PBMC immunophenotyping samples. | Section 8.2.8.3 PBMC immunophenotyping sample |
| Revised PK and immunogenicity variables and analysis set definitions to be consistent across the sponsor's protocols. | Section 4.2.2 Pharmacokinetic Variables<br>Section 4.2.3 Immunogenicity Variables<br>Section 10.3.3 Pharmacokinetic Analysis Set<br>Section 10.3.4 Immunogenicity Analysis Set<br>Section 10.4.6 Analysis of Immunogenicity Data |
| The timeframe for recording concomitant medications and procedures has been extended to 90 days following the last administration of study drug, or until the start of start of non-protocol anti-lymphoma therapy, whichever is first. This change takes into consideration the interval comprising 5 half-lives of study drug (approximately 3 months) and is aligned with the safety follow-up period in this protocol amendment. | Section 7.6 Concomitant Medications and Procedures |
| Updated NCI-CTCAE grading for tumor flare. | Section 9.5.1 Evaluation of Severity |
| Added Patients may be followed remotely by the study site (eg, by telephone) to obtain survival information | Section 8.2.6 Survival Data Collection |
| Added a clarification to the hospitalization seriousness criterion.<br>Updated and renamed Section 9.7 to be consistent across the sponsor's protocols. | Section 9.3.2 Serious Adverse Events<br>Section 9.7 Investigator Alert Notification |
| Revised the sample size for the FL grade 1-3a cohort from 100 to 112 patients.<br><br>Added a table to summarize the interim futility analysis for MCL, MZL, and other B-NHL specific disease cohorts. | Clinical Study Protocol Synopsis<br>Section 10.2.1 Follicular Lymphoma Grade 1-3a Cohort<br>Table 15 Interim Futility Analysis |
| Five observation periods have been specified for the analysis of safety variables. In addition, the definition of the on-treatment, core, and re-treatment periods has been revised to comprise the time of the first dose of study drug until 90 days after last dose, or until the start of non-protocol anti-lymphoma therapy, whichever is first. | Section 10.4.4.1 Adverse Events |
| Due to the change in treatment duration in this study, updated the assessment time points in the Schedule of Events.<br>Added definition of the follow-up period to include safety follow-up, extended follow-up, and survival monitoring.<br>Clarification has been made that the additional 7-day interval for the screening period will also | Table 9 Schedule of Events<br><br>Section 8.1.1 Footnotes for the Schedule of Events |

| Change and Rationale for Change | Section Changed |
|---|---|
| allow for imaging and central histopathology confirmation.<br>Clarification has been made for EORTC-QLQ-C30 and EQ5D-3L time points and the addition of the FACT-Lym questionnaire.<br>Updated footnote to specify lymphocyte immunophenotyping (whole blood), TruCulture, and PBMC immunophenotyping sample collection time points.<br>Updated footnotes to clarify when ADA samples will be collected.<br>A bone marrow aspirate and/or bone marrow biopsy will be performed at week 12 instead of week 10.<br>Clarified that computed tomography or magnetic resonance imaging is required at screening and during on-study tumor assessment time points.<br>Due to the change in treatment duration in this study, updated detailed sample collection time points for cytokines, pharmacokinetics, lymphocyte immunophenotyping (whole blood), PBMC immunophenotyping, TruCulture whole blood, c-reactive protein, and ferritin. | Section 5.1.2.3 Study Assessments<br>Section 8.2.2.1 Computed Tomography Imaging/Magnetic Resonance Imaging and $^{18}$F-Fluorodeoxyglucose-Positron Emission Tomography<br>Table 10 Schedule of Events Detailed Sample Collection Time Points for Cytokines, Pharmacokinetics, Whole Blood Immune Monitoring, PBMC Immunophenotyping, C-Reactive Protein, and Ferritin<br>Section 8.1.2 Footnotes schedule of events detailed sample collection time points for cytokines, pharmacokinetics, lymphocyte immunophenotyping (whole blood), PBMC immunophenotyping, TruCulture whole blood, c-reactive protein, and ferritin |
| Specified the laboratory tests that will be analyzed by local and/or central laboratory.<br>Added instructions that if there are significant laboratory abnormalities prior to study drug administration, then that study drug administration should be temporarily withheld until resolution of those abnormalities. | Section 8.2.3.7 Laboratory Testing |
| Added lymphoma response to immunomodulatory therapy (LYRIC) criteria as a new appendix | Appendix 3 |
| Minor, editorial changes for consistency, clarity and correction of typographical errors. | Throughout the protocol |



VV-RIM-00192456-1.0 Approved - 11 Apr 2022 GMT-5:00

**Amendment 2 US**

The purpose of this amendment is to revise the study protocol following Health Authority (HA) review.

The following table outlines the changes made to the protocol:

| Change and Rationale for Change | Sections Changed |
|---|---|
| Removal of the term 'clinically significant' when describing tumor lysis syndrome (TLS) management. | Section 7.3.5.1 Guidelines for Tumor Lysis Syndrome Prophylaxis<br>Section 7.3.5.2 Recommended Monitoring for Tumor Lysis Syndrome<br>Section 7.3.5.3 Dose Modification of odronextamab for Patients who Experience Tumor Lysis Syndrome |

**Amendment 1 US**

The primary purpose of this amendment is to revise the study protocol:

- Following Health Authority (HA) review
- To include additional risk minimization measures for tumor lysis syndrome (TLS)
- To provide revised cytokine release syndrome (CRS) toxicity grading.

The following table outlines the changes made to the protocol:

| Change and Rationale for Change | Sections Changed |
|---|---|
| The follow-up period after treatment discontinuation was updated by adding a safety follow-up visit at 8 weeks following the last dose to capture all adverse events until 50 days after the last dose of odronextamab (ie, 5 half-lives) or until the start of new treatment for lymphoma, whichever is first. | Figure 1 Patient Study Flow Diagram<br>Section 5.1.3 Follow-up Period<br>Table 8 Schedule of Events. Footnotes 1, 3, 10, 11, 13, 14, 15, 16, 23<br>Table 9 Schedule of Events Detailed Sample Collection Time Points for Cytokines, Pharmacokinetics, Lymphocyte Immunophenotyping, Ferritin, and C-Reactive Protein<br>Section 7.3.1 Study Treatment Discontinuation<br>Section 7.6 Concomitant Medications and Procedures<br>Section 8.1.3 Early Termination Visit<br>Section 9.4.1 Adverse Events<br>Section 9.4.2 Serious Adverse Events<br>Section 9.4.6 Follow-up<br>Section 10.4.4.1 Adverse events |
| A clarification to allow patients for retreatment who had a response during the initial treatment period, but did not complete the full treatment period, in the event of recurrence was included. | Section 5.1.4 Re-treatment |
| Increased the number of sites to approximately 130 sites. | Clinical Study Protocol Synopsis<br>Section 6.1 Number of Patients Planned |
| Modified exclusion criterion 6 to exclude from study eligibility patients who have a history of uncontrolled seizure disorders, defined as any seizure within 12 months prior to study enrollment. | Section 6.2.2 Exclusion Criteria – criterion 6 |
| Patients with Gilbert syndrome can have coexisting liver disease, and in such circumstances study enrollment might not be appropriate; therefore, the exclusion criteria were updated to exclude patients with Gilbert syndrome if the total bilirubin value is >4 x upper limit of normal (ULN) for the local general population. | Section 6.2.1 Inclusion Criteria #7 |

| Change and Rationale for Change | Sections Changed |
|---|---|
| Early phases of pregnancy cannot be visualized by ultrasound, and therefore women of childbearing potential (WOCBP) with a positive serum pregnancy test will be excluded. | Section 6.2.2 Exclusion Criteria #16 |
| Exclusion criterion #19 was revised to include the definitions for women of childbearing potential and postmenopausal state as per Clinical Trial Facilitation Group (CTFG) guidance.<br>In addition, clarification has been included that having a vasectomized partner constitutes a highly effective birth control method provided that this person is the sole sexual partner of the WOCBP trial participant and that the vasectomized partner has received a medical confirmation of vasectomy success. | Section 6.2.2 Exclusion Criteria #19 |
| Added permanent study drug discontinuation in the setting of clear evidence of progressive disease. Clarified that patients with suspected pseudo-progression of disease and who are adequately tolerating odronextamab may continue study treatment, as specified in Section 5.1.2.4 . This addition is consistent with the study design. | Section 7.3.1.1 Reasons for Permanent Discontinuation of Study Drug |
| Included statement that emergency equipment and medication for the treatment of CRS/IRR events must be available. | Section 7.3.2.1 Acute Allergic/Hypersensitivity Infusion Reactions |
| The protocol has been updated to reference established standards for the infection prophylaxis in patients with cancer. | Section 7.3.4 B-Cell Depletion<br>Section 22 References |
| Safety monitoring has been modified to include mandatory hospitalization for each infusion up to and including the first QW nominal dose administered as a single infusion or until the week 4 infusion, whichever is later. In-patient observation should be for approximately 48 hours from the start of study treatment administration and also for approximately 24 hours beyond the end of the second split infusion or the end of the single infusion. | Clinical Study Protocol Synopsis<br>Section 5.1.2.1 Study Treatment<br>Section 8.2.3.1 Safety Monitoring<br>Table 8 Schedule of Events. Footnote 6. |

| Change and Rationale for Change | Sections Changed |
|---|---|
| Although most patients with FL are expected to have undergone testing for HIV, HBV and HCV as part of their previous oncologic treatment. some patients might not have had such testing performed previously. Therefore, such testing will be performed in patients who have unknown viral serologic status prior to screening or who have not had testing within 12 months prior to study drug administration. | Table 8 Schedule of Events. Footnote 22 |
| Revised cytokine release syndrome toxicity grading based on the American Society for Transplantation and Cellular Therapy (ASTCT) consensus grading (Lee 2019). | Section 7.3.3 Grading and Management of Infusion-related Reaction and Cytokine Release Syndrome<br>Table 1: Cytokine Release Syndrome Toxicity Grading<br>Table 2 Management Guidance and odronextamab Dosing in Setting of Cytokine-Mediated Toxicities: Infusion-related reactions<br>Table 3: Management Guidance and odronextamab Dosing in Setting of Cytokine Mediated Toxicities – CRS and Other Adverse Events<br>Section 22 References |
| Additional risk minimization measures for tumor lysis syndrome (TLS) have been added to the protocol, including recommendations for TLS prophylaxis, guidelines for risk stratification, monitoring and management, including dose modification for odronextamab based on new safety data. | Clinical Study Protocol Synopsis: Treatment<br>Section 7.3 Dose Modification, Study Drug Discontinuation, and Management of Specific Toxicities<br>Section 7.3.1.1 Reasons for Permanent Discontinuation of Study Drug<br>Section 7.3.2 Safety Monitoring and Management of Specific Toxicities<br>Section 7.3.5 Tumor Lysis Syndrome (all sub sections)<br>Table 5 Tumor Lysis Syndrome Risk Classification and Monitoring<br>Table 6 Diagnosis of Laboratory and Clinical Tumor Lysis Syndrome<br>**Table 7** Management Guidance and Study Drug Dosing in Setting of Tumor Lysis Syndrome<br>Table 8 Schedule of Events (blood chemistry sample timepoint added)<br>Section 8.1.1 Footnotes for the Schedule of Events<br>Section 22 References |
| Clarification regarding when patients with infections and HBV reactivation may commence treatment has been included in the protocol. | Section 7.3.4 B-Cell Depletion and Infection |

| Change and Rationale for Change | Sections Changed |
|---|---|
| Multi-focal leukoencephalopathy and clear evidence of disease progression were each added as a criterion for permanent treatment discontinuation. | Section 5.1.2.4 Treatment Continuation with Suspected Initial Pseudo-Progression<br>Section 7.3.1.1 Reasons for Permanent Discontinuation of Study Drug |
| Updated timeframe for when live vaccine administration is prohibited taking into account the drug exposure period within 5 half-lives of odronextamab as well as the recovery of B-lymphocyte number and function to prevent disseminated viral infection. | Section 7.6.1 Prohibited Medications |
| Specified NCI-CTCAE v5.0 as severity grading criteria for IRR. Table 2 has been edited to provide management guidance for severity grades 2, 3, 4. | Table 2 Management Guidance and odronextamab Dosing in Setting of Cytokine-Mediated Toxicities-Infusion-related Reactions |
| Reference to a study reference manual in the laboratory and efficacy procedures sections were revised to specify the laboratory manual. The laboratory manual includes instructions on sample handling only.<br>Listed laboratory tests that will be analyzed by local laboratory. Clarified INR should be PT (INR). | Section 8.2.2.3 Bone Marrow Aspirate and Bone Marrow Biopsy<br>Section 8.2.2.4 Lymph Node and/or Tumor Biopsy<br>Section 8.2.3.7 Laboratory Testing<br>Section 8.2.8.1 Biomarker Assessments |
| Clarified laboratory tests for PT (INR) in table and footnotes for Schedule of Events.<br>Clarified EORTC-QLQ-C30 and EQ5D-3L timepoints.<br>Revised blood samples collection timepoints for biomarker assessments.<br>Added Lymphatic exam in the footnote 11 to be consistent with Schedule of Events table.<br>Updated footnote for Schedule of Events to include a window for Fluorodeoxyglucose positron emission tomography and clarified when FDG-PET will be performed.<br>Updated footnote for Schedule of Events to specify whole blood (tumor DNA) as specified in the table. | Table 8 Schedule of Events<br>Table 9 Schedule of Events Detailed Sample Collection Time Points for Cytokines, Pharmacokinetics, Lymphocyte Immunophenotyping, Ferritin, and C-Reactive Protein |
| Reference to a study reference manual in protocol Section 9.4 'Recording and Reporting Adverse Events' has been deleted, because it was previously included in error. The protocol includes sufficient instruction regarding the recording and reporting of adverse events. | Section 9.4.2 Serious Adverse Events<br>Section 9.4.3 Other Events that Require Accelerated Reporting to Sponsor<br>Section 9.4.4 Reporting Adverse Events Leading to Withdrawal from the Study |

# CLINICAL STUDY PROTOCOL SYNOPSIS

| | |
|---|---|
| **Title** | An Open-Label Study to Assess the Anti-Tumor Activity and Safety of REGN1979, an anti-CD20 x anti-CD3 Bispecific Antibody, in Patients with Relapsed or Refractory B-cell non-Hodgkin Lymphoma |
| **Site Locations** | Multiple sites across the United States, Canada, Europe, and Asia-Pacific regions |
| **Objectives** | |
| **Primary Objective:** | The primary objective of this study is to assess the anti-tumor activity of single agent odronextamab as measured by the objective response rate (ORR) according to the Lugano Classification of response in malignant lymphoma (Cheson, 2014) non-Hodgkin lymphoma (B-NHL) subgroups:<br>• In patients with follicular lymphoma (FL) grade 1-3a that has relapsed after or is refractory to at least 2 prior lines of systemic therapy, including an anti-CD20 antibody and an alkylating agent.<br>• In patients with diffuse large B-cell lymphoma (DLBCL) that has relapsed after or is refractory to at least 2 prior lines of systemic therapy, including an anti-CD20 antibody and an alkylating agent.<br>• In patients with mantle cell lymphoma (MCL) that has relapsed after or is refractory to a Bruton's tyrosine kinase (BTK) inhibitor. This cohort will also include patients who have relapsed or have disease refractory to prior systemic therapy, or patients who have demonstrated intolerance to BTK inhibitor therapy and who have progressed after other systemic therapy.<br>• In patients with marginal zone lymphoma (MZL) that has relapsed after or is refractory to at least 2 prior lines of systemic therapy.<br>• In patients with other B-NHL subtypes that have relapsed after or are refractory to at least 2 prior lines of systemic therapy. |
| **Secondary Objectives:** | The secondary objectives of this study are:<br>• To assess the anti-tumor activity of single agent odronextamab in each of the 5 disease-specific cohorts, as measured by:<br>  • ORR according to the Lugano Classification and as assessed by local investigator evaluation.<br>  • Complete response (CR) rate according to the Lugano Classification and as assessed by local investigator evaluation and by independent central review.<br>  • Progression free survival (PFS) according to Lugano Classification and as assessed by:<br>    ▪ Independent central review, and<br>    ▪ Local investigator evaluation<br>  • Overall survival (OS)<br>  • Duration of response (DOR) according to the Lugano Classification and as assessed by:<br>    ▪ Independent central review, and<br>    ▪ Local investigator evaluation |



VV-RIM-00192456-1.0 Approved - 11 Apr 2022 GMT-5:00

| | |
|---|---|
| | - Disease control rate (DCR) according to the Lugano Classification and as assessed by:<br>  ▪ Independent central review, and<br>  ▪ Local investigator evaluation<br>- To evaluate the safety and tolerability of odronextamab<br>- To assess the pharmacokinetics (PK) of odronextamab<br>- To assess the immunogenicity of odronextamab<br>- To assess the effect of odronextamab on patient reported outcomes, including health-related quality of life (HRQL), as measured by the validated instruments European Organisation for Research and Treatment of Cancer Quality of Life Questionnaire (EORTC QLQ-C30), Functional Assessment of Cancer Therapy-Lymphoma (FACT-Lym), and EuroQoL 5 Dimensions 3 Levels (EQ-5D-3L). |
| **Study Design** | This is a phase 2, open-label, multi-cohort, multi-center study of odronextamab administered as an IV infusion in patients with B-NHL that has relapsed after or is refractory to prior systemic therapy. The study consists of 5 disease-specific cohorts, each with independent parallel enrollment. Patients in the DLBCL cohort will be randomized 1:1 to either Arm 1 or Arm 2 in the initial step of this cohort with 2 different odronextamab dose regimens. After the initial step, enrollment will only continue in the 160 mg QW/ 320 mg Q2W arm until a total of 112 patients (up to approximately 127 patients, including the randomized patients in the arm) are reached for that dose level. In each disease-specific cohort, each patient will follow the same study flow beginning with a screening period of up to 28 days. The screening period begins with the signing of the informed consent form (ICF) and ends when the patient has been confirmed to be eligible for the study and initiates treatment, or with the determination that the patient is ineligible and has been designated as a screen failure. Cohort assignments will be based on the patient's diagnosis and treatment history at the time of study enrollment.<br><br>The treatment period will comprise 12 weekly doses followed by every 2 weeks (Q2W) dosing until the time of disease progression or other protocol-defined reason for treatment discontinuation. In addition, if a patient has demonstrated a CR and has shown a durable response for at least 9 months after the initial determination of CR, then the frequency of study drug administration at the assigned dose will be decreased from Q2W to once every 4 weeks (Q4W) intervals, based on local investigator evaluation. Patients must have received the assigned QW full (nominal) dose at the Q2W dosing schedule for at least 3 preceding doses before switching from Q2W to Q4W dosing.<br><br>Patients will be followed for efficacy until the time of disease progression or start of non-protocol anti-lymphoma therapy.<br><br>Odronextamab is administered as a single agent intravenously (IV) at an initial split dose of 0.7 mg (0.2/0.5 mg), followed by an intermediate split dose-1 of 4 mg and then an intermediate split dose-2 of 20 mg. Subsequently, dosing in each disease-specific cohort is as follows:<br>- In the FL grade 1-3a and MZL cohorts, odronextamab will be administered at a QW full dose of 80 mg during QW dosing |

| | |
|---|---|
| | followed by 160 mg during Q2W dosing. Enrollment of patients in the MZL cohort is on pause as of global amendment 4.<br>• In the MCL cohort and Other B-NHL cohorts, odronextamab will be administered at a QW full dose of 160 mg during QW dosing followed by 320 mg during Q2W dosing. Enrollment of patients in the MCL cohort is on pause as of global amendment 3.<br>• Patients in the DLBCL cohort will be randomized 1:1 to either Arm 1 or Arm 2 in the initial step of this cohort with 2 different odronextamab dose regimens. Patients in Arm 1 will receive a QW full dose of 160 mg during QW dosing followed by 320 mg during Q2W dosing. Patients in Arm 2 will receive a QW full dose of 320 mg during QW dosing followed by 320 mg during Q2W dosing. Subsequent enrollment will proceed only in the 160 mg QW/320 mg Q2W arm until 112 patients (up to approximately 127 patients) are reached in this dose level, and enrollment into the other arm will close. Enrollment in the 320 mg cohort is complete as of global amendment 3.<br><br>In each disease-specific cohort, enrollment follows an open-label, design. |
| **Study Duration** | The treatment duration comprises 12 weekly doses followed by Q2W dosing until the time of disease progression or other protocol-defined reason for treatment discontinuation. In addition, if a patient has demonstrated a CR and has shown a durable response for at least 9 months after the initial determination of CR, then the frequency of study drug administration at the assigned dose will be decreased from Q2W to Q4W intervals, based on local investigator evaluation. Patients must have received the assigned QW full dose at the Q2W dosing schedule for at least 3 preceding doses before switching from Q2W to Q4W dosing.<br><br>The post-treatment follow-up period will begin after the last dose of study treatment. All patients will be followed for safety Q4W until 12 weeks following the last dose. For patients who have discontinued study treatment for any reason other than disease progression, disease response will be assessed at the protocol-specified timepoints during the extended post-treatment follow-up period until the time of disease progression, death, start of non-protocol anti-lymphoma therapy, or patient withdrawal of consent for follow-up, whichever is sooner. All patients will be followed in the survival follow up period every 12 weeks for survival status until the time of death, loss to follow-up, patient withdrawal of consent for follow-up, or study termination by the sponsor, whichever is earliest. |
| **End of Study Definition** | The study and all follow-up will end when all study patients have been discontinued from the study or at the time of study closure by the sponsor, whichever is earlier. The end of study for an individual patient will be at the time when the patient discontinues from the study up to the end of the extended follow-up and prior to survival follow-up. All patients will continue survival follow-up until death, loss to follow-up, withdrawal of consent for follow-up, or study termination by the sponsor, whichever is earliest. |
| **Population** | |
| **Sample Size:** | In the main global study, up to a total of approximately 512 patients are planned for enrollment and treatment across 5 disease-specific cohorts at multiple sites:<br>• 128 patients with FL grade 1-3a |

| | |
|---|---|
| | • 161 patients with DLBCL (a total of 68 randomized patients in Arm 1 and Arm 2, and a total of 112 patients (up to approximately 127 patients) in the 160 mg QW/320 mg Q2W dose regimen)<br>• 78 patients with MCL after failure of BTK inhibitor therapy<br>• 78 patients with MZL<br>• 67 patients with other B-NHL subtypes<br>Applicable for Japan: Enrollment in Japan will include approximately 12 patients each, in the FL grade 1-3a cohort and the DLBCL cohort at the recommended dosing regimens. Any remaining patients in Japan exceeding the global enrollment for the FL grade 1-3a and DLBCL cohorts will be enrolled into a Japan extension for these cohorts.<br>Applicable for China: Enrollment in China will include approximately 20 patients in the FL grade 1-3a cohort and 26 patients in the DLBCL cohort at the recommended dosing regimens. Any remaining patients in China exceeding the global enrollment for the FL grade 1-3a and DLBCL cohorts will be enrolled into a China extension for these cohorts.<br>If the modified step-up regimen is not deemed acceptable in a specific cohort (ie, FL grade 1-3a, DLBCL/Other B-NHL) per interim safety assessment on grade ≥ 3 CRS, enrollment will be stopped in all cohorts, safety data will be analyzed and a revised regimen will be implemented in subsequent amendment. |
| **Target Population:** | The study population will consist of patients aged 18 years and older.<br>The study population for each of the 5 separate independent enrolling disease-specific cohorts will consist of:<br>• FL grade 1-3a cohort: patients with FL that has relapsed or is refractory to at least 2 prior lines of systemic therapy, including an anti-CD20 antibody and an alkylating agent; patients must have failed combination lenalidomide and rituximab treatment where approved or deemed not appropriate to receive this treatment according to the investigator.<br>• DLBCL cohort: patients with DLBCL that has relapsed or is refractory to at least 2 prior lines of systemic therapy, including an anti-CD20 antibody and an alkylating agent. Patients with de novo DLBCL or DLBCL that is transformed from a lower grade neoplasm (eg, FL or CLL) may be enrolled. Patients with DLBCL transformation from prior CLL can only be enrolled in the absence of a leukemic CLL component. For patients with transformed DLBCL, prior systemic therapies administered for the lower grade neoplasm will not be considered among the prior lines of therapy for the purpose of determining eligibility.<br>• MZL cohort: patients with MZL that has relapsed or is refractory to at least 2 prior lines of systemic therapy. New enrollment is paused for any patient with MZL as of global amendment 4 until safety of the modified step-up regimen has been optimized in other subtypes. Currently enrolled patients who are receiving clinical benefit as per investigator may continue treatment with odronextamab as per protocol.<br>• Other B-NHL cohort: patients with other B-NHL subtypes (excluding WM, SLL, CLL, Burkitt lymphoma and Burkitt-like lymphoma with 11q aberration) that have relapsed or are refractory to at least 2 prior lines of systemic therapy. |

| | |
|---|---|
| | - Patients with a current diagnosis of mixed histology of B-NHL with an aggressive component (such as concurrent FL and DLBCL) will be allowed.<br>• MCL after BTK inhibitor therapy cohort: new enrollment is paused for any patient with MCL as of global amendment 3 until further risk mitigation measures are put in place for this patient population. Currently enrolled patients who are receiving clinical benefit as per investigator may continue dosing with odronextamab as per protocol. |
| **Treatment** | |
| **Study Drug: Dose/Route/Schedule:** | REGN1979 (odronextamab) will be administered by IV infusion at an initial dose of 0.7 mg during treatment week 1 split as 0.2 mg on day 1 and 0.5 mg on day 2, an intermediate dose-1 of 4 mg during treatment week 2 (equal split infusions over 2 days), intermediate dose-2 of 20 mg (equal split infusions over 2 days) during treatment week 3, and then the assigned QW dose from treatment week 4 through week 12 followed by assigned Q2W dose. In the FL grade 1-3a and MZL cohorts, odronextamab will be administered at a dose of 80 mg during QW dosing and 160 mg during Q2W dosing. In the MCL cohort and other B-NHL cohort, odronextamab will be administered at a dose of 160 mg during QW dosing and 320 mg during Q2W dosing. In the DLBCL cohort, patients in Arm 1 will receive a dose of 160 mg QW followed by 320 mg during Q2W dosing. Patients in Arm 2 will receive a dose of 320 mg during QW dosing followed by 320 mg during Q2W dosing.<br><br>The initial dose (0.7 mg [0.2/0.5 mg]), intermediate dose-1 of 4 mg, and intermediate dose-2 of 20 mg are always administered as a split infusion over 2 days, preferably consecutive but no more than 3 days apart, even if these doses were delayed beyond treatment week 3. Administration of the QW full (nominal) dose will only proceed if the full initial, full intermediate dose-1 and intermediate dose-2 were received and tolerated [Note: Patients who received >90% of the intended dose are considered to have received the full dose].<br><br>In patients who do not experience grade 3 cytokine release syndrome (CRS) with the initial dose, intermediate dose-1 and intermediate dose-2, the full QW dose of odronextamab will be administered as single infusion. Patients should have received full initial dose prior to intermediate dose-; patients should have received full intermediate dose-1 prior to intermediate dose-2 and should have received full intermediate dose-2 prior to receiving full single infusion. Doses beyond treatment week 4, odronextamab will be administered as a single infusion. However, if a patient experiences grade 3 CRS with the initial dose, the intermediate dose-1 or intermediate dose-2, then the first odronextamab QW full dose will be administered as a split infusion over 2 days.<br><br>If the administration of the first full QW dose is tolerated as a single infusion, subsequent doses may be administered as a single infusion over 1 to 4 hours depending on previous tolerability. |
| **Endpoint(s)** | |
| **Primary:** | The primary endpoint for each of the 5 disease-specific cohorts is the ORR according to the Lugano Classification of response in malignant lymphoma |

| | |
|---|---|
| | (Cheson, 2014) and as assessed by independent central review. The ORR is assessed from the time of the first patient first dose until all patients have completed 52 weeks tumor assessment in FL/MZL, 36 weeks tumor assessment in DLBCL/MCL/Other B-NHL or have withdrawn from the study. |
| **Secondary:** | The secondary endpoints for each of the 5 disease-specific cohorts are:<br>• ORR according to the Lugano Classification and as assessed by local investigator evaluation. The ORR is assessed from the time of the first patient first dose until all patients have completed 52 week tumor assessment in FL/MZL, 36 week tumor assessment in DLBCL/MCL/Other B-NHL or have withdrawn from the study.<br>• Complete response (CR) rate according to the Lugano Classification and as assessed by local investigator evaluation and independent central review. The CR rate is assessed from the time of the first patient first dose until patients have completed 52 week tumor assessment for FL grade 1-3a/MZL and 36 week tumor assessment for DLBCL/MCL/Other B-NHLs or have withdrawn from the study.<br>• DCR is assessed from the time of the first patient first dose until patients have completed 52 week tumor assessment for FL grade 1-3a/MZL and 36 week tumor assessment for DLBCL/MCL/Other B-NHLs or have withdrawn from the study.<br>• Pharmacokinetics: Concentration of odronextamab<br>• Immunogenicity: Incidence and titer of anti-drug antibodies (ADA) and incidence of neutralizing antibodies (NAb) to odronextamab over time<br>• Changes in scores of patient-reported outcomes, as measured by the validated instruments EORTC QLQ-C30, FACT-Lym, and EQ-5D-3L. Changes in patient-reported outcomes will be assessed from the time of the first patient first dose until the end of the study. |
| **Procedures and Assessments** | For all patients, the disease will be assessed radiologically using computed tomography (CT) or magnetic resonance imaging (MRI) and by 18F-fluorodeoxyglucose-positron emission tomography (FDG-PET) imaging. Tumor response according to the Lugano Classification criteria will be adjudicated by independent central radiology review. Bone marrow aspirate, bone marrow biopsy, and lymph node and/or tumor biopsy will be performed, and samples will be evaluated histologically and may be used for other studies, including for immunohistochemistry.<br><br>Safety will be evaluated by the assessment of vital signs, physical examination, Eastern Cooperative Oncology Group (ECOG) performance status, electrocardiogram (ECG), incidence of AEs, and reporting of concomitant medications. Laboratory evaluations include complete blood count with differential, blood chemistry values, serum immunoglobulins G (IgG), serum pregnancy testing (if relevant), ferritin, and C-reactive protein (CRP).<br><br>Blood samples for PK and anti-drug antibody (ADA) assessment will be collected. |

| | |
|---|---|
| | Peripheral blood samples will be collected to assess changes in biomarkers (eg, cytokine production, serum levels of pro-inflammatory cytokines, and changes in lymphocyte subsets and activation status). In addition, these samples will permit tumor or somatic genetic analyses for variations that impact the clinical course of underlying disease or modulate treatment side effects.<br><br>Quality of life assessments will be performed electronically using the self-administered EORTC QLQ-C30, FACT-Lym, and EQ-5D-3L questionnaires. |
| **Statistical Plan** | This study is designed to evaluate the efficacy and safety of odronextamab in patients with relapsed or refractory B-NHL in 5 disease-specific cohorts. The final analysis for the primary efficacy endpoint will be performed for each disease-specific cohort separately after all patients in the cohort have completed 52 weeks tumor assessment in FL/MZL, 36 weeks tumor assessment in DLBCL/MCL/Other B-NHL, or have withdrawn from the study. |

**Justification of Sample Size**

- **FL Grade 1-3a Cohort**

A single-stage exact binomial design is adopted for the primary endpoint of ORR. The 2-sided 95% confidence intervals for the observed ORRs were calculated based on the original sample size of 112. The enrollment in this cohort will continue beyond 112 patients in order to have at least 60 patients treated with the modified step-up regimen. Assuming that a clinically meaningful ORR is greater than 49%, with 112 patients an ORR of at least 59% will have a lower bound of the confidence interval that excludes 49%. In addition, if the observed ORR is at least 65%, 70%, and 75%, the lower limit of 95% CI will exclude the ORR of 55%, 60% and 65%, respectively (ie, the ORR will be significantly different from 55%, 60%, and 65%, as shown in the table below).

**The 2-sided 95% Exact Confidence Intervals for Observed ORR Given a Sample Size of 112 Patients**

| Number of Responders | Observed ORR | 95%CI – lower | 95% CI – upper |
|---|---|---|---|
| 50 | 0.45 | 0.352 | 0.543 |
| 56 | 0.50 | 0.404 | 0.596 |
| 62 | 0.55 | 0.457 | 0.648 |
| 66 | 0.59 | 0.492 | 0.681 |
| 67 | 0.60 | 0.501 | 0.690 |
| 73 | 0.65 | 0.556 | 0.739 |
| 78 | 0.70 | 0.602 | 0.780 |
| 84 | 0.75 | 0.659 | 0.827 |

With a sample size of 112 patients, if the true treatment effect of odronextamab is 64%, 70%, 75%, or 80%, the probability is 89%, 89%, 92%, or 92%, for the observed lower bound of the 95% CI to exclude 49%, 55%, 60%, or 65% respectively.

The enrollment in this cohort will continue beyond 112 patients in order to have at least 60 patients treated with the modified step-up regimen. With the additional patients, the probability stated above will be higher.

- **DLBCL Cohort**



VV-RIM-00192456-1.0 Approved - 11 Apr 2022 GMT-5:00

As an initial step, up to 68 patients will be randomized into the 2 treatment arms with a 1:1 ratio (Arm 1: 160 mg QW dosing followed by 320 mg Q2W dosing; Arm 2: 320 mg QW dosing followed by 320 mg Q2W dosing). Data from the initial step will be used for descriptive analyses that support PK/pharmacodynamic and advance our understanding of the exposure-response relationship for odronextamab. After the initial step, enrollment will only continue in the 160 mg QW/ 320 mg Q2W arm until a total of 112 patients (up to approximately 127 patients including the randomized patients in the arm) are reached to evaluate efficacy at this selected dose regimen. The enrollment in this cohort will continue beyond 112 patients in order to have at least 60 patients of DLBCL or Other aggressive B-NHL (minimum 45 DLBCL patients) treated with the modified step-up regimen.

An exact binomial design will be adopted for the primary endpoint of ORR. The 2-sided 95% confidence intervals for the observed ORR were calculated based on a sample size of 112. Assuming that a clinically meaningful ORR is greater than 35%, with 112 patients an ORR of at least 45% will have a lower bound of the confidence interval that excludes 35%. In addition, if the observed ORR is at least 50%, 55% and 60%, the lower limit of 95% CI will exclude the ORR of 40%, 45% and 50% respectively (ie, the ORR is significantly different from 40%, 45% and 50%).

With a sample size of 112 patients, if the true treatment effect of odronextamab is 50%, 55%, 60% or 65%, the probability is 89%, 88%, 86% or 89% for the observed lower bound of 95% CI to exclude 35%, 40%, 45% and 50%, respectively. With the additional patients, the probability stated above will be higher.

- **MCL after BTK inhibitor Cohort**

An exact binomial design will be adopted for the primary endpoint ORR. The 2-sided 95% confidence intervals for the observed ORRs were calculated based on a sample size of 78. Assuming that a clinically meaningful ORR is greater than 15%, with 78 patients an ORR of at least 24% will have a lower bound of the confidence interval that excludes 15%. In addition, if the observed ORR is at least 36%, 41% and 47%, the lower limit of 95% CI will exclude the ORR of 25%, 30% and 36%, respectively (ie, the ORR rate is significantly different from 25%, 30% and 36%, as shown in the table below). No change to sample size in the MCL cohort is being made as it is on pause for enrollment.

**The 2-sided 95% Exact Confidence Intervals for Observed ORR Given a Sample Size of 78 Patients**

| Number of Responders | Observed ORR | 95%CI – lower | 95% CI – upper |
|---|---|---|---|
| 19 | 0.24 | 0.153 | 0.354 |
| 24 | 0.31 | 0.208 | 0.422 |
| 28 | 0.36 | 0.253 | 0.476 |
| 32 | 0.41 | 0.3001 | 0.527 |
| 37 | 0.47 | 0.3601 | 0.591 |
| 41 | 0.53 | 0.409 | 0.640 |

With a sample size of 78 patients, if the true treatment effect of odronextamab is 30%, 40%, 45%, or 51%, the probability is 89%, 80%, 79%, or 77% for the observed lower bound of 95% CI to exclude 15%, 25%, 30%, or 36%, respectively.

- **MZL Cohort**

An exact binomial design will be adopted for the primary endpoint ORR. The 2-sided 95% confidence intervals for the observed ORRs were calculated based on a sample size of 78. Assuming that a clinical meaningful ORR is greater than 20%, with 78 patients an ORR of at least 31% will have a lower bound of the confidence interval that excludes 20%. In addition, if the observed ORR is at least 41%, 47% and 53%, the lower limit of 95% CI will exclude the ORR of 30%, 36% and 40% respectively (ie, the ORR rate is significantly different from 30%, 36% and 40%).

With a sample size of 78 patients, if the true treatment effect of odronextamab is 35%, 45%, 51%, or 55%, the probability is 82%, 79%, 77%, or 71% for the observed lower bound of 95% CI to exclude 20%, 30%, 36%, or 40%, respectively. No change to sample size in the MCL cohort is being made as it is on pause for enrollment.

- **Other B-NHL Cohort**

An exact binomial design will be adopted for the primary endpoint of ORR. The 2-sided 95% confidence intervals for the observed ORRs were calculated based on a sample size of 67. Assuming that a clinical meaningful ORR is greater than 10%, with 67 patients, an ORR of at least 19% will have a lower bound of the confidence interval that excludes 10%. In addition, if the observed ORR is at least 31% and 43%, the lower limit of 95% CI will exclude the ORR of 20% and 31% respectively; (ie, the ORR is significantly different from 20% and 31%, as shown in the table below).

**The 2-sided 95% Exact Confidence Intervals for Observed ORR Given a Sample Size of 67 Patients**

| Number of Responders | Observed ORR | 95%CI – lower | 95% CI – upper |
|---|---|---|---|
| 13 | 0.19 | 0.108 | 0.309 |
| 21 | 0.31 | 0.206 | 0.438 |
| 29 | 0.43 | 0.312 | 0.560 |

With a sample size of 67 patients, if the true treatment effect of odronextamab is 25%, 35%, or 46%, the probability is 88%, 77%, or 71% for the observed lower bound of 95% CI to exclude 10%, 20%, or 31%, respectively.

**Statistical Methods**

The analyses will be performed by disease-specific cohort.

Demographic and baseline characteristics will be summarized descriptively.

The primary efficacy endpoint is the ORR according to the Lugano Classification, which is (Cheson, 2014) based on independent central review and will be summarized along with the 2-sided 95% confidence interval for each disease-specific cohort. Patients who are not evaluable for the best overall response will be considered to be non-responders.

The secondary efficacy endpoints of ORR as determined by investigator review, CR rate and DCR as determined by independent central review or by investigator review according to Lugano Classification (Cheson, 2014) will be summarized along with the respective 2-sided 95% confidence interval for each disease-specific cohort.

The other secondary efficacy endpoints, including DOR, PFS, and OS will be summarized by median and its 95% confidence interval using the Kaplan-



VV-RIM-00192456-1.0 Approved - 11 Apr 2022 GMT-5:00

| | |
|---|---|
| | Meier method according to Lugano Classification for each disease-specific cohort.<br>Quality of life measured by validated electronic instruments EORTC QLQ-C30, FACT-Lym, and EQ-5D-3L will be summarized using descriptive statistics.<br>Safety observations and measurements including drug exposure, AEs, laboratory data, vital signs, and ECOG performance status will be summarized and presented in tables and listings for each disease specific cohort by original dose regimen and modified dose regimen for the step-up dosing.<br>The final analysis for primary efficacy endpoint will be performed after all the patients have completed 52 weeks tumor assessment in FL grade 1-3a/MZL, 36 weeks tumor assessment in DLBCL/MCL/Other B-NHL or have withdrawn from the study earlier.<br>**Interim Analysis**<br>An interim efficacy analysis will be conducted when 80 patients with FL grade 1-3a have completed 52-week assessment and 127 patients in the DLBCL cohort have completed 36-week assessment or have withdrawn from the study, whichever is later. The ORR and associated 95% confidence interval will be summarized for FL grade 1-3a cohort. As the primary objective of this interim efficacy analysis is point estimation on ORR and characterization of the precision of point estimation, the study will not be stopped for perceived efficacy. For other efficacy endpoints, 2-sided 95% confidence interval will also be presented.<br><br>The sponsor will perform an administrative review for efficacy after at least 70 patients have been treated with the 0.7/4/20 mg step-up regimen and have completed a 4-week safety review. At the administrative review, summary of safety for all cohorts and summary of efficacy for the FL grade 1-3a and DLBCL cohorts will be performed.<br><br>An interim futility analysis will be performed in each of the MCL, MZL, and other B-NHL disease-specific cohorts after the pre-specified number of evaluable patients in the cohort have completed tumor assessments at 28 weeks or have withdrawn from the study earlier (see table below). The futility boundary is determined based on lack of clinically meaningful activity below the threshold in each disease-specific cohort. The enrollment for each disease-specific cohort will continue unless its futility boundary is crossed.<br>• MCL after BTK inhibitor therapy cohort: The futility boundary of 15% ORR is determined due to the lack of standard of care options for patients who have failed 2 or more lines of prior therapy. Therefore, if fewer than 5 responders are observed among the first 27 patients, this cohort will be stopped for futility.<br>• MZL cohort: The futility boundary of 20% ORR is determined due to enrollment of patients with relapsed/refractory disease following failure of standard of care therapies. Therefore, if fewer than 6 responders are observed among the first 27 patients, this cohort will be stopped for futility.<br>• Other B-NHL cohort: The futility boundary of 10% ORR is determined for the entire cohort due to the enrollment of heterogenous subtypes with aggressive lymphomas that have poor outcomes in relapsed/refractory setting. Due to low incidence, it is anticipated that there will be very few patients of each subtype in |

this cohort limiting formal statistical analysis of any particular subtype. Therefore, if fewer than 4 responders are observed among the first 34 evaluable patients, this cohort will be stopped for futility.

**Interim Futility Analysis**

| Study cohort | Total sample size N | Sample size at Interim Futility n1 | Interim futility stopping boundary |
|---|---|---|---|
| FL grade 1-3a | 128 | NA | NA |
| DLBCL | 112 to 127 | NA | NA |
| MZL | 78 | 27 | <6 responders |
| MCL after BTK inhibitor | 78 | 27 | <5 responders |
| Other B-NHL | 67 | 34 | <4 responders |



VV-RIM-00192456-1.0 Approved - 11 Apr 2022 GMT-5:00

# TABLE OF CONTENTS


AMENDMENT HISTORY ....3

CLINICAL STUDY PROTOCOL SYNOPSIS....33

LIST OF ABBREVIATIONS AND DEFINITIONS OF TERMS....53

1. INTRODUCTION ....56

1.1. Follicular Lymphoma ....56

1.1.1. Systemic Therapies for Relapsed or Refractory Follicular Lymphoma ....56

1.2. Diffuse Large B-Cell Lymphoma....58

1.2.1. Systemic Therapies for Relapsed or Refractory Diffuse Large B-Cell Lymphoma....58

1.3. Mantle Cell Lymphoma....59

1.3.1. Systemic Therapies for Relapsed or Refractory Mantle Cell Lymphoma....59

1.4. Marginal Zone Lymphoma....59

1.4.1. Systemic Therapies for Relapsed or Refractory Marginal Zone Lymphoma....60

1.5. Other B-cell non-Hodgkin Lymphomas ....60

1.6. Odronextamab Anti-CD20 x Anti-CD3 Bispecific Antibody ....60

1.7. Summary of In Vitro and In Vivo Pharmacology Studies....60

1.8. Clinical Background and Current Study....61

2. STUDY OBJECTIVES ....61

2.1. Primary Objective....61

2.2. Secondary Objectives ....62

2.3. Exploratory Objectives ....63

3. HYPOTHESIS AND RATIONALE ....64

3.1. Hypothesis ....64

3.2. Rationale....64

3.2.1. Rationale for Patient Population and Study Design ....64

3.2.2. Rationale for Dose Selection ....66

3.2.3. Rationale for Treatment Duration....69

4. STUDY ENDPOINTS AND VARIABLES....71

4.1. Endpoints....71

4.1.1. Primary Endpoint....71

4.1.2. Secondary Endpoints ....71

4.1.3. Exploratory Endpoints ....72

4.2. Study Variables....72
4.2.1. Demographic and Baseline Characteristics....72
4.2.2. Pharmacokinetic Variables....72
4.2.3. Immunogenicity Variables....72
5. STUDY DESIGN....73
5.1. Study Description....73
5.1.1. Study Conduct in Response to COVID-19....76
5.1.1.1. COVID-19 Vaccination During the Study....76
5.1.2. Screening Period....77
5.1.3. Treatment Period....77
5.1.3.1. Study Treatment....77
5.1.3.2. Treatment Delays....80
5.1.3.3. Study Assessments....80
5.1.3.4. Treatment in the Setting of Suspected Lymphoma Pseudo-Progression....80
5.1.3.5. Follow-up Period....81
5.1.4. Resumption of Treatment....82
5.1.5. End of Study Definition....82
5.2. Planned Interim Analysis....82
6. SELECTION, WITHDRAWAL, AND REPLACEMENT OF PATIENTS....83
6.1. Number of Patients Planned....83
6.2. Study Population....84
6.2.1. Inclusion Criteria....84
6.2.2. Exclusion Criteria....87
6.3. Study Committees....90
6.3.1. Independent Data Monitoring Committee....90
6.4. Premature Discontinuation of Study Medications and Premature Withdrawal from Study....90
6.5. Replacement of Patients....90
7. STUDY TREATMENTS....90
7.1. Investigational Treatment....90
7.2. Premedications....92
7.2.1. The following premedications apply to odronextamab from the initial dose through the first QW dose as a single infusion. If the patient experiences

IRR and/or CRS of any grade with first QW dose, continue premedications until the full QW dose is tolerated without experiencing IRR and/or CRS................92
7.2.2. First dose administration following QW full dose administered as a single infusion without experiencing an IRR and/ or CRS of any grade with 20 mg of dexamethasone IV ....................92
7.3. Dose Modification, Study Drug Discontinuation, and Management of Specific Toxicities ....................93
7.3.1. Study Treatment Discontinuation ....................93
7.3.1.1. Reasons for Permanent Discontinuation of Study Drug....................93
7.3.1.2. Restarting Study Drug Administration Following Temporary Treatment Interruption ....................94
7.3.2. Safety Monitoring and Management of Specific Toxicities....................94
7.3.2.1. Acute Allergic/Hypersensitivity Infusion Reactions ....................95
7.3.3. Grading and Management of Infusion-related Reaction and Cytokine Release Syndrome ....................96
7.3.3.1. Standard Intervention of Tocilizumab and Corticosteroid ....................96
7.3.3.2. Earlier Intervention of Tocilizumab and Enhanced Corticosteroid Pre- and Post- Medication ONLY Following Sponsor Notification....................102
7.3.4. B-Cell Depletion and Infection Prophylaxis....................104
7.3.4.1. Recommendations for Prophylaxis to Decrease the Risk of Infections: ....................104
7.3.5. Tumor Lysis Syndrome ....................105
7.3.5.1. Recommendations for Tumor Lysis Syndrome Prophylaxis....................105
7.3.5.2. Required Monitoring for Tumor Lysis Syndrome....................106
7.3.5.3. Dose Modification of Odronextamab for Patients who Experience Tumor Lysis Syndrome ....................108
7.3.6. Central Nervous System Toxicity....................109
7.4. Method of Treatment Assignment ....................109
7.4.1. Blinding ....................110
7.5. Treatment Logistics and Accountability....................110
7.5.1. Packaging, Labeling, and Storage ....................110
7.5.2. Supply and Disposition of Treatments ....................110
7.5.3. Treatment Accountability ....................110
7.5.4. Treatment Compliance....................110
7.6. Concomitant Medications and Procedures ....................110
7.6.1. Prohibited Medications....................111

7.6.2. Permitted Medications and Procedures ....................111
8. STUDY SCHEDULE OF EVENTS AND PROCEDURES ....................112
8.1. Schedule of Events ....................112
8.1.1. Footnotes for the Schedule of Events Table ....................117
8.1.2. Changes to the Schedule of Events/Study Procedures in Case of Dose Modification: ....................121
8.1.3. Footnotes for the Schedule of Events Detailed Sample Collection Time Points for Cytokines, Pharmacokinetics, Lymphocyte Immunophenotyping (Whole Blood), PBMC Immunophenotyping, C-Reactive Protein, and Ferritin ....................126
8.1.4. Early Termination Visit ....................127
8.1.5. Unscheduled Visits ....................127
8.2. Study Procedures ....................127
8.2.1. Procedures Performed Only at the Screening/Baseline Visit ....................127
8.2.2. Efficacy Procedures ....................128
8.2.2.1. Computed Tomography Imaging/Magnetic Resonance Imaging and $^{18}$F-Fluorodeoxyglucose-Positron Emission Tomography ....................128
8.2.2.2. Radiographic Disease Assessment ....................128
8.2.2.3. Bone Marrow Aspirate and Bone Marrow Biopsy ....................128
8.2.2.4. Lymph Node and/or Tumor Biopsy ....................129
8.2.3. Safety Procedures ....................129
8.2.3.1. Safety Monitoring ....................129
8.2.3.2. Vital Signs ....................129
8.2.3.3. Physical Examination ....................129
8.2.3.4. Assessment of B Symptoms ....................130
8.2.3.5. Performance Status ....................130
8.2.3.6. Electrocardiogram ....................130
8.2.3.7. Laboratory Testing ....................130
8.2.4. Pharmacokinetic Assessments ....................132
8.2.5. Anti-Drug Antibody Measurements and Samples ....................132
8.2.6. Survival Data Collection ....................132
8.2.7. Quality of Life Procedures ....................132
8.2.8. Pharmacodynamic and Exploratory Biomarker Procedures ....................132
8.2.8.1. Biomarker Assessments ....................132

8.2.8.2. Lymphocyte immunophenotyping (Whole Blood) Monitoring Sample ..................133
8.2.8.3. PBMC Immunophenotyping Sample..........................................................................133
8.2.8.4. Future Biomedical Research.........................................................................................133
8.2.8.5. Genomics Sub-study - Optional....................................................................................134
9. SAFETY DEFINITIONS, REPORTING, AND MONITORING ...........................134
9.1. Obligations of Investigator ...........................................................................................134
9.2. Obligations of Sponsor .................................................................................................134
9.3. Definitions ....................................................................................................................135
9.3.1. Adverse Event...............................................................................................................135
9.3.2. Serious Adverse Event...................................................................................................135
9.3.3. Adverse Events of Special Interest ...............................................................................136
9.3.4. Infusion Reactions and Cytokine Release Syndrome ...................................................136
9.4. Recording and Reporting Adverse Events....................................................................136
9.4.1. Adverse Events .............................................................................................................136
9.4.2. Serious Adverse Events ................................................................................................137
9.4.3. Other Events that Require Accelerated Reporting to Sponsor ......................................137
9.4.4. Reporting Adverse Events Leading to Withdrawal from the Study ..............................138
9.4.5. Abnormal Laboratory, Vital Signs, or Electrocardiogram Results................................138
9.4.6. Follow-up......................................................................................................................138
9.5. Evaluation of Severity and Causality ............................................................................138
9.5.1. Evaluation of Severity ..................................................................................................138
9.5.2. Evaluation of Causality.................................................................................................139
9.6. Safety Monitoring.........................................................................................................141
9.7. Investigator Alert Notification......................................................................................141
10. STATISTICAL PLAN...................................................................................................142
10.1. Statistical Hypothesis....................................................................................................142
10.2. Justification of Sample Size.........................................................................................142
10.2.1. Follicular Lymphoma Grade 1-3a Cohort ....................................................................142
10.2.2. Diffuse Large B-Cell Lymphoma.................................................................................143
10.2.3. Mantle Cell Lymphoma after BTK inhibitor therapy Cohort........................................144
10.2.4. Marginal Zone Lymphoma Cohort................................................................................145
10.2.5. Other B-Cell non-Hodgkin Lymphoma Cohort.............................................................145
10.3. Analysis Sets.................................................................................................................147

10.3.1. Efficacy Analysis Sets ....147
10.3.2. Safety Analysis Set ....147
10.3.3. Pharmacokinetic Analysis Set ....147
10.3.4. Immunogenicity Analysis Set ....147
10.4. Statistical Methods ....147
10.4.1. Patient Disposition ....147
10.4.2. Demography and Baseline Characteristics ....148
10.4.3. Efficacy Analyses ....148
10.4.3.1. Primary Efficacy Analysis ....148
10.4.3.2. Secondary Efficacy Analysis ....148
10.4.4. Safety Analysis ....149
10.4.4.1. Adverse Events ....149
10.4.4.2. Stopping Rule for Grade ≥3 CRS ....150
10.4.4.3. Other Safety Assessments ....152
10.4.4.4. Treatment Exposure ....152
10.4.4.5. Treatment Compliance ....152
10.4.5. Pharmacokinetics ....152
10.4.5.1. Analysis of Drug Concentration Data ....152
10.4.6. Analysis of Immunogenicity Data ....153
10.4.7. Analysis of Quality of Life Data ....153
10.4.8. Analysis of Biomarker Data ....154
10.4.9. Pharmacokinetic/Pharmacodynamic Analyses ....154
10.4.10. Exposure-Response Analysis ....154
10.5. Interim Analysis ....154
10.6. Additional Statistical Data Handling Conventions ....155
10.7. Statistical Considerations Surrounding the Premature Termination of a Study ....156
11. DATA MANAGEMENT AND ELECTRONIC SYSTEMS ....156
11.1. Data Management ....156
11.2. Electronic Systems ....156
12. STUDY MONITORING ....156
12.1. Monitoring of Study Sites ....156
12.2. Source Document Requirements ....157
12.3. Case Report Form Requirements ....157

13. AUDITS AND INSPECTIONS ....157
14. ETHICAL AND REGULATORY CONSIDERATIONS ....158
14.1. Good Clinical Practice Statement ....158
14.2. Informed Consent ....158
14.3. Patients Confidentiality and Data Protection ....158
14.4. Ethics Committee ....159
14.5. Clinical Study Data Transparency ....159
15. PROTOCOL AMENDMENTS ....159
16. PREMATURE TERMINATION OF THE STUDY OR CLOSE-OUT OF A SITE ....159
16.1. Premature Termination of the Study ....159
16.2. Close-out of a Site ....159
17. STUDY DOCUMENTATION ....160
17.1. Certification of Accuracy of Data ....160
17.2. Retention of Records ....160
18. DATA QUALITY ASSURANCE ....160
19. CONFIDENTIALITY ....162
20. FINANCING AND INSURANCE ....162
21. PUBLICATION POLICY ....162
22. REFERENCES ....163
23. INVESTIGATOR'S AGREEMENT ....169
SIGNATURE OF SPONSOR'S RESPONSIBLE OFFICERS ....174

# LIST OF TABLES

Table 1: Study Drug QW Dose and Q2W Dose for Each Disease-Specific Cohort ....91
Table 2: Cytokine Release Syndrome Toxicity Grading* ....97
Table 3: Management Guidance and Odronextamab Dosing in Setting of Cytokine-Mediated Toxicities- Infusion-related Reactions (IRR) ....98
Table 4: Management Guidance and Odronextamab Dosing in Setting of Cytokine Mediated Toxicities: Cytokine Release Syndrome (CRS) and Other Adverse Events ....99
Table 5: Guidelines for Administration of Tocilizumab and Corticosteroids in the Setting of Cytokine Release Syndrome ....101

Table 6: Guidelines for EARLIER Administration of Tocilizumab in the Setting of Cytokine Release Syndrome ....103
Table 7: Tumor Lysis Syndrome Risk Classification and Monitoring ....106
Table 8: Diagnosis of Laboratory and Clinical Tumor Lysis Syndrome* ....107
Table 9: Management Guidance and Study Drug Dosing in Setting of Tumor Lysis Syndrome ....108
Table 10: Schedule of Events ....113
Table 11: Schedule of Events Detailed Sample Collection Time Points for Cytokines, Pharmacokinetics, Lymphocyte Immunophenotyping (Whole Blood), PBMC Immunophenotyping, C-Reactive Protein, and Ferritin ....123
Table 12: NCI-CTCAE Severity Grading System for Adverse Events ....139
Table 13: The 2-sided 95% Exact Confidence Intervals for Observed ORR Given a Sample Size of 112 Patients ....143
Table 14: The 2-sided 95% Exact Confidence Intervals for Observed ORR Given a Sample Size of 78 Patients ....144
Table 15: The 2-sided 95% Exact Confidence Intervals for Observed ORR Given a Sample Size of 67 Patients ....146
Table 16: Sample Size at Original and Modified Step-up Regimens in Each Cohort ....146
Table 17: Percent of Patients with Grade ≥2 CRS with 1-Sided 80% Confidence Interval ....152
Table 18: Interim Futility Analysis ....155

# LIST OF FIGURES

Figure 1: Study Schema ....73
Figure 2: Schema for CRS Interim Safety Assessments ....75
Figure 3: Patient Study Flow Diagram ....76
Figure 4: Odronextamab Treatment for FL grade 1-3a Cohort ....79
Figure 5: Odronextamab Treatment for DLBCL Cohort Arm 1 and Other B-NHL Cohort ....79

# LIST OF APPENDICES

APPENDIX 1. EASTERN COOPERATIVE ONCOLOGY GROUP CRITERIA ..................170

APPENDIX 2. MALIGNANT LYMPHOMA RESPONSE DEFINITIONS PER LUGANO CRITERIA ..........................................................................171

APPENDIX 3. LYMPHOMA RESPONSE TO IMMUNOMODULATORY THERAPY CRITERIA (LYRIC) CRITERIA .................................................173



VV-RIM-00192456-1.0 Approved - 11 Apr 2022 GMT-5:00

# LIST OF ABBREVIATIONS AND DEFINITIONS OF TERMS

| | |
|---|---|
| ADA | Anti-drug antibody |
| ADCC | Antibody-dependent cell-mediated cytotoxicity |
| ADL | Activities of daily living |
| ADR | Adverse drug reactions |
| AE | Adverse event |
| AESI | Adverse event of special interest |
| ALL | Acute lymphoblastic leukemia |
| ALT | Alanine aminotransferase |
| ASTCT | American Society for Transplantation and Cellular Therapy |
| AST | Aspartate aminotransferase |
| B-NHL | B-cell non-Hodgkin lymphoma |
| BOR | Best Overall Response |
| BR | Bendamustine + rituximab |
| BTK | Bruton’s tyrosine kinase |
| BUN | Blood urea nitrogen |
| CAR-T | Chimeric antigen receptor T cells |
| CHOP | Cyclophosphamide, doxorubicin, vincristine, and prednisone |
| CDC | Complement dependent cytotoxicity |
| CLL | Chronic lymphocytic leukemia |
| CMV | Cytomegalovirus |
| CNS | Central nervous system |
| CR | Complete response |
| CRF | Case report form (electronic or paper) |
| CRO | Contract research organization |
| CRP | C-reactive protein |
| CRS | Cytokine release syndrome |
| CTCAE | Common Terminology Criteria for Adverse Events |
| CVP | cyclophosphamide, vincristine, and prednisone |
| DCR | Disease control rate |
| DLBCL | Diffuse large B-cell lymphoma |
| DLT | Dose-limiting toxicity |
| DOR | Duration of response |
| EC | Ethics Committee |
| ECG | Electrocardiogram |
| ECOG | Eastern Cooperative Oncology Group |
| EDC | Electronic data capture |

| | |
|---|---|
| EOI | End of infusion |
| EORTC QLQ-C30 | European Organisation for Research and Treatment of Cancer Quality of Life Questionnaire |
| EQ-5D-3L | EuroQoL 5 Dimensions 3 Levels |
| E-R | Exposure-response |
| EU | European Union |
| FACT-Lym | Functional Assessment of Cancer Therapy–Lymphoma |
| FAS | Full analysis set |
| FDG-PET | $^{18}$F-fluorodeoxyglucose-positron emission tomography |
| FL | Follicular lymphoma |
| FLIPI | Follicular Lymphoma International Prognostic Index |
| GCP | Good Clinical Practice |
| HBV | Hepatitis B virus |
| HCV | Hepatitis C virus |
| HIV | Human immunodeficiency virus |
| HRQL | Health-related Quality of Life |
| ICF | Informed consent form |
| ICH | International Council for Harmonisation |
| IRB | Institutional Review Board |
| IRR | Infusion-related reaction |
| IV | Intravenous |
| IWG | International Working Group |
| LDH | Lactate dehydrogenase |
| LYRIC | Lymphoma response to immunomodulatory therapy criteria |
| mAb | Monoclonal antibody |
| MCL | Mantle Cell Lymphoma |
| MedDRA | Medical Dictionary for Regulatory Activities |
| MRD | Minimal residual disease |
| MZL | Marginal Zone Lymphoma |
| NAb | Neutralizing antibody |
| NCA | Non-compartmental analysis |
| NCI-CTCAE | National Cancer Institute-Common Terminology Criteria for Adverse Events |
| NHL | Non-Hodgkin lymphoma |
| NOS | Not otherwise specified |
| ORR | Objective response rate |
| OS | Overall survival |
| PBMC | Peripheral blood mononuclear cell |

| | |
|---|---|
| PD | Progressive disease |
| PD-1 | Programmed Death-1 |
| PE | Physical exam |
| PFS | Progression-free survival |
| PI3K | Phosphatidylinositol 3-kinase |
| PK | Pharmacokinetic |
| PPD | Product of the perpendicular diameters |
| PR | Partial response |
| PT | Preferred term |
| QW | Once every week |
| Q2W | Once every 2 weeks |
| Q4W | Once every 4 weeks |
| Q12W | Once every 12 weeks |
| RBC | Red blood cell |
| R-CHOP | Rituximab in combination with chemotherapy – cyclophosphamide, doxorubicin, vincristine, prednisone |
| R-CVP | Rituximab in combination with chemotherapy – cyclophosphamide, vincristine, prednisone |
| Regeneron | Regeneron Pharmaceuticals, Inc. |
| SAE | Serious adverse event |
| SAF | Safety analysis set |
| SAP | Statistical Analysis Plan |
| SAS | Statistical Analysis System software |
| SCT | Stem cell transplant |
| SD | Stable disease |
| SOC | System organ class |
| SPD | Sum of the perpendicular diameters |
| SUSAR | Suspected unexpected serious adverse reaction |
| TEAE | Treatment-emergent adverse event |
| ULN | Upper limit of normal |
| US | United States |
| TLS | Tumor lysis syndrome |
| WBC | White blood cell |
| WHO | World Health Organization |
| WM | Waldenström macroglobulinemia |



VV-RIM-00192456-1.0 Approved - 11 Apr 2022 GMT-5:00

# 1. INTRODUCTION

Non-Hodgkin lymphomas (NHL) comprise a heterogeneous group of malignancies with lymphoid characteristics that arise from hematopoietic progenitor cells; collectively, NHL neoplasms comprise the seventh most common malignancy and account for approximately 4.5% of all cancers occurring in the US. In the US, there will be approximately 74,200 new cases and 19,970 deaths due to NHL in 2019 (Siegel, 2019). In Europe, the estimated incidence of NHL in 2018 was 115,118 with a mortality of 48,096 (Global Cancer Observatory, 2020). The largest proportion of NHLs (>90%) are of B-lymphoid origin, and the remainder are T/NK-lymphomas or are of indeterminate or mixed lineage (Teras, 2016). NHLs are commonly grouped into those that exhibit an initially indolent clinical course (such as follicular lymphoma [FL] and marginal zone lymphoma [MZL]) and those that are typically aggressive (such as diffuse large B-cell lymphoma [DLBCL]) at the time of initial presentation.

This study will include 5 disease-specific B-NHL cohorts and one cohort comprising a diverse group of additional B-NHL subtypes. In each cohort, the safety and efficacy of odronextamab will be evaluated.

## 1.1. Follicular Lymphoma

Among the most common NHL subtypes are those of mature B-cells (B-NHL) (Swerdlow, 2017), and among these follicular lymphoma (FL) is the most prevalent. In 2016, the estimated number of new cases of FL in the US was 13,530 (Teras, 2016). The disease is rare in children and adolescents, and the incidence increases with age; the median age of diagnosis is between 60 and 65 years.

The initial treatment of advanced FL provides meaningful responses in a large proportion of patients, but high-risk groups have been identified in which the response duration is typically brief. In a retrospective evaluation of patients with previously untreated advanced FL, patients with high risk clinical and molecular features had a 5-year failure-free survival of 25.0 to 38.3% (Pastore, 2015). In patients prospectively evaluated in the National LymphoCare Study who received front-line chemo-immunotherapy for FL, 20% of patients experienced progressive disease within 2 years of diagnosis, and for these patients the 5-year overall survival (OS) was 50% (Casulo, 2015); other retrospective evaluations have confirmed this finding (Maurer, 2016). Despite the effectiveness of chemo-immunotherapy in the first-line treatment setting, relapses occur in almost all patients; response durations are progressively shorter with successive lines of therapy, and disease relapse is the cause of death in most patients (Sorigue, 2016). In addition, transformation to a more clinically aggressive large cell lymphoma occurs at a rate of approximately 2 to 3% per year in the era of chemo-immunotherapy treatment (eg, rituximab with chemotherapy) (Wagner-Johnston, 2015).

### 1.1.1. Systemic Therapies for Relapsed or Refractory Follicular Lymphoma

As noted above, patients with advanced FL generally are not cured with available therapies. The standard of care for patients with newly diagnosed advanced stage symptomatic FL or disease with evidence of organ impairment includes an anti-CD20 antibody (eg, obinutuzumab or rituximab) with multi-agent chemotherapy comprising cyclophosphamide, doxorubicin, vincristine, and prednisone, collectively (termed “CHOP”); obinutuzumab or rituximab with cyclophosphamide, vincristine, and prednisone (termed “CVP”) in patients unfit for anthracycline therapy; or

obinutuzumab or rituximab with the antimetabolite and alkylating agent bendamustine. With the immuno-chemotherapy regimens, overall response rates of more than 90% with complete remissions of 20% to 60% and median progression-free survival (PFS) exceeding 4 to 5 years have been observed (Marcus, 2017) (Hiddemann, 2014).

Despite the achievement of response in some patients, the relapse rate for FL has remained constant over time and is the principal cause of death (Sorigue, 2016). In the relapsed setting, long-lasting remissions can be achieved by salvage therapy followed by autologous stem cell transplantation (Hiddemann, 2014), but this therapeutic modality is not available to all patients given the occurrence of co-morbidities. For second-line, lower-grade FL, chemo-immunotherapy regimens with an anti-CD20 antibody are commonly used. Single-agent rituximab, bendamustine, single-agent chemotherapy, radio-immunotherapy, or obinutuzumab-bendamustine are used. However, CR rates are low, and the median PFS is short (9.3 months overall for indolent B-NHL) (Cheson, 2010) (Friedberg, 2008) (Kahl, 2010). In a randomized phase 3 study (AUGMENT), the combination of lenalidomide and rituximab was associated with a significant improvement in the median PFS when compared to placebo and rituximab in patients with relapsed or refractory indolent lymphoma (with FL comprising the largest enrolled disease subtype) who received at least one prior line of therapy. Based on this data, lenalidomide in combination with rituximab was approved in US for the treatment of adult patients with previously treated FL (REVLIMID® [Package Insert], 2019). Notably, patients with rituximab-refractory disease were excluded in this study (Leonard, 2019).

For patients who develop disease that is refractory to anti-CD20-based therapy, the only approved treatment option is phosphatidylinositol 3-kinase (PI3K) inhibitor therapy. Idelalisib received standard approval in the EU (ZYDELIG®, Prescribing Information, 2018)[1] for patients with FL who have received at least 2 prior lines of systemic therapy; the objective response rate (ORR) was 54% (complete response [CR] was 8%), and the drug carries black box warnings including fatal and/or serious hepatotoxicity, diarrhea or colitis, pneumonitis, and infections, among others. Copanlisib, a PI3K alpha/delta isoform inhibitor, received accelerated approval in the US based on the phase 2 CHRONOS-1 study in patients who had relapsed or were refractory to ≥2 prior lines of treatment and who were previously treated with rituximab and an alkylating agent. The ORR was 59%, including a CR rate of 14% and a partial response (PR) rate of 44%, and the median duration of response (DOR) was 12.2 months (ALIQOPA™, Prescribing Information, 2018). Warnings include infections, hyperglycemia, hypertension, non-infectious pneumonitis, neutropenia, and severe cutaneous reactions, among others. At present, copanlisib is not approved in the European Union (EU). Duvelisib received accelerated approval in US for adult patients with relapsed or refractory follicular lymphoma (FL) after at least 2 prior systemic therapies, based on ORR of 42% and 1 patient achieving a CR out of 83 patients (COPIKTRA® [Package Inert], 2018). However, only 43% and 17% of patients were maintaining response at 6 months and 12 months respectively. At present, duvelisib is not approved in the EU.

Thus, despite the initially indolent clinical courses of FL and responsiveness to chemo-immunotherapy, there remains a substantial unmet medical need among patients with relapsed or progressive disease.

[1] ZYDELIG® is a registered trademark of Gilead Sciences, Inc.

## 1.2. Diffuse Large B-Cell Lymphoma

Diffuse large B-cell lymphoma (DLBCL) is the most common subtype of NHL and accounts for approximately one-third of all newly diagnosed cases. Similar to FL, clinical risk factors have been identified to assess prognosis in patients with aggressive NHL using the International Prognostic Index (IPI) for aggressive NHL, and these were shown to retain prognostic discrimination in the era of anti-CD20 therapy in a Revised International Prognostic Index (IPI) (Sehn, 2007). Patients with 2 or more risk factors have a less than 50% chance of relapse-free survival and overall survival (OS) at 5 years. In addition, molecular markers have been identified that are known to confer a poor prognosis; these include the presence of chromosomal rearrangements involving the MYC and BCL-2 and/or BCL-6 coding regions, and other aggregated molecular genetic characteristics have also been shown to be prognostic (Schmitz, 2018) (Chapuy, 2018). For patients with newly diagnosed aggressive B-cell lymphoma such as DLBCL, chemoimmunotherapy with an anti-CD20 antibody and an anthracycline-based regimen (often R-CHOP) is standard for patients who are expected to tolerate such treatment.

### 1.2.1. Systemic Therapies for Relapsed or Refractory Diffuse Large B-Cell Lymphoma

Patients who have DLBCL that has relapsed or is refractory to systemic therapy may receive salvage therapy with platinum-based immune-chemotherapy regimens such as with rituximab, ifosfamide, carboplatin, etoposide (R-ICE) or rituximab, dexamethasone, high-dose cytarabine, cisplatin (R-DHAP), among others; if an adequate response is achieved, such patients may then receive consolidation with high dose systemic therapy and autologous stem cell transplantation (ASCT). The approval was based on a randomized study that showed improvement in the CR rate (40% vs 18%) and response duration in polatuzumab vedotin (P) in combination with BR compared to BR alone; among the 25 patients who achieved a response to P+BR, 16 (64%) had response durations of at least six months and 12 (48%) had response durations of at least 12 months. Patients who have chemotherapy-insensitive disease and/or are ineligible for ASCT have a particularly dismal prognosis, and in some series these patients had a median OS of only 4 months (Friedberg, 2011). In addition to the option for ASCT, 2 anti-CD19 chimeric antigen receptor T-cell (CAR-T) therapies, axicabtagene ciloleucel and tisagenlecleucel, received marketing approvals in the United States (US) and the European Union (EU) to treat adult patients with relapsed or refractory DLBCL.

In June 2019, polatuzumab vedotin in combination with bendamustine and rituximab (BR) received accelerated approval in the US for the treatment of adult patients with relapsed or refractory DLBCL after at least 2 prior therapies (POLIVY™ [Package Insert], 2019). Axicabtagene ciloleucel received Food and Drug Administration (FDA) approval in 2017 and EU approval in 2018 to treat adult patients with relapsed or refractory large B-cell lymphoma (including DLBCL) after 2 or more lines of systemic therapy; in this population the overall response rate (ORR) was 72% with a complete response (CR) rate of 51%. Among patients achieving CR, the estimated median DOR was not reached after a median follow-up of 7.9 months. However, the estimated median DOR among patients in PR was only 2.1 months. Grade 3 or higher cytokine release syndrome (CRS) was noted in 13% of patients, and grade 3 or higher neurologic toxicities occurred in 31% of patients (YESCARTA™ [Package Insert], 2017).

Tisagenlecleucel received FDA approval in 2018 to treat adult patients with relapsed or refractory large B-cell lymphoma (including DLBCL) after 2 or more lines of systemic therapy including,

and it received EU approval in 2018 for the treatment of adult patients with relapsed or refractory DLBCL after 2 or more lines of systemic therapy; the overall response rate (ORR) was 50% with a complete response (CR) rate of 32%. The median DOR was not reached among all patients; however, the estimated median DOR among patients who achieved a best response of PR was only 3.4 months. Grade 3 or higher cytokine release syndrome (CRS) was noted in 13% of patients, and grade 3 or higher neurologic toxicities occurred in 28% of patients. (KYMRIAH™ [Package Insert], 2017).

Nonetheless, the use of CAR-T therapy might not be feasible for patients with comorbidities or who are otherwise frail, and some patients have progressive disease during CART-cell manufacture that precludes administration of this therapy. Thus, there is a considerable unmet medical need for therapy in such patients.

## 1.3. Mantle Cell Lymphoma

Mantle cell lymphoma (MCL) comprises approximately 7% of B-NHL in the US and Europe. It is characterized in the majority of cases by a t(11;14) chromosomal translocation that results in dysregulation of cyclin D1 gene (*CCND1*) expression. The majority of patients have aggressive course at the time of initial diagnosis and are treated with chemoimmunotherapy consisting of rituximab, bendamustine (BR) or R-CHOP as initial therapy. The addition of rituximab to CHOP was shown to provide an ORR of 94% and CR rate of 34% (Lenz, 2005). The MCL International Prognostic Index (MIPI) is used as a prognostic stratification tool for patients with MCL. In a recent report, 5-year OS rates in MIPI low, intermediate, and high-risk groups were 83%, 63%, and 34%, respectively (Hoster, 2014).

### 1.3.1. Systemic Therapies for Relapsed or Refractory Mantle Cell Lymphoma

Two Bruton’s tyrosine kinase (BTK) inhibitors, ibrutinib and acalabrutinib, have received accelerated approval from the FDA for previously treated patients with MCL based on single arm studies. Ibrutinib is approved in the EU for the treatment of adult patients with relapsed or refractory MCL. In an open-label study of ibrutinib in previously treated patients with MCL, an ORR of 65.8% and CR rate of 17.1% was observed with a median DOR of 17.8 months (IMBRUVICA® [Package Insert], 2013). In the phase 2 study of acalabrutinib (Trial LY-004) in patients with mantle cell lymphoma who received at least one prior line of therapy, an ORR of 80% and CR rate of 40% was noted (CALQUENCE® [Package Insert], 2017). Despite encouraging overall response rates, patients have a very poor prognosis after ibrutinib failure. The median OS of patients after cessation of ibrutinib has been reported to be only 2.9 months (Martin, 2016). The median OS of patients not receiving post-ibrutinib treatment and those receiving subsequent therapy after ibrutinib failure was 0.8 months and 5.8 months respectively. There are no proven therapies that have shown benefit after ibrutinib failure, and there is an important need for therapies in relapsed or refractory MCL.

## 1.4. Marginal Zone Lymphoma

Marginal zone lymphoma (MZL) originates from memory B lymphocytes in the marginal zone of lymphoid follicles of the spleen, mucosal-associated lymphoid tissues, and lymph nodes. MZL accounts for 8%–12% of all B-NHLs. Based on the site of involvement and molecular characteristics, MZL is classified into 3 subtypes comprising extranodal marginal zone lymphoma

(EMZL), also called mucosa-associated lymphatic tissue (MALT) lymphoma; splenic MZL (SMZL); and nodal MZL (NMZL). The initial therapy for MZL differs greatly based upon the subtype and underlying etiology. MZL that is associated with a viral or bacterial etiology is treated with antiviral or antibacterial therapy as primary treatment. However, patients with advanced disease are often treated with chemoimmunotherapy, similar to that for other indolent lymphomas. In a prospective, randomized trial of BR versus R-CHOP in indolent lymphomas in first-line, the median PFS was significantly greater in the BR group than in the R-CHOP group (Rummel, 2013). In a single arm, phase 2 study of BR in MALT lymphoma, event-free survival at 2 years and 4 years was 93% and 88% respectively (Salar, 2014).

### 1.4.1. Systemic Therapies for Relapsed or Refractory Marginal Zone Lymphoma

Treatment for relapsed or treatment-refractory MZL is similar to that of other indolent B-NHL subtypes such FL. Various chemoimmunotherapy regimens have been used in relapsed/refractory MZL (Dreyling, 2013). In the phase 2 CHRONOS-1 study, copanlisib monotherapy in patients with relapsed/refractory MZL (n=23) demonstrated an ORR of 70% and CR rate of 9% (Dreyling, 2017). Ibrutinib received accelerated approval by US FDA in 2017 for treatment of relapsed/refractory MZL in patients who had at least 1 prior line of therapy based on a single arm, Phase II trial which demonstrated an ORR of 46.0% and a CR rate of 3.2%. At a median follow-up of 19.4 months, the median PFS was 14.2 months, and the median overall survival was not yet reached (IMBRUVICA® [Package Insert], 2013). However, the low CR rate and treatment failures present an unmet need for curative therapies in relapsed/refractory MZL.

## 1.5. Other B-cell non-Hodgkin Lymphomas

Survival in patients with other relapsed/ refractory B-NHL including follicular lymphoma grade 3b, B-cell lymphoblastic lymphoma, Burkitt lymphoma, primary mediastinal large B-cell lymphoma remain poor; and patients with such uncommon B-NHL subtypes will be included in this cohort. However, patients with Waldenström macroglobulinemia (WM, lymphoplasmacytic lymphoma) will be excluded from this study due to potential rheologic effects resulting from hyperviscosity due to release of monoclonal IgM during treatment. WM treatment with odronextamab may be evaluated in a separate investigation in the future. Patients with a current diagnosis of mixed histology of B-NHL will be allowed into the study.

## 1.6. Odronextamab Anti-CD20 x Anti-CD3 Bispecific Antibody

REGN1979 (odronextamab) is a human IgG4-based bispecific antibody that binds to CD20 and to CD3, a T cell antigen associated with the T-cell receptor complex. The anti-CD3 heavy chain possesses a 2-amino acid substitution in the CH3 domain, which abrogates binding to protein A. Consequently, when the anti-CD20 and anti-CD3 heavy chains are co-expressed, the resulting CD20 homodimer, CD20xCD3 heterodimer, and CD3 homodimer antibodies have relatively high, intermediate, or low avidity for Protein A, respectively. This differential binding makes it possible to selectively isolate the CD20xCD3 heterodimer (ie, odronextamab) by Protein A affinity chromatography.

## 1.7. Summary of In Vitro and In Vivo Pharmacology Studies

The primary mechanism of action of odronextamab is through directed T cell-mediated killing of CD20+ target cells. Odronextamab -bound Fcγ receptors with no or low affinity do not induce an

antibody-dependent cell-mediated cytotoxicity (ADCC), thus showing only weak complement-dependent cytotoxicity (CDC) activity.

In 2 mouse B-cell lymphoma models, odronextamab -targeting of Raji (B cell) tumors resulted in significant tumor growth suppression. In addition, in a single-dose monkey pharmacology study with rituximab and odronextamab administered as single-agents, odronextamab more effectively depleted B-cells deep within lymphoid tissues of normal monkeys than did rituximab (Smith, 2015).

Safety pharmacology studies were performed in a repeat-dose monkey toxicology study with odronextamab given intravenously (IV; 0.01, 0.1, or 1 mg/kg) once weekly (QW) over 4 weeks (total 5 administrations). Evaluation of cardiac conduction (electrocardiograms [ECG] and heart rate by jacketed external telemetry) and hemodynamics (blood pressure by tail cuff), neurologic function, and respiration rate showed no odronextamab-related effects. Moreover, there were no deleterious gross or microscopic changes observed in tissues associated with these systems.

## 1.8. Clinical Background and Current Study

In addition to this trial, there are 2 ongoing clinical studies with odronextamab.

**Study R1979-HM-1333** is a phase 1, first-in-human, open-label, multi-center, dose escalation study of single agent odronextamab administered as an IV infusion in patients with CD20+ B-cell malignancies (non-Hodgkin lymphoma [NHL] and chronic lymphocytic leukemia [CLL]) previously treated with anti-CD20 antibody therapy. The primary objective of the study is to assess the safety, tolerability, and dose-limiting toxicities (DLTs) of odronextamab. Updated safety and efficacy data for this study can be found in the odronextamab Investigator's Brochure.

**Study R1979-ONC-1504** is a phase 1, open-label, multicenter, dose-escalation study with multiple dose escalation and expansion arms. One of the study arms includes patients with indolent or aggressive B-NHL to receive odronextamab combined with cemiplimab (REGN2810), an anti-Programmed Death-1 (PD-1) monoclonal antibody drug that is currently approved in the US for treatment of advanced cutaneous squamous cell carcinoma. The primary objective of this part of the study is to assess safety, tolerability, and DLTs of the drug combination.

Collectively, information from pre-clinical and clinical studies indicates that odronextamab has an acceptable safety profile and promising single agent clinical activity in patients with relapsed or refractory B-NHL, including FL. Detailed information on safety and efficacy for odronextamab is available in the Investigator's Brochure.

Considering the non-clinical experience and clinical experience from the phase 1 single agent study, odronextamab presents a highly attractive opportunity for therapeutic intervention in patients with previously treated B-NHL with high unmet medical need.

# 2. STUDY OBJECTIVES

## 2.1. Primary Objective

The primary objective of this study is to assess the anti-tumor activity of single agent odronextamab as measured by the objective response rate (ORR) according to the Lugano

Classification of response in malignant lymphoma (Cheson, 2014) and as assessed by independent central review in each of the following B-cell non-Hodgkin lymphoma (B-NHL) subgroups:

- In patients with follicular lymphoma (FL) grade 1-3a that has relapsed after or is refractory to at least 2 prior lines of systemic therapy, including an anti-CD20 antibody and an alkylating agent.
- In patients with diffuse large B-cell lymphoma (DLBCL) that has relapsed after or is refractory to at least 2 prior lines of systemic therapy, including an anti-CD20 antibody and an alkylating agent.
- In patients with mantle cell lymphoma (MCL) that has relapsed after or is refractory to a BTK inhibitor. This cohort will also include patients who have relapsed or have disease refractory to prior systemic therapy, or patients who have demonstrated intolerance to BTK inhibitor therapy, and who have progressed after other systemic therapy.
- In patients with marginal zone lymphoma (MZL) that has relapsed after or is refractory to at least 2 prior lines of systemic therapy.
- In patients with other B-NHL subtypes that have relapsed after or are refractory to at least 2 prior lines of systemic therapy.

## 2.2. Secondary Objectives

The secondary objectives of this study are:

- To assess the anti-tumor activity of single agent odronextamab in each of 5 disease-specific cohorts, as measured by:
  - ORR according to the Lugano Classification and as assessed by local investigator evaluation.
  - Complete response (CR) rate according to the Lugano Classification and as assessed by local investigator evaluation and independent central review.
  - Progression free survival (PFS) according to Lugano Classification and as assessed by:
    - Independent central review, and
    - Local investigator evaluation
  - Overall survival (OS)
  - Duration of response (DOR) according to the Lugano Classification and as assessed by:
    - Independent central review, and
    - Local investigator evaluation

- Disease control rate (DCR) according to the Lugano Classification and as assessed by:
  - Independent central review, and
  - Local investigator evaluation

- To evaluate the safety and tolerability of odronextamab
- To assess the pharmacokinetics (PK) of odronextamab
- To assess the immunogenicity of odronextamab
- To assess the effect of odronextamab on patient reported outcomes, including health-related quality of life (HRQL), as measured by the validated instruments European Organisation for Research and Treatment of Cancer Quality of Life Questionnaire (EORTC QLQ-C30), Functional Assessment of Cancer Therapy-Lymphoma (FACT-Lym), and EuroQoL 5 Dimensions 3 Levels (EQ-5D-3L)

## 2.3. Exploratory Objectives

- To assess changes in serum C-reactive protein (CRP), ferritin, and cytokine levels in patients treated with odronextamab, and to assess the relationship between cytokine increases during study drug treatment, measures of efficacy, the incidence of adverse events (AEs), and relationship to tumor burden and location
- To characterize the rate of undetectable minimal residual disease (MRD) status over the course of the treatment
- To evaluate blood pharmacodynamic biomarkers potentially related to the mechanism of action of odronextamab, including the activation and distribution of peripheral blood mononuclear cell (PBMC) subsets
- Markers of T-cell activation over time
- Molecular and cellular profile of malignant lymph node tissue at baseline on treatment and at disease progression (eg, B cell CD20 expression, T cell subsets and activation markers, DNA and RNA sequence profile)
- To evaluate the relationship between PK and pharmacodynamic measures for relevant biomarkers and concentration-response (C-R) relationships for efficacy endpoints
- To assess the burden of hospitalization for study drug administration and safety events in patients treated with odronextamab

# 3. HYPOTHESIS AND RATIONALE

## 3.1. Hypothesis

In this study, each disease-specific cohort (FL grade 1-3a, DLBCL, MCL after BTK inhibitor therapy, MZL, and Other B-NHL [excluding WM, CLL, SLL, Burkitt lymphoma and Burkitt-like lymphoma with 11q aberration]) is evaluated separately, and no formal hypotheses will be tested.

## 3.2. Rationale

### 3.2.1. Rationale for Patient Population and Study Design

The first-in-human (FIH) study of odronextamab (R1979-HM-1333) includes patients with CD20-positive B-NHL relapsed after or refractory to CD20-directed antibody therapy. During the course of this study, efficacy has been observed in a broad range of B-NHL subtypes, including FL grade 1-3a, DLBCL without prior CAR-T therapy, DLBCL after failure of CD19-directed CAR-T therapy, mantle cell lymphoma (MCL), marginal zone lymphoma (MZL), and other B-cell malignancies that express CD20. An interim safety data review has shown that the identified risks for odronextamab include CRS and IRR events. However, the majority of events of CRS or IRR reported with odronextamab monotherapy were non-serious and were of mild to moderate severity; for CRS or IRR events, interventions such as temporary study treatment interruption, administration of anti-pyretic and/or corticosteroid therapy, or other supportive care were effective in limiting the severity and duration; a minority of cases prompted the use of tocilizumab (anti-IL-6R antibody). Guidelines for the identification and management of CRS/IRR are described in the protocol Section 7.3.3.

Overall, the safety and efficacy analyses suggest a favorable risk-benefit profile in each of these subtypes of B-NHL. Based on these promising clinical observations, the anti-tumor activity and safety of odronextamab will be evaluated in this present study in patients with a broad range of relapsed/refractory B-NHL in separate disease-specific cohorts. Additional information can be found in the odronextamab Investigator's Brochure.

This study will include 5 disease-specific cohorts, and the rationale for inclusion of each B-NHL patient population is as follows:

**FL grade 1-3a**

There are limited treatment options for patients with FL that has recurred after 2 or more lines of therapy or with disease that is refractory to anti-CD20 therapy administered as a single agent or in combination therapy. Idelalisib and copanlisib each were approved in the US under the accelerated pathway in the third-line setting (see Section 1.1.1); these PI3K inhibitors are associated with considerable toxicities, and the clinical benefits of these agents remain to be verified in the respective confirmatory trials. Outside of the US, the treatment options in this setting are even more limited. Idelalisib was approved in the EU, Canada, South Korea, and Australia. However, the phase 3 trials of idelalisib in combination with other cancer medicines were terminated due to increased deaths and serious adverse events (SAEs). Copanlisib has not been approved outside of the US.

In the R1979-HM-1333 study of odronextamab in patients with B-NHL relapsed after therapy containing an anti-CD20 antibody, this bispecific antibody has shown promising clinical activity,



VV-RIM-00192456-1.0 Approved - 11 Apr 2022 GMT-5:00

including that in patients with relapsed/refractory FL, and acceptable tolerability (see Section 1.8). As of 05 May 2019, in patients with FL grade 1-3a treated at doses ≥ 5mg of odronextamab (N=17), the efficacy evaluation has shown a best response of CR in 9 patients, PR in 5 patients, SD in 1 patient, and too early to evaluate in 2 patients. Additional details on efficacy are in Section 5.2.4 of the odronextamab Investigator's Brochure.

The overview provided in Section 1.1.1 outlines the typical clinical course of FL: the ORR for FL has improved with newer chemo-immunotherapy regimens, but disease relapse or transformation to a high-grade lymphoma is characteristic of the usual clinical course for this disease. Patients with relapsed/refractory FL grade 1-3a comprise a population with considerable unmet medical need for effective and tolerable therapies.

In this study, a separate disease-specific cohort will evaluate the efficacy and safety of odronextamab single agent therapy in patients with relapsed or refractory FL.

**DLBCL cohort**

Patients with relapsed or refractory DLBCL who have chemotherapy-insensitive disease or are deemed to be ineligible for ASCT have a dismal prognosis, with a median OS of only 4 months (Friedberg, 2011). Recently, 2 anti-CD19 chimeric antigen receptor T-cell therapies, axicabtagene ciloleucel and tisagenlecleucel, received marketing approvals to treat adult patients with relapsed or refractory DLBCL in third line. However, CAR T-cell therapy is not feasible in all circumstances due to patient co-morbidities or disease progression during the preparation of the therapy.

As of 05 May 2019, in patients with DLBCL treated at doses ≥5mg of odronextamab (N=33), the efficacy evaluation has shown a best response of CR in 7 patients, PR in 6 patients, SD in 10 patients, and too early to evaluate in 4 patients. In patients with DLBCL treated at doses 18-40 mg of odronextamab, the efficacy evaluation has shown an ORR of 54.5% (6 out of 11 patients) and a CR rate of 18.2% (2 out of 11 patients). In patients treated at the 80 mg dose, 3 out of 5 patients (60%) had a CR; 1 out of 4 patients treated at 160 mg with DLBCL had a CR. Additional information regarding the observed efficacy in this patient population is in Section 5.2.4 of the odronextamab Investigator's Brochure.

A separate disease-specific cohort for DLBCL will evaluate further the efficacy and safety of odronextamab monotherapy in patients with DLBCL.

**MCL after BTK inhibitor therapy**

Despite an ORR of 65.8% and a median DOR of 17.8 months with ibrutinib monotherapy, a significant portion of patients with MCL either do not respond or progress on ibrutinib therapy (Wang, 2015). The median OS of patients after cessation of ibrutinib is brief at 2.9 months (Martin, 2016). The median OS of patients not receiving post-ibrutinib treatment and those receiving subsequent therapy after ibrutinib failure was 0.8 months and 5.8 months, respectively. There are no proven salvage therapies that have shown benefit after BTK inhibitor failure and such patients have high unmet need at this time (Smith, 2016).

As of 05 May 2019, in patients with MCL treated at doses ≥5mg of odronextamab (N=3), the efficacy evaluation has shown a best response of CR in 2 patients and PR in 1 patient. One CR was noted in a patient with MCL with 8 prior lines of therapy and who had disease that was refractory to the BTK inhibitor ibrutinib. Updated safety and efficacy data for patients with



VV-RIM-00192456-1.0 Approved - 11 Apr 2022 GMT-5:00

relapsed or refractory MCL in the ongoing first-in-human study (R1979-HM-1333) can be found in the odronextamab Investigator's Brochure. In this present study, a separate disease-specific cohort for patients with MCL who have progressed after prior BTK inhibitor therapy will further evaluate the efficacy and safety of odronextamab monotherapy.

**MZL**

In a single arm, phase II study, ibrutinib demonstrated an ORR of 46.0% in patients with relapsed or refractory MZL who had at least 1 prior line of therapy. However, the CR rate was only 3.2%, and the median PFS was only 14.2 months with a median follow-up of 19.4 months (IMBRUVICA® [Package Insert], 2013). In this study, 5% of patients were noted to be refractory to ibrutinib, and 32% of patients experienced disease progression following an initial response (Epperla, 2019) (Noy, 2017). These data suggest an unmet need for effective salvage therapies for relapsed or treatment-refractory MZL.

As of 05 May 2019, in patients with MZL treated at doses ≥5mg of odronextamab (N=5), the efficacy evaluation has shown a best response of CR in 2 patients and PR in 1 patient. Updated safety and efficacy data for patients with relapsed or refractory MZL in the ongoing first-in-human study (R1979-HM-1333) can be found in the odronextamab Investigator's Brochure. In this present study, a separate disease-specific cohort for patients with MZL who have had at least 2 prior lines of systemic therapy will evaluate the efficacy and safety of odronextamab monotherapy.

**Other B-NHL**:

The prognosis for patients with other relapsed/ refractory B-NHL subtypes including follicular lymphoma grade 3b, B-cell lymphoblastic lymphoma, Burkitt lymphoma, and primary mediastinal large B-cell lymphoma, among others, is generally poor. In a retrospective analysis of patients with relapsed or refractory Burkitt and high-grade B-cell leukemia/lymphoma, the median OS was 2.8 months, with a 1-year OS rate of 11% (Short, 2017).

As of 05 May 2019, in patients with FL grade 3b, Undetermined, and Other B-NHL treated at all dose levels of odronextamab (n=4), a CR was noted in 1 patient (FL grade 3b), SD in 1 patient (FL unknown grade) and Unable to Evaluate in 2 patients at the time of data cut-off. Updated safety and efficacy data for patients with other B-NHL in the ongoing FIH study (R1979-HM-1333) can be found in the odronextamab Investigator's Brochure. To better understand the efficacy of odronextamab monotherapy in other relapsed or refractory B-NHL subtypes, a separate disease specific cohort will be included in this present study. Patients with Waldenström macroglobulinemia (WM, lymphoplasmacytic lymphoma) will be excluded from this study due to potential rheologic effects resulting from hyperviscosity due to monoclonal IgM. Such patients might be evaluated in a separate study of odronextamab in the future.

### 3.2.2. Rationale for Dose Selection

Selection of the odronextamab regimen in this phase 2 study was informed by the integrated safety, efficacy and PK information obtained from Study R1979-HM-1333, in which doses ranging from 0.03 mg to 320 mg have been evaluated in patients with B-NHL. Odronextamab was well tolerated up to 320 mg QW, the highest dose tested, and there were no DLTs observed in patients with B-NHL treated through the 320 mg cohort. Additional information on the odronextamab clinical safety are provided in the odronextamab Investigator's Brochure.

As observed in study R1979-HM-1333, odronextamab concentrations in serum increased in a dose-dependent manner. Based on the observed odronextamab concentrations in the 80 mg cohort in study R1979-HM-1333, the mean (SD) trough concentration odronextamab at week 12 (the last QW dose) and week 20 (predose of 5th 80 mg Q2W dose) was 35.2 (21.5) mg/L and 22.4 (21.5) mg/L, respectively (ie, Q2Wwk20/QWwk12=0.64). Since the data for 160 mg Q2W in week 20 was not available at present, the extrapolated value in week 20 of 160 mg dose was 68 mg/L (ie, 107 mg/L x 0.64) based on the observed data at week 12 of 160 mg QW (mean=107 mg/L). With a further linear extrapolation, a mean trough concentration by week 20 of 320 mg Q2W dose could be ~136 mg/L (ie, 68 mg/L x2), which is above the mean trough concentration of 160 mg QW in week 12. This provides the rationale for selecting 2 times the QW dose during the Q2W treatment period to maintain sufficient odronextamab exposures. Further details on PK are provided in the Investigator’s Brochure.

**Rationale for implementing changes to the step-up dosing regimen including, reduction in the dose at week 1 day 1 and addition of intermediate dose-1 (introduced in global amendment 4):**

As of 04 Nov 2020, the rate of grade ≥3 CRS was 9% in all patients with B-NHL treated with the 1/20 regimen (1/20 regimen includes an initial dose of 1 mg, an intermediate dose of 20 mg, followed by a full dose of >20 mg odronextamab) in studies R1979-HM-1333 and R1979-ONC-1625 combined. The rate of grade ≥3 CRS is highest during the intermediate dosing period followed by the initial dosing period of the step-up dosing with the 1/20 regimen (grade ≥ 3 CRS rate is 6.4% with the actual doses of >1 to 20 mg [intermediate dosing] period and 2.3% with the actual doses of ≤1 mg [initial dosing] period). During the initial dosing period, grade ≥3 CRS events occurred predominantly after the first split infusion when compared to the second infusion (1.9% versus 0.4%).

To mitigate the rate of grade ≥3 CRS during step-up dosing in patients with B-cell malignancies, the step-up dosing period has been extended from 3 weeks to 4 weeks and the dose at week 1 day 1 is being reduced from 0.5 mg to 0.2 mg (total dose at week 1 will be 0.7 mg [split as 0.2 mg/ 0.5 mg to be given on 2 consecutive days], and an additional intermediate dose 1 of 4 mg is being introduced which is to be given as split infusion (2 mg/ 2 mg) between the initial dose of 0.7 mg (0.2 mg/0.5 mg) and the intermediate dose 2 of 20 mg (10 mg/ 10 mg) based on the review of safety data and a quantitative systems pharmacology (QSP) model. The proposed step-up dosing regimen of 0.7 mg/4 mg/20 mg/160 mg from week 1 to week 4 was supported by predictions of IL-6 concentration-time profile simulations in weeks 1 to 4 with the QSP model. The prediction indicates IL-6 levels in weeks 2 to 3 could be significantly reduced with the proposed 4-week step-up regimen when compared to that with a 3-week step-up dosing regimen of 1/20/160 mg during weeks 1 to 3. Split dosing has attenuated the risk of CRS during the dose escalation portion of the FIH study, therefore, the additional intermediate dose (4 mg) will be given as split dose. In this modified dose regimen, patients will receive the first full QW dose as a single infusion at week 4. Based on sub analysis of best overall response rate in patients who received the full QW dose at week 3 versus those who received the full QW dose at week 4 or later in study R1979-HM-1333 as of 14 Oct 2020 data cut-off date, there were no differences observed in the efficacy in the overall B-NHL population or in the subtypes of lymphomas due to dose modifications leading to delayed time to reach full QW dose. These data support that the proposed modified dose regimen that has an additional intermediate dose is not anticipated to have an impact on efficacy.

Up to global protocol amendment 3, step-up dosing included an initial dose of 1 mg during week 1, an intermediate dose of 20 mg during week 2, and a full QW dose administered weekly from week 3 to week 12 followed by Q2W dosing. As of global protocol amendment 4, regimen tested in this phase 2 study will consist of an initial split dose of 0.7 mg [0.2/0.5 mg] during treatment week 1, an intermediate split dose -1 of 4 mg during treatment week 2, an intermediate split dose -2 of 20 mg during treatment week 3, and a full QW dose administered weekly from treatment weeks 4 to 12 followed by Q2W dosing. For each disease-specific cohort of patients treated at a QW full (nominal) dose, the Q2W dose is twice that of the QW dose. However, for DLBCL cohort Arm 2, the Q2W dose of 320 mg is the same as the QW dose. The rationale for the QW full dose selected for each disease-specific cohort are provided below.

**FL grade 1-3a and MZL cohorts:** Both FL grade 1-3a and MZL are indolent lymphomas, and therefore a single dose has been selected for these 2 cohorts based on the observed clinical efficacy in the R1979-HM-1333 study. As of 05 May 2019, in patients with FL grade 1-3a treated at doses of ≥5mg of odronextamab (N=17) in Study R1979-HM-1333, the efficacy evaluation has shown a best response of CR in 9 patients, PR in 5 patients, SD in 1 patient, and too early to evaluate in 2 patients. In patients with MZL treated at all dose levels of odronextamab (n=6), a CR was achieved in 2 patients (both patients treated at 80 mg odronextamab) and a PR was achieved in 2 patients (one patient treated at 4 mg and the other at 27 mg odronextamab). Although objective responses were observed at odronextamab doses between 5-320 mg in study R1979-HM-1333, only a limited number of patients have been treated at each dose level. Given the clonal cellular heterogeneity of lymphomas, and the tolerable safety profile at all dose levels tested, a QW full dose of 80 mg during QW dosing and 160 mg during Q2W dosing has been selected for testing in the FL grade 1-3a and MZL cohorts.

**MCL cohort and other B-NHL cohorts:** Patients in the MCL and the other B-NHL cohorts have aggressive lymphomas. In Study R1979-HM-1333, higher doses (80 mg and above) were required to achieve optimal response rates for aggressive lymphomas compared to those for indolent lymphomas. Given the limited number of patients tested in each dose-escalation cohort, the clonal cellular heterogeneity of aggressive lymphomas, and the presence of bulky disease in some patients with relapsed or refractory aggressive B-NHLs, a QW full dose of 160 mg during QW dosing and 320 mg during Q2W dosing has been selected for these 2 cohorts.

**DLBCL cohort:** In Study R1979-HM-1333, a constant odronextamab clearance was achieved at higher doses. This indicates that the odronextamab PK becomes linear when the tumor target is saturated. Based on clinical efficacy data (ORR and durability of responses) in the FIH study, R1979-HM-1333, in conjunction with doses at which linear or near-linear PK were achieved, the following RP2D regimen was selected for further testing in DLBCL: 160 mg QWx12 weeks followed by 320 mg Q2W.

To better understand the PK/pharmacodynamic measures, and exposure-response relationship, the sponsor plans to randomize patients to 2 different treatment arms as an initial step in this phase 2 study. In Arm 1, patients will receive a QW full dose of 160 mg during QW dosing and 320 mg during Q2W dosing. In Arm 2, patients will receive a QW full dose of 320 mg during QW dosing and continue 320 mg during Q2W dosing. After the initial step, enrollment will proceed at the selected dose of 160 mg during QW dosing and 320 mg during Q2W dosing to a maximum of 127 patients for that dose.

**Rationale for study drug administration as a single infusion at Week 3 for cohorts with a weekly QW full (nominal) dose of 80 mg or 160 mg (up to Global Amendment 3).**

During the dose-escalation portion in Study R1979-HM-1333, patients treated in DL17 (1/20/320 mg) received the first QW full dose of 320 mg at week 3 that was split over 2 separate infusions of 160 mg each on 2 consecutive days. At the time of this protocol amendment, this dose regimen was determined to be tolerable. This safety experience from study R1979-HM-1333 support administration of 160 mg odronextamab or less as a single infusion in week 3 for cohorts with a QW full dose of 80 mg or 160 mg, respectively. Because the single infusion of each dose has been determined to be safe during week 3, the dose regimen in this study is revised to allow this study drug administration as a single infusion during week 3 to improve patient convenience. However, if a patient experiences grade 3 CRS prior to week 3, then odronextamab will be administered as a split infusion over 2 days at week 3. Because there is no safety data for the 320 mg dose administered as a single infusion at week 3, the QW full dose of 320 mg in Arm 2 of the DLBCL cohort will continue to be administered as 2 separate infusions of 160 mg each at week 3.

**As of the global amendment 4, patients will receive the first full QW dose as single infusion at treatment week 4.**

At treatment week 4 (QW full dose) and beyond, odronextamab will be administered as a single infusion if a patient does not experience grade 3 CRS prior to week 4 (QW full dose) and has received full initial, intermediate-1 and intermediate-2 doses as split infusions. However, if a patient experiences grade 3 CRS with the initial dose or the intermediate dose-1 or intermediate dose-2, then the first odronextamab QW full dose will be administered as a split infusion over 2 days.

### 3.2.3. Rationale for Treatment Duration

Patients with relapsed or refractory B-NHL have limited treatment options (see Section 1). Achieving a complete response to anti-lymphoma therapy in B-NHL correlates with an improved 3-year PFS (Lee, 2011). Considering also the clonal cellular heterogeneity of B-lymphoid neoplasms and the potential for sub-clonal cell populations that require ongoing study treatment to achieve durable disease control, the treatment duration has been updated to continue treatment until the time of disease progression or other protocol-defined reason for treatment discontinuation. In addition, if a patient has demonstrated a CR and has shown a durable response for at least 9 months after the initial determination of CR, then the frequency of study drug administration at the assigned dose will be decreased from Q2W to once every 4 weeks (Q4W) intervals, based on local investigator evaluation. Patients must have received the assigned QW full dose at the Q2W dosing schedule for at least 3 preceding doses before switching from Q2W to Q4W dosing. In the setting of sustained CR, the residual tumor burden and resulting target-mediated drug clearance are expected to be substantially lower than at the start of study treatment, and thus the transition to less frequent odronextamab dosing is justified in these patients. In addition, the reduction in the frequency of infusions from Q2W to Q4W is expected to improve patient convenience.

**Rationale for revising the treatment duration in disease-specific expansion cohorts in Global Amendment 2**

To better characterize the treatment effect in patients demonstrating sustained CR, the dosing schedule has been standardized in this amendment. If a patient has demonstrated a CR and has



VV-RIM-00192456-1.0 Approved - 11 Apr 2022 GMT-5:00

shown a durable response for at least 9 months after the initial determination of CR, the frequency of study drug administration should be reduced at the assigned dose from Q2W to Q4W intervals, based on local investigator evaluation. Patients must have received the assigned QW full dose at the Q2W dosing schedule for at least 3 preceding doses before switching from Q2W to Q4W dosing.

**Rationale for updating recommendations for tocilizumab use and intensification of premedications (introduced in global amendment 4):**

Cytokine analysis from Study R1979-HM-1333 suggests patients experienced grade ≥2 CRS have elevated levels of IL-6. Timely intervention with anti-IL-6 therapy (tocilizumab) for elderly patients or patients with extensive comorbidities, adult patients with persistent (lasting >3 days) and refractory fever in the setting of grade 1 CRS and for managing fluid refractory hypotension, hypoxia, and organ toxicities in the setting of grade 2 CRS and for all patients with grade ≥3 CRS has been recommended in the CARTOX working group guidelines (Neelapu, 2018b) and the Society for Immunotherapy of Cancer (SITC) clinical practice guideline on immune effector cell-related adverse events (Maus, 2020). Patient's age and the extent of the comorbidities will be taken into consideration by the investigator in determining the need for earlier use of tocilizumab for any grade of CRS. Implementing additional corticosteroids (day before, on the day of, and day after treatment), antipyretic, and antihistamine as required premedications during the step-up dosing will further mitigate the risk of CRS.

**Rationale for earlier intervention with tocilizumab and enhanced corticosteroid pre- and post-medication if 2 patients experience grade ≥3 CRS with the modified step-up regimen (introduced in global amendment 4):**

The modified step-up dosing regimen and the intensified premedication measures are expected to mitigate the rate of grade ≥3 CRS in patients with B-NHL. Pausing rules are being implemented so that the limit for grade ≥3 CRS is not exceeded by pausing enrollment after a limited number of patients in each cohort are treated. In addition to these stopping rules, if - at any time - a second patient experiences a grade ≥3 CRS event in all cohorts combined, the dose of oral dexamethasone (or equivalent) before and after odronextamab dosing days will be increased to 20 mg, and earlier tocilizumab intervention will be implemented (ie, tocilizumab use for patients with grade 1 CRS of fever lasting >24 hours and for all patients with grade ≥2 CRS). Recent data from the CAR-T therapy studies in B-NHL and B-ALL indicate that earlier use of tocilizumab and steroids reduces the rate of severe CRS without diminution in efficacy or an increase in infections (Ahmed, 2020) (Caimi, 2020) (Topp, 2019) (Locke, 2017) (Gardner, 2019), (Oluwole, 2021). Safety data from Cohort 6 in ZUMA-1 study which included earlier use of tocilizumab (if no improvement after 24 hours of supportive care for grade 1 CRS) showed a decrease in grade ≥3 CRS rate from 13% to 0% with axicabtagene ciloleucel in refractory aggressive NHL (Oluwole, 2021). Earlier intervention with tocilizumab for persistent fever lasting >10 hours in patients with B-cell acute lymphoblastic leukemia was effective in decreasing the rate of severe CRS from 30% to 15% (Gardner, 2019). Therefore, earlier intervention with tocilizumab and increased corticosteroid pre- and post-medication are justified as additional measures to mitigate severe CRS should the changes in the step-up dosing regimen prove insufficient.

# 4. STUDY ENDPOINTS AND VARIABLES

## 4.1. Endpoints

### 4.1.1. Primary Endpoint

The primary endpoint of the study for each of the 5 disease-specific cohorts is as follows:

- ORR according to the Lugano Classification of response in malignant lymphoma (Cheson, 2014) and as assessed by independent central review. The ORR is assessed from the time of the first patient first dose until all patients have completed 52 week tumor assessment for FL grade 1-3a/MZL and 36 week tumor assessment for DLBCL/MCL/Other B-NHL or have withdrawn from the study.

### 4.1.2. Secondary Endpoints

The secondary endpoints of the study for each of the 5 disease-specific cohorts are:

- ORR according to the Lugano Classification and as assessed by local investigator evaluation. The ORR is assessed from the time of the first patient first dose until all patients have completed 52 week tumor assessment for FL/MZL and 36 week tumor assessment for DLBCL/MCL/Other B-NHL or have withdrawn from the study.
- Complete response (CR) rate according to the Lugano Classification and as assessed by local investigator evaluation and independent central review. The CR rate is assessed from the time of the first patient first dose until patients have completed 52 week tumor assessment for FL grade 1-3a/MZL and 36 week tumor assessment for DLBCL/MCL/Other B-NHL or have withdrawn from the study.
- PFS according to the Lugano Classification and as assessed by:
  - Independent central review, and
  - Local investigator evaluation

The PFS is assessed from the time of the first patient first dose until the end of the study.

- OS is assessed from the time of the first patient first dose until the end of the study.
- DOR according to the Lugano Classification and as assessed by:
  - Independent central review, and
  - Local investigator evaluation

DOR is assessed from the start of response in the first patient until the end of the study.

- DCR according to the Lugano Classification and as assessed by:
  - Independent central review, and
  - Local investigator evaluation

DCR is assessed from the time of the first patient first dose until all patients have completed 52 week tumor assessment for FL grade 1-3a/MZL and 36 week tumor assessment for DLBCL/MCL/Other B-NHL or have withdrawn from the study.

- Incidence and severity of TEAEs from the time of the first patient first dose until the end of the study.
- Pharmacokinetics: Concentration of odronextamab
- Immunogenicity: Incidence and titer of anti-drug antibodies (ADA) and incidence of neutralizing antibodies (NAb) to odronextamab over time
- Changes in scores of patient-reported outcomes, as measured by the validated instruments EORTC QLQ-C30, FACT-Lym, and EQ-5D-3L. Changes in patient-reported outcomes will be assessed from the time of the first patient first dose until the end of the study.

### 4.1.3. Exploratory Endpoints

The exploratory endpoints of the study for each of the 5 for all disease-specific cohorts are:

- Changes in CRP, ferritin, and cytokine levels in patients treated with odronextamab from the time of the first patient first dose until the end of the study.
- Rate of undetectable MRD from the time of the first patient first dose until the end of the study.
- Number and duration of hospitalizations (eg, general ward, ICU, ER visits) for the duration of the study for each patient.

## 4.2. Study Variables

### 4.2.1. Demographic and Baseline Characteristics

Baseline characteristics to be collected for each patient will include standard demography (eg, age, race, and gender, among other patient characteristics), disease characteristics, and general medical and oncologic disease history.

### 4.2.2. Pharmacokinetic Variables

The PK variables are odronextamab concentrations in serum and time. The sampling time points are provided in Table 11.

### 4.2.3. Immunogenicity Variables

The immunogenicity variables are ADA status, titer, neutralizing antibody (NAb) status, and time-point/visit. Samples in this study will be collected at the clinic visits specified in Table 10.

# 5. STUDY DESIGN

## 5.1. Study Description

This is a phase 2, open-label, multi-cohort, multi-center study of odronextamab administered as an IV infusion to patients with B-NHL that has relapsed or is refractory to prior systemic therapy. The study consists of 5 disease-specific cohorts, each with independent parallel enrollment. Cohort assignments will occur based on the patient's diagnosis and treatment history at the time of enrollment (Figure 1). Patients in the DLBCL cohort will be randomized 1:1 to either Arm 1 or Arm 2 in the initial step of this cohort with 2 different odronextamab dose regimens. After the initial step, enrollment will only continue in the 160 mg QW/ 320 mg Q2W arm until a total of 112 patients but can be increased up to 127 patients (including the randomized patients in the arm) are reached for that dose level.

**Figure 1: Study Schema**

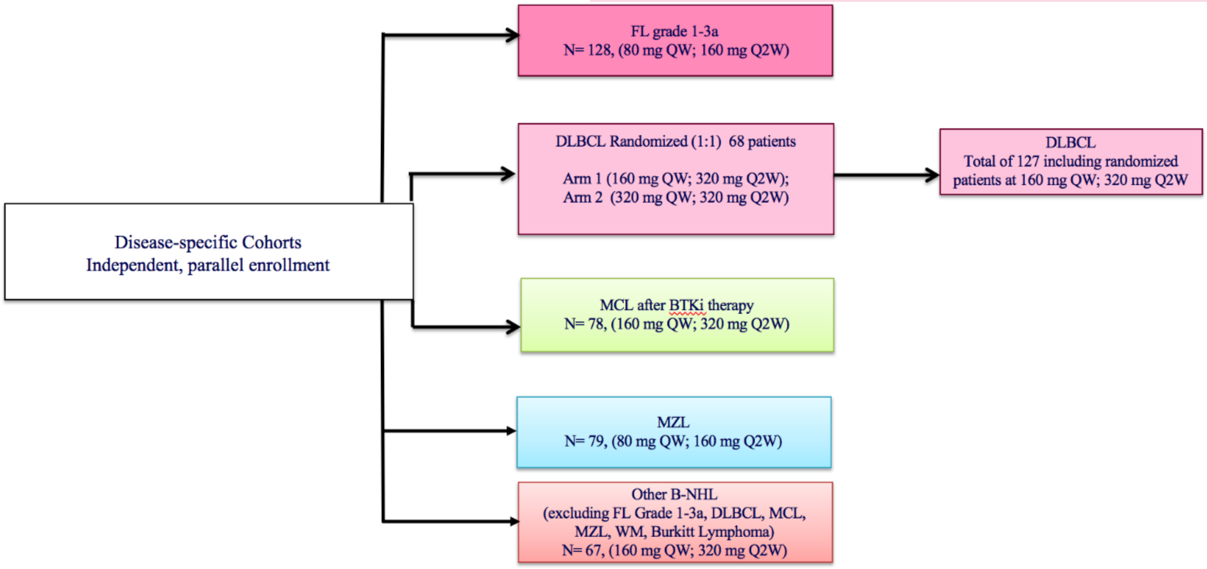


.

Note: New enrollment is paused for the MCL cohort as of global amendment 3 and for the MZL cohort as of global amendment 4 (See Section 6.2.1 for further details). MCL and MZL patients who have received and tolerated the initial dose, intermediate doses, and first QW dose will continue to receive treatment if they are experiencing clinical benefit as per investigator decision.

Enrollment into disease specific cohorts will be as shown below (Figure 2). For additional information on interim safety analysis please refer to Section 10.4.4.2.

1. Patients will be enrolled in FL grade 1-3a, DLBCL, and Other B-NHL cohorts simultaneously. The first CRS interim safety assessment will be performed in the first 25 patients (FL grade 1-3a and DLBCL/Other NHL combined) treated with the modified step-up regimen. Further enrollment in the study will be paused until the 25th patient has received the full QW dose or discontinued early.
2. If safety is cleared in the first CRS interim safety assessment, then parallel enrollment will resume. Enrollment will pause in the FL cohort once the 25$^{th}$ patient with FL grade 1-3a is enrolled and will pause in both the DLBCL and Other B-NHL cohorts once the 25$^{th}$ patient with DLBCL or other B-NHL is enrolled. For the purpose of CRS interim safety

assessment, the DLBCL and Other B-NHL cohorts will be combined (see Section 10.4.4.2).

3. If safety is cleared in the second CRS interim safety assessment, then enrollment will resume until a total of 60 patients are enrolled in each respective cohort (ie, FL grade 1-3a and combined DLBCL/Other B-NHL cohorts).
4. Patients enrolled in the first and second interim safety assessments will be counted towards the total of 25 and 60 patients enrolled in each cohort.
5. Enrollment is paused for patients with MZL and MCL until the modified step-up regimen has been evaluated in the other cohorts and will be reopened in a future amendment.
6. If stopping is triggered in one cohort (FL grade 1-3a or DLBCL/Other NHL), enrollment in all cohorts will be stopped until a revised regimen is implemented. If the modified step-up regimen in other odronextamab monotherapy studies is deemed not acceptable, then further enrollment in this study (FL grade 1-3a or DLBCL/Other NHL) will also be stopped until a revised regimen is implemented.
7. If at any time a second patient in FL grade 1-3a and DLBCL/Other B-NHL cohorts combined experiences a grade ≥3 CRS event, the dose of oral dexamethasone (or equivalent) before and after odronextamab dosing days will be increased to 20 mg, and earlier tocilizumab intervention will be implemented (ie, tocilizumab use for patients with grade 1 CRS of fever lasting >24 hours and for all patients with grade ≥2 CRS) for all ongoing and subsequently enrolled patients (See Section 7.3.3.2).

**Figure 2: Schema for CRS Interim Safety Assessments**

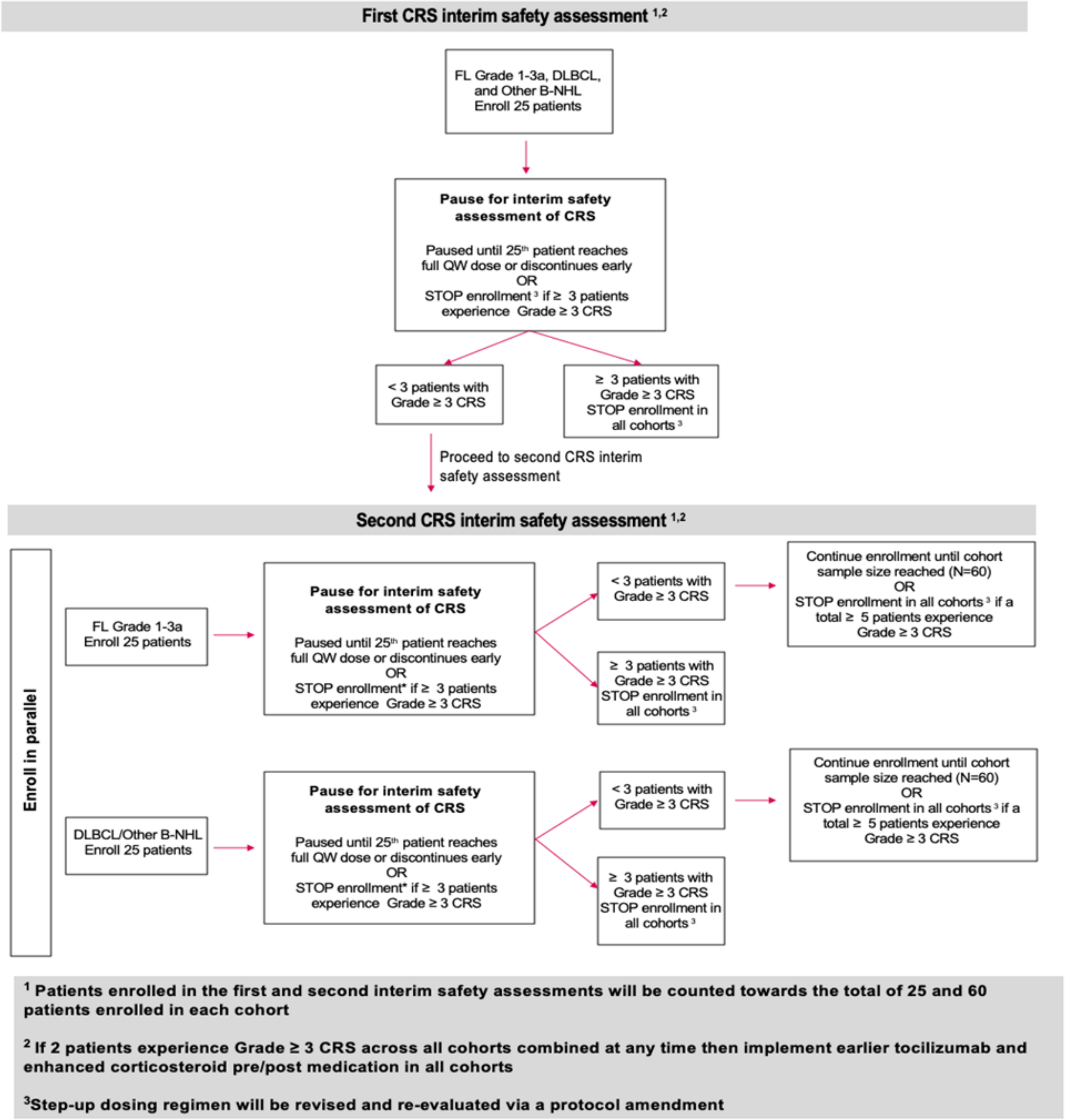


The treatment duration will comprise 12 QW doses followed by Q2W dosing until the time of disease progression or other protocol-defined reason for treatment discontinuation (Figure 3). In addition, if a patient has demonstrated a CR and has shown a durable response for at least 9 months after the initial determination of CR, then the frequency of study drug administration at the assigned dose will be decreased from Q2W to Q4W intervals, based on local investigator evaluation. Patients must have received the assigned full QW dose* at the Q2W dosing schedule for at least 3 preceding doses before switching from Q2W to Q4W dosing. Refer to Section 10.4.4.2 for safety pausing rules for transitioning from Q2W to Q4W odronextamab dosing. Patients will be followed for efficacy until the time of disease progression or start of non-protocol anti-lymphoma therapy. *NOTE: Patients who are receiving a lower dose than the

protocol-specified Q2W dose can switch to Q4W dosing after 9 months of maintained CR, after discussion and approval from Sponsor's Medical Monitor.

**Figure 3: Patient Study Flow Diagram**

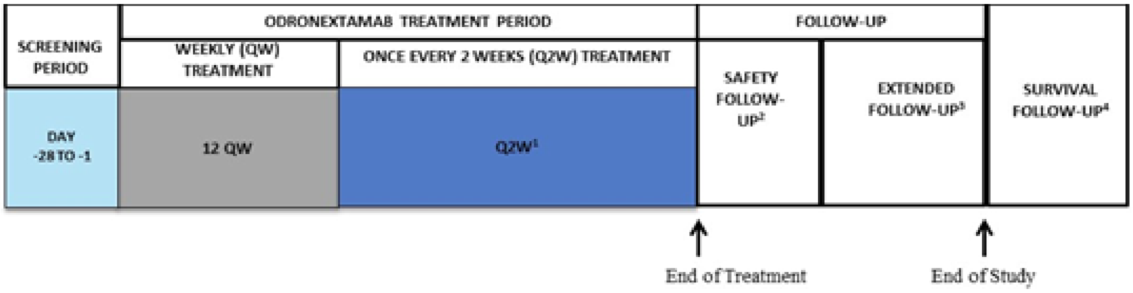


1. Study treatment to continue until the time of disease progression or other protocol-defined reason for treatment discontinuation. If a patient has demonstrated a CR and has shown a durable response for at least 9 months after the initial determination of CR, then the frequency of study drug administration at the assigned dose will be decreased from Q2W to Q4W intervals, based on local investigator evaluation.
2. **Safety follow-up:** The safety follow-up period consists of three Q4W Safety Follow-up Visits with safety follow-up visits at 4 weeks following the last dose (Safety Follow-up Visit 1), at 8 weeks following the last dose (Safety Follow-up Visit 2) and 12 weeks following the last dose (Safety Follow-up Visit 3)
3. **Extended follow-up**: Commences after the Safety Follow-up Visit 2 only for patients who have discontinued study drug for reasons other than disease progression, start of a non-protocol anti-lymphoma therapy, withdrawal of consent, or death. Disease response will be assessed until the time of disease progression, death, start of a non-protocol anti-lymphoma therapy, or patient withdrawal of consent for follow-up of disease status, whichever is sooner.
4. **Survival Follow-up:** Patients who discontinue from extended follow-up, but have not withdrawn consent will continue survival follow-up until death, loss to follow-up, withdrawal of consent or study termination by the sponsor, whichever is earlier. Patients may be followed remotely for survival information.

### 5.1.1. Study Conduct in Response to COVID-19

Recognizing that the "Coronavirus Disease 2019" (COVID-19) pandemic will have an impact on the conduct of clinical trials, the Sponsor does not intend to screen any patients in this study until the impact of the COVID-19 pandemic is deemed manageable and no longer interfering with the conduct of trials at individual sites, and patients can safely participate in this study. Until then, the Sponsor plans to obtain approvals from Health Authorities/Ethics Committees to enable initiation of study sites for this study, as allowed by local laws and regulations.

#### 5.1.1.1. COVID-19 Vaccination During the Study

It is recommended for patients to complete COVID-19 vaccination (initial series or booster) at least 1 week prior to receiving the first dose of odronextamab.

If a patient intends to receive COVID-19 vaccine (initial series or booster) after starting study treatment, it is recommended to delay COVID-19 vaccination until patients have received the initial gradual step-up dosing and have received and tolerated the recommended QW full dose of study treatment.

For more information regarding the permitted timing of COVID-19 vaccinations, see Section 7.6.2.

### 5.1.2. Screening Period

The screening period of up to 28 days duration begins with the signing of the informed consent form (ICF) and ends when the patient has been confirmed to be eligible for the study and has been assigned to the appropriate disease-specific cohort based on patient's diagnosis at the initiation of treatment, or with the determination that the patient is ineligible and has been designated as a screen failure. Patients may choose to participate in the optional genomics component of the study by signing the respective ICF.

After patients sign the main ICF, they will undergo screening assessments for eligibility, which are detailed in Section 8.1.

For patients who show laboratory abnormalities or clinical parameters that are clearly reversible, the screening interval may be extended by an additional 7 days to allow for resolution and retesting of the particular parameters that initially did not meet the study eligibility criteria. The additional 7-day interval for the screening period will also be allowed for imaging and central histopathology confirmation if this is required for administrative feasibility. Such 7-days extension may be granted only after discussion of the circumstances and rationale by the Investigator and the sponsor.

Rescreening is allowed for patients who failed screening at an earlier time point. Patients who fail screening may be screened one additional time, and an ICF will need to be signed at the re-screen. Some procedures may not need to be repeated if they were previously completed within 28 days (±7days) prior to first dose.

### 5.1.3. Treatment Period

Patients will be enrolled and begin treatment once they have demonstrated eligibility in the screening phase. Patients must start treatment within 2 days of being enrolled. The treatment period extends from the first dose of study drug until the last dose of study drug, or death, or loss to follow-up, whichever is sooner.

#### 5.1.3.1. Study Treatment

During the treatment period, patients will receive odronextamab with 12 QW treatments followed by Q2W treatments until the time of disease progression or other protocol-defined reason for treatment discontinuation. In addition, if a patient has demonstrated a CR and has shown a durable response for at least 9 months after the initial determination of CR, then the frequency of study drug administration at the assigned dose will be decreased from Q2W to Q4W intervals, based on local investigator evaluation. Patients who are receiving a lower dose than the protocol-specified Q2W dose can switch to Q4W dosing after 9 months of maintained CR, after discussion and approval from Sponsor's Medical Monitor. Patients will be followed for efficacy until the time of disease progression or start of or start of non-protocol anti-lymphoma therapy.

During the 12 QW treatment period, the first 4 weeks of treatment will comprise increasing step-up doses of odronextamab up to the dose of:

- FL grade 1-3a cohort: 80 mg QW followed by 160 mg Q2W
- DLBCL cohort Arm 1: 160 mg QW followed by 320 mg Q2W
- DLBCL cohort Arm 2: 320 mg QW followed by 320 mg Q2W



VV-RIM-00192456-1.0 Approved - 11 Apr 2022 GMT-5:00

- MCL after BTK inhibitor therapy: 160 mg QW followed by 320 mg Q2W
- MZL: 80 mg QW followed by 160 mg Q2W
- Other B-NHL: 160 mg QW followed by 320 mg Q2W

Enrollment in the DLBCL cohort Arm 2 is complete as of Global Amendment 3 and enrollment in the MZL and MCL cohorts is paused as of Global Amendment 3 and 4, respectively.

Each patient will receive odronextamab at an initial dose of 0.7 mg during treatment week 1 (administered as a split dose of 0.2 mg and 0.5 mg), followed by an intermediate dose-1 of 4 mg at treatment week 2, intermediate dose-2 of 20 mg at treatment week 3 and then the assigned QW full dose at treatment week 4 through 12 followed by Q2W dose. The dosing schema is depicted in Figure 4, and Figure 5 provided in Section 7.1. The initial dose (0.7 mg [0.2/0.5 mg]) and intermediate dose-1 of 4 mg and intermediate dose-2 of 20 mg are always administered as a split infusion over 2 days, preferably consecutive but no more than 3 days apart, even if these doses were delayed beyond treatment week 3. Administration of the QW full dose will only proceed if the full initial and full intermediate dose -1 and intermediate dose -2 were received and tolerated. [Note: Patients who received >90% of the intended dose are considered to have received the full dose]. For Arm 2 of the DLBCL cohort (320 mg QW dose), patients will receive the first QW full dose at week 4 as a split infusion administered over 2 days, with each split dose comprising a 160 mg infusion. For all other cohorts (80 mg or 160 mg QW dose) and for Arm 1 of the DLBCL cohort (160 mg QW dose). If a patient does not experience grade 3 CRS with the initial dose, the intermediate dose-1 and intermediate dose-2, then at treatment week 4 and beyond, QW full dose of odronextamab will be administered as a single infusion. However, if a patient experiences grade 3 CRS with the initial dose or the intermediate dose-1 or intermediate dose-2, the first odronextamab QW dose will be administered as a split infusion over 2 days.

For the initial treatment during week 1 up to and including the first full QW dose study treatment will be in an inpatient hospital setting. The inpatient hospital admission is required for approximately 24 hours (±4 hours) beyond the end of the second split infusion or the end of the single infusion. Inpatient hospital admission for study drug administration and observation at subsequent infusions may be implemented at the discretion of the investigator.

Additional details on safety monitoring of patients is located in Section 8.2.3.1.

Premedication is required prior to the start of odronextamab infusion for each split of initial dose, each split of intermediate dose-1, each split of intermediate dose-2, each split QW dose (if applicable), and the first administration of the QW full dose as a single infusion, as detailed in Section 7.2. If no IRR and/ or CRS of any grade are experienced following the first QW full dose administered as a single infusion, patients may initiate tapering of the dexamethasone premedication over subsequent administrations of odronextamab at the QW dose, as detailed in Section 7.2.

The Q2W odronextamab dose treatment will continue until the patient experiences disease progression or unacceptable toxicity, withdraws consent for treatment or from the study, or there is any other reason for study drug discontinuation (see Section 7.3.1).

Information on odronextamab resumption may be found in Section 5.1.4 .

**Figure 4: Odronextamab Treatment for FL grade 1-3a Cohort**

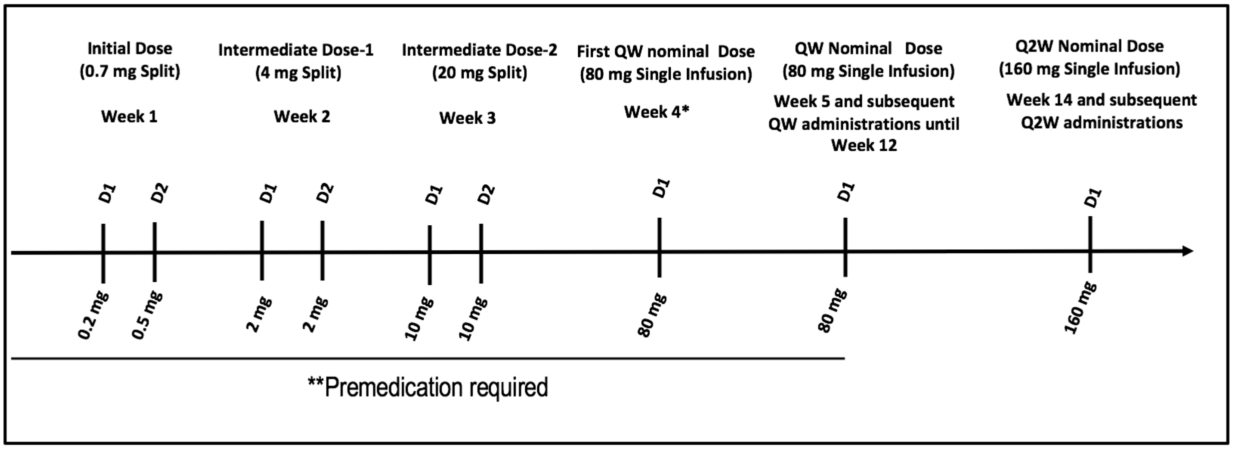


* If a patient does not experience grade 3 CRS prior to treatment week 4, at treatment week 4 and beyond, odronextamab will be administered as a single infusion. However, if a patient experiences grade 3 CRS prior to treatment week 4, then odronextamab will be administered as a split infusion over 2 days at treatment week 4.
** For premedication guidelines please refer to Section 7.2 .
Initial dose (0.7 mg administered as a split dose of 0.2 and 0.5 mg) and intermediate dose-1 (4 mg) and intermediate dose-2 (20 mg) are always administered as split infusions over 2 days, preferably consecutive but no more than 3 days apart, even if delayed beyond treatment week 3 (see Section 7.1). Proceed to QW full dose only if full initial (0.7 mg), intermediate dose 1 (4 mg) and intermediate dose 2 (20 mg) were received and tolerated. [Note: Patients who received >90% of the intended dose are considered to have received the full dose]
Study treatment will continue until the time of disease progression or other protocol-defined reason for treatment discontinuation. If a patient has demonstrated a CR (based on local investigator evaluation) and has shown a durable response for at least 9 months after the initial determination of CR, then the frequency of study drug administration at the assigned dose will be decreased from Q2W to Q4W intervals. Patients must have received the assigned full QW dose at the Q2W dosing schedule for at least 3 preceding doses before switching from Q2W to Q4W dosing.

**Figure 5: Odronextamab Treatment for DLBCL Cohort Arm 1 and Other B-NHL Cohort**

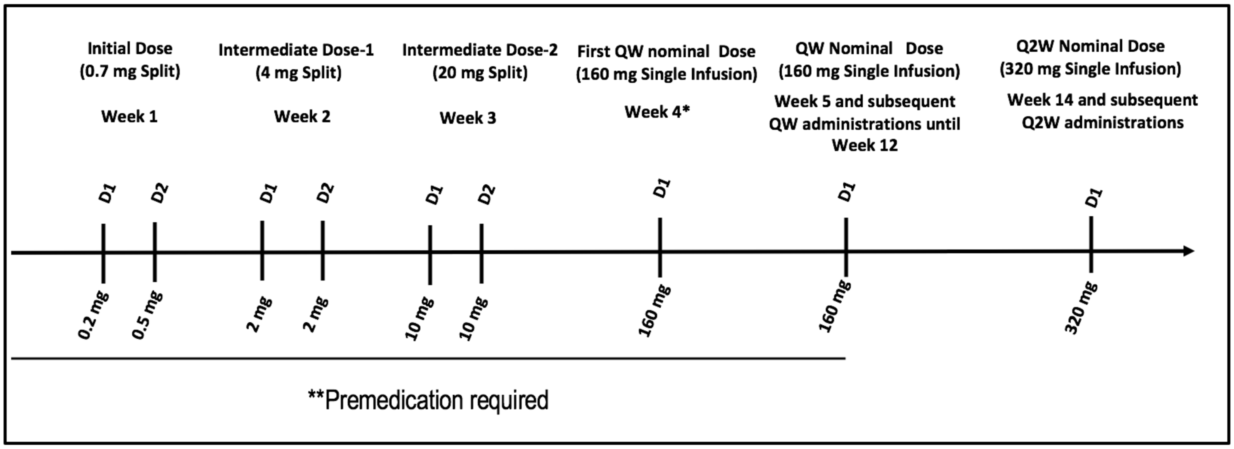


* If a patient does not experience grade 3 CRS prior to treatment week 4, at treatment week 4 and beyond, odronextamab will be administered as a single infusion. However, if a patient experiences grade 3 CRS prior to treatment week 4, then odronextamab will be administered as a split infusion over 2 days at treatment week 4.
** For premedication guidelines please refer to Section 7.2


VV-RIM-00192456-1.0 Approved - 11 Apr 2022 GMT-5:00

Initial dose (0.7 mg administered as a split dose of 0.2 and 0.5 mg) and intermediate dose-1 (4 mg) and intermediate dose-2 (20 mg) are always administered as split infusions over 2 days, preferably consecutive but no more than 3 days apart, even if delayed beyond treatment week 3 (see Section 7.1). Proceed to QW dose only if full initial (0.7 mg), intermediate dose 1 (4 mg) and intermediate dose-2 (20 mg) were received. [Note: Patients who received >90% of the intended dose are considered to have received the full dose].

Study treatment will continue until the time of disease progression or other protocol-defined reason for treatment discontinuation. If a patient has demonstrated a CR (based on local investigator evaluation) and has shown a durable response for at least 9 months after the initial determination of CR, then the frequency of study drug administration at the assigned dose will be decreased from Q2W to Q4W intervals,. Patients must have received the assigned full QW dose at the Q2W dosing schedule for at least 3 preceding doses before switching from Q2W to Q4W dosing.

#### 5.1.3.2. Treatment Delays

Patients who have dose interruptions or delays during the treatment period followed by resumption of treatment must complete the initial 12 QW doses prior to starting Q2W dosing. Thus, patients will receive a total of 12 QW doses prior to receiving Q2W dosing (refer to Section 7.3 for dose modification following treatment delay).

#### 5.1.3.3. Study Assessments

During the treatment period, safety will be assessed using routine clinical and laboratory safety assessments as described in Table 10. Treatment-emergent adverse events and concomitant medications will be monitored continuously. Radiologic assessments of disease will be performed according to Table 10 in Section 8.1. Quality of life assessments will be administered electronically using the EORTC-QLQ-C30, FACT-Lym and EQ5D-3L questionnaires, prior to any study procedures performed at a given study visit.

Patients will provide blood samples for PK and immunogenicity assessments and for research evaluation of biomarkers. A lymph node and/or tumor biopsy will be performed prior to treatment and is encouraged at week 6 (±7 days) or after patient receives 2 full doses (whichever is later) and at the time of tumor progression if in the opinion of the investigator the patient has an accessible lesion and the sample can be obtained without significant risk to the patient. A bone marrow aspirate and/or bone marrow biopsy will be performed at screening and, if positive for lymphoma infiltration, will be repeated at week 12 (12 weeks from commencing treatment) per calendar. If the bone marrow is positive for lymphoma infiltration at week 12, this assessment will be repeated subsequently at the time of suspected CR and at the time of tumor progression (if relevant). Tumor imaging will be performed on schedule even if there is interruption in treatment; if there are dosing delays then the imaging assessments might not align with study treatment schedule.

#### 5.1.3.4. Treatment in the Setting of Suspected Lymphoma Pseudo-Progression

During study treatment, patients might have a tumor assessment scan that is consistent with pseudo-progression based on the LYRIC criteria (Cheson, 2016) as outlined below and in Appendix 3:

1. Indeterminate response (IR1): ≥50% increase in the sum of the perpendicular diameters (SPD) of target lesions in first 12 weeks following start of study treatment
2. IR2: <50% increase in SPD of target lesions, with
    a. New lesion(s), or

   b. ≥50% increase in the product of the perpendicular diameters (PPD) of a lesion or set of lesions at any time during treatment

3. IR3: Increase in FDG uptake by PET-CT without a concomitant increase in lesion size meeting criteria for progressive disease (PD)

Patients who are suspected to have pseudo-progression of disease and are adequately tolerating odronextamab may be considered for continuation of study treatment until repeat imaging is obtained (8 to 12 weeks) that clarifies the response assessment. Prior to continuing study therapy after suspected pseudo-progression according to the LYRIC criteria (Cheson, 2016), the Investigator must discuss the relevant clinical and radiographic findings with the Sponsor and receive approval in writing to continue study treatment. If the collective response evaluation that incorporates this subsequent imaging or other disease assessments indicates definitive evidence of disease progression rather than pseudo-progression, then the patient must discontinue study treatment.

#### 5.1.3.5. Follow-up Period

The post-treatment follow-up period will begin after the last dose of study treatment.

##### 5.1.3.5.1. Safety Follow-Up

All patients will be followed for safety Q4W until 12 weeks following the last dose. The safety follow-up period consists of three Q4W Safety Follow-up Visits: 4 weeks following last dose (Safety Follow-up Visit 1), at 8 weeks following the last dose (Safety Follow-up Visit 2) and at 12 weeks following the last dose (Safety Follow-up Visit 3). Safety follow-up continues until all 3 visits are completed, or until start of a non-protocol anti-lymphoma therapy, or patient withdrawal of consent, whichever is sooner. See Section 9.4 regarding recording and reporting adverse events for more information. Patients will undergo tumor response, safety assessments, and quality of life assessments according to Table 10.

##### 5.1.3.5.2. Extended Follow-Up:

The extended follow-up is for patients who have discontinued study drug for any reason other than disease progression, start of non-protocol anti-lymphoma therapy, withdrawal of consent, or death. Disease response will be assessed until the time of disease progression, death, start of a non-protocol anti-lymphoma therapy, or patient withdrawal of consent for follow-up of disease status, whichever is sooner. For patients who discontinue from the study earlier than 12 weeks following the last dose (Safety Follow-up Visit 3), all assessments described in Table 10 for Safety Follow-up Visit 3 should be performed prior to study discontinuation.

##### 5.1.3.5.3. Survival Follow-Up:

After the safety follow-up period and, if applicable, the extended follow-up period, all study patients will be followed at Q12W intervals for survival until the time of death, loss to follow-up, patient withdrawal of consent for follow-up, or study termination by the sponsor, whichever is earlier. Survival follow-up status may be determined at clinic visits or remotely by the study site (such as by telephone). Survival monitoring is conducted as described in Section 8.2.6.

### 5.1.4. Resumption of Treatment

For patients who were newly enrolled or continued on study treatment at the time of Amendment 1 Global, re-treatment is not applicable. For patients who were off treatment at the time when Amendment 1 Global went into effect, resumption of treatment may be considered as described below. All decisions for resumption of treatment will be made after discussion between the treating physician and the sponsor's medical monitor.

Patients who experience disease recurrence may be considered for resumption of treatment if all of the following conditions are met:

- Have documented disease recurrence
- Initially showed clinical benefit from odronextamab (defined as a best response of CR or PR) during the treatment period
- May not have received intervening systemic anti-lymphoma therapy
- Adequately tolerated study treatment during the treatment period
- Continue to meet inclusion criteria 5, 6, 7, and 8 (Section 6.2.1) and all exclusion criteria (Section 6.2.2), except for study treatment (odronextamab) for exclusion criterion 2.
- The reason for discontinuation of initial treatment was not due to an AE related to odronextamab.

Prior to resuming treatment, patients will be required to re-sign the informed consent form and meet eligibility criteria for resuming treatment. Patients who are eligible to resume treatment will receive QW treatment doses starting with the initial dose, intermediate dose-1, intermediate dose-2 and full QW dose for 12 weeks followed by Q2W doses according to the treatment schedule described in Section 7.1. Such patients will be evaluated clinically for safety according to the schedule of events (Table 10). Disease evaluations may be scheduled as clinically indicated according to the treating physician. Research testing is optional in this setting and will be discussed in advance with the sponsor if collected.

### 5.1.5. End of Study Definition

The study and all follow-up will end when all study patients have been discontinued from the study or at the time of study closure by the sponsor, whichever is earlier. The end of study for an individual patient will be at the time when the patient discontinues from the study up to the end of the extended follow-up and prior to survival follow-up. All patients will continue survival follow-up until death, loss to follow-up, withdrawal of consent for follow-up, or study termination by the sponsor, whichever is earlier.

## 5.2. Planned Interim Analysis

An interim efficacy analysis will be conducted when 80 patients with FL grade 1-3a have completed 52-week assessment and 127 patients in the DLBCL cohort have completed 36-week assessment or have withdrawn from the study, whichever is later. The ORR and associated 95% confidence interval will be summarized for FL grade 1-3a cohort. As the primary objective of this interim efficacy analysis is point estimation on ORR and characterization of the precision of point

estimation, the study will not be stopped for perceived efficacy. For other efficacy endpoints, 2-sided 95% confidence interval will also be presented.

The sponsor will perform an administrative review for efficacy after at least 70 patients have been treated with the 0.7/4/20 mg step-up regimen and have completed a 4-week safety review. At the administrative review, summary of safety for all cohorts and summary of efficacy for the FL grade 1-3a and DLBCL cohorts will be performed.

An interim futility analysis for each of the MCL and MZL, disease-specific cohorts will be performed.

The details of the interim analysis are specified in Section 10.5.

# 6. SELECTION, WITHDRAWAL, AND REPLACEMENT OF PATIENTS

## 6.1. Number of Patients Planned

In the main global study, approximately a total of 512 patients are planned for enrollment and treatment across 5 disease-specific cohorts at multiple sites within US, Canada, Europe, and Asia-Pacific regions. As of global amendment 4, total sample size has been adjusted to allow enrollment with the modified step-up dosing regimen in different disease-specific cohorts as follows:

- 128 patients with FL grade 1-3a [Up to 60 patients with FL grade 1-3a will be enrolled with the modified step-up dosing regimen]
- 161 patients with DLBCL (68 randomized patients in Arm 1 and Arm 2, and a total of 112 patients (approximately 127 patients) including the randomized patients in the 160 mg QW/320 mg Q2W dose regimen) [The enrollment in this cohort will continue beyond 112 patients in order to have at least 60 patients of DLBCL treated with the modified step-up regimen]
- 67 patients with other B-NHL subtypes [Up to 53 patients with Other B-NHL subtypes will be enrolled with the modified step-up dosing regimen]

Applicable for Japan: Enrollment in Japan will include approximately 12 patients each, in the FL grade 1-3a cohort and the DLBCL cohort at the recommended dose. Any remaining patients in Japan exceeding the global enrollment for the FL grade 1-3a and DLBCL cohorts will be enrolled into a Japan extension for these cohorts.

Applicable for China: Enrollment in China will include approximately 20 patients in the FL grade 1-3a cohort and 26 patients in the DLBCL cohort at the recommended dose. Any remaining patients in China exceeding the global enrollment for the FL grade 1-3a and DLBCL cohorts will be enrolled into a China extension for these cohorts.

The following cohorts are not enrolling patients as of global amendment 4 and 3, respectively:

- 78 patients with MZL (not enrolling)
- 78 patients with MCL after failure of BTK inhibitor therapy (not enrolling)

In addition, if the modified step-up regimen is not deemed acceptable in either FL grade 1-3a or DLBCL/Other B-NHL cohorts per interim safety assessment based on grade ≥3 CRS, enrollment in all cohorts will be stopped until a revised regimen is implemented. If the modified step-up regimen in other odronextamab monotherapy studies is deemed not acceptable, then further enrollment into this study (FL and DLBCL/Other NHL) will be stopped until a revised regimen is implemented.

## 6.2. Study Population

The study population for each of the 5 disease-specific cohorts will consist of:

- FL grade 1-3a that has relapsed after or is refractory to at least 2 prior lines.  of systemic therapy, including an anti-CD20 antibody and an alkylating agent
- DLBCL that has relapsed after or is refractory to at least 2 prior lines of systemic therapy, including an anti-CD20 antibody and an alkylating agent
- MCL after BTK inhibitor therapy
- MZL that has relapsed or is refractory to at least 2 prior lines of systemic therapy
- Other B-NHL (excluding WM, CLL, SLL, Burkitt lymphoma and Burkitt-like lymphoma with 11q aberration) that has relapsed or is refractory to at least 2 prior lines of systemic therapy
  - Patients with mixed histology B-NHL with an aggressive component (such as concurrent FL and DLBCL)

### 6.2.1. Inclusion Criteria

Each patient must meet the following criteria to be eligible for inclusion in the study:

1. Age 18 years or greater
2. **For the FL grade 1-3a cohort only:** Central histopathologic confirmation of the FL grade 1 to 3a diagnosis must be obtained before study enrollment.  Patients with FL grade 3b are ineligible for this cohort but may be included in the “Other B-NHL” cohort. Follicular lymphoma  subtyping is based on the World Health Organization (WHO) classification (Swerdlow, 2017).
3. Disease-specific cohorts:

   Patients should, in the judgment of the investigator, require systemic therapy for lymphoma at the time of study enrollment and should be deemed not appropriate for any other approved therapy with established benefit for that indication. Refractory is defined as no response (SD/PD) or relapse within ≤6 months of last treatment.

   - **FL grade 1-3a cohort:** patients with FL grade 1-3a that has relapsed after or is refractory to at least 2 prior lines of systemic therapy, including an anti-CD20 antibody and an alkylating agent; patients must have failed combination lenalidomide and rituximab treatment where approved or deemed not appropriate to receive this treatment according to the investigator.



VV-RIM-00192456-1.0 Approved - 11 Apr 2022 GMT-5:00

- **DLBCL cohort:** patients with DLBCL that has relapsed after or is refractory to at least 2 prior lines of systemic therapy, including an anti-CD20 antibody and an alkylating agent. Patients with de novo DLBCL or DLBCL that is transformed from a lower grade neoplasm (eg, FL or CLL) may be enrolled. Patients with DLBCL transformation from prior CLL can only be enrolled in the absence of a leukemic CLL component. For patients with transformed DLBCL, prior systemic therapies administered for the lower grade neoplasm will not be considered among the prior lines of therapy for the purpose of determining eligibility.

  The following subtypes based on the WHO classification (Beham-Schmid, 2017) are eligible:

  – DLBCL not otherwise specified (NOS)

    – Germinal center B-cell type

    – Activated B-cell type

- **MCL after BTK inhibitor therapy cohort:** As of Global Amendment 3, new enrollment is paused for patients with MCL until further risk mitigation measures are put in place for this patient population.

- **MZL cohort:** patients with MZL that has relapsed or is refractory to at least 2 prior lines of systemic therapy. As of Global Amendment 4, new enrollment is paused for patients with MZL until safety of the modified step-up regimen has been optimized in other subtypes. Please refer to Section 10.4.4.2 for more information.

  The following subtypes based on the WHO classification (Beham-Schmid, 2017) are eligible:

  – Extranodal MZL of mucosa-associated lymphoid tissue (MALT lymphoma)

  – Nodal marginal zone lymphoma

  – Splenic marginal zone lymphoma

- **Other B-NHL cohort:** patients with B-NHL other than FL grade 1-3a, DLBCL, MCL, or MZL) that has relapsed or is refractory to at least 2 prior lines of systemic therapy.

  The following subtypes based on the WHO classification (Beham-Schmid, 2017) are eligible:

  – Primary mediastinal (thymic) large B-cell lymphoma

  – T-cell/histiocyte-rich large B-cell lymphoma

  – Epstein-Barr virus (EBV)+ DLBCL, NOS

  – High-grade B-cell lymphoma, with MYC and BCL2 and/or BCL6 rearrangements

  – High-grade B-cell lymphoma, NOS

  – B-cell lymphoma, unclassifiable, with features intermediate between DLBCL and classical Hodgkin lymphoma

- Follicular lymphoma, grade 3b

Note:

- Patients with a current diagnosis of mixed histology B-NHL with an aggressive component (such as, concurrent FL and DLBCL) are included in Other B-NHL cohort.
- Patients with a current diagnosis of transformed DLBCL, that have relapsed or are refractory to at least 2 prior lines of systemic therapy administered for lower grade neoplasm which are considered as standard regimens in aggressive lymphoma, will be included in the Other B-NHL cohort.
- Patients with Waldenström macroglobulinemia (WM, lymphoplasmacytic lymphoma), small lymphocytic leukemia (SLL) and chronic lymphocytic leukemia (CLL), Burkitt lymphoma and Burkitt-like lymphoma with 11q aberration are excluded.

4. Measurable disease on cross sectional imaging (defined as at least 1 bi-dimensionally measurable nodal lesion of ≥1.5 cm in the greatest transverse diameter (GTD) regardless of the short axis diameter) documented by diagnostic imaging (computed tomography [CT], or magnetic resonance imaging [MRI]).
5. Eastern Cooperative Oncology Group (ECOG) performance status 0 or 1.
6. Adequate bone marrow function as documented by:
   a. Platelet count ≥50 x $10^9$/L. A patient may not have received platelet transfusion therapy within 7 days prior to first dose of odronextamab in order to meet the platelet eligibility criterion.
   b. Hemoglobin ≥9.0 g/dL
   c. Absolute neutrophil count (ANC) ≥1.0 x $10^9$/L. A patient may not have received granulocyte colony stimulating factor within 2 days prior to first dose of odronextamab in order to meet the ANC eligibility criterion.
      - Patients with bone marrow involvement or splenic sequestration should meet the following hematologic parameters:
        - Platelet count ≥25 x $10^9$/L. A patient may not have received platelet transfusion therapy within 3 days prior to first dose of odronextamab in order to meet the platelet eligibility criterion.
        - Hemoglobin ≥7.0 g/dL
        - Absolute neutrophil count (ANC) ≥ 0.5 x $10^9$/L. A patient may not have received granulocyte colony stimulating factor within 2 days prior to first dose of odronextamab in order to meet the ANC eligibility criterion.
7. Adequate hepatic function:
   a. Total bilirubin ≤1.5 × upper limit of normal (ULN) (≤3 × ULN if attributed to lymphoma infiltration of liver)
   b. Alanine aminotransferase (ALT) and aspartate aminotransferase (AST) ≤2.5 × ULN (≤5 × ULN if attributed to lymphoma infiltration of liver)

c. Alkaline phosphatase (ALP) ≤2.5 × ULN (≤5 × ULN if attributed to lymphoma infiltration of liver)

**NOTES:**

∗ Irrespective of the presence of lymphoma infiltration of the liver, a patient with an AST >2.5 × ULN and/or ALT >2.5 x ULN concurrent with a total bilirubin >1.5 x ULN will be excluded.

∗ Patients with known Gilbert syndrome will be excluded if the total bilirubin value is >4 x upper limit of normal (ULN) for the local general population.

8. Serum creatinine ≤1.5 × ULN, or calculated creatinine clearance by Cockcroft-Gault formula ≥50 mL/min.

   **NOTE:** Patients with a calculated creatinine clearance <50 mL/min may be considered for enrollment if a measured creatinine clearance (based on 24-hour urine collection or other reliable method) is ≥50 mL/min.

9. Willingness to undergo tumor biopsy at baseline. If an investigator has determined that a baseline tumor biopsy cannot be obtained safely, the sponsor may grant an exception to the requirement for biopsy only after discussion with and approval by the medical monitor.
10. Ability to understand the purpose and risks of the study and provide signed and dated informed consent and authorization to use protected health information (in accordance with national and local subject privacy regulations).
11. Willing and able to comply with clinic visits and study-related procedures
12. Provide informed consent signed by study patient or legally acceptable representative
13. Able to understand and complete study-related questionnaires

### 6.2.2. Exclusion Criteria

A patient who meets any of the following criteria will be excluded from the study:

1. Primary central nervous system (CNS) lymphoma or known involvement by non-primary CNS NHL (suspected CNS lymphoma should be evaluated by lumbar puncture, as appropriate, in addition to the mandatory head CT or MRI).
2. Treatment with any systemic anti-lymphoma therapy within 5 half-lives or within 28 days prior to first administration of study drug, whichever is shorter.
3. History of allogeneic stem cell transplantation.
4. Prior treatment with any chimeric antigen receptor T-cell (CAR-T) therapy.
5. Continuous systemic corticosteroid treatment with more than 10 mg per day of prednisone or anti-inflammatory equivalent within 72 hours of start of study drug.
6. History of neurodegenerative condition or CNS movement disorder. Patients with a history seizure within 12 months prior to study enrollment are excluded.
7. Vaccination within 28 days prior to first study drug administration with a vector that has replicative potential.

8. Another malignancy except B-NHL in the past 5 years, with the exception of non-melanoma skin cancer that has undergone potentially curative therapy or in situ cervical carcinoma, or any other tumor that has been deemed to be effectively treated with definitive local control and with curative intent.
9. Evidence of significant concurrent disease or medical condition that could interfere with the conduct of the study or put the patient at significant risk, including but not limited to significant cardiovascular disease (eg, New York Heart Association Class III or IV cardiac disease, myocardial infarction within the previous 6 months, unstable arrhythmias, or unstable angina) and/or significant pulmonary disease (eg, obstructive pulmonary disease and history of symptomatic bronchospasm).
10. Cardiac ejection fraction <40% by echocardiogram or multigated acquisition (MUGA) scan.
11. Any infection requiring hospitalization or treatment with IV anti-infectives within 2 weeks of first administration of study drug.
12. Uncontrolled infection with human immunodeficiency virus (HIV), hepatitis B or hepatitis C infection; or other uncontrolled infection.
    a. Patients with HIV who have controlled infection (undetectable viral load and CD4 count above 350 cells/microliter either spontaneously or on a stable antiviral regimen) are permitted.
    b. Patients with hepatitis B (HepBsAg+) who have controlled infection (serum hepatitis B virus DNA polymerase chain reaction [PCR] that is below the limit of detection AND receiving anti-viral therapy for hepatitis B) are permitted.
    c. Patients who are hepatitis C virus antibody positive (HCV Ab +) who have controlled infection (undetectable HCV RNA by PCR either spontaneously or in response to a successful prior course of anti-HCV therapy) are permitted.
    d. Cytomegalovirus (CMV) infection as noted by detectable levels on a blood PCR assay. Patients who show detectable levels of CMV at screening will need to be treated with appropriate antiviral therapy and demonstrate at least 2 undetectable levels of CMV by PCR assay (at least 7 days apart) before being reconsidered for eligibility.
13. History of severe allergic reaction attributed to compounds with a similar chemical or biologic composition as that of the study drug or excipient. A severe allergic reaction is defined for this purpose as that requiring hospitalization and/or treatment with epinephrine.
14. Known hypersensitivity to both allopurinol and rasburicase
15. Member of the clinical site study team or his/her immediate family, unless prior approval granted by the sponsor.
16. Women of childbearing potential (WOCBP) with a positive serum β-hCG pregnancy test are ineligible for this study.
17. Patients who are committed to an institution by virtue of an order issued either by the judicial or the administrative authorities.

18. Pregnant or breastfeeding women.
19. Women of childbearing potential* or men who are unwilling to practice highly effective contraception prior to the initial dose/start of the first treatment, during the study, and for at least 6 months after the last dose. Sperm donation is prohibited during the study and for 6 months after the last dose of study drug. Highly effective contraceptive measures include:

    a. stable use of combined (estrogen and progestogen containing) hormonal contraception (oral, intravaginal, transdermal) or progestogen-only hormonal contraception (oral, injectable, implantable) associated with inhibition of ovulation initiated 2 or more menstrual cycles prior to screening
    b. intrauterine device (IUD); intrauterine hormone-releasing system (IUS)
    c. bilateral tubal ligation
    d. vasectomized partner (provided that the male vasectomized partner is the sole sexual partner of the study participant and that the partner has obtained medical assessment of surgical success for the procedure).
    e. and/or sexual abstinence†, ‡.

    * Women of childbearing potential are defined as women who are fertile following menarche until becoming post-menopausal, unless permanently sterile. Permanent sterilization methods include hysterectomy, bilateral salpingectomy, and bilateral oophorectomy.

    A post-menopausal state is defined as no menses for 12 months without an alternative medical cause. A high follicle stimulating hormone (FSH) level in the postmenopausal range may be used to confirm a post-menopausal state in women not using hormonal contraception or hormonal replacement therapy. However, in the absence of 12 months of amenorrhea, a single FSH measurement is insufficient to determine the occurrence of a post-menopausal state. The above definitions are according to Clinical Trial Facilitation Group (CTFG) guidance.

    Pregnancy testing and contraception are not required for women with documented hysterectomy.

    † Sexual abstinence is considered a highly effective method only if defined as refraining from heterosexual intercourse during the entire period of risk associated with the study drugs. The reliability of sexual abstinence needs to be evaluated in relation to the duration of the clinical trial and the preferred and usual lifestyle of the patient.

    ‡ Periodic abstinence (calendar, symptothermal, post-ovulation methods), withdrawal (coitus interruptus), spermicides only, and lactational amenorrhea method (LAM) are not acceptable methods of contraception. Female condom and male condom should not be used together.

20. Prior treatment with an anti-CD20 x anti-CD3 bispecific therapy.



VV-RIM-00192456-1.0 Approved - 11 Apr 2022 GMT-5:00

## 6.3. Study Committees

### 6.3.1. Independent Data Monitoring Committee

An Independent Data Monitoring Committee (IDMC) composed of members who are independent from the sponsor and the study sites will be established to conduct formal reviews of accumulated clinical data.

The Independent Data Monitoring Committee (IDMC) for this study will review study safety data on an ongoing basis. The IDMC may make a recommendation to the sponsor to implement changes to study conduct or to halt the study. Such a recommendation will prompt a review by the sponsor, which will decide to implement, modify, or reject the recommendation. Applicable regulatory procedures will be followed as required by local laws in relation to any decisions related to a change in study conduct, temporary halt, study termination, or study restart.

All activities and responsibilities of the IDMC will be described in the IDMC charter.

## 6.4. Premature Discontinuation of Study Medications and Premature Withdrawal from Study

A patient has the right to withdraw from the study at any time, for any reason, and without repercussion.

The investigator and/or sponsor have the right to withdraw a patient from the study if it is no longer in the interest of the patient to continue in the study, or if the patient's continuation in the study places the scientific outcome of the study at risk (eg, if a patient does not or cannot follow study procedures). An excessive rate of withdrawals would render the study uninterpretable; therefore, unnecessary withdrawal of patients should be avoided.

Patients who are withdrawn prematurely from the study will be asked to complete study assessments, as described in Section 8.1.

Rules for discontinuation of study drug (permanent or temporary) are discussed in Section 7.3.

## 6.5. Replacement of Patients

A patient who is prematurely discontinued from the study will not be replaced.

# 7. STUDY TREATMENTS

## 7.1. Investigational Treatment

REGN1979 (odronextamab) will be supplied as a liquid in sterile, single-use vials. A pharmacist or other qualified individual will be identified at each site to prepare odronextamab for administration. The dose administered will be a fixed dose and will not be determined by patient weight or body surface area. Study drug will be administered as an IV infusion. Details and instructions for preparation are provided in the pharmacy manual.

Odronextamab will be administered as a single agent IV at an initial dose of 0.7 mg (0.2 mg/ 0.5 mg), followed by an intermediate dose-1 of 4 mg and intermediate dose-2 of 20 mg, and an assigned QW dose, followed by a Q2W dose (Table 1).

**Table 1: Study Drug QW Dose and Q2W Dose for Each Disease-Specific Cohort**

| Cohort | Odronextamab (mg) QW Dose | Odronextamab (mg) Q2W Dose |
|---|---|---|
| **FL grade 1-3a** | 80 | 160 |
| **DLBCL** | | |
| **Arm 1** | 160 | 320 |
| **Arm 2** | 320 | 320 |
| **MCL after BTK inhibitor therapy** | 160 | 320 |
| **MZL** | 80 | 160 |
| **Other B-NHL** | 160 | 320 |

For the initial dose (0.7 mg administered as a split dose of 0.2 mg and 0.5 mg) and intermediate dose-1 (4 mg) and intermediate dose-2 (20 mg)**,** the treatments will be split into 2 separate infusions, each administered over 4 hours on each of 2 days that are preferably consecutive but no more than 3 days apart (eg, week 1 day 1 and week 1 day 2), with a minimum of 20 hours, as well as a maximum of 76 hours, between the start of the first split infusion and the start of the second split infusion. If a patient does not experience grade 3 CRS prior to treatment week 4, odronextamab will be administered as a single infusion at treatment week 4 and beyond. However, if a patient experiences grade 3 CRS prior to treatment week 4, then odronextamab will be administered as a split infusion over 2 days at treatment week 4. However, in Arm 2 of the DLBCL cohort, patients will receive the first dose of 320 mg at treatment week 4 split administered as 2 separate infusions of 160 mg each, administered each over 4 hours on each of 2 days that are preferably consecutive but no more than 3 days apart; if this odronextamab administration is adequately tolerated, the subsequent administrations may each be as a single infusion.

After treatment week 5 or following the first dose administered as a single infusion, whichever is later, the doses may be administered as a single infusion over 1 to 4 hours depending on tolerability.

## 7.2. Premedications

### 7.2.1. The following premedications apply to odronextamab from the initial dose through the first QW dose as a single infusion. If the patient experiences IRR and/or CRS of any grade with first QW dose, continue premedications until the full QW dose is tolerated without experiencing IRR and/or CRS.

1. **12-24 hours prior to planned start time of first split infusion and prior to planned start of second split infusion if administered on non-consecutive days for each odronextamab dose:**
   a. Dexamethasone 10 mg by mouth, orally (PO) or equivalent dose of steroid
2. **Premedication on each day of split infusion of odronextamab and on the day of QW full dose as a single infusion:**
   a. Dexamethasone 20 mg IV 1 to 3 hours prior to start of infusion on the day of treatment
   b. Diphenhydramine 25 mg IV or PO 30 to 60 min before (can be replaced with another equivalent antihistamine)
   c. Acetaminophen 650 mg PO 30 to 60 min before, unless the patient has received it within the past 4 hours prior to infusion with odronextamab, or is allergic to acetaminophen
3. **24 (±4) hours from the end of second split infusion or end of the first QW full dose as a single infusion**
   a. Dexamethasone 10 mg PO or equivalent dose of steroid

### 7.2.2. First dose administration following QW full dose administered as a single infusion without experiencing an IRR and/ or CRS of any grade with 20 mg of dexamethasone IV

**Premedication for day of odronextamab as a single infusion:**

a. Dexamethasone 10 mg IV 1 to 3 hours prior to start of infusion on the day of treatment
b. Diphenhydramine 25 mg IV or PO 30 to 60 min before (can be replaced with another equivalent anti-histamine)
c. Acetaminophen 650 mg PO 30 to 60 min before, unless the patient has received it within the past 4 hours prior to infusion with odronextamab, or is allergic to acetaminophen

For subsequent doses no premedication is required if the single infusion is tolerated without experiencing an IRR and/ or CRS of any grade with the reduced 10 mg of dexamethasone IV.

NOTE:

- Equivalent dose of steroid includes prednisone/prednisolone 60 mg or methylprednisolone 50 mg (PO dose only).
- Additional premedication with anti-histamines, acetaminophen, and/or nonsteroidal anti-inflammatory drugs (NSAIDs) may also be considered.

If earlier intervention of tocilizumab and enhanced corticosteroid pre- and post-medication is to be implemented following Sponsor notification, please refer to Section 7.3.3.2 .

**GI prophylaxis with corticosteroid usage:** Proton pump inhibitors (PPI) and/or histamine 2 receptor antagonists (H2RA) should be considered for GI prophylaxis against peptic ulcer disease when a patient is on corticosteroids during the step-up dosing period as standard of care.

## 7.3. Dose Modification, Study Drug Discontinuation, and Management of Specific Toxicities

Patients who experience grade ≥3 treatment-related toxicity (excluding laboratory abnormalities that are considered to be clinically insignificant and that do not meet criteria for an AE), that is not otherwise specified in the protocol, should discontinue study treatment until the toxicity resolves to grade 1 or baseline, or until the toxicity is stable and manageable through supportive therapy. The patient may restart study treatment at the same dose or a reduced dose if in the judgment of the investigator and the sponsor it is in the best interest of the patient.

If a prolonged, treatment-free interval refer to Section 7.3.1.2.

For patients who experience CRS, refer to Section 7.3.3 for dose modification guidelines.

For patients who experience tumor lysis syndrome (TLS), refer to Section 7.3.5 for dose modification guidelines.

### 7.3.1. Study Treatment Discontinuation

During the treatment period, patients who discontinue study drug but do not withdraw from the study will be asked to return to the clinic for follow-up visits starting with Safety Follow-up Visits at 1, 2, and 3 and will be expected to continue with all relevant study assessments (eg, efficacy assessments and/or safety assessments) according 8.1. Patients who have discontinued study drug for any reason other than disease progression, start of non-protocol anti-lymphoma therapy, withdrawal of consent, or death, disease response will be assessed until the time of disease progression, death, start of a non-protocol anti-lymphoma therapy, or patient withdrawal of consent for follow-up of disease status, whichever is sooner.

During the treatment period and follow-up period, a patient who withdraws consent to continue participation in the study will not be followed for any reason after consent has been withdrawn. Patients who have discontinued from extended follow-up but have not withdrawn consent will continue survival follow-up until the time of death, loss to follow-up, withdrawal of consent, or study termination by the sponsor, whichever is earlier.

#### 7.3.1.1. Reasons for Permanent Discontinuation of Study Drug

Study drug dosing will be permanently stopped in the event of:

- Evidence of pregnancy
- Severe allergic reactions considered related to study drug (see Section 7.3.2.1)
- Patient withdrawal of consent at any time

- Any medical condition that the investigator or sponsor determines may jeopardize the patient's safety if he or she continues in the study or continues treatment with study drug
- The investigator or sponsor determines it is in the best interest of the patient
- Patient non-compliance (eg, not complying with protocol required visits, assessments, and dosing instructions)
- Start of non-protocol anti-lymphoma therapy
- Tuberculosis or severe uncontrolled opportunistic infections
- Diagnosis of another malignancy during the study with the exception of localized melanoma, non-melanoma skin cancer that has undergone potentially curative therapy or in situ cervical carcinoma, or any other tumor that has been deemed to be effectively treated with definitive local control and with curative intent
- Multi-focal leukoencephalopathy
- Clear evidence of progressive disease (patients with suspected pseudo-progression may continue study drug until such a time that there is definitive evidence of disease progression, as described in Section 5.1.3.4). Other criteria for permanent discontinuation include:
  - Grade 4 CRS, as described in Table 3 and Table 4
  - Grade ≥3 neurotoxicity, as described in Section 7.3.6
  - TLS that does not resolve, as described in Table 9 and Section 7.3.6

#### 7.3.1.2. Restarting Study Drug Administration Following Temporary Treatment Interruption

For patients who had temporary treatment interruption, the sponsor should be consulted to discuss the patient's individual risk factors, such as prior history of CRS, before re-instituting study therapy in the following scenarios:

- patients who received the full QW dose for less than 5 preceding doses and had a temporary treatment interruption lasting greater than 21 days
- patients who received the full QW dose for ≥5 prior doses and had a temporary treatment interruption lasting greater than 42 days

The sponsor and the investigator will develop a plan to minimize the risk of CRS upon resuming study therapy which may include prolonged observation, infusion over 4 hours, premedications, split dosing, and/or resuming therapy with the initial dose used in week 1 prior to increasing the dose the following week.

### 7.3.2. Safety Monitoring and Management of Specific Toxicities

Measures will be taken to ensure the safety of patients participating in this trial. In particular, stringent eligibility criteria (Section 6.2) and close clinical safety monitoring per schedule of assessments (Section 8.1) will be employed. Clinical monitoring will include frequent study visits

and evaluation by study staff, including physical examinations, and routine and specialty laboratory testing, in addition to assessment for toxicity and AEs. For TLS monitoring and management guidance refer to Section 7.3.5.

Detailed procedures on safety monitoring of patients is in Section 8.2.3.1.

#### 7.3.2.1. Acute Allergic/Hypersensitivity Infusion Reactions

Emergency equipment and medication/ hypersensitivity kit that includes epinephrine, normal saline, antihistaminic and corticosteroid must be available for immediate use. Acute allergic/hypersensitivity infusion reactions must be reported as AEs (Section 9.4.1) and graded according to the current version of the National Cancer Institute Common Terminology Criteria for Adverse Events (NCI-CTCAE version 5) (Section 9.5.1).

##### 7.3.2.1.1. Interruption of the Intravenous Infusion

The infusion should be interrupted if any of the following AEs are observed:

- cough
- rigors/chills
- rash, pruritus (itching)
- urticaria (hives, welts, wheals)
- diaphoresis (sweating)
- hypotension
- dyspnea (shortness of breath)
- vomiting
- flushing

The reaction(s) should be treated as appropriate for the clinical signs and symptoms, and the infusion may be restarted at 50% of the original rate.

If the investigator determines that there is a medical need for treatment or discontinuation of the infusion other than described above, he or she should use clinical judgment to provide the appropriate response according to typical clinical practice.

##### 7.3.2.1.2. Termination of the Intravenous Infusion

The infusion should be terminated and NOT restarted if any of the following AEs occur:

- Anaphylaxis*
- Laryngeal/pharyngeal edema
- Severe bronchospasm
- Chest pain
- Seizure
- Severe hypotension

- Other neurological symptoms (confusion, loss of consciousness, paresthesia, paralysis, etc.)
- Any other symptom or sign that, in the opinion of the investigator, warrants termination of the IV infusion

*Consider anaphylaxis if the following is observed (Sampson, 2006): acute onset of an illness (minutes to several hours) with involvement of the skin, mucosal tissue, or both (eg, generalized hives, pruritus or flushing, swollen lips-tongue-uvula) AND AT LEAST ONE OF THE FOLLOWING

- Respiratory compromise (eg, dyspnea, wheeze-bronchospasm, stridor, reduced peak expiratory flow, hypoxemia)
- Reduced blood pressure or associated symptoms of end-organ dysfunction (eg, hypotonia [collapse], syncope, incontinence)

### 7.3.3. Grading and Management of Infusion-related Reaction and Cytokine Release Syndrome

#### 7.3.3.1. Standard Intervention of Tocilizumab and Corticosteroid

An acute IRR is defined as any AE that occurs less than 6 hours from the start of the infusion, or within 2 hours after completion of the infusion (whichever is later), and is associated with typical signs and symptoms including, but not limited to, flushing, tachycardia, hypotension, dyspnea, bronchospasm, back pain, fever, urticaria, edema, nausea, and rashes. Infusion-related reactions (IRRs), defined in Section 9.3.4 must be reported as AEs (Section 9.4.1) and graded according to the NCI-CTCAE v5 (Section 9.5.1).

Cytokine release syndrome is a disorder characterized by fever, tachypnea, headache, tachycardia, hypotension, rash, and/or hypoxia; in this study it is defined as such an event that occurs 6 or more hours from the start of the infusion or more than 2 hours after completion of the infusion (whichever is later). Emergency equipment and medication for the treatment of these potential adverse effects (eg, tocilizumab, antihistamines, bronchodilators, IV saline, corticosteroids, acetaminophen, and/or epinephrine) must be available for immediate use. Cytokine release syndrome must be reported as AEs (Section 9.4) and graded according to the criteria adapted from (Lee, 2014)(Table 2). Cytokine release syndrome has been observed in patients treated with odronextamab (Bannerji, 2017), and it has also been described in published data with other bispecific antibodies that target CD3, such as blinatumomab, and anti-CD19 chimeric antigen receptor T-cells (Davila, 2014) (Nagorsen, 2012) (Teachey, 2013). These therapies have been associated with early onset and reversible IRR or CRS. Refer to the Investigator's Brochure for further details on CRS.

An extensive clinical and laboratory monitoring plan is in place to allow for early identification of patients who are more likely to develop CRS, and this allows for early initiation of increased patient monitoring (eg, hospital admission) and therapeutic intervention.

Patients who develop symptoms consistent with severe CRS, including but not limited to persistent fever, hypotension, or hypoxia, should be considered for pharmacologic intervention with tocilizumab, corticosteroid and other interventions according to the clinical judgment of the

investigator (Davila, 2014) (Grupp, 2013) (Lee, 2014). Additional guidance regarding the use of tocilizumab is provided in Table 5. Patient's age and the extent of the comorbidities will need to be taken into consideration by the investigator in determining the need for earlier use of tocilizumab for any grade of CRS. In addition, sites should ensure that two doses of tocilizumab are available before any study drug is administered during step-up dosing of every patient.

Cytokine release syndrome management guidelines, including clinical evaluation and monitoring, are detailed in Table 3, Table 4, and Table 5.

Emergency equipment and medication for the treatment of infusion associated reactions must be available for immediate use.

### Table 2: Cytokine Release Syndrome Toxicity Grading*

| CRS Parameter | Grade 1 | Grade 2 | Grade 3 | Grade 4 |
|---|---|---|---|---|
| Fever[1] | Temperature ≥ 38°C | Temperature ≥ 38°C | Temperature ≥ 38°C | Temperature ≥ 38°C |
| With | | | | |
| Hypotension | None | Not requiring vasopressors | Requiring a vasopressor with or without vasopressin | Requiring multiple vasopressors (excluding vasopressin) |
| And/or[2] | | | | |
| Hypoxia | None | Requiring low-flow nasal cannula[3] or blow-by | Requiring high-flow nasal cannula[3], facemask, nonrebreather mask, or Venturi mask | Requiring positive pressure (eg CPAP, BiPAP, intubation and mechanical ventilation) |

*Adapted from American Society for Transplantation and Cellular Therapy (ASTCT) Cytokine Release Syndrome Consensus Grading (Lee, 2019).

CPAP= continuous positive airway pressure, bilevel positive airway pressure, BiPAP=bilevel positive airway pressure.

Organ toxicities associated with CRS may be graded according to CTCAE v5.0 but they do not influence CRS grading.

[1] Fever is defined as temperature ≥ 38°C not attributable to any other cause. In patients who have CRS then receive antipyretic or anti-cytokine therapy such as tocilizumab or steroids, fever is no longer required to grade subsequent CRS severity. In this case, CRS grading is driven by hypotension and/or hypoxia.

[2] CRS grade is determined by the more severe event: hypotension or hypoxia not attributable to any other cause. For example, a patient with temperature of 39.5°C, hypotension requiring 1 vasopressor, and hypoxia requiring low-flow nasal cannula is classified as grade 3 CRS.

[3] Low-flow nasal cannula is defined as oxygen delivered at ≤ 6 L/minute. Low flow also includes blow-by oxygen delivery, sometimes used in pediatrics. High-flow nasal cannula is defined as oxygen delivered at >6 L/minute.

**Table 3: Management Guidance and Odronextamab Dosing in Setting of Cytokine-Mediated Toxicities- Infusion-related Reactions (IRR)**

| NCI-CTCAE v5.0 Severity Grade* | Action with Odronextamab | Management Guide | Rechallenge: |
|---|---|---|---|
| 1 | No action taken | Intervention not indicated | No dose modification required |
| 2 | Temporarily pause study drug | Treat symptomatically. Supportive care, antipyretics (eg, acetaminophen/paracetamol), meperidine | No dose modification required<br>May resume when clinical symptoms are at baseline |
| 3 | Temporarily pause study drug | Treat symptomatically. Supportive care, antipyretics (eg, acetaminophen/paracetamol), meperidine. Consider corticosteroids | May resume treatment when clinical symptoms are grade 1 or baseline<br>• If with initial dose, retreat with initial dose and escalate as tolerated<br>• If with intermediate dose-1 or intermediate dose-2, reduce dose by 50% of intermediate dose retreat with dose that is no higher than intermediate dose and escalate as tolerated<br>• If with first QW full dose, reduce to no more than 50% of the QW full dose and escalate to QW full dose as tolerated. Continue dosing if no recurrence of toxicity. |
| 4 | Permanently discontinue | Treat symptomatically. Supportive care, antipyretics (eg, acetaminophen/paracetamol), meperidine. Consider corticosteroids | |

* Infusion-related reactions will be graded according to criteria specified in NCI-CTCAE v5.0. Consistent with these criteria, grade 1 infusion-related reactions require no infusion interruption or intervention.

**Table 4: Management Guidance and Odronextamab Dosing in Setting of Cytokine Mediated Toxicities: Cytokine Release Syndrome (CRS) and Other Adverse Events**

| Event | Grade | Action with Odronextamab | Management Guide | Rechallenge |
|---|---|---|---|---|
| CRS* | 1 | Temporarily pause study drug | • Vigilant supportive care (assess for infection;<br>• treat febrile neutropenia if present, monitor fluid balance,<br>• antipyretics/analgesics and IV fluids as needed;<br>• monitor cardiac and other organ functions closely;<br>• symptomatic management of organ toxicities).<br>• Monitor vital signs at least every hour ±30 minutes until resolution of clinical symptoms. Additional monitoring may be required as clinically indicated<br><br>Treatment: **Refer to** Table 5<br><br>**If notified by Sponsor to implement earlier intervention of tocilizumab and enhanced corticosteroid Pre/Post Administration refer to** Table 6 | No dose modification required.<br>May resume when clinical symptoms are resolved to baseline |
| CRS* | 2 | Temporarily pause study drug | • Vigilant supportive care (assess for infection;<br>• treat febrile neutropenia if present, monitor fluid balance, antipyretics/analgesics and IV fluids as needed;<br>• monitor cardiac and other organ functions closely;<br>• symptomatic management of organ toxicities); supplemental oxygen as needed.<br>• Monitor vital signs every hour ±30 minutes until resolution of clinical symptoms.<br><br>Treatment: | No dose modification required.<br>May resume when clinical symptoms are baseline |

| Event | Grade | Action with Odronextamab | Management Guide | Rechallenge |
|---|---|---|---|---|
| | | | **Refer to** Table 5<br><br>**If notified by Sponsor to implement earlier intervention of tocilizumab and enhanced corticosteroid Pre/Post Administration refer to** Table 6 | |
| | 3 | Temporarily pause study drug | • Vigilant supportive care as per grade 2;<br>• vasopressor support for fluid refractory hypotension and intensive care unit (ICU) care as needed;<br>• supplemental oxygen as needed.<br><br>Treatment: **Refer to** Table 5<br><br>**If notified by Sponsor to implement earlier intervention of tocilizumab and enhanced corticosteroid Pre/Post Administration refer to** Table 6 | Repeat dose if it occurred with initial dose. For intermediate dose-1 or intermediate dose-2, or full QW dose, reduce by at least 50%. May resume when clinical symptoms are baseline |
| | 4 | Permanently discontinue | • Vigilant supportive care as per grade 2;<br>• ICU care, hemodynamic monitoring, vasopressor support, ventilatory support as needed;<br><br>Treatment: **Refer to** Table 5<br><br>**If notified by Sponsor to implement earlier intervention of tocilizumab and enhanced corticosteroid Pre/Post Administration refer to** Table 6 | Not applicable |
| CRS associated transaminase elevation | Grade ≥3 | Temporarily pause study drug | Supportive care as needed, monitor closely. Consider corticosteroids if not trending toward normal within 7 days.<br>Study drug will be temporarily withheld, and subsequent study drug treatment administrations will not | No change to dose if not associated with clinical symptoms and is trending towards normal within 7 days. May re-challenge with |

| Event | Grade | Action with Odronextamab | Management Guide | Rechallenge |
|---|---|---|---|---|
| | | | resume until laboratory parameters are trending toward normal | either initial dose or a previous lower dose if resolution longer than 7 days. |

If subsequent administration is tolerated, patients who received a reduced dose may have their dose increased at subsequent administration but no higher than the QW full dose, based on the clinical judgment of the investigator in consultation with the sponsor.
*Adapted from (Lee, 2019) and (Neelapu, 2018b).

### Table 5: Guidelines for Administration of Tocilizumab and Corticosteroids in the Setting of Cytokine Release Syndrome

| CRS Grade | Administer tocilizumab* in the following scenarios | Administer corticosteroid |
|---|---|---|
| **Any grade CRS** | Earlier use of tocilizumab is recommended for elderly patients (age> 65 years) or for patients with co-morbidities as per investigator's clinical judgement | • Refer to corresponding grade of CRS |
| **Grade 1 CRS** | • Patients with persistent [lasting >3 days] and refractory fever | • Dexamethasone 10 mg IV<br>• (or equivalent corticosteroid) every 24 hours |
| **Grade 2 CRS** | • Hypotension that is refractory to fluid boluses,<br>• Hypoxia<br>• Left ventricular ejection fraction <40% by echocardiogram<br>• Creatinine >2.5-fold higher than the most recent level prior to odronextamab infusion<br>• Activated partial thromboplastin time >2x the upper limit of normal<br>• Clinically significant bleeding<br>• Creatine kinase >5x the upper limit of normal for longer than 2 days | • Dexamethasone 10 mg IV (or equivalent corticosteroid) every 12-24 hours.<br>• If no improvement or rapid progression of CRS, increase dexamethasone to 10-20 mg IV (or equivalent corticosteroid) every 6-12 hours |
| **Grade 3 CRS** | • Any grade 3 | • Dexamethasone 10-20 mg IV (or equivalent corticosteroid) every 6-12 hours |
| **Grade 4 CRS** | • Any grade 4 | • Dexamethasone 20 mg IV (or equivalent corticosteroid) every 6 hours |

| CRS Grade | Administer tocilizumab* in the following scenarios | Administer corticosteroid |
|---|---|---|
| *** tocilizumab dose:** 8 mg/kg infused over 1 hour, dose not to exceed 800 mg. Repeat doses of tocilizumab may be administered as required according to the label (if no clinical improvement in the signs and symptoms of CRS occurs after the first dose, up to 3 additional doses may be administered. The interval between consecutive doses should be at least 8 hours)<br>Sites should ensure that two doses of tocilizumab are available before any study drug is administered during step-up dosing of every patient<br>Guidelines adapted from(Brudno, 2016), (Neelapu, 2018b, Maus, 2020) | | |

### 7.3.3.2. Earlier Intervention of Tocilizumab and Enhanced Corticosteroid Pre- and Post-Medication ONLY Following Sponsor Notification

**THIS WILL BE IMPLEMENTED ONLY FOLLOWING SPONSOR NOTIFICATION IF TWO GRADE ≥3 CRS EVENTS ARE OBSERVED WITH THE REVISED STEP-UP REGIMEN).**

#### 7.3.3.2.1. Premedication

The following premedications apply to odronextamab administration from the initial dose through the first QW dose as a single infusion. If the patient experiences IRR and/or CRS of any grade with first QW dose, continue premedications until the full QW dose is tolerated without experiencing IRR and/or CRS.

1. **12-24 hours prior to planned start time of first split infusion and prior to planned start of second split infusion if administered on non-consecutive days for each odronextamab dose:**
   a. Dexamethasone 20 mg PO or equivalent dose of steroid
2. **Premedication on each day of split infusion of odronextamab and on the day of QW full dose as a single infusion:**
   a. Dexamethasone 20 mg IV 1 to 3 hours prior to start of infusion on the day of treatment
   b. Diphenhydramine 25 mg IV or PO 30 to 60 min before (can be replaced with another equivalent antihistamine)
   c. Acetaminophen 650 mg PO 30 to 60 min before, unless the patient has received it within the past 4 hours prior to infusion with odronextamab, or is allergic to acetaminophen
3. **24 (±4) hours from the end of second split infusion or end of the first QW full dose as a single infusion**
   a. Dexamethasone 20 mg PO or equivalent dose of steroid

**First dose administration** following QW full dose administered as a single infusion without experiencing an IRR and/ or CRS of any grade with 20 mg of dexamethasone IV

a) Dexamethasone 10 mg IV 1 to 3 hours prior to start of infusion on the day of treatment
b) Diphenhydramine 25 mg IV or PO 30 to 60 min before (can be replaced with another equivalent anti-histamine)

c) Acetaminophen 650 mg PO 30 to 60 min before, unless the patient has received it within the past 4 hours prior to infusion with odronextamab, or is allergic to acetaminophen

For subsequent doses no premedication is required if the single infusion is tolerated without experiencing an IRR and/ or CRS of any grade with the reduced 10 mg of dexamethasone IV.

NOTE:

- Equivalent dose of steroid includes prednisone/prednisolone 120 mg or methylprednisolone 100 mg (PO dose only).
- Additional premedication with anti-histamines, acetaminophen, and/or nonsteroidal anti-inflammatory drugs (NSAIDs) may also be considered.

**GI prophylaxis with corticosteroid usage:** Proton pump inhibitors (PPI) and/or histamine 2 receptor antagonists (H2RA) should be considered for GI prophylaxis against peptic ulcer disease when a patient is on corticosteroids during the step-up dosing period as standard of care.

#### 7.3.3.2.2. Administration of Tocilizumab

Please refer to Table 6.

**Table 6: Guidelines for EARLIER Administration of Tocilizumab in the Setting of Cytokine Release Syndrome**

| Grade | Administer tocilizumab in the following scenarios | Administer corticosteroid |
|---|---|---|
| **Any grade CRS** | • Earlier use of tocilizumab is recommended for elderly patients (age> 65 years) or for patients with co-morbidities as per investigator's clinical judgement | • Refer to corresponding grade of CRS |
| **Grade 1 CRS** | • Patients with persistent fever (lasting > 24 hours) and refractory fever | • Dexamethasone 10 mg IV<br>• (or equivalent corticosteroid) every 24 hours |
| **Grade 2 CRS** | Any grade ≥ 2 | • Dexamethasone 10 mg IV (or equivalent corticosteroid) every 12-24 hours.<br>• If no improvement or rapid progression of CRS, increase dexamethasone to 10-20 mg IV (or equivalent corticosteroid) every 6-12 hours |
| **Grade 3 CRS** | | • Dexamethasone 10-20 mg IV (or equivalent |



VV-RIM-00192456-1.0 Approved - 11 Apr 2022 GMT-5:00

| Grade | Administer tocilizumab in the following scenarios | Administer corticosteroid |
|---|---|---|
| | | corticosteroid) every 6-12 hours |
| **Grade 4 CRS** | | • Dexamethasone 20 mg IV (or equivalent corticosteroid) every 6 hours |
| *** tocilizumab dose:** 8 mg/kg infused over 1 hour, dose not to exceed 800 mg. Repeat doses of tocilizumab may be administered as required according to the label (if no clinical improvement in the signs and symptoms of CRS occurs after the first dose, up to 3 additional doses may be administered. The interval between consecutive doses should be at least 8 hours)<br>Sites should ensure that two doses of tocilizumab are available before any study drug is administered during step-up dosing of every patient<br><br>Guidelines adapted from (Brudno, 2016), (Neelapu, 2018b, Maus, 2020) | | |

### 7.3.4. B-Cell Depletion and Infection Prophylaxis

Treatment with odronextamab is expected to result in pronounced and prolonged B-cell depletion resulting in increased risk of infection. Patients with known active bacterial, viral, fungal, mycobacterial, or other infection or any major episode of infection requiring hospitalization or treatment with IV anti-infectives within 2 weeks prior to first administration of odronextamab are excluded from the trial.

Patients with infections other than tuberculosis or opportunistic infection may resume treatment with study drug only if the infection is adequately controlled. In the case of HBV reactivation, the patient must have an undetectable viral load by HBV DNA-PCR (or equivalent method) on anti-viral therapy for hepatitis B.

Cytomegalovirus (CMV) PCR testing in blood will be done during screening, week 6 (±1 week), week 12 (±1 week), week 24 (±2 weeks) and at other timepoints when clinically indicated. Cytomegalovirus PCR must be undetectable prior to enrollment.

If CMV DNA levels are detected at any time, consider antiviral treatment for CMV as deemed appropriate by the investigator and contact the Medical Monitor for additional recommendations; continue weekly monitoring by PCR until viral load decreases, and then every 2 weeks (±1 week) until 2 consecutive undetectable results.

Infections of grade 3 severity and above and CMV infection of any grade should be reported as adverse events of special interest (AESI).

#### 7.3.4.1. Recommendations for Prophylaxis to Decrease the Risk of Infections:

1. Patients should have evaluation of IgG levels at baseline as well as periodic monitoring throughout the study. In patients with severe hypogammaglobulinaemia (<400 mg/dL) or in patients with recurrent episodes of infection with immunoglobulin levels between 400-600 mg/dL, supplementation with intravenous immunoglobulin (IVIG) is recommended in accordance with the local institutional guidelines.

2. For patients with positive hepatitis B surface antigen, Hepatitis B core antibody (HBcAB), and/or measurable viral load, appropriate antiviral agent for HBV is recommended.
3. Prophylaxis for Pneumocystis jirovecii pneumonia (PJP) is recommended for all patients. The choice of the prophylactic agent and the duration of prophylaxis is in accordance with the local institutional guidelines.
4. Appropriate antiviral prophylaxis for patients with prior Herpes Simplex (HSV) or CMV infections is recommended. The choice of the prophylactic agent and the duration of prophylaxis is in accordance with the local institutional guidelines.
5. Standard measures of prophylaxis for infections should be considered in accordance with the local institutional standards, as well as National Comprehensive Cancer Network (National Comprehensive Cancer Network [NCCN], 2019), American Society of Clinical Oncology (ASCO) (Taplitz, 2018), or European Society for Medical Oncology (ESMO) (Klastersky, 2016) guidelines.

### 7.3.5. Tumor Lysis Syndrome

#### 7.3.5.1. Recommendations for Tumor Lysis Syndrome Prophylaxis

All patients should have adequate fluid intake (approximately 2 to 3 L/day) oral or IV (if unable to take oral fluids) starting 1 to 2 days prior to the odronextamab infusion and continued for at least 24 hours after end of infusion until the patient tolerates the QW full dose of odronextamab administered as a single infusion, through week 4 of study treatment, or until the investigator determines that the patient is not at risk for TLS, whichever is later.

Patients who are considered to be at risk for TLS (for example, patients with aggressive NHL, and patients otherwise classified as intermediate or high risk (as described in Table 7 below) should have the following additional measures taken for TLS prophylaxis:

- Such patients should receive prophylaxis with allopurinol (or other hypouricemic agent). Allopurinol should begin preferably 7 to 10 days prior to the first infusion of study drug, but not less than 48 hours prior to the first administration. Patients who cannot tolerate allopurinol or other hypouricemic agent and who are at risk for TLS should be monitored closely and treated with rasburicase according to the prescribing information and pertinent institutional guidelines for TLS prophylaxis.
- In addition to oral hydration noted above, IV hydration (approximately 1.5−2 L) should be administered as permitted by the patient's hemodynamic status and according to the investigator's clinical judgment.
- If laboratory abnormalities (see Table 8 below)
- that in the investigator's judgment indicate ongoing TLS are observed in this baseline laboratory assessment, the first dose of study treatment must be delayed until resolution of laboratory abnormalities. If needed, the patient should receive an extended period of TLS prophylaxis prior to the initiation of odronextamab dosing.
- Patients should continue oral hypouricemic agent (if feasible) or rasburicase until the patient tolerates the QW full dose of odronextamab administered as a single infusion,

through week 4 of study treatment, or until the investigator determines that the patient is not at risk for TLS, whichever is later.

#### 7.3.5.2. Required Monitoring for Tumor Lysis Syndrome

The TLS risk (Table 7) should be assessed by the investigator before each study drug administration (including an assessment prior to each split infusion, if applicable) until the patient has been determined to tolerate the QW full dose of odronextamab administered as a single infusion, through week 4 of study treatment, or until the investigator determines that the patient is not at risk for TLS, whichever is later.

**Table 7: Tumor Lysis Syndrome Risk Classification and Monitoring**

| Risk Classification | Criteria (any one of the below) | Required Monitoring |
|---|---|---|
| **Low Risk** | • All measurable lymph nodes with largest diameter <5 cm by radiologic assessment<br>• An absolute lymphocyte counts $<25 \times 10^9$/L.<br>• LDH <2 x ULN | Should be monitored according to standard clinical practice or local institutional guidelines. |
| **Intermediate Risk** | • Any measurable lymph node with largest diameter ≥5 cm and <10 cm by radiologic assessment<br>• An absolute lymphocyte count $\geq 25 \times 10^9$/L<br>• LDH ≥2 x ULN | Evaluations for TLS should be performed prior to study drug administration and approximately 8 and 24 hours following the start of study drug administration. |
| **High Risk** | • The presence of any lymph node with the largest diameter ≥10 cm by radiologic assessment<br>• The presence of<br>  ○ an absolute lymphocyte count $\geq 25 \times 10^9$/L AND<br>  ○ a measurable lymph node with the largest diameter ≥5 cm by radiologic assessment<br>• Patients with intermediate risk AND<br>  ○ Renal dysfunction (creatinine clearance <80 mL/min OR<br>  ○ uric acid, potassium and/or phosphate >ULN<br>• Bone marrow infiltration ≥5%<br>• Splenomegaly due to lymphoma | Evaluations for TLS should be performed prior to study drug administration and approximately 4, 8, 12 and 24 hours following the start of study drug administration. |

Laboratory investigations for TLS pre-dose and at approximately 4, 8, 12 and 24 hours following the start of study drug administration, as discussed in Table 7 and below, should include serum calcium, potassium, phosphate, uric acid, LDH and creatinine (Table 8).

Laboratory samples should be sent for immediate analysis, and the results must be reviewed promptly by the investigator. It is important to ensure that a nephrology consultation, emergency dialysis, and telemetry are available if needed to manage TLS.

If any laboratory or clinical abnormality indicative of TLS in the investigator's clinical judgment is observed in the pre-dose laboratory assessment (including prior to each split infusion if applicable), the study treatment must be delayed until resolution. Pre-dose laboratory results must be reviewed prior to administration of odronextamab.

Patients who are considered to be at intermediate or high risk for TLS by the investigator must be hospitalized for study drug administration and monitored in an inpatient setting. The 24-hour laboratory results must be reviewed before the patient is discharged. If there is evidence of ongoing TLS, additional laboratory assessments and close observation should be considered beyond 24 hours following the study drug administration.

**Table 8: Diagnosis of Laboratory and Clinical Tumor Lysis Syndrome***

| Metabolic Abnormality | Criteria for Classification of Laboratory Tumor Lysis Syndrome | Criteria for Classification of Clinical Tumor Lysis Syndrome |
|---|---|---|
| **Hyperuricemia** | Uric acid >8.0 mg/dL (475.8 µmol/L) | |
| **Hyperphosphatemia** | Phosphorus >4.5 mg/dL (1.5 mmol/L) | |
| **Hyperkalemia** | Potassium >6.0 mmol/L | Cardiac dysrhythmia or sudden death probably or definitely caused by hyperkalemia |
| **Hypocalcemia** | Corrected calcium <7.0 mg/dL (1.75 mmol/L) or ionized calcium <4.5 mg/dL (1.12 mmol/L)† | Cardiac dysrhythmia, sudden death, seizure, neuromuscular irritability (tetany, paresthesia, muscle twitching, carpopedal spasm, Trousseau's sign, Chvostek's sign, laryngospasm, or bronchospasm), hypotension, or heart failure probably or definitely caused by hypocalcemia |
| **Acute kidney injury ‡** | Not applicable | Increase in the serum creatinine level of 0.3 mg/dL (26.5 µmol/L) (or a single value >1.5 times the upper limit of the age appropriate normal range if no baseline creatinine measurement is available) or the presence of oliguria, defined as an average urine output of <0.5 mL/kg/hr for 6 hr |

* In laboratory tumor lysis syndrome, 2 or more metabolic abnormalities must be present during the same 24-hour period within 3 days before the start of therapy or up to 7 days afterward. Clinical tumor lysis syndrome requires the presence of laboratory tumor lysis syndrome plus an increased creatinine level, seizures, cardiac dysrhythmia, or death. The laboratory abnormalities or clinical abnormalities should be suggestive of TLS in the opinion of the investigator.

† The corrected calcium level in milligrams per deciliter = measured calcium level in milligrams per deciliter + 0.8 x (4 – albumin in grams per deciliter).

‡ Acute kidney injury is defined as an increase in the creatinine level of at least 0.3 mg per deciliter (26.5 µmol per liter) or a period of oliguria lasting 6 hours or more. By definition, if acute kidney injury is present, along with at least 1 other metabolic abnormality indicative of tumor lysis syndrome, (as noted in Table 8) then the patient is considered to have clinical tumor lysis syndrome. In case of acute kidney injury, other co-existing conditions or differential diagnoses such as CRS, IRR, and other relevant pre-renal, intrinsic renal and post-renal causes should also be considered.

(Adapted from (Howard, 2011)).



VV-RIM-00192456-1.0 Approved - 11 Apr 2022 GMT-5:00

### 7.3.5.3. Dose Modification of Odronextamab for Patients who Experience Tumor Lysis Syndrome

If a patient receiving study treatment develops one or more laboratory or clinical abnormalities that are judged by the investigator to indicate TLS, study drug administration must be suspended immediately until all laboratory abnormalities and clinical signs/symptoms of TLS have resolved. Thus, if the patient is receiving a split dose, laboratory evaluation for TLS must be done after the first half dose, and the second half dose must not be administered during the current cycle if there are any signs of TLS.

**Table 9: Management Guidance and Study Drug Dosing in Setting of Tumor Lysis Syndrome**

<table>
<tr><th>Study Drug Administration</th><th>Outcome of TLS[1]</th><th>Action with Odronextamab</th><th>Rechallenge[2]</th><th>TLS Management Guidance</th></tr>
<tr><td rowspan="2">TLS occurs with Initial dose</td><td>Any grade of TLS that does not resolve</td><td>Permanently discontinue</td><td>Not applicable</td><td rowspan="3">A patient who has laboratory or clinical abnormalities indicative of TLS should be hospitalized for monitoring.<br>IV fluids should be initiated (approximately 150 to 200 mL/h).<br>A rapidly rising serum potassium level is a medical emergency and should be managed according to standard clinical practice or local institutional guidelines. Other electrolyte abnormalities including hypocalcemia, hyperphosphatemia and hyperuricemia should be managed according to standard clinical practice or local institutional guidelines.<br>Monitor for symptoms or signs of TLS. If any clinical features are observed, recheck potassium, phosphorus, uric acid, calcium and creatinine with an expedited assessment.<br>Once the diagnosis of TLS is determined, intensive monitoring and multi-disciplinary management will be according to standard clinical practice or local institutional guidelines. Strongly recommend consultation with the nephrology service to ensure that emergency dialysis is available. Ensure that telemetry is available for monitoring.</td></tr>
<tr><td>Any grade of TLS that resolves</td><td>Temporary pause</td><td>Upon resolution of TLS, resume treatment with only the initial split dose (0.2 mg) and monitor for TLS (Table 8).<br>If no recurrence of TLS, then increase next dose to 0.7 mg administered as split doses (0.2/0.5 mg) at least 2 days apart but not more than 3 days apart.<br>If no recurrence of TLS, dose may be escalated to a split intermediate dose- 1 at least 2 days apart but not more than 3 days apart.<br>If no recurrence of TLS with intermediate dose-1, escalate to next higher dose specified in protocol (ie, intermediate dose-2) as split dose at least 2 days apart but not more than 3 days apart.</td></tr>
<tr><td>TLS occurs with Intermediate doses and/or subsequent doses</td><td>Any grade of TLS that does not resolve</td><td>Permanently discontinue</td><td>Not applicable</td></tr>
</table>

| Study Drug Administration | Outcome of TLS[1] | Action with Odronextamab | Rechallenge[2] | TLS Management Guidance |
|---|---|---|---|---|
| | Any grade of TLS that resolves | Temporary pause | Upon resolution of TLS, reduce previously received dose by 50% and monitor for TLS (Table 8).<br><br>If no recurrence of TLS, then escalate to the dose where TLS was last noted. This dose needs to be administered as a split dose **at least 2 days** apart but not more than 3 days apart.<br><br>If no recurrence of TLS, dose may escalate to next higher dose specified in protocol as split dose **at least 2 days** apart but not more than 3 days apart. | |

1 Resolution of TLS is defined as no clinical or laboratory abnormalities suggestive of TLS in the investigator's judgment.
2 If 2 consecutive TLS events occur, discuss the plan of study treatment with the Regeneron medical monitor. The goal is to idle the patient at a dose just below that which produces TLS until there is sufficient debulking of the tumor to allow dose escalation.

### 7.3.6. Central Nervous System Toxicity

Patients who experience grade ≥3 signs or symptoms consistent with CNS toxicity (eg, seizure, somnolence) at any time on treatment will be required to discontinue study drug permanently, if the signs or symptoms are partially or wholly attributed to odronextamab with no plausible alternate etiology. If an etiology other than odronextamab treatment is likely to be the major causative factor, then study drug must be suspended until signs and symptoms resolve to grade 1 or baseline, and study drug may be resumed at the discretion of the investigator after discussion with the medical monitor. Patients who experience lower grade signs or symptoms consistent with CNS toxicity must also suspend study drug at least temporarily until symptoms resolve to grade 1 or baseline. Patients should undergo appropriate evaluation to determine the etiology of the symptoms. Any decision to restart treatment upon resolution of symptoms must be discussed and agreed upon by the investigator and the sponsor.

## 7.4. Method of Treatment Assignment

All patients who sign the ICF will be assigned a patient number. For the initial step of the DLBCL cohort, treatment assignment will be performed by randomization (1:1) to Arm 1 or Arm 2. Following the initial randomization step, patients in the DLBCL cohort will be assigned to the 160 mg QW/320 mg Q2W dose regimen. For the other disease-specific cohorts, treatment will be assigned to the regimen which was prespecified for the cohort.

### 7.4.1. Blinding

Not applicable. This is an open-label study.

## 7.5. Treatment Logistics and Accountability

### 7.5.1. Packaging, Labeling, and Storage

Open-label study drug will display the product lot number on the label.

Study drug will be stored at the site at a temperature of 2ºC to 8ºC; storage instructions will be provided in the pharmacy manual.

### 7.5.2. Supply and Disposition of Treatments

Study drug will be shipped at a temperature of 2ºC to 8ºC to the investigator or designee at regular intervals or as needed during the study. At specified time points during the study (eg, interim site monitoring visits), at the site close-out visit, and following drug reconciliation and documentation by the site monitor, all opened and unopened study drug will be destroyed / returned to the sponsor or designee.

### 7.5.3. Treatment Accountability

All drug accountability records must be kept current.

The investigator must be able to account for all opened and unopened study drug. These records should contain the dates, quantity, and study medication

- dispensed to each patient,
- returned from each patient (if applicable), and
- disposed of at the site (must be approved by the sponsor) or returned to the sponsor or designee.

All accountability records must be made available for inspection by the sponsor and regulatory agency inspectors; photocopies must be provided to the sponsor at the conclusion of the study.

### 7.5.4. Treatment Compliance

All drug compliance records must be kept current and made available for inspection by the sponsor and regulatory agency inspectors.

## 7.6. Concomitant Medications and Procedures

During the treatment period, patients should not receive any treatment of their malignancy other than odronextamab. Any other medication, which is considered necessary for the patient's welfare and which is not expected to interfere with the evaluation of the study drug, may be given at the discretion of the investigator.

All treatments administered during the study (from the time informed consent is obtained until 90 days following the last dose or start of non-protocol anti-lymphoma therapy, whichever occurs first) are regarded as concomitant treatments. All concomitant medications, including the generic name, dose, dose unit, frequency, and start/stop date must be recorded in the study case report

form (CRF). Any treatments that are started after 90 after the last dose of study treatment, or start of non-protocol anti-lymphoma therapy, whichever occurs first, should not be recorded unless the medication is started to treat SAEs deemed related to study drug.

Transient release of cytokines has been observed/expected predominantly during initial weeks of dosing. Cytokines may suppress CYP450 enzymes (Morgan, 2001). For patients who are receiving concomitant CYP450 substrates, particularly those with a narrow therapeutic index, monitoring of the effect (eg, warfarin) or drug concentration (eg, cyclosporine or theophylline) is recommended especially during the initial weeks of therapy.

### 7.6.1. Prohibited Medications

While participating in this study, a patient may not receive any of the following during the period defined for concomitant treatments in Section 7.6, unless otherwise specified below:

- Corticosteroids: Ongoing systemic treatment of more than 10 mg per day of prednisone or anti-inflammatory equivalent, except as specified in the protocol or for treatment of an AE
- Live vaccination: Patients should not receive live vaccination within 28 days of the first administration of odronextamab. In addition, no live vaccination should be administered during the odronextamab treatment period until at least 12 weeks after the last dose of odronextamab, or until recovery of B-lymphocytes to normal ranges following the last treatment, whichever is later. The treating physician should take into consideration the recovery of B-lymphocyte number and function according to the local institutional guidelines for vaccination of immunocompromised patients.
  - No other anti-lymphoma therapies (including chemotherapy, radiation, immune therapy, other experimental therapies) are permitted while patient is receiving study treatment.

### 7.6.2. Permitted Medications and Procedures

The following medications and procedures will be permitted in the following circumstances:

- Any medication required to treat an AE, including systemic corticosteroids
- Corticosteroid therapy for patients requiring physiologic replacement therapy, or requiring a brief course for prophylaxis (eg, for contrast dye allergy) or for treatment of non-autoimmune conditions (eg, delayed-type hypersensitivity reaction caused by contact allergen).
- Granulocyte-colony stimulating factor (G-CSF), platelet, or red blood cell transfusions are permitted for patients who require hematopoietic support.

  A patient may not have received G-CSF within 2 days prior to first dose of odronextamab and platelet transfusion within 7 days prior to first dose of odronextamab in order to meet the ANC and platelet eligibility criteria, respectively.
- COVID-19 Vaccination: It is recommended for patients to complete COVID-19 vaccination (initial series or booster) at least 1 week prior to receiving the first dose of odronextamab. If a patient intends to receive COVID-19 vaccine (initial series or

booster) after starting study treatment, it is also recommended to delay COVID-19 vaccination until patients have received the initial gradual step-up dosing and have received and tolerated the recommended QW full dose of study treatment. Live vaccines remain prohibited as noted above.

- Any other medication, which is considered necessary for the patient's welfare and which is not expected to interfere with the evaluation of the study drug, may be administered at the discretion of the investigator.

# 8. STUDY SCHEDULE OF EVENTS AND PROCEDURES

## 8.1. Schedule of Events

In light of the public health emergency related to COVID-19, the continuity of clinical study conduct and oversight may require implementation of temporary or alternative mechanisms. Examples of such mechanisms may include, but are not limited to, any of the following: phone contact, virtual visits, telemedicine visits, online meetings, non-invasive remote monitoring devices, use of local clinic or laboratory locations, and home visits by skilled staff. Additionally, no waivers to deviate from protocol enrollment criteria due to COVID-19 will be granted. All temporary mechanisms utilized, and deviations from planned study procedures, in response to COVID-19, are to be documented as being related to COVID-19 and will remain in effect only for the duration of the public health emergency.

Study assessments and procedures are presented by study period and visit in Table 10 and Table 11.

## Table 10: Schedule of Events

| | Prior To Treatment | Treatment Period | | | | | | | | | Follow-Up Period [3] | | | | Survival |
|---|---|---|---|---|---|---|---|---|---|---|---|---|---|---|---|
| | Screening[2] | QW Odronextamab Dosing | | | | | | | | Q2W Odronextamab Dosing | Safety Follow-up Visit 1 | Safety Follow-up Visit 2 | Safety Follow-up Visit 3 | Extended Follow-up (12 weeks after S-FU Visit 3 and Q12W thereafter) | Every 12 weeks post end of study |
| | | W1, W2, W3[29] | | | W4 | | | W5-11 | W12 | W14 until disease progression[30] | | | | | |
| **Study Procedure** | | | | | | | | | | | | | | | |
| **Visit Day[1]** | **Day -28 to 1** | **D 1** | **D 2** | **D 3** | **D 1** | **D 2** | **D 3** | **D1** | **D1** | **D1** | | | | | |
| **Visit Window (± day)** | | ±2 | ±2 | ±2 | ±2 | ±2 | ±2 | ±2 | ±2 | ±2 | ±7 | ±7 | ±7 | ±7 | |
| **Screening/Baseline** | | | | | | | | | | | | | | | |
| Informed consent[4] | X | | | | | | | | | | | | | | |
| Inclusion/exclusion criteria | X | | | | | | | | | | | | | | |
| Medical/oncology History | X | | | | | | | | | | | | | | |
| Demographics | X | | | | | | | | | | | | | | |
| Central histopathology review (FL grade 1-3a only) | X | | | | | | | | | | | | | | |
| Complete physical exam[5] | X | | | | | | | | | | | | | | |
| Brain MRI (or CT, if MRI not feasible and approved by sponsor) | X (+7-day window) | Performed per standard-of-care | | | | | | | | | | | | | |
| **Hospitalization** | | | | | | | | | | | | | | | |
| Inpatient Observation[6] | | X | X | | X | | | | | | | | | | |
| **Treatment** | | | | | | | | | | | | | | | |
| Odronextamab administration[6] | | X | X | | X | | | X | X | X | | | | | |
| **Efficacy** | | | | | | | | | | | | | | | |
| Radiographic disease assessment: CT or MRI [7] | X (+7-day window) | | | | | | | | X[7] | X[7] | | | X | Q12W at E-FU 1, 2, 3; Q24W thereafter | |
| FDG-PET [7] | X (+7-day window) | | | | | | | | X[7] | X[7] | Annually | | | | |
| BMA/BMBx [8] | X | | | | | | | | X | X (Then per standard of care to confirm CR/ suspected progression) | | | | | |
| Survival monitoring[3] | | X | | | | | | | | | | | | | |
| **Safety** | | | | | | | | | | | | | | | |
| Vital Signs[9] | X | X | X | | X | | | X | X | X | X | X | X | X | |

| | Prior To Treatment | Treatment Period | | | | | | | | | Follow-Up Period [3] | | | | Survival |
|---|---|---|---|---|---|---|---|---|---|---|---|---|---|---|---|
| | Screening[2] | QW Odronextamab Dosing | | | | | | | | Q2W Odronextamab Dosing | Safety Follow-up Visit 1 | Safety Follow-up Visit 2 | Safety Follow-up Visit 3 | Extended Follow-up (12 weeks after S-FU Visit 3 and Q12W thereafter) | Every 12 weeks post end of study |
| | | W1, W2, W3[(29)] | | | W4 | | | W5-11 | W12 | W14 until disease progression[30] | | | | | |
| **Study Procedure** | | | | | | | | | | | | | | | |
| **Visit Day[1]** | **Day -28 to 1** | **D 1** | **D 2** | **D 3** | **D 1** | **D 2** | **D 3** | **D1** | **D1** | **D1** | | | | | |
| **Visit Window (± day)** | | ±2 | ±2 | ±2 | ±2 | ±2 | ±2 | ±2 | ±2 | ±2 | ±7 | ±7 | ±7 | ±7 | |
| Height [10] | X | | | | | | | | | | | | | | |
| Weight [10] | X | X (W1D1, and then Q4W i.e. W1, W4, W8, W12 etc) | | | | | | | | | X | X | X | X | |
| Limited physical exam [11] | | X (W1D1, and then Q4W) | | | | | | | | X | X | X | X | X | |
| Lymphatic Exam[5, 11] | X | X (W1D1, and then Q4W) | | | | | | | | X | X | X | X | X | |
| 12-Lead ECG [12] | X | | | | | | | | X | | | | X | | |
| Echo or MUGA [13] | X | | | | | | | | | | | | | | |
| ECOG performance status [14] | X | X (W1D1, and then Q4W) | | | | | | | | X | X | X | X | X | |
| B symptoms [14] | X | X (W1D1, and then Q4W) | | | | | | | | X | X | X | X | X | |
| Adverse events [15] | ONGOING | | | | | | | | | | | | | Only Related SAE | |
| Concomitant medications (CM)/procedures [16] | ONGOING | | | | | | | | | | | | | Only CM/procedures for Related SAE | |
| **Laboratory Tests 33** | | | | | | | | | | | | | | | |
| Hematology [17] | X | X | X | X | X | | | X | X | X | X | X | X | X | |
| Blood chemistry [18] | X | X | X | X | X | X | | X | X | X | X | X | X | X | |
| C-reactive protein (CRP) [19] | | X | X | X | X | X | | | | X (W14) | | | | | |
| Ferritin [19] | | X | X | X | X | X | | | | X (W14) | | | | | |
| Pregnancy Test [20] | X | X (W1D1, and then Q4W) | | | | | | | | X (W14 Q4W) | X | | X | X | |
| Urinalysis [21] | X | X (W1D1, and then Q4W) | | | | | | | | X | X | X | X | | |
| PT (INR) /PTT or aPTT [17] | X | | | | | | | | | | | | | | |
| Serum IgG[31] | X | | | | | | | | X | X (W24 then Q24W) | | | X | X (Q24W after S-FU 3) | |
| HIV, HCV, HBV [22] | X | | | | | | | | | | | | | | |
| CMV PCR testing[34] | X | | | | | | | X(W6) | X | X (W24) | | | | | |

| | Prior To Treatment | Treatment Period | | | | | | | | | Follow-Up Period [3] | | | | Survival |
|---|---|---|---|---|---|---|---|---|---|---|---|---|---|---|---|
| | Screening[2] | QW Odronextamab Dosing | | | | | | | | Q2W Odronextamab Dosing | Safety Follow-up Visit 1 | Safety Follow-up Visit 2 | Safety Follow-up Visit 3 | Extended Follow-up (12 weeks after S-FU Visit 3 and Q12W thereafter) | Every 12 weeks post end of study |
| | | W1, W2, W3[(29)] | | | W4 | | | W5-11 | W12 | W14 until disease progression[30] | | | | | |
| **Study Procedure** | | | | | | | | | | | | | | | |
| **Visit Day[1]** | **Day -28 to 1** | **D 1** | **D 2** | **D 3** | **D 1** | **D 2** | **D 3** | **D1** | **D1** | **D1** | | | | | |
| **Visit Window (± day)** | | ±2 | ±2 | ±2 | ±2 | ±2 | ±2 | ±2 | ±2 | ±2 | ±7 | ±7 | ±7 | ±7 | |
| **PK and ADA Sampling 34** | | | | | | | | | | | | | | | |
| PK Sample [19] | | X | X | X | X | X | | X | X | X (W14, 18, 24, 36, then Q8W thereafter) | X | X | X | | |
| Anti-drug antibody (ADA sample) [23] | | X | | | | | | | X | X (W36 then Q24W) | X | | X | | |
| **External Lab Samples [33]** | | | | | | | | | | | | | | | |
| Cytokine profiling (serum) [19] | | X | X | X | X | X | | | | X (W14) | | | | | |
| PBMC immunophenotyping [19, 26] | | X | | | X | | | X (W5 only) | | X (W14) | | | | X (24 weeks following last dose) | |
| Lymphocyte immunophenotyping (whole blood) [19, 25] | | X | | | X | | | X (W5 only) | | X (W14) | | | | X (24 weeks following last dose) | |
| Blood collection – genomic DNA (optional) [24] | | X | | | | | | | | | | | | | |
| MRD-Whole blood (tumor DNA) [27] | | X | | | | | | | X[7] | X[7] | | | X | E-FU 1, 2, 3; Q24W thereafter | |
| MRD-Plasma ctDNA[27] | | X | | | | | | | X[7] | X[7] | | | X | E-FU 1, 2, 3; Q24W thereafter | |
| **Pathology Sample** | | | | | | | | | | | | | | | |
| Lymph node/tumor biopsy [28] | X | | | | | | | X (W6) | | Per standard-of-care and at time of progression | | | | | |
| **Quality of Life Assessment** | | | | | | | | | | | | | | | |
| EORTC-QLQ-C30[32] | X | X | | | X | | | X (W10) | | X (Q8W in first year, Q12W in 2nd year) | | | X | X (Q24W in 3rd and 4th year) | |

| | Prior To Treatment | Treatment Period | | | | | | | | | Follow-Up Period [3] | | | | Survival |
|---|---|---|---|---|---|---|---|---|---|---|---|---|---|---|---|
| | Screening[2] | QW Odronextamab Dosing | | | | | | | | Q2W Odronextamab Dosing | Safety Follow-up Visit 1 | Safety Follow-up Visit 2 | Safety Follow-up Visit 3 | Extended Follow-up (12 weeks after S-FU Visit 3 and Q12W thereafter) | Every 12 weeks post end of study |
| | | W1, W2, W3[(29)] | | | W4 | | | W5-11 | W12 | W14 until disease progression[30] | | | | | |
| **Study Procedure** | | | | | | | | | | | | | | | |
| **Visit Day[1]** | **Day -28 to 1** | **D 1** | **D 2** | **D 3** | **D 1** | **D 2** | **D 3** | **D1** | **D1** | **D1** | | | | | |
| **Visit Window (± day)** | | ±2 | ±2 | ±2 | ±2 | ±2 | ±2 | ±2 | ±2 | ±2 | ±7 | ±7 | ±7 | ±7 | |
| EQ5D-3L[32] | X | X | | | X | | | X (W10) | | X (Q8W in first year, Q12W in 2nd year) | | | X | X (Q24W in 3rd and 4th year) | |
| FACT-Lym[32] | X | X | | | X | | | X (W10) | | X (Q8W in first year, Q12W in 2nd year | | | X | X (Q24W in 3rd and 4th year) | |

ADA = anti-drug antibody; aPTT = activated partial thromboplastin time; BMA = bone marrow aspirate; BMBx = bone marrow biopsy; CRP = C-reactive protein; CT = computed tomography; Echo = echocardiogram; EORTC-QLQ-C30 = European Organisation for Research and Treatment of Cancer Quality of Life Questionnaire ; EQ-5D-3L = EuroQoL 5 Dimensions 3 Levels; E-FU= Extended Follow-up Visit; FACT-Lym = Functional Assessment of Cancer Therapy–Lymphoma; FDG = 2-deoxy-2[F-18]fluoro-D-glucose; HBV = hepatitis B virus; HCV = hepatitis C virus; HIV = human immunodeficiency syndrome; IgG = immunoglobulin; INR = international normalized ratio; MRI = magnetic resonance imaging; MUGA = multigated acquisition scan; PD = progressive disease; PET = positron emission tomography; PK = pharmacokinetic; QW = Once every week; Q2W = Once every 2 weeks; Q4W = Once every 4 weeks; Q8W = Once every 8 weeks; Q12W = Once every 12 weeks; Q24W = Once every 24 weeks; S-FU= Safety Follow-up Visit.

Standard of care - If at any time assessment is performed because either clinically indicated or as part of standard of care, collection of that information in the CRF is requested; **NOTE:** In the event a protocol specific laboratory test is not routinely performed at a center, and instead, the center performs a different but equivalent test (eg, $CO_2$ instead of bicarbonate; urea instead of blood urea nitrogen, urine urobilinogen instead of urine bilirubin, or creatine phosphokinase instead of creatine kinase ), the equivalent test should be performed and results collected. At centers where the bicarbonate (or equivalent) test is not performed as part of the routine chemistry panel, the test may be omitted.

### 8.1.1. Footnotes for the Schedule of Events Table

1. Clinical Assessments consist of physical exam, weight, ECOG, assessment for B symptoms, hematology, chemistry, urinalysis, and pregnancy test.

   *Study visit and assessment windows:* The initial QW odronextamab dosing and Q2W dosing, visits may occur within a ±2-day window. No 2 treatment visits (not including the split dose visits) may be less than 5 days apart. During the post-treatment follow-up period, follow-up study visits/phone calls may occur within a ±7-day window.

   *Clinical Assessments*: Clinical assessments required prior to the first study drug administration may be performed up to 3 days before the visit unless otherwise specified. Clinical assessments during treatment may be performed within 24 hours prior to study drug administration.

2. *Screening window:* For laboratory abnormalities or other clinical parameters that are clearly reversible, the screening window may be extended an additional 7 days to allow for correction and retesting of the specific parameter(s) that do not meet entry criteria. The additional 7-day window for screening period will also be allowed for imaging and central histopathology confirmation, if this is required for administrative feasibility. Such a 7-day extension may be granted only after discussion of the circumstances and rationale by the Investigator and the sponsor. If the screening clinical assessments are performed within 72 hours of week 1 day 1, the week 1 day 1 assessment does not need to be repeated.

3. Follow-up period consists of:

   a. Safety follow-up which starts after the last dose of odronextamab with the first follow-up being the Safety Follow-up Visit 1 at 4 weeks following the last dose, Safety Follow-up Visit 2 at 8 weeks following the last dose, and Safety Follow-up Visit 3 at 12 weeks following the last dose, or after the start of a non-protocol anti-lymphoma therapy or patient withdrawal of consent, whichever is sooner.

   b. Extended follow-up which starts after the Safety Follow-up Visit 3 only for patients who have discontinued study drug for any reason other than disease progression, start of non-protocol anti-lymphoma therapy, withdrawal of consent, or death, disease response will be assessed until the time of disease progression, death, start of a non-protocol anti-lymphoma therapy, or patient withdrawal of consent for follow-up of disease status, whichever is sooner.

   c. After safety follow-up and, if applicable extended follow-up, all study patients will remain to be followed Q12W for survival until death, loss to follow-up, patient withdrawal of consent for follow-up, or study termination by the sponsor, whichever is earlier. Survival follow-up status may be determined at clinic visits or remotely. Survival monitoring is conducted as described in Section 8.2.6.

4. For patients who were newly enrolled or continued on study treatment at the time of Amendment 1 Global, re-treatment is not applicable. For patients who were off treatment at the time when Amendment 1 Global went into effect, resumption of treatment may be considered. The study includes an option for patients to consent to participate in the genomics component of the study. Prior to resumption of treatment, patients will be required to re-sign the main ICF and meet eligibility criteria for resumption of treatment.

5. A complete physical examination (PE) is required only at screening and includes a lymphatic examination. Thereafter a limited PE and a lymphatic examination may be performed at subsequent visits (Section 8.2.3.3). If the physical exam is performed within 72 hours prior to week 1 day 1, the assessment does not have to be repeated on week 1 day 1.

6. For the initial treatment during week 1 up to and including the first QW full dose administered as a single infusion or until the week 4 treatment, whichever is later , study treatment will be in an inpatient hospital setting, refer to Section 8.2.3.1 for further details. Patients who are considered to be at intermediate or high risk for TLS by the investigator must be hospitalized for study drug administration and monitored in an inpatient setting.

7. *Computed tomography/Magnetic Resonance Imaging*: Diagnostic quality CT with contrast (unless contraindicated) or MRI is required during screening, week 12 (±7 days), then approximately Q8W up to week 52 (±7 days) in first year, Q12W up to week 100 (±7 days) in second year, at safety follow-up visit 3, Q12W at Extended Follow-up visits 1, 2, and 3, and Q24W (±14 days) thereafter, until progression, start of non-protocol anti-lymphoma therapy or at any time when disease progression is suspected.

   *Fluorodeoxyglucose positron emission tomography*: FDG-PET will be performed at screening. If positive at screening FDG-PET will be performed at week 12 (±7 days), week 20 (±7 days) (if CR is not observed at week 12), and week 36 (±7 days) in year 1. FDG-PET will then be performed once each at the end of years 2 and annually thereafter at a time point that coincides with the planned CT or MRI tumor assessment (Section 8.2.2.1). FDG-PET must be repeated in patients with FDG-avid disease to confirm response. Any suspected CR should be confirmed by FDG-PET scan. Unscheduled FDG-PET scans are allowed based on observed clinical progression by CT. Unscheduled FDG-PET data, including imaging, if performed per standard of care, will be collected.

   If the patient discontinued treatment due to reasons other than clinical or radiologic PD, disease assessments will continue to be performed until PD according to the follow-up period in the protocol.

   **NOTE:** Tumor assessment scans will be performed on schedule even if there is interruption in treatment and may not align with treatment weeks

   Procedures for CT and FDG-PET scans are in Section 8.2.2.1.

8. Bone marrow aspirate (BMA) and/or bone marrow biopsy (BMBx) will be performed during screening, week 12 (±7 days), if patient has a CR to confirm response; and if progression is suspected (see Section 8.2.2.3). All bone marrow evaluations after screening will be required only if evidence of bone marrow disease is present at baseline (evidence of bone marrow disease may be by conventional pathology or by assessment of MRD), and in such instances a bone marrow evaluation may be performed at any time at the discretion of the investigator. For the bone marrow aspirate (BMA) the first research sample will be collected for DNA isolation and the second research sample for RNA isolation, as detailed in the laboratory manual.

9. Vital signs assessed are noted in Section 8.2.3.1. Vital signs assessment time points for the first 4 weeks and until the full QW dose is given as a single infusion after screening are: week 1 day 1/day 2, week 2 day 1/day 2, week 3 day 1/day 2 and week 4 day 1 (day 2 only applicable if split dose administered). Vital signs will be assessed and documented prior to the infusion, every 30 minutes (±15 mins) for the first 4 hours until end of infusion (EOI), and then hourly (±15 mins) until 4 hours following the EOI. For subsequent doses, vital signs will be assessed and documented prior to the infusion, every 30 minutes (±15 mins) for the first 1 hour from start of infusion, and then hourly (±15 mins) until EOI when applicable. During study visits where no study drug is administered, vital signs are only required once at any time during the visit and pulse oximetry is not required on these days. When scheduled at the same time as other procedures, vital signs should be measured prior to clinical laboratory assessments, PK, or exploratory sample collection. If a patient experiences CRS of any grade, then monitor vital signs every hour ±30 minutes until resolution of clinical symptoms.
10. Height is collected only once during screening. Weight is collected during screening, week 1 day 1, and then every 4 weeks during treatment period (ie, on week 4, week 8, week 12, etc); at the Safety Follow-up Visits 1, 2, and 3; and during extended follow-up visits for patients who had discontinued treatment for reasons other than progression.
11. Lymphatic exam as well as limited PE, which includes examination of the lungs, heart, abdomen, and skin, is performed as described in Section 8.2.3.3 from week 1 day 1 and then Q4W during treatment period (ie, on week 4, week 8, week 12, etc) and at the Safety Follow-up Visits 1, 2, 3 and during extended follow-up visits for patients who had discontinued treatment for reasons other than progression.
12. 12-lead ECG will be performed at screening, week 12 and at the safety follow-up visit 3 or prior to commencing non-protocol anti-lymphoma therapy, and it may be performed at any other time if clinically indicated (Section 8.2.3.6). Electrocardiograms should be performed before blood is drawn during visits requiring blood draws.
13. Ejection fraction is measured by echocardiogram (Echo) or multigated acquisition scan (MUGA) (see Section 8.2.3.6)
14. B symptom and ECOG performance status (Appendix 1) are assessed during screening, week 1 day 1, and Q4W during treatment period (ie, on week 4, week 8, week 12, etc); at the Safety Follow-up visits 1, 2, and 3; and during extended follow-up visits for patients who had discontinued treatment for reasons other than progression, as detailed in Section 8.2.3.4 and Section 8.2.3.5.
15. Adverse events and SAEs should be collected throughout the study (see Section 9.4). Additionally, any SAE that the investigator believes may be related to study treatment and that occurs after 90 days following the last dose or after the start of non-protocol anti-lymphoma therapy, whichever occurs first should be reported.
16. Concomitant medications and procedures recorded from date of ICF up until 90 days following the last dose or until the start of non-protocol anti-lymphoma therapy, whichever occurs first. After 90 days following the last dose or after the start of non-protocol anti-lymphoma therapy, whichever occurs first, any treatments that are started to treat SAEs deemed related to study drug should also be recorded.

17. Hematology, PT (INR) and PTT or aPTT testing are detailed in Section 8.2.3.7.
18. Chemistry tests to be performed are detailed in Section 8.2.3.7. Creatinine clearance will be recorded based on serum creatinine by Cockcroft-Gault or from 24-hour urine collection. For tumor lysis syndrome laboratory tests refer to Section 7.3.5.2.
19. See Table 11 for detailed schedule of events for the acquisition of samples for PK, cytokine profiling, lymphocyte immunophenotyping (whole blood), and PBMC immunophenotyping, ferritin, and CRP. All analyses for CRP and ferritin will be performed locally.
20. Pregnancy test only in women of childbearing potential; a serum ß-HCG must be obtained at screening and within 72 hours of first administration of odronextamab. After screening, subsequent pregnancy tests may be performed on urine samples.
21. Urinalysis testing will be performed as detailed in Section 8.2.3.7.
22. HIV, HBV, and HCV testing will be performed in patients who have unknown serologic status prior to screening or who have not had testing within 12 months of prior to study drug administration. Uncontrolled infection, as described in Section 6.2.2 exclusion criterion 12, will render a patient ineligible for enrollment in the study.
23. ADA samples will be collected prior to the first dose at week 1 day 1 and predose at weeks 12, 36 and Q24W thereafter. This is applicable for both Q2W and Q4W dose regimens. During the safety follow-up, ADA samples will be collected at Safety Follow-up Visits 1 and 3. Should a treatment-emergent or treatment-boosted ADA positivity be detected in the first post dose sample collected at week 12 day 1, the PK sample collected at week 4 day1-predose will be tested for anti-odronextamab antibodies.
24. A single blood sample for genomic DNA (optional) will be collected pre-dose at week 1 day 1. If the sample collection is missed at that time point, 1 sample will be collected at any other study visit (further details are provided in Section 8.2.8.5). Only 1 sample is requested.
25. Lymphocyte immunophenotyping (whole blood) sample will be collected in all cohorts at day 1 of weeks 1, 2, 3, 4, 5, and 14 prior to study drug administration and at 24 weeks after last dose.
26. PBMC immunophenotyping sample will be collected in all cohorts at day 1 of weeks 1, 2, 3, 4, 5, and 14 prior to study drug administration and at 24 weeks after last dose.
27. For the determination of minimal residual disease, whole blood (tumor DNA) and Plasma ctDNA samples are obtained at the following time points: prior to study drug administration at baseline (week 1 day 1), at week 12, and, if the patient achieves CR, at every time point that MRI/CT is performed (as described in footnote 7).
28. Lymph node and/or tumor biopsy (performed as described in Section 8.2.2.4) is required before administration of the first odronextamab dose. It may be performed at week 6 (±7 days) or after patient receives 2 full doses (whichever is later) and with PD if, in the opinion of the investigator, the patient has an accessible lesion and the sample can be obtained without significant risk to the patient. If pre-treatment biopsy is not feasible, an archival sample should be submitted. Additional tumor biopsies may be performed if

clinically indicated (eg, tumor swelling after administration of odronextamab) or as part of standard of care, and a sample will be sent to central labs.

29. If a patient does not experience grade 3 CRS prior to treatment week 4 (when the full QW dose is to be administered), at treatment week 4 and beyond, odronextamab will be administered as a single infusion. However, if a patient experiences grade 3 CRS prior to treatment week 4, then odronextamab will be administered as a split infusion over 2 days (see Section 7.3.3 for details). For those patients receiving a single infusion at treatment week 4, the week 4 day 2 visit will not be conducted per table above. Study procedures noted for week 4 day 3 and day 4 will be obtained approximately 24 hours and 48 hours respectively, after the week 4 single infusion.
30. If patient has demonstrated a CR and has shown a durable response for at least 9 months after initial determination of CR, then the frequency of study drug administration at the assigned dose will be decreased from Q2W to Q4W intervals, based on local investigator evaluation. Patients must have received the assigned QW full dose at the Q2W dosing schedule for at least 3 preceding doses before switching from Q2W to Q4W dosing. Treatment will continue until disease progression or other protocol-defined reason for treatment discontinuation.

    NOTE: Patients who are receiving a lower dose than the protocol-specified Q2W dose can switch to Q4W dosing after 9 months of maintained CR, after discussion and approval from sponsor.
31. Serum IgG will be assessed at baseline, week 12, week 24, and Q24W thereafter during study treatment, Safety Follow-up Visit 3 and Q24W thereafter in follow-up.
32. Patient reported outcomes EORTC QLQ-C30, EQ5D-3L, and FACTLym will be administered prior to study drug administration and study procedures. These assessments should be collected on dosing visits when a patient moves to Q4W dosing.
33. Additional lab samples, with the exception of the blood genomic DNA (optional) sample, may be collected at any time to further investigate safety, after discussion between the investigator and the medical monitor.
34. CMV PCR testing in blood will be performed during screening, week 6 (±1 week), week 12 (±1 week), week 24 (±2 weeks), and at other time points when clinically indicated (see Section 7.3.4).

### 8.1.2. Changes to the Schedule of Events/Study Procedures in Case of Dose Modification:

General guidance: Treatment Weeks are numbered continuously and align with any dose administered, regardless of whether the actual dose was according to the per-protocol dosing regimen or from a dose modification.

For scenarios where the dosing administration differs from per-protocol dosing regimen

- For initial dose, intermediate dose-1, intermediate dose-2, and the first full QW dose sites should follow week 1, week 2, week 3, and week 4 respectively for inpatient hospitalization, safety monitoring, and laboratory tests.



VV-RIM-00192456-1.0 Approved - 11 Apr 2022 GMT-5:00

- Inpatient hospitalization, safety monitoring, and laboratory tests also apply to any modified doses during the step-up dosing

If any ***of the per-protocol specified*** initial dose, intermediate dose-1, intermediate dose-2 were repeated or first full QW dose administered at a delayed treatment week timepoint, the below study procedures should remain on the treatment week schedule.

- limited physical exam
- lymphatic exam
- 12-lead ECG
- ECOG
- B symptoms
- pregnancy test
- urinalysis
- serum IgG

- For PK and Biomarker samples: Sample collection during the visits where the initial dose, intermediate dose-1, intermediate dose-2, and the first full QW dose are administered for the first time (this includes when the patient received only first split in prior week and had to repeat the dose again)
  - PK and biomarker blood samples: Will be collected according to the treatment week schedule of events that correspond to the particular step-up dose

- Sample collection during the visits where the initial dose, intermediate dose-1, intermediate dose-2, and the first full QW dose are repeated (this is when the patient received both split doses in prior week [and had the samples collected] and had to repeat the dose again in the current week due to any reason)
  - PK samples: For any repeated doses, only the pre-dose and EOI PK samples will be collected.
  - Biomarker blood samples: For any repeated dose, samples will be collected again according to the treatment week schedule of events that correspond to the particular step-up dose.

**Table 11: Schedule of Events Detailed Sample Collection Time Points for Cytokines, Pharmacokinetics, Lymphocyte Immunophenotyping (Whole Blood), PBMC Immunophenotyping, C-Reactive Protein, and Ferritin**

| | | Central lab | | | | | Local lab |
|---|---|---|---|---|---|---|---|
| Visit | Time Point | Cytokines [1,2,8] | PK [1,2,3,8] | ADA[1,2,7,8] | Lymphocyte immunophenotyping (whole blood)[4,8] | PBMC immunophenotyping[4,8] | CRP, Ferritin [2, 8] |
| Screening | | | | | | | |
| Treatment Period QW Odronextamab Dosing | | | | | | | |
| Week 1 Day 1 | Pre-dose | X | X | X | X | X | X |
| | EOI | X | X | | | | X |
| | T2H | X | X | | | | X |
| Week 1 Day 2 | Pre-dose | X | X | | | | X |
| | EOI | X | X | | | | X |
| | T2H | | X | | | | |
| Week 1 Day 3 | T24H | X | X | | | | X |
| Week 2 Day 1 | Pre-dose | X | X | | X | X | X |
| | EOI | X | X | | | | X |
| | T2H | X | | | | | X |
| Week 2 Day 2 | Pre-dose | X | X | | | | X |
| | EOI | X | X | | | | X |
| | T2H | | X | | | | |
| Week 2 Day 3 | T24H | X | X | | | | X |
| Week 3 Day 1 | Pre-dose | X | X | | X | X | X |
| | EOI | X | X | | | | X |
| | T2H | X | | | | | X |
| Week 3 Day 2 | Pre-dose | X | X | | | | X |
| | EOI | X | X | | | | X |
| | T2H | | X | | | | |
| Week 3 Day 3 | T24H | X | X | | | | X |

| Visit | Time Point | Central lab | | | | | Local lab |
|---|---|---|---|---|---|---|---|
| | | Cytokines [1,2,8] | PK [1,2,3,8] | ADA[1,2,7,8] | Lymphocyte immunophenotyping (whole blood)[4,8] | PBMC immunophenotyping[4,8] | CRP, Ferritin [2, 8] |
| **Week 4 Day 1** | Pre-dose | X | X | | X | X | X |
| | EOI | X | X | | | | X |
| **Week 4 Day 2** | T24H | X | X | | | | X |
| **Week 5 Day 1** | Pre-dose | | X | | X | X | |
| | EOI | | X | | | | |
| **Week 8 Day 1** | Pre-dose | | X | | | | |
| | EOI | | X | | | | |
| **Week 12 Day 1** | Pre-dose | | X | X | | | |
| | EOI | | X | | | | |
| **Treatment Period Q2W Odronextamab Dosing[5]** | | | | | | | |
| **Week 14 Day 1** | Pre-dose | X | X | | X | X | X |
| | EOI | X | X | | | | X |
| **Week 16 Day 1** | Pre-dose | | | | | | |
| | EOI | | | | | | |
| **Week 18 Day1** | Pre-dose | | X | | | | |
| | EOI | | X | | | | |
| **Week 20 Day1** | Pre-dose | | | | | | |
| | EOI | | | | | | |
| **Week 24 Day 1** | Pre-dose | | X | | | | |
| | EOI | | X | | | | |
| **Week 36 Day 1[6]** | Pre-dose | | X | X[7] | | | |
| | EOI | | X | | | | |
| **After 36 weeks[6]** | Pre-dose | | X[6] | | | | |
| | EOI | | X[6] | | | | |
| **Follow-up Period** | | | | | | | |
| **Safety Follow-up Visit 1 (4 weeks following last dose)** | | | X | X | | | |



VV-RIM-00192456-1.0 Approved - 11 Apr 2022 GMT-5:00

| Visit | Time Point | Central lab | | | | | Local lab |
|---|---|---|---|---|---|---|---|
| | | Cytokines [1,2,8] | PK [1,2,3,8] | ADA[1,2,7,8] | Lymphocyte immunophenotyping (whole blood)[4,8] | PBMC immunophenotyping[4,8] | CRP, Ferritin [2,8] |
| **Safety Follow-up Visit 2 (8 weeks following last dose)** | | | X | | | | |
| **Safety Follow-up Visit 3 (12 weeks following last dose)** | | | X | X | | | |
| **Extended Follow-Up 1** | | | | | X (24 weeks following last dose) | X (24 weeks following last dose) | |

CRP = C-reactive protein; EOI = End of infusion; PK = pharmacokinetic; T2H = 2 hours after EOI; T24H = 24 hours after EOI.

### 8.1.3. Footnotes for the Schedule of Events Detailed Sample Collection Time Points for Cytokines, Pharmacokinetics, Lymphocyte Immunophenotyping (Whole Blood), PBMC Immunophenotyping, C-Reactive Protein, and Ferritin

1. Regardless of infusion duration, the end of infusion (EOI) is the time when all the study drug present in the IV infusion bag and the IV line has been infused.
2. Sample collection instruction: pre-dose (within 2 hours prior to starting study drug infusion), EOI (±5 min), T2H (±30 min), and 24 hours (±2 hours) after the EOI. The actual sampling time at each time point must be recorded.
3. PK samples should be drawn from the opposite arm rather than from the IV line for study drug administration to ensure accuracy of measured drug concentration in serum and avoid sample contamination. In a special case that samples cannot be drawn from the opposite arm and need to be drawn from the IV line for study drug administration, the IV line needs to be flushed and the first sample collected must be discarded before collecting any PK samples; the case must be documented accordingly.
4. Lymphocyte immunophenotyping (whole blood) and PBMC immunophenotyping samples will be collected in all cohorts at day 1 of weeks 1, 2, 3, 4, 5, and 14 prior to study drug administration and at 24 weeks after last dose.
5. If patient has demonstrated a CR and has shown a durable response for at least 9 months after initial determination of CR, then the frequency of study drug administration at the assigned dose will be decreased from Q2W to Q4W intervals, based on local investigator evaluation. Patients must have received the assigned QW full dose at the Q2W dosing schedule for at least 3 preceding doses before switching from Q2W to Q4W dosing.
6. PK samples will be collected at predose and end of infusion every 8 weeks starting from week 36 for both Q2W and Q4W regimens until the last dose or up to two years of treatment (ie, sampling at weeks 36, 44, 52, 60, 68, 76, 84, 92, 100, and 108). If a patient switches from Q2W to Q4W dosing after two years of treatment, PK samples of predose and end of infusion must be collected for 3 consecutive visits at an 8-week interval from the beginning of Q4W regimen (eg, if the first Q4W dose starts at week X after 2 years of treatment, the PK samples will be collected at weeks X, X+8 and X+16). Sampling time for the safety follow-up visits is scheduled at Safety Follow-up Visits 1, 2, and 3.
7. Anti-odronextamab antibody (ADA) samples may be collected every 24 weeks starting at treatment week 36. This is applicable for both Q2W and Q4W dose regimens. Should a treatment-emergent or treatment-boosted ADA positivity be detected in the first post dose sample collected at week 12 day 1, the PK sample collected at week 4 day 1-predose will be tested for anti-odronextamab antibodies.
8. Additional lab samples may be collected at any time to further investigate safety, after discussion between the investigator and the medical monitor.

### 8.1.4. Early Termination Visit

During the treatment period, patients who discontinue study drug prematurely, but do not withdraw from the study, will be asked to return to the clinic for follow-up visits. Patients will be expected to continue with all relevant study assessments (eg, efficacy assessments and/or safety assessments) per Table 10 and Table 11.

### 8.1.5. Unscheduled Visits

All attempts should be made to keep patients on the study schedule. Unscheduled visits may be necessary to repeat testing following abnormal laboratory results, for follow-up of AEs, or for any other reason, as warranted.

Relevant assessments that occur during unscheduled visits should be reported in the respective CRF for the visit date.

## 8.2. Study Procedures

### 8.2.1. Procedures Performed Only at the Screening/Baseline Visit

The following procedures will be performed for the sole purpose of determining study eligibility or characterizing the baseline population:

- Medical/oncology history
- Demographics
- Complete physical examination (Section 8.2.3.3)
- Height
- Brain MRI or CT: During screening (within 28 days prior to start of study drug administration), a mandatory brain MRI (or CT imaging if MRI is infeasible) will be performed to evaluate for evidence of CNS involvement of malignancy and/or for other findings that might preclude inclusion in the trial according to the protocol eligibility criteria. If brain MRI is infeasible, a CT scan may be substituted if approved by the sponsor.

  If imaging is performed during treatment or follow-up period as part of standard of care, data will also be collected.
- Viral serologies: HIV, HBV, HCV testing (Section 8.2.3.7)
- Echocardiogram or MUGA (cardiac ejection fraction)
- Coagulation parameters: PT (INR) and aPTT or PTT (Section 8.2.3.7)
- For FL grade 1-3a patients only: Central histopathology review: For all patients, a central histopathology review of tissue will be conducted before study enrollment. Eligibility for this study will be confirmed upon establishing a diagnosis of follicular lymphoma grade 1 to 3a.

Rescreening is allowed for patients who failed screening at an earlier time point.



VV-RIM-00192456-1.0 Approved - 11 Apr 2022 GMT-5:00

### 8.2.2. Efficacy Procedures

For all patients, disease will be radiologically evaluated according to the Lugano Classification (Cheson, 2014) for the primary endpoint based on independent central review and for the secondary endpoint as determined by investigator review. The CT or MRI for tumor assessment will be performed as detailed in Table 10.

All radiological imaging will be submitted to a central repository and will be reviewed centrally.

#### 8.2.2.1. Computed Tomography Imaging/Magnetic Resonance Imaging and $^{18}$F-Fluorodeoxyglucose-Positron Emission Tomography

A diagnostic quality (≤5 mm slices) MRI or CT scan with contrast of the neck, chest, abdomen, and pelvis, as well as any other known sites of disease will be performed as detailed in Table 10 and at any time when disease progression is suspected. A mandatory imaging of brain at baseline should be performed as described in Section 8.2.1.

For each patient, the same method of measurements and the same technique must be used to evaluate each lesion throughout the study. If a patient inadvertently misses a prescribed tumor evaluation or a technical error prevents the evaluation, the patient may continue treatment until the next scheduled assessment, unless signs of clinical progression are present. If, at any time during the treatment period, there is suspicion of disease progression based on clinical or laboratory findings (and before the next scheduled assessment), an unscheduled tumor assessment should be performed.

$^{18}$F-fluorodeoxyglucose-positron emission tomography (FDG-PET) scans will also be performed as detailed in Table 10. If FDG-PET is negative at screening, it does not need to be repeated. PET-CT cannot replace a diagnostic quality CT scan unless the CT portion of the PET-CT is of diagnostic quality. More detailed information is provided in the imaging manual.

#### 8.2.2.2. Radiographic Disease Assessment

For each enrolled patient, tumor response at each time point will be evaluated by independent central radiology review according to the Lugano Classification criteria (Cheson, 2014). The primary endpoint of ORR will be evaluated according to the Lugano Classification. Response assessment based on imaging will be reviewed centrally according to an independent review charter. For MZL, overall response assessment will be evaluated based on both 1) CT-only response criteria (without the use of PET-CT) and 2) the combined CT and PET-CT response criteria.

#### 8.2.2.3. Bone Marrow Aspirate and Bone Marrow Biopsy

For all patients, a bone marrow aspirate (BMA) and/or biopsy (BMBx) will be performed as described in Table 10. Additional bone marrow exams should be performed as clinically indicated by the investigator. Samples obtained from BMA/BMBx will be evaluated locally at the site histologically and by additional ancillary studies including immunohistochemistry, flow cytometry, and cytogenetics (eg, *MYC*, *BCL2*, and *BCL6* gene rearrangements). Samples will also be shipped to the central laboratory and will be utilized for additional research testing (Section 8.2.8.2). Detailed instructions for sample collection and preparation are provided in the laboratory flowchart.

Samples will be sent for local analysis (flow cytometry, immunohistochemistry, molecular/cytogenetic analysis) and submitted to the central laboratory, as specified in the laboratory flowchart.

#### 8.2.2.4. Lymph Node and/or Tumor Biopsy

Lymph node/tumor biopsy will be performed at visits specified in Table 10. Samples will be sent for local standard analysis and a sample will also be submitted to the central laboratory, as specified in the laboratory flowchart. Additional tumor biopsies may be performed if clinically indicated (eg, tumor swelling after administration of odronextamab), as part of standard of care and at progression. For these additional biopsies, the collection of local analysis information in the CRF is requested and a sample will also be submitted to the central laboratory. Detailed instructions for sample collection and preparation are provided in the laboratory flowchart.

### 8.2.3. Safety Procedures

#### 8.2.3.1. Safety Monitoring

Post-infusion safety monitoring of patients will include, but will not be limited to, vital signs (with specific attention to temperature and blood pressure), oxygen saturation, and clinical assessments.

For the initial treatment during Week 1 up to and including the first QW full dose administered as a single infusion or until the Week 4 treatment, whichever is later, study treatment will be in an inpatient hospital setting.

The inpatient hospital admission is required for approximately 24 hours (±4 hours) beyond the end of the second split infusion or the end of the single infusion. Inpatient hospital admission for study drug administration and observation at subsequent infusions may be implemented at the discretion of the investigator.

#### 8.2.3.2. Vital Signs

Vital signs including temperature, blood pressure, pulse, pulse oximetry, and respiration rate will be collected at study visits as indicated in Table 10.

Note: blood pressure should be measured after the patient has been resting quietly for at least 5 minutes. Blood pressure measurements should be obtained from a seated or recumbent position and should be performed consistently throughout the study.

#### 8.2.3.3. Physical Examination

A physical examination is to be conducted at study visits as indicated in Table 10. Complete physical examination will include examination of skin, head, eyes, nose, throat, neck, joints, lungs, heart, pulse, abdomen (including liver and spleen), lymph nodes, and extremities, as well as a brief neurologic examination.

Limited physical examination will include lungs, heart, abdomen, and skin.

Care should be taken to examine and assess any abnormalities that have been indicated as potentially present by medical history.

#### 8.2.3.4. Assessment of B Symptoms

Assessment of B symptoms will occur as part of the clinical assessment. B symptoms include:

- Fever (ie, temperature >38°C [>100.4°F]) for 3 consecutive days
- Weight loss exceeding 10% of body weight in 6 months
- Drenching night sweats

#### 8.2.3.5. Performance Status

Performance status will be assessed according to ECOG criteria (Table 10).

#### 8.2.3.6. Electrocardiogram

A standard 12-lead ECG will be performed at time points according to Table 10. The heart rate will be recorded from the ventricular rate, and the PR, QRS, and QT, intervals and QTcF interval will be derived. The ECG tracings will be retained with the source.

#### 8.2.3.7. Laboratory Testing

Samples for laboratory testing will be collected at time points according to Table 10.

- Hematology, blood chemistry, urinalysis, additional clinical laboratory tests (eg. pregnancy testing, serum IgG testing, HIV/HCV/HBV, CMV PCR (can be performed at central laboratory if local testing is not possible), INR/PTT or aPTT, CRP and Ferritin) will be analyzed by local laboratory.
- PK and ADA samples, serum cytokine profiling, whole blood immune monitoring (lymphocyte immunophenotyping and MRD samples), PBMC immunophenotyping, blood collection-genomic DNA (optional), whole blood (tumor DNA), plasma ctDNA, will be analyzed by a central laboratory.
- Lymph node/tumor biopsy samples and BMA/BMBx samples will be sent for analysis to both the local laboratory and the central laboratory.

Tests will include:

***Blood Chemistry***

| | | |
|---|---|---|
| Sodium | Phosphorus | Lactate dehydrogenase (LDH) |
| Potassium | Creatinine | Uric Acid |
| Calcium | Blood urea nitrogen (BUN) | Chloride |
| Glucose (non-fasting) | Aspartate aminotransferase (AST) | Bicarbonate |
| Albumin | Alanine aminotransferase (ALT) | Triglycerides (non-fasting) |
| Total and direct bilirubin | Gamma-glutamyl transferase (GGT) | Creatine kinase (CK) |
| Alkaline phosphatase | Total protein, serum | |



VV-RIM-00192456-1.0 Approved - 11 Apr 2022 GMT-5:00

### *Hematology*

| | |
|---|---|
| Hemoglobin | Differential: |
| Red blood cells (RBCs) | Neutrophils |
| White blood cells (WBCs) | Lymphocytes |
| Platelet count | Monocytes |
| | Basophils |
| | Eosinophils |
| | Other forms |

### *Urinalysis*

| | | |
|---|---|---|
| pH | Bilirubin | Bacteria |
| Specific gravity | Leukocyte esterase | Epithelial cells |
| Ketones | Nitrite | Crystals |
| Protein | White blood cells (WBC) | Yeast and other forms |
| Glucose | Hyaline and other casts | Red blood cells (RBC) |
| Blood | | |

### *Additional Clinical Laboratory Tests*

Pregnancy test: Serum β-HCG will be measured at screening (≤72 hours prior to first dose of study drug administration) and urine or serum β-HCG will be measured at all other time points.

Serum Calcium Corrected for Albumin (derived): Levels of unbound/free calcium (ie, biologically active calcium) will be calculated by adjusting for abnormal albumin levels ("corrected serum calcium").

Creatinine clearance (derived): Calculated based on serum creatinine by Cockcroft Gault formula or from 24-hour urine collection

Coagulation: Assessed by activated partial thromboplastin time (aPTT) or partial thromboplastin time (PTT) and PT (International Normalized Ratio [INR]).

Immunoglobulin (IgG): Serum IgG will be evaluated according to Table 10.

HIV, HCV, and HBV Testing: HIV, HBV, and HCV testing will be performed in patients who have unknown serologic status prior to screening or who have not had testing within 12 months of first study drug administration. The presence of positive test results for HIV, hepatitis B, HB surface antigen [HBsAg], total core HB antibody [anti-HB-c]), or hepatitis C virus (HCV antibody serology testing) will need evaluation by molecular methodologies to assess viral load (refer to Section 6.2.2).

CMV Testing: Patients will be assessed for CMV infection with peripheral blood PCR assay at screening. CMV PCR must be undetectable prior to enrollment. PCR surveillance will continue on therapy at pre-specified time points (Table 10) and as clinically indicated.

C-reactive protein and ferritin: Samples for C-reactive protein and ferritin will be collected according to Table 10 and Table 11. All analyses for CRP and ferritin will be performed locally.

Laboratory Assessment for tumor lysis monitoring: Refer to Section 7.3.5.2.



VV-RIM-00192456-1.0 Approved - 11 Apr 2022 GMT-5:00

***Abnormal Laboratory Values and Laboratory Adverse Events***

All laboratory values must be reviewed by the investigator or authorized designee.

Significantly abnormal test results that occur after start of treatment must be repeated to confirm the nature and degree of the abnormality. When necessary, appropriate ancillary investigations should be initiated. If the abnormality fails to resolve or cannot be explained by events or conditions unrelated to the study drug or its administration, the Medical/Study Director must be consulted. Criteria for reporting laboratory values as an AE are provided Section 9.4.5.

If there are significant laboratory abnormalities prior to study drug administration (including the split infusion) then study drug should be temporarily held until resolution of those abnormalities.

### 8.2.4. Pharmacokinetic Assessments

Samples for drug concentration measurements and PK analysis will be collected at time points listed in Table 10 and Table 11. Any unused samples collected for these assessments may be used for exploratory biomarker research or to investigate AEs as appropriate.

### 8.2.5. Anti-Drug Antibody Measurements and Samples

Samples for ADA and NAb assessment will be collected at time points listed in Table 10 and Table 11. Any unused samples may be used for exploratory biomarker research or for investigation of AEs as appropriate.

### 8.2.6. Survival Data Collection

Every effort will be made to collect survival data on all patients, including patients who withdraw from the study for any reason but have not withdrawn consent to provide survival information. If the death of a patient is not reported, the date of the last patient contact in this study will be used in the determination of the patient's last known date of alive. Patients may be followed remotely by the study site (eg, by telephone) to obtain survival information.

### 8.2.7. Quality of Life Procedures

Patient-reported outcomes will be measured at a frequency indicated in Table 10 using validated electronic patient self-administered EORTC QLQ-C30, FACT-Lym, and EQ-5D-3L questionnaires. Patients will be asked to complete these questionnaires prior to any study procedures being performed at a given study visit.

### 8.2.8. Pharmacodynamic and Exploratory Biomarker Procedures

In this study, biomarker assessments will be performed to explore mechanism of action of odronextamab and evaluate predictive value of specific biomarkers in respect to safety and efficacy.

#### 8.2.8.1. Biomarker Assessments

Biomarker samples will be collected at time points according to Table 10 and Table 11.

Based on the observation of significant but transient increases in CRP and cytokine levels in R1979-HM-1333 and R1979-ONC-1504 studies, CRP, ferritin, and cytokine levels will be closely

monitored in this study. Cytokine upregulation triggered by target engagement may reflect T cell activation. Understanding the magnitude and time course of pharmacodynamic changes in serum cytokines may inform dosing and help understand and manage possible safety signals. Serum samples collected for cytokine analysis may also be used to explore additional biomarkers of interest (for example, baseline rituximab levels, other inflammatory factors, etc).

Changes in lymphocyte subsets and activation status may also be anticipated based on preclinical data and the proposed mechanism of action. Immunophenotyping of T-cell and B-cell subsets and other cell populations (including monocytes and natural killer [NK] cells) in peripheral blood (by flow cytometry) will be performed to assess potential changes upon odronextamab treatment. Further, preliminary data on target occupancy in R1979-HM-1333 and R1979-ONC-1504 studies indicate potential correlation to PK and may be informative in determining the optimal dose and understanding the mechanism of action (MOA). Additional exploratory analysis on PBMC including but not limited to RNA analysis may be conducted.

Minimal Residual Disease (MRD) will be assessed on blood samples collected as described in the laboratory manual. Minimal Residual Disease is a good predictive marker of response to T cell engagers with similar MOA in acute lymphoblastic leukemia (ALL), however, this is still exploratory for NHL. In this study, changes in MRD will be monitored in blood at baseline, at approximately week 12 (± 7 days), and, if the patient achieves CR, at each time point that MRI/CT scans are performed, to evaluate predictive value of MRD in patients with NHL treated with odronextamab.

Tumor and bone marrow specimens will be analyzed for changes in biomarkers of interest including number and distribution of activated T cells, CD20 expression, expression of checkpoint modulators, etc. Additional analyses including, but not limited to, tumor RNA expression and tumor DNA may also be performed.

#### 8.2.8.2. Lymphocyte immunophenotyping (Whole Blood) Monitoring Sample

Lymphocyte immunophenotyping (whole blood) sample will be obtained in order to assess absolute numbers of immune cell subsets at baseline and during treatment, as detailed in Table 10 and Table 11.

#### 8.2.8.3. PBMC Immunophenotyping Sample

Whole blood sample will be obtained as detailed in Table 10 and Table 11, and shipped to the central laboratory for PBMC isolation and cryopreservation. The cryopreserved PBMC sample set will be retrospectively analyzed to characterize the baseline and on-treatment changes in T lymphocyte subsets and activation status. Additional molecular analyses of PBMC sample may be performed (ie, T cell receptor sequencing, RNAseq).

#### 8.2.8.4. Future Biomedical Research

The unused biomarker samples for study-related research, as well as unused PK and ADA samples, will be stored for up to 15 years following completion of the study. The unused samples may be utilized for future biomedical research on indication, odronextamab T cell-mediated killing of CD20+ target cells, and related diseases. No additional samples will be collected for future biomedical research. After 15 years, any residual samples will be destroyed. The results of these future biomedical research analyses will not be presented in the CSR.

#### 8.2.8.5. Genomics Sub-study - Optional

Patients who agree to participate in the genomics sub-study will be required to indicate their consent to participate in the sub-study on the ICF form before collection of the samples. Patients are not required to participate in the genomics sub-study in order to enroll in the primary study. A blood sample for optional genomic DNA extraction will be collected prior to the first dose of odronextamab at week 1 day 1. If a sample is not obtained at that time point, a sample may be collected once at any other study visit.

DNA samples for the genomics sub-study will be coded as defined by the International Council for Harmonisation (ICH) guideline E15. Sub-study samples will be stored for up to 15 years after the final date of the database lock and may be used for research purposes. The purpose of the genomic analyses is to identify genomic associations with clinical or biomarker response, other clinical outcome measures, and possible AEs. In addition, associations between genomic variants and prognosis or progression of NHL as well as other related diseases may also be studied. These data may be used or combined with data collected from other studies to identify and validate genomic markers related to the study drug or to other diseases. Analyses may include sequence determination or single nucleotide polymorphism studies of candidate genes and surrounding genomic regions. Other methods, including whole-exome sequencing, whole-genome sequencing, and DNA and copy number variation may also be performed. The list of methods may be expanded to include novel methodology that may be developed during the course of this study or during the sample storage period.

Results from the genomics sub-study will not be included in the CSR.

# 9. SAFETY DEFINITIONS, REPORTING, AND MONITORING

## 9.1. Obligations of Investigator

The investigator must promptly report to the Institutional Review Board (IRB)/Ethics Committee (EC) all unanticipated problems involving risks to patients/subjects, according to local regulations. This may include death from any cause and all SAEs related to the use of the study drug. It is recommended that all SAEs be reported to the IRB/EC, according to local regulations.

## 9.2. Obligations of Sponsor

During the course of the study, the sponsor will report in an expedited manner all SAEs that are both unexpected and at least reasonably related to the study drug (suspected unexpected serious adverse reaction [SUSAR]), to the health authorities, ECs/IRBs as appropriate, and to the investigators.

Any AE not listed as an expected event in the Reference Safety Information section of the Investigator's Brochure will be considered as unexpected. Any worsening of or new onset of symptoms related to FL/DLBCL/MCL/MZL and other B-NHL which occur during the screening/washout period prior to study drug administration will be considered expected.

In addition, the sponsor will report any other SAEs to the health authorities, according to local regulations.

At the completion of the study, the sponsor will report all safety observations made during the conduct of the trial in the clinical study report to health authorities and IECs/IRBs as appropriate.

## 9.3. Definitions

### 9.3.1. Adverse Event

An AE is any untoward medical occurrence in a patient administered a study drug that may or may not have a causal relationship with the study drug. Therefore, an AE is any unfavorable and unintended sign (including abnormal laboratory finding), symptom, or disease, which is temporally associated with the use of a study drug, whether or not considered, related to the study drug.

An AE also includes any worsening (ie, any clinically significant change in frequency and/or intensity) of a pre-existing condition that is temporally associated with the use of the study drug.

Progression of underlying malignancy will not be considered an AE if it is clearly consistent with the typical progression pattern of the underlying cancer (including time course, affected organs, etc). Clinical symptoms of progression may be reported as AEs if the symptom cannot be determined as exclusively due to the progression of the underlying malignancy or does not fit the expected pattern of progression for the disease under study.

If there is any uncertainty about an AE being due only to progression of the underlying malignancy, it should be reported as an AE or SAE as outlined in Section 9.3.2.

### 9.3.2. Serious Adverse Event

An SAE is any untoward medical occurrence that at any dose:

- Results in **death** – includes all deaths, even those that appear to be completely unrelated to study drug (eg, a car accident in which a patient is a passenger).
- Is **life-threatening** – in the view of the investigator, the patient is at immediate risk of death at the time of the event. This does not include an AE that had it occurred in a more severe form, might have caused death.
- Requires in-patient **hospitalization** or **prolongation of existing hospitalization**. In-patient hospitalization is defined as hospital admission of any duration or an emergency room visit for longer than 24 hours. Prolongation of existing hospitalization is defined as a hospital stay that is longer than was originally anticipated for the event or is prolonged due to the development of a new AE as determined by the investigator or treating physician.
- Results in persistent or significant **disability/incapacity** (substantial disruption of one's ability to conduct normal life functions).
- Is a **congenital anomaly/birth defect**

- Is an **important medical event** – Important medical events may not be immediately life-threatening or result in death or hospitalization, but they might jeopardize the patient or may require intervention to prevent one of the other serious outcomes listed above (eg, intensive treatment in an emergency room or at home for allergic bronchospasm; blood dyscrasias or convulsions that do not result in hospitalization; or development of drug dependency or drug abuse).

Hospitalization or death due solely to manifestations consistent with typical progression of underlying malignancy will not be considered an SAE. Planned hospitalization solely for observation after study drug administration will not be considered an SAE.

The sponsor's medical monitor should be contacted if the investigator is unsure if an event that occurred during or prior to hospitalization for observation, or an event that may be due to disease progression, should be considered serious.

Criteria for reporting SAEs must be followed for these events. See Section 9.4.2 for more information on recording and reporting SAEs.

### 9.3.3. Adverse Events of Special Interest

An AESI (serious or non-serious) is one of scientific and medical concern specific to the sponsor's product or program, for which ongoing monitoring and rapid communication by the investigator to the sponsor can be appropriate. Such an event might warrant further investigation in order to characterize and understand it. Depending on the nature of the event, rapid communication by the trial sponsor to other parties (eg, health authorities) might also be warranted (Section 9.4.3).

### 9.3.4. Infusion Reactions and Cytokine Release Syndrome

Refer to Section 7.3.3.

## 9.4. Recording and Reporting Adverse Events

### 9.4.1. Adverse Events

The investigator (or designee) will seek information on AEs at each patient contact and record all AEs that occur from the time the informed consent is signed until last study follow-up visit. Prior to initiation of study treatment, only the following categories of AEs should be reported on the AE CRF:

- SAEs
- Non-SAEs associated with a protocol-mandated intervention (eg, AEs related to an invasive procedure such as a biopsy)

Other AEs that occur prior to first treatment should be reported on the medical history CRF.

All AEs after the initiation of study treatment up to 90 days following the last dose, or until the start of non-protocol anti-lymphoma therapy (whichever is earlier), regardless of relationship to study treatment will be reported on the AE CRF. In addition, any SAE that the investigator believes may be related to study treatment and that occurs after 90 days following the last dose or after the start of non-protocol anti-lymphoma therapy, whichever is earlier, must be reported.



VV-RIM-00192456-1.0 Approved - 11 Apr 2022 GMT-5:00

Information on follow-up for AEs is provided in Section 9.4.6. Laboratory, vital signs, or ECG abnormalities are to be recorded as AEs as outlined in Section 9.4.5.

### 9.4.2. Serious Adverse Events

All SAEs occurring after the initiation of study treatment up to 90 days following the last dose, or until the start of non-protocol anti-lymphoma therapy (whichever is earlier), regardless of assessment of causal relationship to study drug, must be reported to the sponsor (or designee) within 24 hours.

Information not available at the time of the initial report must be documented in a follow-up report. Substantiating data such as relevant hospital or medical records and diagnostic test reports may also be requested.

In the event the investigator is informed of an SAE that occurs after 90 days following the last dose, or after the start of non-protocol anti-lymphoma therapy (whichever is earlier), only those SAEs deemed by the investigator to be related to study drug will be reported to the sponsor. The investigator should make every effort to obtain follow-up information on the outcome of a treatment-related SAE until the event is resolved or considered chronic and/or stable.

### 9.4.3. Other Events that Require Accelerated Reporting to Sponsor

The following events also require reporting to the sponsor (or designee) within 24 hours of learning of the event:

**Overdose of Study Drug:** Accidental or intentional overdose of at least 2 times the intended dose of study drug within the intended therapeutic window.

**Pregnancy:** Although pregnancy is not considered an AE, it is the responsibility of the investigator to report to the sponsor (or designee), within 24 hours of identification, any pregnancy occurring in a female or female partner of a male study patient, during the study or within 6 months of the last dose of study drug. Any complication of pregnancy affecting a female study patient or female partner of a male study patient, and/or fetus and/or newborn that meets the SAE criteria must be reported as an SAE. Outcome for all pregnancies should be reported to the sponsor.

**Adverse Events of Special Interest:** All AESIs, serious and nonserious, must be reported within 24 hours of identification using the same reporting process as for SAE reporting, per Section 9.4.2. Adverse events of special interest for this study include the following:

- Grade 3 or higher TLS
- Grade 2 or greater CRS (Lee, 2019)
- Grade 3 or greater IRR
- Grade 3 or greater allergic reactions
- Grade 3 or greater infections
- Grade 2 or greater CNS events

- Hepatitis B re-activation
- CMV infection or re-activation

### 9.4.4. Reporting Adverse Events Leading to Withdrawal from the Study

All AEs that lead to a patient's withdrawal from the study must be reported to the sponsor's medical monitor within 30 days.

### 9.4.5. Abnormal Laboratory, Vital Signs, or Electrocardiogram Results

The criteria for determining whether an abnormal objective test finding should be reported as an AE include:

- the test result is associated with accompanying symptoms, and/or
- the test result requires additional diagnostic testing or medical/surgical intervention, and/or
- the test result leads to a change in dosing (outside of protocol-stipulated dose adjustments), discontinuation from the study, significant additional concomitant drug treatment, or other therapy

Contact the medical monitor in the event the investigator feels that an abnormal test finding should be reported as an AE, although it does not meet any of the above criteria.

Repeating an abnormal test, in the absence of any of the above conditions, does not constitute an AE. Any abnormal test result that is determined to be an error does not require reporting as an AE.

Evaluation of severity of laboratory abnormalities will be assessed according to the scale outlined in Section 9.5.1.

### 9.4.6. Follow-up

Information for any non-SAE that starts following initiation of study treatment up to 90 days following the last dose, or until the start of non-protocol anti-lymphoma therapy (whichever is earlier), will be collected from the time of the event until resolution of the event, or until the patient's last study visit, whichever is earlier.

Serious adverse event information will be collected until the event is considered resolved, or is determined to be chronic, and/or stable.

## 9.5. Evaluation of Severity and Causality

### 9.5.1. Evaluation of Severity

The severity of AEs (including test findings classified as AEs) will be graded using the current version of the NCI-CTCAE v 5.0 grading system.

Cytokine Release Syndrome will be graded according to Table 2.

Tumor flare will be graded according to Table 12.

Adverse events not listed in the NCI-CTCAE v5, will be graded according to the scale in Table 12.

**Table 12: NCI-CTCAE Severity Grading System for Adverse Events**

| Grade | Severity | Description |
|---|---|---|
| 1 | Mild | Asymptomatic or mild symptoms; clinical or diagnostic observations only; intervention not indicated. |
| 2 | Moderate | Minimal, local, or noninvasive intervention indicated; limiting age-appropriate instrumental ADL*. |
| 3 | Severe | Severe or medically significant but not immediately life-threatening; hospitalization or prolongation of hospitalization indicated; disabling; limiting self-care ADL**. |
| 4 | Life–threatening | Life-threatening consequences; urgent intervention indicated. |
| 5 | Death | Death related to AE |

* Instrumental Activities of Daily Living (ADL) refer to preparing meals, shopping for groceries or clothes, using the telephone, managing money, etc.

**Self-care ADL refer to bathing, dressing and undressing, feeding self, using the toilet, taking medications, and not bedridden.

### 9.5.2. Evaluation of Causality

***Relationship of Adverse Events to Study Drug:***

The relationship of AEs to study drug will be assessed by the investigator and will be a clinical decision based on all available information. The following question will be addressed:

Is there a reasonable possibility that the AE may have been caused by the study drug?

The possible answers are:

**Not Related:** There is no reasonable possibility that the event may have been caused by the study drug

**Related:** There is a reasonable possibility that the event may have been caused by the study drug

The investigator should justify the causality assessment of each SAE.

A list of factors to consider when assessing the relationship of AEs to study drug is provided below. Please note that this list is not exhaustive.

Is there a reasonable possibility that the event may have been caused by the study drug?

No:

- due to external causes such as environmental factors or other treatment(s) being administered
- due to the patient's disease state or clinical condition
- do not follow a reasonable temporal sequence following the time of administration of the dose of study drug
- do not reappear or worsen when dosing with study drug is resumed

- are not a suspected response to the study drug based upon preclinical data or prior clinical data

Yes:

- could not be explained by environmental factors or other treatment(s) being administered
- could not be explained by the patient's disease state or clinical condition
- follow a reasonable temporal sequence following the time of administration of the dose of study drug
- resolve or improve after discontinuation of study drug
- reappear or worsen when dosing with study drug
- are known or suspected to be a response to the study drug based upon preclinical data or prior clinical data

***Relationship of Adverse Events to Study Conduct (Protocol Specified Procedure):***

The relationship of AEs to a protocol specified procedure will be assessed by the investigator and will be a clinical decision based on all available information. The following question will be addressed:

Is there a reasonable possibility that the AE may have been caused by a protocol specified procedure?

The possible answers are:

**Not Related:** There is no reasonable possibility that the event may have been caused by a protocol specified procedure

**Related:** There is a reasonable possibility that the event may have been caused by a protocol specified procedure

The investigator should justify the causality assessment of each SAE.

A list of factors to consider when assessing the relationship of AEs to a protocol specified procedure is provided below. Please note that this list is not exhaustive.

Is there a reasonable possibility that the event may have been caused by a protocol specified procedure?

No:

- due to external causes such as environmental factors or other treatment(s) being administered
- due to the patient's disease state or clinical condition
- do not follow a reasonable temporal sequence following the course of the study.
- do not reappear or worsen when dosing with study participation is resumed

Yes:

- could not be explained by environmental factors or other treatment(s) being administered
- could not be explained by the patient's disease state or clinical condition
- follow a reasonable temporal sequence following the course of the study.
- resolve or improve after discontinuation from study participation.
- reappear or worsen when study participation is resumed

## 9.6. Safety Monitoring

The investigator will monitor the safety of study patient at his/her site(s) as per the requirements of this protocol and consistent with current Good Clinical Practice (GCP). Any questions or concerns should be discussed with the sponsor in a timely fashion. The sponsor will monitor the safety data from across all study sites. The medical monitor will have primary responsibility for the emerging safety profile of the compound, but will be supported by other departments (eg, Global Patient Safety; Biostatistics and Data Management). Safety monitoring will be performed on an ongoing basis (eg, individual review of SAEs) and on a periodic cumulative aggregate basis.

## 9.7. Investigator Alert Notification

Regeneron (or designee) will inform all investigators participating in this clinical trial, as well as in any other clinical trial using the same investigational drug, of any SAE that meets the relevant requirements for expedited reporting (an AE that is serious, unexpected based on the reference safety information section of odronextamab Investigator's Brochure and has a reasonable suspected causal relationship to the study drug).

# 10. STATISTICAL PLAN

This section provides the basis for the statistical analysis plan (SAP) for the study. The SAP may be revised during the study to accommodate amendments to the clinical study protocol and to make changes to adapt to unexpected issues in study execution and data that may affect the planned analyses. The final SAP will be issued before the database is locked.

Analysis variables are listed in Section 10.4.

## 10.1. Statistical Hypothesis

There is no formal statistical hypothesis in this study.

## 10.2. Justification of Sample Size

### 10.2.1. Follicular Lymphoma Grade 1-3a Cohort

There are limited treatment options for patients with relapsed/refractory FL following 2 lines of treatment. Idelalisib and copanlisib were approved in the US under the accelerated pathway. Idelalisib was approved in the EU, Canada, South Korea, and Australia. However, the phase 3 trials of idelalisib in combination with other cancer medicines were terminated due to increased deaths and serious adverse events. Copanlisib has not been approved outside of the US.

Idelalisib showed an ORR of 57% in a single arm, phase 2 study (DELTA). Copanlisib showed an ORR of 59.2% and a CR rate of 12% in a phase 2 study (CHRONOS-1) for patients who were previously treated with rituximab and an alkylating agent (Dreyling, 2017). The clinical benefits of these agents remain to be verified in the respective confirmatory trials. The preliminary evaluation of odronextamab in a phase 1 study, demonstrated its effectiveness in relapsed/refractory FL, and treatment with this bispecific antibody to date has been associated with favorable tolerability.

This study cohort is designed to evaluate further the efficacy and safety of odronextamab for patients with FL that has relapsed or is refractory to at least 2 prior lines of systemic therapy. A single-stage exact binomial design will be adopted for the primary endpoint of ORR. The 2-sided 95% confidence intervals for the observed ORRs were calculated based on a sample size of 112. Assuming a clinical meaningful ORR is greater than 49%, with 112 patients, an ORR of at least 59% will have a lower bound of the confidence interval that excludes 49%. In addition, if the observed ORR is at least 65%, 70%, and 75%, the lower limit of 95% CI will exclude the ORR of 55%, 60% and 65% respectively; ie, the ORR is significantly different from 55%, 60%, and 65% (Table 13).

**Table 13: The 2-sided 95% Exact Confidence Intervals for Observed ORR Given a Sample Size of 112 Patients**

| Number of Responders | Observed ORR | 95%CI – lower | 95% CI – upper |
|---|---|---|---|
| 50 | 0.45 | 0.352 | 0.543 |
| 56 | 0.50 | 0.404 | 0.596 |
| 62 | 0.55 | 0.457 | 0.648 |
| 66 | 0.59 | 0.492 | 0.681 |
| 67 | 0.60 | 0.501 | 0.690 |
| 73 | 0.65 | 0.556 | 0.739 |
| 78 | 0.70 | 0.602 | 0.780 |
| 84 | 0.75 | 0.659 | 0.827 |

With a sample size of 112 patients, if the true treatment effect of odronextamab is 64%, 70%, 75%, or 80%, the probability is 89%, 89%, 92%, or 92%, respectively, for observed lower bound of 95% CI to exclude 49%, 55%, 60%, or 65%, respectively.

The enrollment in this cohort will continue beyond 112 patients in order to have at least 60 patients treated with the new step-up regimen. With the additional patients, the probability stated above will be higher.

### 10.2.2. Diffuse Large B-Cell Lymphoma

There are limited treatment options for patients with relapsed/refractory DLBCL following 2 lines of treatment. Polatuzumab vedotin (CD79b ADC, MMAE payload) in combination with bendamustine and rituximab (Pola-BR) was granted priority review by FDA in this indication with a PDUFA date of August 19, 2019. Pola-BR showed an ORR of 45% and CR rate of 40% by independent review committee in patients with DLBCL after at least 1 prior line of therapy. Recently, two anti-CD19 chimeric antigen receptor T-cell therapies, axicabtagene ciloleucel and tisagenlecleucel, received marketing approvals to treat adult patients with relapsed or refractory DLBCL in third line, with a CR rate of 54% and 40% respectively, by independent review committee assessment (Schuster, 2019) (Neelapu, 2018a). However, CAR-T therapy is not feasible in all circumstances due to patient co-morbidities or to progressive disease during preparation of the therapy or availability in certain regions. The preliminary evaluation of odronextamab in a phase 1 study, demonstrated its effectiveness in relapsed/refractory DLBCL, and treatment with this bispecific antibody to date has been associated with favorable tolerability.

This study cohort is designed to further evaluate the efficacy and safety of odronextamab for patients with DLBCL that has relapsed or is refractory to at least 2 prior lines of systemic therapy. As an initial step, 68 patients will be randomized into the two treatment arms with 1:1 ratio (Arm 1: 160 mg QW dosing followed by 320 mg Q2W dosing; Arm 2: 320 mg QW dosing followed by 320 mg Q2W dosing). Data from the initial step will be used for descriptive analyses that support PK/pharmacodynamic and advance our understanding of the exposure-response relationship. After the initial step, enrollment will only continue in the 160 mg QW/ 320 mg Q2W arm until a total

of 112 patients (up to approximately 127 patients including the randomized patients in the arm) are reached to further study efficacy at this selected dose regimen.

An exact binomial design will be adopted for the primary endpoint of ORR. The 2-sided 95% confidence intervals for the observed ORR were calculated based on a sample size of 112. Assuming a clinical meaningful ORR is greater than 35%, with 112 patients, an ORR of at least 45% will have a lower bound of the confidence interval that excludes 35%. In addition, if the observed ORR is at least 50%, 55% and 60%, the lower limit of 95% CI will exclude the ORR of 40%, 45% and 50% respectively; ie, the ORR is significantly different from 40%, 45% and 50% (Table 13).

With a sample size of 112 patients, if the true treatment effect of odronextamab is 50%, 55%, 60%or 65%, the probability is 89%, 88%, 86% or 89% respectively, for observed lower bound of 95% CI to exclude 35%, 40%, 45% and 50%, respectively.

The enrollment in this cohort will continue beyond 112 patients in order to have at least 60 patients of DLBCL or Other aggressive B-NHL (minimum 45 DLBCL patients) treated with the new step-up regimen. With the additional patients, the probability stated above will be higher.

### 10.2.3. Mantle Cell Lymphoma after BTK inhibitor therapy Cohort

The median OS of patients after failure of ibrutinib therapy is brief at 2.9 months. There are no proven salvage therapies that have shown benefit after BTK inhibitor failure and such patients have high unmet need at this time (Smith, 2016).

This study cohort is designed to further evaluate the efficacy and safety of odronextamab for patients with MCL that has relapsed or is refractory to systemic therapy and has failed BTK inhibitor therapy. An exact binomial design will be adopted for the primary endpoint ORR. The 2-sided 95% confidence intervals for the observed ORRs were calculated based on a sample size of 78. Assuming a clinical meaningful ORR is greater than 15%, with 78 patients, an ORR of at least 24% will have a lower bound of the confidence interval that excludes 15%. In addition, if the observed ORR is at least 36%, 41% and 47%, the lower limit of 95% CI will exclude the ORR of 25%, 30% and 36% respectively; ie, the ORR rate is significantly different from 25%, 30% and 36% (Table 14).

**Table 14: The 2-sided 95% Exact Confidence Intervals for Observed ORR Given a Sample Size of 78 Patients**

| Number of Responders | Observed ORR | 95%CI – lower | 95% CI – upper |
|---|---|---|---|
| 19 | 0.24 | 0.153 | 0.354 |
| 24 | 0.31 | 0.208 | 0.422 |
| 28 | 0.36 | 0.253 | 0.476 |
| 32 | 0.41 | 0.3001 | 0.527 |
| 37 | 0.47 | 0.3601 | 0.591 |
| 41 | 0.53 | 0.409 | 0.640 |

With a sample size of 78 patients, if the true treatment effect of odronextamab is 30%, 40%, 45%, or 51%, the probability is 89%, 80%, 79%, or 77%, respectively, for observed lower bound of 95% CI to exclude 15%, 25%, 30%, or 36%, respectively.

No change to sample size in the MCL cohort is being made as it is on pause for enrollment.

### 10.2.4. Marginal Zone Lymphoma Cohort

There are limited treatment options for patients with relapsed/refractory MZL following at least one line of treatment. Although ibrutinib received accelerate approval in US with an ORR of 48%, the CR rate was only 3%. It suggests an unmet need for effective salvage therapies for relapsed or treatment-refractory MZL.

This study cohort is designed to further evaluate the efficacy and safety of odronextamab for patients with MZL that has relapsed or is refractory to at least two prior lines of systemic therapy.

An exact binomial design will be adopted for the primary endpoint ORR. The 2-sided 95% confidence intervals for the observed ORRs were calculated based on a sample size of 78. Assuming a clinical meaningful ORR is greater than 20%, with 78 patients, an ORR of at least 31% will have a lower bound of the confidence interval that excludes 20%. In addition, if the observed ORR is at least 41%, 47% and 53%, the lower limit of 95% CI will exclude the ORR of 30%, 36% and 40% respectively; ie, the ORR rate is significantly different from 30%, 36% and 40% (Table 14).

With a sample size of 78 patients, if the true treatment effect of odronextamab is 35%, 45%, 51%, or 55%, the probability is 82%, 79%, 77%, or 71%, respectively, for observed lower bound of 95% CI to exclude 20%, 30%, 36%, or 40%, respectively.

No change to sample size in the MZL cohort is being made as it is on pause for enrollment

### 10.2.5. Other B-Cell non-Hodgkin Lymphoma Cohort

The prognosis for patients with other relapsed/ refractory B-NHL including follicular lymphoma grade 3b, B-cell lymphoblastic lymphoma, Burkitt lymphoma, primary mediastinal large B-cell lymphoma is generally poor. In a retrospective analysis, patients with relapsed or refractory Burkitt and high-grade B-cell leukemia/lymphoma, the median OS was 2.8 months, with a 1-year OS rate of 11% (Short, 2017). These patients have an unmet need for salvage therapies in relapsed/refractory setting.

This study cohort is designed to further evaluate the efficacy and safety of odronextamab for patients with other B-NHL that has relapsed or is refractory to at least two prior lines of systemic therapy. An exact binomial design will be adopted for the primary endpoint of ORR. The 2-sided 95% confidence intervals for the observed ORRs were calculated based on a sample size of 67. Assuming a clinical meaningful ORR is greater than 10%, with 67 patients, an ORR of at least 19% will have a lower bound of the confidence interval that excludes 10%. In addition, if the observed ORR is at least 31% and 43%, the lower limit of 95% CI will exclude the ORR of 20% and 31% respectively; ie, the ORR is significantly different from 20% and 31% (Table 15).

**Table 15: The 2-sided 95% Exact Confidence Intervals for Observed ORR Given a Sample Size of 67 Patients**

| Number of Responders | Observed ORR | 95%CI – lower | 95% CI – upper |
|---|---|---|---|
| 13 | 0.19 | 0.108 | 0.309 |
| 21 | 0.31 | 0.206 | 0.438 |
| 29 | 0.43 | 0.312 | 0.560 |

With a sample size of 67 patients, if the true treatment effect of odronextamab is 20%, 30%, or 41%, the probability is 88%, 77%, or 71% respectively, for observed lower bound of 95% CI to exclude 10%, 20%, or 31%, respectively.

Table 16 summarizes the sample size at the original and modified step-up regimens in each cohort.

**Table 16: Sample Size at Original and Modified Step-up Regimens in Each Cohort**

| | Current enrollment at original step-up regimen as of Amendment 4 | Additional enrollment planned at modified step-up regimen | Maximum cohort size |
|---|---|---|---|
| FL grade 1-3a | 68 | 60 | 128 |
| DLBCL | 101 (67 in Arm 1, 34 in Arm 2) | 45-60 | 161 (127 in Arm 1, 34 in Arm 2) |
| MCL* | 14 | 64 | 78 |
| MZL* | 19 | 60 | 78 |
| Other B-NHL | 14 | 53 | 67 |

* MCL and MZL cohorts will remain on enrollment pause until the proposed step-up dosing regimen has been tested in other disease-specific cohorts.

In addition, if the modified step-up regimen is not deemed acceptable in a specific cohort (i.e., FL grade 1-3a, DLBCL/Other B-NHL, MZL) per interim assessment on grade ≥3 CRS, a revised regimen will be selected for testing in a new sample of 60 patients in that cohort. The primary efficacy endpoints ORR according to Lugano Classification (Cheson, 2014) (Appendix 2) based on independent central review will be summarized along with the 2-sided 95% confidence interval for each disease-specific cohort. Patients who are not evaluable for the Best Overall Response (BOR) will be considered as non-responders.

The ORR as determined by investigator review, or CR rate and DCR as determined by independent central review or by investigator review according to Lugano Classification (Cheson, 2014), which are considered secondary efficacy endpoints, will be summarized along with the 2-sided 95% confidence interval for relevant disease-specific cohorts.

The other secondary efficacy endpoints as measured by DOR, duration of disease control, PFS, and OS will be summarized by median and its 95% confidence interval using the Kaplan-Meier method according to Lugano Classification for each disease-specific cohort.

Quality of Life measured by validated instruments EORTC QLQ-C30, FACT-Lym, and EQ-5D-3L will be summarized by descriptive statistics for each disease-specific cohort.

Safety observations and measurements including drug exposure, AEs, laboratory data, vital signs, and ECOG performance status will be summarized and presented in tables and listings for each disease-specific cohort by original dose regimen and modified dose regimen for the step-up dosing.

## 10.3. Analysis Sets

### 10.3.1. Efficacy Analysis Sets

The full analysis set (FAS) includes all patients who received any study drug. Efficacy and baseline variables will be analyzed using the FAS.

### 10.3.2. Safety Analysis Set

The safety analysis set (SAF) includes all patients who received any study drug. Treatment compliance/administration and all clinical safety variables will be analyzed using the SAF.

### 10.3.3. Pharmacokinetic Analysis Set

The PK analysis set includes all patients who received at least 1 dose of odronextamab and had at least 1 non-missing drug concentration result following the first dose of odronextamab.

### 10.3.4. Immunogenicity Analysis Set

The ADA analysis set includes all treated patients who received at least 1 dose of odronextamab and had at least 1 non-missing anti-drug antibody result following the first dose of study drug.

The NAb analysis set includes all patients who received any study drug and who are negative in the ADA assay or with at least 1 non-missing result in the NAb assay (patients who are ADA negative are set to negative in the NAb analysis set).

## 10.4. Statistical Methods

For continuous variables, descriptive statistics will include the following: the number of patients reflected in the calculation (n), mean, median, standard deviation, Q1, Q3, minimum, and maximum.

For categorical or ordinal data, frequencies and percentages will be displayed for each category.

For time-to-event data, Kaplan-Meier curves and estimates, median survival, survival rate at the key landmark time point along with 95% confidence interval will be provided, if applicable.

In general, the analysis will be performed by disease-specific cohort.

### 10.4.1. Patient Disposition

The following will be provided:

- The total number of screened patients
- The total number of patients included in FAS/SAF

- The total number of patients who discontinued the study treatment, and the reasons for treatment discontinuation
- The total number of patients who discontinued from the study, and the reasons for study discontinuation
- A listing of patients discontinued from treatment/study, along with reasons for discontinuation

### 10.4.2. Demography and Baseline Characteristics

Demographic and baseline characteristics will be summarized descriptively.

### 10.4.3. Efficacy Analyses

#### 10.4.3.1. Primary Efficacy Analysis

Objective response rate (ORR) is defined as the proportion of patients who achieve a best overall response CR or PR during or following study treatment. The primary efficacy endpoint is the ORR according to the Lugano Classification (Cheson, 2014) based on independent central review.

For MZL, overall response assessment will be evaluated using 2 different methods:

1) based on CT-only assessment (without the use of PET-CT)
2) based on the combined CT and PET-CT assessment.

The ORR along with the 2-sided 95% confidence interval will be summarized. Patients who are not evaluable for BOR will be considered as non-responders.

The final analysis for primary efficacy endpoint will be performed after the patients have completed 52 weeks tumor assessments in FL/MZL, 36 weeks tumor assessment in DLBCL/MCL/Other B-NHL or have withdrawn from the study earlier.

Descriptive analysis for the 68 randomized patients in the 2 arms in DLBCL will be performed to support PK/pharmacodynamic and advance the understanding of the exposure-response analysis for the two arms.

An interim efficacy analysis for the primary efficacy endpoint will be performed when 80 patients with FL grade 1-3a have completed 52-week assessment and 127 patients in the DLBCL cohort have completed 36-week assessment or have withdrawn from the study, whichever is later. The details are described in Section 10.5.

#### 10.4.3.2. Secondary Efficacy Analysis

- The ORR as determined by investigator review according to the Lugano Classification will be summarized along with the respective 2-sided 95% confidence interval.
- The CR rate as determined by independent central review or by investigator review according to the Lugano Classification will be summarized along with the respective 2-sided 95% confidence interval.

- The DCR is defined as the proportion of patients who achieve a best overall response CR, PR, or stable disease (SD). Best overall response of SD must have met the response SD criteria at least once ≥12 weeks after start of study treatment. Disease control rate according to investigator evaluation and independent central review will be summarized along with 2-sided 95% confidence interval.
- DOR is defined for responders (patients with a best overall response of CR or PR). It is the time from the date of the first documented CR or PR until the date of the first date of progressive disease, or death due to any cause, whichever occurs first. If a patient has not progressed or died by the analysis cutoff date, DOR will be censored at the time of the last adequate tumor assessment on or before the cutoff date. Duration of response based on investigator evaluation and independent central review will be summarized by the Kaplan-Meier method, if applicable.
- PFS is defined as the time from the start of study treatment until the first date of progressive disease, or death due to any cause, whichever occurs first. If a patient has not progressed or died by the analysis cutoff date, PFS will be censored at the time of the last adequate tumor assessment on or before the cutoff date. Progression free survival based on investigator evaluation and independent central review will be summarized by Kaplan-Meier method, if applicable.
- OS is measured from the start of study treatment until death due to any cause. If a patient is not known to have died at the date of the analysis cut-off, OS will be censored at the last date that patient is documented to be alive. Overall survival will be summarized by the Kaplan-Meier method, if applicable.

### 10.4.4. Safety Analysis

Safety observations and measurements including drug exposure, AEs, laboratory data, vital signs, and ECOG performance status will be summarized and presented in tables and listings.

#### 10.4.4.1. Adverse Events

***Definitions***

For safety variables, 3 observation periods are defined for analysis.

- The **pre-treatment period** is defined as the time between the day of informed consent and the time prior to the first dose of study treatment.
- The **on-treatment period** is defined as the time from the first dose of study treatment up to 90 days after the last dose day of treatment, or the day prior to the start of non-protocol anti-lymphoma therapy, whichever comes first .
- The **post-treatment period** is defined as the time after the on-treatment period .

**Treatment Emergent Adverse Events (TEAEs)** are defined as those AEs that newly occurred or worsened during the on-treatment period and any treatment-related SAEs that occurred during the post-treatment period .

***Analysis***

All AEs reported in this study will be coded using the currently available version of the Medical Dictionary for Regulatory Activities (MedDRA®). Coding will be to lowest level terms. The verbatim text, the preferred term (PT), and the primary system organ class (SOC) will be listed.

Summaries of all TEAEs will include:

- The number (n) and percentage (%) of patients with at least 1 TEAE by SOC and PT
- TEAEs by severity (according to the grading scale outlined in Section 4.2.1), presented by SOC and PT
- TEAEs by relationship to treatment (related, not related), presented by SOC and PT
- Treatment-emergent AESIs (defined with a PT or a prespecified grouping)

Deaths and other SAEs will be listed and summarized.

Treatment-emergent adverse events leading to permanent treatment discontinuation will be listed and summarized.

#### 10.4.4.2. Stopping Rule for Grade ≥3 CRS

To monitor the rate of grade ≥3 CRS with the modified step-up regimen and additional measures for mitigation of CRS, a safety interim assessment rule will be implemented for FL grade 1-3a and DLBCL/other -NHL cohorts as follows.

The first CRS interim safety assessment will be performed in the first 25 patients (FL and DLBCL/Other NHL combined) treated with the modified step-up regimen. Further enrollment in the study will be paused until the 25th patient has received the full QW dose or has discontinued early.

- If 3 or more patients among the first 25 patients treated at the modified step-up regimen have grade ≥3 CRS events, further patient enrollment will stop and an alternative priming regimen, based on the timing of the observed CRS events, will be explored. Stopping and revising the regimen will also be implemented before the total of 25 patients are enrolled if at any time the number of patients experiencing grade ≥3 CRS events reaches 3.
- If 2 or less patients experience grade ≥3 CRS in the first 25 overall patients, enrollment will resume for all cohorts.

The second CRS interim safety assessment will be cohort-specific (ie, FL and DLBCL/Other NHL) and will be performed in the first 25 patients (in the specific cohort) treated with the modified step-up regimen. Further enrollment in the cohort will be paused until the 25th patient in that cohort has received the full QW dose or discontinued early.

- If 3 or more patients among the first 25 patients treated at the modified step-up regimen have grade ≥3 CRS events, further patient enrollment will stop and an alternative priming regimen, based on the timing of the observed CRS events, will be explored. Stopping and revising the regimen will also be implemented before the total of 25 patients are enrolled if at any time the number of patients experiencing grade ≥3 CRS events reaches 3.

- If 2 or less patients experience grade ≥3 CRS in the first 25 patients, enrollment will continue for the cohort. In this case, the final safety assessment of the regimen will be performed at 60 patients:
  - If a total of 5 or more patients experience grade ≥3 CRS in 60 patients, or at any time prior to a total of 60 patient enrolled, enrollment will stop, and a revised regimen will be selected for testing in a new sample of 60 patients and follow the same decision criteria described above.
  - If a total of 4 or less patients out of 60 experience grade ≥3 CRS, the regimen will be deemed acceptable.

If stopping is triggered in one cohort (FL or DLBCL/Other NHL), enrollment in both cohorts will be stopped until a revised regimen is implemented.

The aforementioned criteria were determined based on the lower bound of the 1-sided 80% confidence interval (CI) of the incidence rate of grade ≥3 CRS events being lower than 4%. In addition to these stopping rules, if at any time a second patient in both FL grade 1-3a and DLBCL/Other B-NHL cohorts combined experiences a grade ≥3 CRS event, the dose of oral dexamethasone (or equivalent) before and after odronextamab dosing days will be increased to 20 mg, and earlier tocilizumab intervention will be implemented (i.e., tocilizumab use for patients with grade 1 CRS of fever lasting >24 hours and for all patients with grade ≥2 CRS).

During the conduct of the study, safety data will be reviewed on an ongoing basis, with close monitoring of grade 3 or higher CRS events by the Sponsor's medical monitoring team and global patient safety lead. If CRS events fulfil protocol defined criteria for stopping, then further enrolment will be stopped for all cohorts, all available safety data related to CRS events will be analyzed and a revised regimen will be implemented in subsequent amendment.

**Safety Pausing Rule after Transition from Q2W to Q4W Dosing**

Transition from Q2W to Q4W will be paused if the lower bound of the 1-sided 80% confidence interval of the estimated grade ≥2 CRS rate excludes 20% (

Table 17). This evaluation will be performed for patients treated at 160 mg Q4W and 320 mg Q4W from all cohorts with that dose, respectively.

For each of these doses (160 mg and 320 mg) transitioned to Q4W dosing, if the lower bound of the 1-sided 80% confidence interval of the grade ≥2 CRS rate is greater than 20% (ie, 5 or more patients among the first 16 patients treated with at least 2 doses after the transition from Q2W to Q4W dosing at 160 mg or 320 mg odronextamab have grade ≥2 CRS), then the transition to Q4W dosing will be paused, and all patients will continue at Q2W dosing. Patients who experience grade ≥2 CRS after the first dose following transition from Q2W to Q4W dosing will be included in the analysis.

Further, patients must have received the assigned QW full dose at a Q2W schedule for at least 3 preceding doses before switching from Q2W to Q4W dosing.

**Table 17: Percent of Patients with Grade ≥2 CRS with 1-Sided 80% Confidence Interval**

| Dose | N | n | Grade ≥2 CRS rate | 80% lower bound | Action |
|---|---|---|---|---|---|
| 160 mg Q4W | 16 | 4 | 0.250 | 0.159 | Continue if no more than 4 patients with grade ≥2 CRS |
| | | 5 | 0.312 | 0.215 | Pause transition if at least 5 patients with grade ≥2 CRS |
| 320 mg Q4W | 16 | 4 | 0.250 | 0.159 | Continue if no more than 4 patients with grade ≥2 CRS |
| | | 5 | 0.312 | 0.215 | Pause transition if at least 5 patients with grade ≥2 CRS |

N: number of patients who receive at least two Q4W doses or experience grade ≥2 CRS after the first dose following transition from Q2W to Q4W dosing.
n: number of patients who receive at least one Q4W dose and experience grade ≥2 CRS during Q4W dosing.

#### 10.4.4.3. Other Safety Assessments

***Vital Signs***

Vital signs (temperature, pulse, pulse oximetry, blood pressure, and respiration rate) will be summarized by baseline and change from baseline to each scheduled assessment time with descriptive statistics.

***Laboratory Evaluations***

Laboratory test results will be summarized by baseline and change from baseline to each scheduled assessment time with descriptive statistics.

Shift tables based on baseline normal/abnormal and other tabular and graphical methods will be used to present the results for laboratory tests of interest.

Listings will be provided with flags indicating the out of laboratory range values.

#### 10.4.4.4. Treatment Exposure

Treatment duration, dose intensity, and number of doses administered will be summarized.

#### 10.4.4.5. Treatment Compliance

Not applicable.

### 10.4.5. Pharmacokinetics

#### 10.4.5.1. Analysis of Drug Concentration Data

Odronextamab concentrations in serum over time will be measured.

Pharmacokinetic parameters will be generated and summarized with descriptive statistics. Odronextamab PK parameters may include but are not limited to:

- Cmax: maximal concentration in a dosing interval
- Cmin: trough concentration in a dosing interval

Data collected in this study may be used for population PK analysis. The result will be described in a separate report.

### 10.4.6. Analysis of Immunogenicity Data

Immunogenicity will be characterized by the ADA and NAb response observed:

- ADA negative, defined as ADA negative response in the ADA assay at all time points, regardless of any missing samples.
- Pre-existing immunoreactivity, defined as a positive ADA assay response at baseline, with all post-dose ADA results negative, or a positive assay response at baseline, with all post-dose ADA assay responses less than 9-fold over baseline titer levels
- Treatment-emergent ADA response, defined as any post-dose positive ADA assay response when the baseline results are negative
  - Treatment-emergent ADA response may be further characterized as persistent, transient, or indeterminate
- Treatment boosted ADA response, defined as any post-dose positive ADA assay response that is 9-fold over baseline titer levels when baseline is positive in the ADA assay
- Maximum ADA Titer Category
  - Low (titer <1,000)
  - Moderate (1,000≤ titer ≤10,000)
  - High (titer >10,000)

Listings of pre-existing, treatment-boosted, and treatment-emergent ADA responses, ADA titers and NAb positivity by patient, time point, and dose cohort will be provided. Incidence of treatment-emergent ADA and NAb will be assessed as absolute occurrence (N) and percent of patients (%), grouped by study cohorts and ADA titer level.

Plots of drug concentrations will be examined and the influence of ADAs and NAbs on individual PK profiles may be evaluated. Assessment of impact of ADA and NAbs on safety and efficacy may be provided.

### 10.4.7. Analysis of Quality of Life Data

Quality of life measured by validated instruments EORTC QLQ-C30, FACT-Lym, and EQ-5D-3L will be summarized by descriptive statistics.

### 10.4.8. Analysis of Biomarker Data

Biomarker analyses will be summarized in a separate report. Detailed description of statistical methods that will be used for biomarker data analyses will be provided in a separate Biomarker Analytical Plan.

### 10.4.9. Pharmacokinetic/Pharmacodynamic Analyses

Pharmacokinetic/pharmacodynamic analyses may be conducted on exploratory biomarkers, as appropriate.

### 10.4.10. Exposure-Response Analysis

Exposure-Response (E-R) analyses may be conducted on efficacy endpoints, safety, and relevant exploratory biomarkers, as appropriate.

## 10.5. Interim Analysis

An interim efficacy analysis for will be performed when 80 patients with FL grade 1-3a have completed 52-week assessment and 127 patients in the DLBCL cohort have completed 36-week assessment or have withdrawn from the study earlier, whichever is later. The ORR and associated 95% confidence interval will be summarized for FL cohort. As the primary objective of this interim efficacy analysis is point estimation on ORR and characterization of the precision of point estimation, the study will not be stopped for perceived efficacy. For other efficacy endpoints, 2-sided 95% confidence interval will also be presented.

The sponsor will perform an administrative review for efficacy after at least 70 patients have been treated with the 0.7/4/20 mg step-up regimen and have completed a 4-week safety review. At the administrative review, summary of safety for all cohorts and summary of efficacy for the FL grade 1-3a and DLBCL cohorts will be performed.

An interim futility analysis will be performed for each of the MCL, MZL, and other B-NHL disease-specific cohorts after the pre-specified number of evaluable patients in the cohort have completed tumor assessments at 28 weeks or have withdrawn from the study earlier (see Table 18). The futility boundary is determined based on lack of clinically meaningful activity below the threshold in each disease-specific cohort. The enrollment for each disease-specific cohort will continue unless its futility boundary is crossed.

- MCL after BTK inhibitor therapy cohort: The futility boundary of 15% ORR is determined due to the lack of standard of care options. Therefore, if fewer than 5 responders are observed among the first 27 patients, this cohort will be stopped for futility.
- MZL cohort: The futility boundary of 20% ORR is determined due to enrollment of patients with relapsed/refractory disease following failure of standard of care therapies. Therefore, if fewer than 6 responders are observed among the first 27 patients, this cohort will be stopped for futility.
- Other B-NHL cohort: The futility boundary of 10% ORR is determined for the entire cohort due to the enrollment of heterogenous subtypes with aggressive lymphomas that have poor outcomes in relapsed/refractory setting. Due to low incidence, it is



VV-RIM-00192456-1.0 Approved - 11 Apr 2022 GMT-5:00

anticipated that there will be very few patients of each subtype in this cohort limiting formal statistical analysis of any particular subtype. Therefore, if fewer than 4 responders are observed among the first 34 patients, this cohort will be stopped for futility.

**Table 18: Interim Futility Analysis**

| Study cohort | Total sample size N | Sample size at Interim Futility n1 | Interim futility stopping boundary |
|---|---|---|---|
| FL grade 1-3a | 128 | NA | NA |
| DLBCL | 112 to 127 | NA | NA |
| MZL | 78 | 27 | <6 responders |
| MCL after BTK inhibitor | 78 | 27 | <5 responders |
| Other B-NHL | 67 | 34 | <4 responders |

## 10.6. Additional Statistical Data Handling Conventions

The following analysis and data conventions will be followed:

Definition of baseline:

- Unless otherwise specified, the last assessment before the initial administration of odronextamab will be considered the baseline evaluation.

General rules for handling missing data:

- Rules for handling missing data for assessment (other than efficacy)
- If the start date of an AE or concomitant medication is incomplete or missing, it will be assumed to have occurred on or after the intake of study medication, except if an incomplete date (eg, month and year) clearly indicates that the event started prior to treatment. If the partial date indicates the same month or year of the intake of study medication date, then the start date by the study medication intake date will be imputed; otherwise, the missing day or month by the first day or the first month will be imputed.
- No imputations for missing laboratory data, ECG data, vital sign data, or physical examination data will be made.

Visit windows:

- Assessments taken outside of protocol allowable intervals will be displayed according to the CRF assessment recorded by the investigator.

Unscheduled assessments:

- Extra assessments (laboratory data or vital signs associated with non-protocol clinical visits or obtained in the course of investigating or managing AEs) will be included in listings, but not by visit summaries. If more than 1 laboratory value is available for a given visit, the first observation will be used in summaries and all observations will be presented in listings.

## 10.7. Statistical Considerations Surrounding the Premature Termination of a Study

If the study is terminated prematurely, only those parameters required for the development program and/or reporting to regulatory authorities will be summarized. Investigator and sponsor responsibilities surrounding the premature termination of a study are presented in Section 16.1.

# 11. DATA MANAGEMENT AND ELECTRONIC SYSTEMS

## 11.1. Data Management

A data management plan specifying all relevant aspects of data processing for the study (including data validation, cleaning, correcting, releasing) will be maintained and stored at Regeneron.

A medical coding plan will specify the processes and the dictionary used for coding. All data coding (eg, AEs, baseline findings, medication, medical history) will be done using internationally recognized and accepted dictionaries.

The CRF data for this study will be collected with an electronic data capture (EDC) tool. User training must be documented before the user is granted access to the EDC system.

Data management procedures will be implemented to be able to consider the re-treated patients for the entirety of exposure to study drug.

## 11.2. Electronic Systems

Electronic systems that may be used to process and/or collect data in this study will include the following:

- IVRS/IWRS system – study drug supply and management
- EDC system – data capture
- Statistical Analysis System – statistical review, analysis, and reporting
- Pharmacovigilance safety database

# 12. STUDY MONITORING

## 12.1. Monitoring of Study Sites

The study monitor and/or designee (eg, contract research organization [CRO] monitor) will visit each site prior to enrollment of the first patient, and periodically during the study.

The investigator must allow study-related monitoring.

The study monitors will perform ongoing source data review to verify that data recorded in the CRF by authorized site personnel are accurate, complete, and verifiable from source documents, that the safety and rights of patients are being protected, and that the study is being conducted in accordance with the current approved protocol version and any other study agreements, ICH GCP, and all applicable regulatory requirements

## 12.2. Source Document Requirements

Investigators are required to prepare and maintain adequate and accurate patient records (source documents).

The investigator must keep all source documents on file with the CRF (throughout this protocol, CRF refers to either a paper CRF or an electronic CRF). Case report forms and source documents must be available at all times for inspection by authorized representatives of the sponsor and regulatory authorities.

## 12.3. Case Report Form Requirements

Study data obtained in the course of the clinical study will be recorded on electronic Case Report Forms (CRFs) within the EDC system by trained site personnel. All required CRFs must be completed for each and every patient enrolled in the study. After review of the clinical data for each patient, the investigator must provide an electronic signature. A copy of each patient CRF casebook is to be retained by the investigator as part of the study record and must be available at all times for inspection by authorized representatives of the sponsor and regulatory authorities.

Corrections to the CRF will be entered in the CRF by the investigator or an authorized designee. All changes, including date and person performing corrections, will be available using the audit trail, which is part of the EDC system. For corrections made through data queries, a reason for any alteration must be provided.

# 13. AUDITS AND INSPECTIONS

This study may be subject to a quality assurance audit or inspection by the sponsor or regulatory authorities. Should this occur, the investigator is responsible for:

- Informing the sponsor of a planned inspection by the authorities as soon as notification is received, and authorizing the sponsor’s participation in the inspection
- Providing access to all necessary facilities, study data, and documents for the inspection or audit
- Communicating any information arising from inspection by the regulatory authorities to the sponsor immediately
- Taking all appropriate measures requested by the sponsor to resolve the problems found during the audit or inspection

Documents subject to audit or inspection include but are not limited to all source documents, CRFs, medical records, correspondence, ICFs, EC files, documentation of certification and quality control of supporting laboratories, and records relevant to the study maintained in any supporting pharmacy facilities. Conditions of study material storage are also subject to inspection. In addition, representatives of the sponsor may observe the conduct of any aspect of the clinical study or its supporting activities both within and outside of the investigator's institution.

In all instances, the confidentiality of the data must be respected.

# 14. ETHICAL AND REGULATORY CONSIDERATIONS

## 14.1. Good Clinical Practice Statement

It is the responsibility of both the sponsor and the investigator(s) to ensure that this clinical study will be conducted in accordance with the ethical principles that have their origin in the Declaration of Helsinki, and that are consistent with the ICH guidelines for GCP and applicable regulatory requirements.

## 14.2. Informed Consent

The principles of informed consent are described in ICH guidelines for GCP.

The ICF used by the investigator must be reviewed and approved by the sponsor prior to submission to the appropriate EC. A copy of the EC -approved ICF and documentation of approval must be provided to the sponsor before study drug will be shipped to the study site.

It is the responsibility of the investigator or designee (if acceptable by local regulations) to obtain written informed consent from each patient prior to his/her participation in the study and after the aims, methods, objectives, and potential hazards of the study have been explained to the patient in language that he/she can understand. The ICF should be signed and dated by the patient and by the investigator or authorized designee who reviewed the ICF with the patient.

- Patients who can write but cannot read will have the ICF read to them before signing and dating the ICF.
- Patients who can understand but who can neither write nor read will have the ICF read to them in presence of an impartial witness, who will sign and date the ICF to confirm that informed consent was given.

The original ICF must be retained by the investigator as part of the patient's study record, and a copy of the signed ICF must be given to the patient.

If new safety information results in significant changes in the risk/benefit assessment, the ICF must be reviewed and updated appropriately. All study patients must be informed of the new information and provide their written consent if they wish to continue in the study. The original signed revised ICF must be maintained in the patient's study record and a copy must be given to the patient.

## 14.3. Patients Confidentiality and Data Protection

The investigator must take all appropriate measures to ensure that the anonymity of each study patient will be maintained. Patients should be identified by a patient identification number only on CRFs or other documents submitted to the sponsor. Documents that will not be submitted to the sponsor (eg, signed ICF) must be kept in strict confidence.

The patient's and investigator's personal data, which may be included in the sponsor database, will be treated in compliance with all applicable laws and regulations. The sponsor shall take all appropriate measures to safeguard and prevent access to this data by any unauthorized third party.

## 14.4. Ethics Committee

An appropriately constituted IRB/EC, as described in ICH guidelines for GCP, must review and approve:

- The protocol, ICF, and any other materials to be provided to the patients (eg, advertising) before any patient may be enrolled in the study
- Any amendment or modification to the study protocol or ICF before implementation, unless the change is necessary to eliminate an immediate hazard to the patient, in which case the IRB/EC should be informed as soon as possible
- Ongoing studies on an annual basis or at intervals appropriate to the degree of risk

In addition, the IRB/EC should be informed of any event likely to affect the safety of patients or the continued conduct of the clinical study.

A copy of the EC approval letter with a current list of the EC members and their functions must be received by the sponsor prior to shipment of drug supplies to the investigator. The approval letter should include the study number and title, the documents reviewed, and the date of the review.

Records of the EC review and approval of all study documents (including approval of ongoing studies) must be kept on file by the investigator.

## 14.5. Clinical Study Data Transparency

Final study results will be published on a public clinical trial website according to applicable local guidelines and regulations. Treatment codes will be disseminated to each investigation site thereafter.

# 15. PROTOCOL AMENDMENTS

The sponsor may not implement a change in the design of the protocol or ICF without an EC-approved amendment. Regulatory approvals will be required in accordance with local regulations.

# 16. PREMATURE TERMINATION OF THE STUDY OR CLOSE-OUT OF A SITE

## 16.1. Premature Termination of the Study

The sponsor has the right to terminate the study prematurely. Reasons may include efficacy, safety, or futility, among others. Should the sponsor decide to terminate the study, the investigator(s) will be notified in writing.

## 16.2. Close-out of a Site

The sponsor and the investigator have the right to close-out a site prematurely.

***Investigator's Decision***

The investigator must notify the sponsor of a desire to close-out a site in writing, providing at least 30 days' notice. The final decision should be made through mutual agreement with the sponsor. Both parties will arrange the close-out procedures after review and consultation.

***Sponsor's Decision***

The sponsor will notify the investigator(s) of a decision to close-out a study site in writing. Reasons may include the following, among others:

- The investigator has received all items and information necessary to perform the study, but has not enrolled any patient within a reasonable period of time
- The investigator has violated any fundamental obligation in the study agreement, including but not limited to, breach of this protocol (and any applicable amendments), breach of the applicable laws and regulations, or breach of any applicable ICH guidelines
- The total number of patients required for the study are enrolled earlier than expected

In all cases, the appropriate EC and Health Authorities must be informed according to applicable regulatory requirements, and adequate consideration must be given to the protection of the patients' interests.

# 17. STUDY DOCUMENTATION

## 17.1. Certification of Accuracy of Data

A declaration assuring the accuracy and content of the data recorded on the CRF must be signed electronically by the investigator. This signed declaration accompanies each set of patients' final CRF that will be provided to the sponsor.

## 17.2. Retention of Records

The investigator must retain all essential study documents, including ICFs, source documents, investigator copies of CRFs, and drug accountability records for at least 15 years following the completion or discontinuation of the study, or longer, if a longer period is required by relevant regulatory authorities. The investigator must consult with the sponsor before discarding or destroying any essential study documents following study completion or discontinuation. Records must be destroyed in a manner that ensures confidentiality.

If the investigator's personal situation is such that archiving can no longer be ensured, the investigator must inform the sponsor and the relevant records will be transferred to a mutually agreed-upon destination.

# 18. DATA QUALITY ASSURANCE

In accordance with ICH E6, the sponsor is responsible for quality assurance to ensure that the study is conducted, and the data generated, recorded, and reported in compliance with the protocol,

GCP, and any applicable regulatory requirement(s). The planned quality assurance and quality control procedures for the study are summarized.

***Data Management***

The sponsor is responsible for the data management of this study including quality checking of the data (Section 11.1).

***Study Monitoring***

The investigator must allow study-related monitoring, IRB/EC review, audits, and inspections from relevant health regulatory authorities, and provide direct access to source data documents (Section 12.1, Section 12.2, and Section 13).

The study monitors will perform ongoing source data review to verify that data recorded in the CRF by authorized site personnel are accurate, complete, and verifiable from source documents, that the safety and rights of subjects/patients are being protected, and that the study is being conducted in accordance with the current approved protocol version and any other study agreements, ICH GCP, and all applicable regulatory requirements (Section 12.1).

All subject/patient data collected during the study will be recorded on paper or electronic CRF unless the data are transmitted to the sponsor or designee electronically (eg, laboratory data). The investigator is responsible for affirming that data entries in the CRF are accurate and correct by electronically signing a declaration that accompanies each set of patient/subject final CRF (Section 12.3 and Section 17.2).

***Study Documentation***

The investigator must maintain accurate documentation (source data) that supports the information entered in the CRF (Section 12.2).

The investigator will retain all records and documents, including signed ICFs, pertaining to the conduct of this study for at least 15 years after study completion, unless local regulations or institutional policies require a longer retention period. No records may be destroyed during the retention period without the written approval of the sponsor. No records may be transferred to another location or party without written notification to the sponsor Section 17.2).

# 19. CONFIDENTIALITY

Confidentiality of information is provided as a separate agreement.

# 20. FINANCING AND INSURANCE

Financing and insurance information is provided as a separate agreement.

# 21. PUBLICATION POLICY

The publication policy is provided as a separate agreement.

# 22. REFERENCES

Ahmed N, Caimi P, Reese JS, Otegbeye F, Patel S, Schneider D, et al. Prophylactic Tocilizumab in Patients with Relapsed Refractory Lymphoma Treated with Anti CD19 Chimeric Antigen Receptor T-Cell Therapy. Biology of Blood and Marrow Transplantation 2020; 26(3, Supplement):S275-S276.

ALIQOPA™. http://labeling.bayerhealthcare.com/html/products/pi/Aliqopa_PI.pdf: Bayer HealthCare Pharmaceuticals Inc.; 2018.^

Bannerji R, Advani R, Brown J, Arnason J, Barnes J, Allan J, et al. Safety and preliminary clinical activity of REGN1979, an anti-CD20 × anti-CD3 bispecific antibody, in patients with B-NHL previously treated with CD20-directed antibody therapy. Presented at: American Society of Hematology Annual Meeting; December 9–12, 2017, 2017; Atlanta, GA.

Beham-Schmid C. Aggressive lymphoma 2016: revision of the WHO classification. Memo 2017; 10(4):248-254.

Brudno JN,Kochenderfer JN. Toxicities of chimeric antigen receptor T cells: recognition and management. Blood 2016; 127(26):3321-3330.

Caimi P, Sharma A, Rojas P, Patel S, Reese J, Otegbeye F, et al. 738 CAR-T Therapy for Lymphoma with Prophylactic Tocilizumab: Decreased Rates of Severe Cytokine Release Syndrome without Excessive Neurologic Toxicity. 2020; 62nd ASH Annual MEeting and Exposition December 5-8, 2020.

CALQUENCE® [Package Insert]. 2017; Astra Pharmaceuticals.

Casulo C, Day B, Dawson KL, Zhou X, Flowers CR, Farber CM, et al. Disease characteristics, treatment patterns, and outcomes of follicular lymphoma in patients 40 years of age and younger: an analysis from the National Lymphocare Studydagger. Ann Oncol 2015; 26(11):2311-2317.

Chapuy B, Stewart C, Dunford AJ, Kim J, Kamburov A, Redd RA, et al. Molecular subtypes of diffuse large B cell lymphoma are associated with distinct pathogenic mechanisms and outcomes. Nat Med 2018; 24(5):679-690.

Cheson BD, Ansell S, Schwartz L, Gordon LI, Advani R, Jacene HA, et al. Refinement of the Lugano Classification lymphoma response criteria in the era of immunomodulatory therapy. Blood 2016; 128(21):2489-2496.

Cheson BD, Fisher RI, Barrington SF, Cavalli F, Schwartz LH, Zucca E, et al. Recommendations for initial evaluation, staging, and response assessment of Hodgkin and non-Hodgkin lymphoma: the Lugano classification. J Clin Oncol 2014; 32(27):3059-3068.

Cheson BD, Wendtner CM, Pieper A, Dreyling M, Friedberg J, Hoelzer D, et al. Optimal use of bendamustine in chronic lymphocytic leukemia, non-Hodgkin lymphomas, and multiple

myeloma: treatment recommendations from an international consensus panel. Clin Lymphoma Myeloma Leuk 2010; 10(1):21-27.

COPIKTRA® [Package Inert]. Verastem, Inc. https://www.accessdata.fda.gov/drugsatfda_docs/label/2018/211155s000lbl.pdf. Published 2018.

Davila ML, Riviere I, Wang X, Bartido S, Park J, Curran K, et al. Efficacy and toxicity management of 19-28z CAR T cell therapy in B cell acute lymphoblastic leukemia. Sci Transl Med 2014; 6(224):224ra225.

Dreyling M, Santoro A, Mollica L, Leppa S, Follows GA, Lenz G, et al. Phosphatidylinositol 3-Kinase Inhibition by Copanlisib in Relapsed or Refractory Indolent Lymphoma. J Clin Oncol 2017; 35(35):3898-3905.

Dreyling M, Thieblemont C, Gallamini A, Arcaini L, Campo E, Hermine O, et al. ESMO Consensus conferences: guidelines on malignant lymphoma. part 2: marginal zone lymphoma, mantle cell lymphoma, peripheral T-cell lymphoma. Ann Oncol 2013; 24(4):857-877.

Epperla N, Shana'ah AY, Jones D, Christian BA, Ayyappan S, Maddocks K, et al. Resistance mechanism for ibrutinib in marginal zone lymphoma. Blood Adv 2019; 3(4):500-502.

Friedberg JW. Relapsed/refractory diffuse large B-cell lymphoma. Hematology Am Soc Hematol Educ Program 2011; 2011:498-505.

Friedberg JW. Treatment of follicular non-Hodgkin's lymphoma: the old and the new. Semin Hematol 2008; 45(3 Suppl 2):S2-6.

Gardner RA, Ceppi F, Rivers J, Annesley C, Summers C, Taraseviciute A, et al. Preemptive mitigation of CD19 CAR T-cell cytokine release syndrome without attenuation of antileukemic efficacy. Blood 2019; 134(24):2149-2158.

Global Cancer Observatory. 908 Europe Fact Sheets. https://gco.iarc.fr/today/data/factsheets/populations/908-europe-fact-sheets.pdf. Published 2020.

Grupp SA, Kalos M, Barrett D, Aplenc R, Porter DL, Rheingold SR, et al. Chimeric antigen receptor-modified T cells for acute lymphoid leukemia. N Engl J Med 2013; 368(16):1509-1518.

Hiddemann W,Cheson BD. How we manage follicular lymphoma. Leukemia 2014; 28(7):1388-1395.

Hoster E, Klapper W, Hermine O, Kluin-Nelemans HC, Walewski J, van Hoof A, et al. Confirmation of the mantle-cell lymphoma International Prognostic Index in randomized trials of the European Mantle-Cell Lymphoma Network. J Clin Oncol 2014; 32(13):1338-1346.

Howard SC, Jones DP,Pui CH. The tumor lysis syndrome. N Engl J Med 2011; 364(19):1844-1854.

IMBRUVICA® [Package Insert]. 2013; Janseen Biotech, Inc.

Kahl BS, Cheson BD,Friedberg JW. Clinical Roundtable Monograph: current treatment options for NHL patients refractory to standard therapy: recent data in single-agent and combination therapy. Clin Adv Hematol Oncol 2010; 8(5):1-16.

Klastersky J, de Naurois J, Rolston K, Rapoport B, Maschmeyer G, Aapro M, et al. Management of febrile neutropaenia: ESMO Clinical Practice Guidelines. Ann Oncol 2016; 27(suppl 5):v111-v118.

KYMRIAH™ [Package Insert]. 2017; Novartis Pharmaceuticals Corporation.

Lee DW, Gardner R, Porter DL, Louis CU, Ahmed N, Jensen M, et al. Current concepts in the diagnosis and management of cytokine release syndrome. Blood 2014; 124(2):188-195.

Lee DW, Santomasso BD, Locke FL, Ghobadi A, Turtle CJ, Brudno JN, et al. ASTCT Consensus Grading for Cytokine Release Syndrome and Neurologic Toxicity Associated with Immune Effector Cells. Biol Blood Marrow Transplant 2019; 25(4):625-638.

Lee L, Wang L,Crump M. Identification of potential surrogate end points in randomized clinical trials of aggressive and indolent non-Hodgkin's lymphoma: correlation of complete response, time-to-event and overall survival end points. Ann Oncol 2011; 22(6):1392-1403.

Lenz G, Dreyling M, Hoster E, Wörmann B, Dührsen U, Metzner B, et al. Immunochemotherapy with rituximab and cyclophosphamide, doxorubicin, vincristine, and prednisone significantly improves response and time to treatment failure, but not long-term outcome in patients with previously untreated mantle cell lymphoma: results of a prospective randomized trial of the German Low Grade Lymphoma Study Group (GLSG). J Clin Oncol 2005; 23(9):1984-1992.

Leonard JP, Trneny M, Izutsu K, Fowler NH, Hong X, Zhu J, et al. AUGMENT: A Phase III Study of Lenalidomide Plus Rituximab Versus Placebo Plus Rituximab in Relapsed or Refractory Indolent Lymphoma. J Clin Oncol 2019; 37(14):1188-1199.

Locke FL, Neelapu SS, Bartlett NL, Siddiqi T, Chavez JC, Hosing CM, et al. Phase 1 Results of ZUMA-1: A Multicenter Study of KTE-C19 Anti-CD19 CAR T Cell Therapy in Refractory Aggressive Lymphoma. Mol Ther 2017; 25(1):285-295.

Marcus R, Davies A, Ando K, Klapper W, Opat S, Owen C, et al. Obinutuzumab for the First-Line Treatment of Follicular Lymphoma. N Engl J Med 2017; 377(14):1331-1344.

Martin P, Maddocks K, Leonard JP, Ruan J, Goy A, Wagner-Johnston N, et al. Postibrutinib outcomes in patients with mantle cell lymphoma. Blood 2016; 127(12):1559-1563.

Maurer MJ, Bachy E, Ghesquieres H, Ansell SM, Nowakowski GS, Thompson CA, et al. Early event status informs subsequent outcome in newly diagnosed follicular lymphoma. Am J Hematol 2016; 91(11):1096-1101.

Maus MV, Alexander S, Bishop MR, Brudno JN, Callahan C, Davila ML, et al. Society for Immunotherapy of Cancer (SITC) clinical practice guideline on immune effector cell-related adverse events. J Immunother Cancer 2020; 8(2).

Morgan ET. Regulation of cytochrome p450 by inflammatory mediators: why and how? Drug Metab Dispos 2001; 29(3):207-212.

Nagorsen D, Kufer P, Baeuerle PA,Bargou R. Blinatumomab: a historical perspective. Pharmacol Ther 2012; 136(3):334-342.

National Comprehensive Cancer Network [NCCN]. NCCN Clinical Practice Guidelines in Oncology (NCCN Guidelines®). Prevention and Treatment of Cancer-Related Infections, Version 1.2020. https://www.nccn.org/professionals/physician_gls/PDF/infections.pdf. Published 2019. Updated 17 Dec 2019. Accessed 01 Apr 2020.

Neelapu S, Ghobadi A, Jacobson C, Miklos D,al e. 2-Year Follow-up and High-Risk Subset Analysis of Zuma-1, the Pivotal Study of Axicabtagene Ciloleucel (Axi-Cel) in Patients with Refractory Large B Cell Lymphoma. Blood 2018a; 132:2967.

Neelapu SS, Tummala S, Kebriaei P, Wierda W, Gutierrez C, Locke FL, et al. Chimeric antigen receptor T-cell therapy - assessment and management of toxicities. Nat Rev Clin Oncol 2018b; 15(1):47-62.

Noy A, de Vos S, Thieblemont C, Martin P, Flowers CR, Morschhauser F, et al. Targeting Bruton tyrosine kinase with ibrutinib in relapsed/refractory marginal zone lymphoma. Blood 2017; 129(16):2224-2232.

Oluwole O, Bouabdallah K, Muñoz J, Guibert S, Vose J, Bartlett N, et al. Abstract 70 - Prophylactic Steroid Use with Axicabtagene Ciloleucel in Patients with Relapsed/Refractory Large B-Cell Lymphoma. The 2021 TCT Meetings of ASTCT & CIBMTR 2021.

Pastore A, Jurinovic V, Kridel R, Hoster E, Staiger AM, Szczepanowski M, et al. Integration of gene mutations in risk prognostication for patients receiving first-line immunochemotherapy for follicular lymphoma: a retrospective analysis of a prospective clinical trial and validation in a population-based registry. Lancet Oncol 2015; 16(9):1111-1122.

POLIVY™ [Package Insert]. 2019; Genentech, Inc.

REVLIMID® [Package Insert]. 2019; Celgene Corporation.

Rummel MJ, Niederle N, Maschmeyer G, Banat GA, von Grünhagen U, Losem C, et al. Bendamustine plus rituximab versus CHOP plus rituximab as first-line treatment for patients

with indolent and mantle-cell lymphomas: an open-label, multicentre, randomised, phase 3 non-inferiority trial. Lancet 2013; 381(9873):1203-1210.

Salar A, Domingo-Domenech E, Panizo C, Nicolás C, Bargay J, Muntañola A, et al. First-line response-adapted treatment with the combination of bendamustine and rituximab in patients with mucosa-associated lymphoid tissue lymphoma (MALT2008-01): a multicentre, single-arm, phase 2 trial. Lancet Haematol 2014; 1(3):e104-111.

Sampson HA, Munoz-Furlong A, Campbell RL, Adkinson NF, Jr., Bock SA, Branum A, et al. Second symposium on the definition and management of anaphylaxis: summary report--Second National Institute of Allergy and Infectious Disease/Food Allergy and Anaphylaxis Network symposium. J Allergy Clin Immunol 2006; 117(2):391-397.

Schmitz R, Wright GW, Huang DW, Johnson CA, Phelan JD, Wang JQ, et al. Genetics and Pathogenesis of Diffuse Large B-Cell Lymphoma. N Engl J Med 2018; 378(15):1396-1407.

Schuster SJ, Bishop MR, Tam CS, Waller EK, Borchmann P, McGuirk JP, et al. Tisagenlecleucel in Adult Relapsed or Refractory Diffuse Large B-Cell Lymphoma. N Engl J Med 2019; 380(1):45-56.

Sehn LH, Berry B, Chhanabhai M, Fitzgerald C, Gill K, Hoskins P, et al. The revised International Prognostic Index (R-IPI) is a better predictor of outcome than the standard IPI for patients with diffuse large B-cell lymphoma treated with R-CHOP. Blood 2007; 109(5):1857-1861.

Short NJ, Kantarjian HM, Ko H, Khoury JD, Ravandi F, Thomas DA, et al. Outcomes of adults with relapsed or refractory Burkitt and high-grade B-cell leukemia/lymphoma. Am J Hematol 2017; 92(6):E114-e117.

Siegel RL, Miller KD,Jemal A. Cancer statistics, 2019. CA Cancer J Clin 2019; 69(1):7-34.

Smith DG,Williams SJ. Immune sensing of microbial glycolipids and related conjugates by T cells and the pattern recognition receptors MCL and Mincle. Carbohydr Res 2016; 420:32-45.

Smith EJ, Olson K, Haber LJ, Varghese B, Duramad P, Tustian AD, et al. A novel, native-format bispecific antibody triggering T-cell killing of B-cells is robustly active in mouse tumor models and cynomolgus monkeys. Sci Rep 2015; 5:17943.

Sorigue M, Ribera JM, Motllo C,Sancho JM. New drugs for follicular lymphoma. Leuk Res 2016; 49:38-46.

Swerdlow SH, Campo E, Harris NL, Jaffe ES, Pileri SA, Stein HT, J., et al. *WHO Classification of Tumours of Haematopoietic and Lymphoid Tissues, Fourth Edition*
Geneva, Switzerland: IARC Press; 2017.

Taplitz RA, Kennedy EB, Bow EJ, Crews J, Gleason C, Hawley DK, et al. Antimicrobial Prophylaxis for Adult Patients With Cancer-Related Immunosuppression: ASCO and IDSA Clinical Practice Guideline Update. J Clin Oncol 2018; 36(30):3043-3054.

Teachey DT, Rheingold SR, Maude SL, Zugmaier G, Barrett DM, Seif AE, et al. Cytokine release syndrome after blinatumomab treatment related to abnormal macrophage activation and ameliorated with cytokine-directed therapy. Blood 2013; 121(26):5154-5157.

Teras LR, DeSantis CE, Cerhan JR, Morton LM, Jemal A,Flowers CR. 2016 US lymphoid malignancy statistics by World Health Organization subtypes. CA Cancer J Clin 2016; 66(6):443-459.

Topp M, Van Meerten T, Houot R, Minnema MC, Milpied N, Lugtenburg PJ, et al. Earlier Steroid Use with Axicabtagene Ciloleucel (Axi-Cel) in Patients with Relapsed/Refractory Large B Cell Lymphoma. Blood 2019; 134(Supplement_1):243-243.

Wagner-Johnston ND, Link BK, Byrtek M, Dawson KL, Hainsworth J, Flowers CR, et al. Outcomes of transformed follicular lymphoma in the modern era: a report from the National LymphoCare Study (NLCS). Blood 2015; 126(7):851-857.

Wang ML, Blum KA, Martin P, Goy A, Auer R, Kahl BS, et al. Long-term follow-up of MCL patients treated with single-agent ibrutinib: updated safety and efficacy results. Blood 2015; 126(6):739-745.

YESCARTA™ [Package Insert]. 2017; Kite Pharma, Inc.

ZYDELIG®. http://www.gilead.com/~/media/Files/pdfs/medicines/oncology/zydelig/zydelig_pi.pdf: Gilead Sciences, Inc.; 2018.^

# 23. INVESTIGATOR'S AGREEMENT

I have read the attached protocol: "An Open-Label Study to Assess the Anti-Tumor Activity and Safety of REGN1979, an anti-CD20 x anti-CD3 Bispecific Antibody, in Patients with Relapsed or Refractory B-cell non-Hodgkin Lymphoma " and agree to abide by all provisions set forth therein.

I agree to comply with the current International Council for Harmonisation Guideline for Good Clinical Practice and the laws, rules, regulations, and guidelines of the community, country, state, or locality relating to the conduct of the clinical study.

I also agree that persons debarred from conducting or working on clinical studies by any court or regulatory agency will not be allowed to conduct or work on studies for the sponsor or a partnership in which the sponsor is involved. I will immediately disclose it in writing to the sponsor if any person who is involved in the study is debarred, or if any proceeding for debarment is pending, or, to the best of my knowledge, threatened.

This document contains confidential information of the sponsor, which must not be disclosed to anyone other than the recipient study staff and members of the EC. I agree to ensure that this information will not be used for any purpose other than the evaluation or conduct of the clinical investigation without the prior written consent of the sponsor.

______________________________________ ______________________________

(Signature of Investigator) (Date)

______________________________________

(Printed Name)

# APPENDIX 1. EASTERN COOPERATIVE ONCOLOGY GROUP CRITERIA

| Grade | Description |
|---|---|
| 0 | Fully active, able to carry on all pre-disease performance without restriction |
| 1 | Restricted in physically strenuous activity but ambulatory and able to carry out work of a light or sedentary nature, eg, light house work, office work |
| 2 | Ambulatory and capable of all self-care but unable to carry out any work activities. Up and about more than 50% of waking hours |
| 3 | Capable of only limited self-care, confined to bed or chair more than 50% of waking hours |
| 4 | Completely disabled. Cannot carry on any self-care. Totally confined to bed or chair |
| 5 | Dead |

# APPENDIX 2. MALIGNANT LYMPHOMA RESPONSE DEFINITIONS PER LUGANO CRITERIA

For CT/MRI-based response, up to 6 of the largest nodal and extranodal lesions that are measurable in 2 diameters (longest diameter [LDi] and shortest diameter) should be selected as target lesions from different body regions representative of the patient's overall disease burden and include mediastinal and retroperitoneal disease, if involved. A measurable nodal lesion must have an LDi greater than 1.5 cm. A measurable extranodal lesion must have an LDi greater than 1.0 cm. All other disease consistent with lymphoma, but not selected as target lesions (including nodal, extra nodal, and assessable disease), should be followed as non-measurable disease. All areas of disease should be evaluated at each tumor assessment time point as per the Lugano criteria (Cheson 2014).

| Response and Site | PET-CT–Based Response | CT-Based Response |
|---|---|---|
| Complete | Complete metabolic response | Complete radiologic response (all of the following) |
| Lymph nodes and extralymphatic sites | Score 1, 2, or 3* with or without a residual mass on 5PS† | Target nodes/nodal masses must regress to ≤ 1.5 cm in LDi |
| | It is recognized that in Waldeyer's ring or extranodal sites with high physiologic uptake or with activation within spleen or marrow (eg, with chemotherapy or myeloid colony-stimulating factors), uptake may be greater than normal mediastinum and/or liver. In this circumstance, complete metabolic response may be inferred if uptake at sites of initial involvement is no greater than surrounding normal tissue even if the tissue has high physiologic uptake | No extralymphatic sites of disease |
| Nonmeasured lesion | Not applicable | Absent |
| Organ enlargement | Not applicable | Regress to normal |
| New lesions | None | None |
| Bone marrow | No evidence of FDG-avid disease in marrow | Normal by morphology; if indeterminate, IHC negative |
| Partial | Partial metabolic response | Partial remission (all of the following) |
| Lymph nodes and extralymphatic sites | Score 4 or 5† with reduced uptake compared with baseline and residual mass(es) of any size | ≥ 50% decrease in SPD of up to 6 target measurable nodes and extranodal sites |
| | At interim, these findings suggest responding disease | When a lesion is too small to measure on CT, assign 5 mm × 5 mm as the default value |
| | At end of treatment, these findings indicate residual disease | When no longer visible, 0 × 0 mm |
| | | For a node > 5 mm × 5 mm, but smaller than normal, use actual measurement for calculation |
| Nonmeasured lesions | Not applicable | Absent/normal, regressed, but no increase |
| Organ enlargement | Not applicable | Spleen must have regressed by > 50% in length beyond normal |
| New lesions | None | None |
| Bone marrow | Residual uptake higher than uptake in normal marrow but reduced compared with baseline (diffuse uptake compatible with reactive changes from chemotherapy allowed). If there are persistent focal changes in the marrow in the context of a nodal response, consideration should be given to further evaluation with MRI or biopsy or an interval scan | Not applicable |

| Response and Site | PET-CT–Based Response | CT-Based Response |
|---|---|---|
| No response or stable disease | No metabolic response | Stable disease |
| Target nodes/nodal masses, extranodal lesions | Score 4 or 5 with no significant change in FDG uptake from baseline at interim or end of treatment | < 50% decrease from baseline in SPD of up to 6 dominant, measurable nodes and extranodal sites; no criteria for progressive disease are met |
| Nonmeasured lesions | Not applicable | No increase consistent with progression |
| Organ enlargement | Not applicable | No increase consistent with progression |
| New lesions | None | None |
| Bone marrow | No change from baseline | Not applicable |
| Progressive disease | Progressive metabolic disease | Progressive disease requires at least 1 of the following |
| Individual target nodes/nodal masses | Score 4 or 5 with an increase in intensity of uptake from baseline and/or | PPD progression: |
| Extranodal lesions | New FDG-avid foci consistent with lymphoma at interim or end-of-treatment assessment | An individual node/lesion must be abnormal with:<br>LDi > 1.5 cm and<br>Increase by ≥ 50% from PPD nadir and<br>An increase in LDi or SDi from nadir<br>0.5 cm for lesions ≤ 2 cm<br>1.0 cm for lesions > 2 cm<br>In the setting of splenomegaly, the splenic length must increase by > 50% of the extent of its prior increase beyond baseline (eg, a 15-cm spleen must increase to > 16 cm). If no prior splenomegaly, must increase by at least 2 cm from baseline<br>New or recurrent splenomegaly |
| Nonmeasured lesions | None | New or clear progression of preexisting nonmeasured lesions |
| New lesions | New FDG-avid foci consistent with lymphoma rather than another etiology (eg, infection, inflammation). If uncertain regarding etiology of new lesions, biopsy or interval scan may be considered | Regrowth of previously resolved lesions<br>A new node > 1.5 cm in any axis<br>A new extranodal site > 1.0 cm in any axis; if < 1.0 cm in any axis, its presence must be unequivocal and must be attributable to lymphoma<br>Assessable disease of any size unequivocally attributable to lymphoma |
| Bone marrow | New or recurrent FDG-avid foci | New or recurrent involvement |

Abbreviations: 5PS, 5-point scale; CT, computed tomography; FDG, fluorodeoxyglucose; IHC, immunohistochemistry; LDi, longest transverse diameter of a lesion; MRI, magnetic resonance imaging; PET, positron emission tomography; PPD, cross product of the LDi and perpendicular diameter; SDi, shortest axis perpendicular to the LDi; SPD, sum of the product of the perpendicular diameters for multiple lesions.

*A score of 3 in many patients indicates a good prognosis with standard treatment, especially if at the time of an interim scan. However, in trials involving PET where de-escalation is investigated, it may be preferable to consider a score of 3 as inadequate response (to avoid undertreatment). Measured dominant lesions: Up to six of the largest dominant nodes, nodal masses, and extranodal lesions selected to be clearly measurable in two diameters. Nodes should preferably be from disparate regions of the body and should include, where applicable, mediastinal and retroperitoneal areas. Non-nodal lesions include those in solid organs (eg, liver, spleen, kidneys, lungs), GI involvement, cutaneous lesions, or those noted on palpation. Nonmeasured lesions: Any disease not selected as measured, dominant disease and truly assessable disease should be considered not measured. These sites include any nodes, nodal masses, and extranodal sites not selected as dominant or measurable or that do not meet the requirements for measurability but are still considered abnormal, as well as truly assessable disease, which is any site of suspected disease that would be difficult to follow quantitatively with measurement, including pleural effusions, ascites, bone lesions, leptomeningeal disease, abdominal masses, and other lesions that cannot be confirmed and followed by imaging. In Waldeyer's ring or in extranodal sites (eg, GI tract, liver, bone marrow), FDG uptake may be greater than in the mediastinum with complete metabolic response, but should be no higher than surrounding normal physiologic uptake (eg, with marrow activation as a result of chemotherapy or myeloid growth factors).

†PET 5PS: 1, no uptake above background; 2, uptake ≤ mediastinum; 3, uptake > mediastinum but ≤ liver; 4, uptake moderately > liver; 5, uptake markedly higher than liver and/or new lesions; X, new areas of uptake unlikely to be related to lymphoma.

# APPENDIX 3. LYMPHOMA RESPONSE TO IMMUNOMODULATORY THERAPY CRITERIA (LYRIC) CRITERIA

## Definition

A patient will be considered to have "indeterminate response" (IR) in 1 or more of the 3 following circumstances.

***1. Increase in overall tumor burden (as assessed by sum of the product of the diameters [SPD]) of ≥50% of up to 6 measurable lesions in the first 12 weeks of therapy, without clinical deterioration [IR(1)].*** This pattern may be seen as a consequence of either delayed response or early immune-mediated flare. At least within the context of clinical trials, a biopsy is encouraged in this case because this may help to distinguish the 2 and, if positive, will confirm the impression of PD. However, if negative for lymphoma, it will support the concept of pseudo progression and contribute to our understanding of this phenomenon. When such a biopsy is neither safe nor feasible, decisions must be based on a repeat scan 12 weeks after the initial determination of IR. It is recognized that "clinical deterioration" is subjective. In some cases, the simple growth of a nodal or tumor mass could worsen the symptoms mechanically related to that mass, such as pain at the tumor site, compression of adjacent structures, etc. Such an increase in symptoms that can be directly attributed to the size of the tumor mass may not be considered as clinical deterioration in this context. However, in most cases, patients should be experiencing clinical stability or improvement by investigator assessment to be considered as having IR, and in all cases, the patient must be considered likely to tolerate continued treatment and not at risk of serious complications should further tumor growth occur.

***2. Appearance of new lesions or growth of one or more existing lesion(s) ≥50% at any time during treatment; occurring in the context of lack of overall progression (<50% increase) of overall tumor burden, as measured by SPD of up to 6 lesions at any time during the treatment [IR(2)].*** This phenomenon may occur early or late in the treatment course, and therefore, unlike IR(1), is not defined by its temporal relationship to treatment initiation. Both within and outside the context of clinical trials, a biopsy is strongly encouraged in such cases. If the biopsy does not confirm the presence of viable tumor in the new or enlarging lesion(s), then the lesion(s) are not considered active disease and should not be used in subsequent SPD assessments.

***3. Increase in FDG uptake of 1 or more lesion(s) without a concomitant increase in lesion size or number [IR(3)]*** Increased immune activity at the site of tumor may manifest as an increase in FDG uptake. Therefore, by itself, changes in uptake should not trigger an assignment of PD with checkpoint inhibitors. The magnitude of increase in uptake in an immune-mediated flare compared with that in true tumor progression is not yet known. It is important to investigate this finding, especially in conjunction with biopsies of the lesion in question. While awaiting a better characterization of this phenomenon, we propose that, under the modified response criteria, an increase in FDG avidity of 1 or more lesions suggestive of lymphoma, without a concomitant increase in size of those lesions meeting PD criteria does not constitute PD.

It is possible that, at a single time point, a patient could fulfill criteria for both [IR(1)] or [IR(2)] and [IR(3)]: for example, there could be a new FDG avid lesion in the absence of overall progression [IR(2)], and, at the same time, increase in FDG uptake of a separate lesion [IR(3)]. In such cases, the designation of [IR(1 or 2)] should take priority [eg, IR (2)] in the above example].

# SIGNATURE OF SPONSOR'S RESPONSIBLE OFFICERS

**(Scientific/Medical Monitor, Regulatory Representative, Clinical Study Team Lead, and Biostatistician)**

*To the best of my knowledge, this protocol accurately describes the conduct of the study.*

| | |
|---|---|
| Study Title: | An Open-Label Study to Assess the Anti-Tumor Activity and Safety of REGN1979, an anti-CD20 x anti-CD3 Bispecific Antibody, in Patients with Relapsed or Refractory B-cell non-Hodgkin Lymphoma |
| Protocol Number: | R1979-ONC-1625 |
| Protocol Version: | R1979-ONC-1625 Amendment 5 Global |

*See appended electronic signature page*

Sponsor's Responsible Scientific/Medical Monitor

*See appended electronic signature page*

Sponsor's Responsible Regulatory Representative

*See appended electronic signature page*

Sponsor's Responsible Clinical Study Team Lead

*See appended electronic signature page*

Sponsor's Responsible Biostatistician

Signature Page for VV-RIM-00192456 v1.0

| | |
|---|---|
| Approval/eSignature | PPD<br>PPD<br>11-Apr-2022 17:45:10 GMT+0000 |

| | |
|---|---|
| Approval/eSignature | PPD<br>PPD<br>11-Apr-2022 17:49:39 GMT+0000 |

| | |
|---|---|
| Approval/eSignature | PPD<br>PPD<br>11-Apr-2022 18:21:52 GMT+0000 |

| | |
|---|---|
| Approval/eSignature | PPD<br>PPD<br>11-Apr-2022 18:59:26 GMT+0000 |

Signature Page for VV-RIM-00192456 v1.0 Approved

Clinical Development and Regulatory Affairs
Biostatistics and Data Management

# STATISTICAL ANALYSIS PLAN
# VERSION: FINAL

AN OPEN-LABEL STUDY TO ASSESS THE ANTI-TUMOR ACTIVITY AND SAFETY OF REGN1979, AN ANTI-CD20 X ANTI-CD3 BISPECIFIC ANTIBODY, IN PATIENTS WITH RELAPSED OR REFRACTORY B-CELL NON-HODGKIN LYMPHOMA

| | |
|---|---|
| Compound: | REGN1979 |
| Protocol Number: | R1979-ONC-1625 |
| Clinical Phase: | Phase 2 |
| Sponsor: | Regeneron Pharmaceuticals, Inc. |
| Study Biostatistician: | PPD |
| Clinical Trial Manager: | PPD |
| Study Medical Director: | PPD |
| Version/Date: | Original Statistical Analysis Plan / December 17, 2021 |

**The approval signatures below indicate that these individuals have reviewed the Statistical Analysis Plan (SAP) and agreed on the planned analysis defined in this document for reporting.**

*See appended electronic signature page*

Study Biostatistician (PPD)

*See appended electronic signature page*

Study Clinical Pharmacologist (PPD)

*See appended electronic signature page*

Study Bioanalytical Sciences (PPD)

*See appended electronic signature page*

Study Medical Director PPD

*See appended electronic signature page*

Project Biostatistician PPD

*See appended electronic signature page*

Head of BDM or designee PPD

# TABLE OF CONTENTS

LIST OF ABBREVIATIONS AND DEFINITIONS OF TERMS ........8
1. OVERVIEW ........10
1.1. Background/Rationale ........10
1.2. Study Objectives ........10
1.2.1. Primary Objectives ........10
1.2.2. Secondary Objectives ........10
1.2.3. Exploratory Objectives ........11
1.2.4. Modifications from the Statistical Section in the Final Protocol ........12
1.2.5. Revision History for SAP Amendments ........12
2. INVESTIGATION PLAN ........13
2.1. Study Design and Randomization ........13
2.2. Sample Size and Power Considerations ........14
2.2.1. Follicular Lymphoma Grade 1-3a Cohort ........14
2.2.2. Diffuse Large B-Cell Lymphoma ........15
2.2.3. Mantle Cell Lymphoma after BTK inhibitor therapy Cohort ........16
2.2.4. Marginal Zone Lymphoma Cohort ........16
2.2.5. Other B-Cell non-Hodgkin Lymphoma Cohort ........17
3. ANALYSIS POPULATIONS ........18
3.1. Full Analysis Set (FAS) ........18
3.2. Safety Analysis Set (SAF) ........18
3.3. Pharmacokinetic Analysis Set ........18
3.4. Immunogenicity Analysis Set ........18
4. ANALYSIS VARIABLES ........19
4.1. Demographic and Baseline Characteristics ........19
4.2. Medical History ........19
4.3. Pre-Treatment / Concomitant Medication ........19
4.4. Prohibited Medication During Study ........20
4.5. Efficacy Variable ........20
4.5.1. Primary Efficacy Variable (s) ........20
4.5.2. Secondary Efficacy Variable(s) ........20
4.5.2.1. Objective Response Rate per local investigator ........20



VV-RIM-00187995-1.0 Approved - 01 Mar 2022 GMT-5:00

4.5.2.2. Complete Response Rate ....20
4.5.2.3. Progression-Free Survival ....21
4.5.2.4. Overall Survival ....21
4.5.2.5. Duration of Response ....21
4.5.2.6. Duration of Complete Response ....21
4.5.2.7. Duration of Partial Response ....22
4.5.2.8. Disease Control Rate ....22
4.5.2.9. Time to Response ....22
4.5.2.10. Time to new anti-lymphoma therapy ....22
4.6. Safety Variables ....22
4.6.1. Adverse Events and Serious Adverse Events ....22
4.6.2. Adverse Events of Special Interest ....23
4.6.3. Laboratory Safety Variables ....24
4.6.4. Vital Signs ....24
4.6.5. 12-Lead Electrocardiography (ECG) ....24
4.6.6. Physical Examination Variables ....24
4.7. Pharmacokinetic Variables ....24
4.8. Immunogenicity Variables ....24
4.9. Biomarker Variables ....24
4.10. Patient-Reported Outcomes Variables ....24
4.10.1. EORTC QLQ-C30 ....25
4.10.2. FACT-Lym ....25
4.10.3. EQ-5D-3L ....25
5. STATISTICAL METHODS ....26
5.1. Demographics and Baseline Characteristics ....26
5.2. Medical History ....27
5.3. Prior/concomitant Illnesses and Medications/Procedures ....27
5.3.1. Prior Lymphoma-related cancer systemic therapies ....27
5.3.2. Prior Lymphoma-related cancer radiotherapy ....28
5.3.3. Prior Lymphoma-related cancer surgery ....28
5.3.4. Prior Hematopoietic stem cell transplants ....28
5.4. Prohibited Medications ....28



VV-RIM-00187995-1.0 Approved - 01 Mar 2022 GMT-5:00

5.5. Subject Disposition ....28
5.6. Extent of Study Treatment Exposure and Compliance ....28
5.6.1. Measurement of Compliance ....29
5.6.2. Exposure to Investigational Product ....29
5.6.3. Modifications of Study Treatment ....29
5.6.3.1. Dose Delays ....29
5.6.3.2. Infusion Interruptions ....30
5.6.3.3. Dose Modifications ....30
5.7. Analyses of Efficacy Variables ....30
5.7.1. Analysis of Primary Efficacy Variable(s) ....30
5.7.2. Analysis of Secondary Efficacy Variables ....32
5.7.2.1. Analysis of ORR per Investigator ....32
5.7.2.2. Analysis of Complete Response Rate ....32
5.7.2.3. Analysis of Progression-Free Survival ....32
5.7.2.4. Analysis of Overall Survival ....32
5.7.2.5. Analysis of Duration of Response ....32
5.7.2.6. Analysis of Duration of Complete Response ....32
5.7.2.7. Analysis of Duration of Partial Response ....33
5.7.2.8. Analysis of Disease Control Rate ....33
5.7.2.9. Analysis of Time to Response ....33
5.7.2.10. Analysis of Time to Anti-Lymphoma Therapy ....33
5.7.3. Adjustment for Multiple Comparison ....33
5.7.4. Analysis of Patient-Reported Outcomes Data ....33
5.7.4.1. Patient Disposition ....33
5.7.4.2. Instrument Completion Rates ....34
5.7.4.3. Descriptive Summary Statistics by Visit ....34
5.7.4.4. Longitudinal Analysis of Change from Baseline ....34
5.7.4.5. Time to definitive deterioration (TTDD) ....34
5.8. Analysis of Safety Data ....35
5.8.1. Adverse Events ....35
5.8.2. Clinical Laboratory Measurements ....37
5.8.3. Analysis of Vital Signs ....37



VV-RIM-00187995-1.0 Approved - 01 Mar 2022 GMT-5:00

5.8.4. Analysis of 12-Lead ECG ....38
5.8.5. Physical Exams ....38
5.8.6. Analysis of Drug Concentration Data ....38
5.8.7. Analysis of Immunogenicity Data ....38
5.8.7.1. Analysis of ADA Data ....38
5.8.7.2. Analysis of Neutralizing Antibody (NAb) Data ....39
5.8.8. Analysis of Biomarkers ....40
5.8.9. Pharmacokinetic/Pharmacodynamic Analyses ....40
5.8.10. Exposure-Response Analysis ....40
5.9. Association of Immunogenicity with Exposure, Safety, and Efficacy ....41
5.9.1. Immunogenicity and Exposure ....41
5.9.2. Immunogenicity and Safety and Efficacy ....41
6. DATA CONVENTIONS ....42
6.1. Definition of Study Periods for Efficacy/Safety Variables ....42
6.2. Data Handling Convention for Efficacy Variables ....42
6.3. Data Handling Convention for Missing Data ....42
6.4. Visit Windows ....44
6.5. Unscheduled Assessments ....44
6.6. Pooling of Centers for Statistical Analyses ....44
6.7. Statistical Technical Issues ....44
7. INTERIM ANALYSIS ....45
8. SOFTWARE ....46
9. REFERENCES ....47
10. APPENDIX ....48
10.1. Summary of Statistical Analyses ....48
10.2. Schedule of Time and Events ....50



VV-RIM-00187995-1.0 Approved - 01 Mar 2022 GMT-5:00

# LIST OF TABLES

Table 1: The 2-sided 95% Exact Confidence Intervals for Observed ORR Given a Sample Size of 112 Patients ....15

Table 2: The 2-sided 95% Exact Confidence Intervals for Observed ORR Given a Sample Size of 78 Patients ....16

Table 3: The 2-sided 95% Exact Confidence Intervals for Observed ORR Given a Sample Size of 67 Patients ....17

Table 4: Sample Size at Original and Modified Step-up Regimens in Each Cohort ....17

Table 5: Minimal Clinically Important Difference (MCID) ....35

Table 6: Interim Futility Analysis ....45

# LIST OF FIGURES

Figure 1: Study Design Schematic ....13

# LIST OF ABBREVIATIONS AND DEFINITIONS OF TERMS

| | |
|---|---|
| ADA | Anti-drug antibody |
| AE | Adverse event |
| AESI | Adverse event of special interest |
| B-NHL | B-cell non-Hodgkin lymphoma |
| BOR | Best Overall Response |
| BTK | Bruton's tyrosine kinase |
| CR | Complete response |
| CRF | Case report form (electronic or paper) |
| CRP | C-reactive protein |
| CRS | Cytokine release syndrome |
| CT | Computer tomography |
| CTCAE | Common Terminology Criteria for Adverse Events |
| DCR | Disease control rate |
| DLBCL | Diffuse large B-cell lymphoma |
| DOR | Duration of response |
| ECG | Electrocardiogram |
| ECOG | Eastern Cooperative Oncology Group |
| EORTC QLQ-C30 | European Organisation for Research and Treatment of Cancer Quality of Life Questionnaire |
| EQ-5D-3L | EuroQoL 5 Dimensions 3 Levels |
| E-R | Exposure-response |
| FACT-Lym | Functional Assessment of Cancer Therapy–Lymphoma |
| FAS | Full analysis set |
| FL | Follicular lymphoma |
| FLIPI | Follicular Lymphoma International Prognostic Index |
| HRQL | Health-related Quality of Life |
| HSCT | Haematopoietic stem cell transplantation |
| ICH | International Council for Harmonisation |
| IRC | Independent Central Reviewers |
| IRR | Infusion-related reaction |
| IV | Intravenous |
| MCL | Mantle Cell Lymphoma |
| MedDRA | Medical Dictionary for Regulatory Activities |
| MIPI | MCL International Prognostic Index |
| MRD | Minimal residual disease |

| | |
|---|---|
| MZL | Marginal Zone Lymphoma |
| NAb | Neutralizing antibody |
| NCI-CTCAE | National Cancer Institute-Common Terminology Criteria for Adverse Events |
| NHL | Non-Hodgkin lymphoma |
| ORR | Objective response rate |
| OS | Overall survival |
| PBMC | Peripheral blood mononuclear cell |
| PD | Progressive disease |
| PE | Physical exam |
| PET-CT | Positron emission tomography |
| PFS | Progression-free survival |
| PI3K | Phosphatidylinositol 3-kinase |
| PK | Pharmacokinetic |
| POD24 | Progression of disease within 2 years |
| PR | Partial response |
| PT | Preferred term |
| QW | Once every week |
| Q2W | Once every 2 weeks |
| Q4W | Once every 4 weeks |
| Regeneron | Regeneron Pharmaceuticals, Inc. |
| SAE | Serious adverse event |
| SAF | Safety analysis set |
| SAP | Statistical Analysis Plan |
| SAS | Statistical Analysis System software |
| SD | Stable disease |
| SOC | System organ class |
| TEAE | Treatment-emergent adverse event |
| US | United States |
| WHO | World Health Organization |

# 1. OVERVIEW

The purpose of the statistical analysis plan (SAP) is to ensure the credibility of the study results by pre-specifying the statistical approaches for the analysis of study data prior to database lock. The SAP is intended to be a comprehensive and detailed description of the strategy and statistical methods to be used in the analysis of data for R1979-ONC-1625 study.

This SAP version is based on the global protocol amendment 4. This plan may be revised during the study to accommodate protocol amendments and/or to make changes to adapt to unexpected issues in study execution and/or data that affect planned analyses.

## 1.1. Background/Rationale

R1979-ONC-1625 is as an open-label, single-arm, multi-center, phase 2 study of odronextamab in patients with 5 disease-specific B-NHL (B-cell non-Hodgkin lymphoma) cohorts: follicular lymphoma (FL) grade 1-3a, diffuse large B-cell lymphoma (DLBCL), mantle cell lymphoma (MCL), marginal zone lymphoma (MZL), and other B-NHL (containing lymphoma subtypes not included in the other 4 cohorts) that have relapsed after or are refractory to at least 2 prior lines of systemic therapy, including an anti-CD20 antibody and an alkylating agent.

## 1.2. Study Objectives

### 1.2.1. Primary Objectives

The primary objective of this study is to assess the anti-tumor activity of single agent odronextamab as measured by the objective response rate (ORR) according to the Lugano classification of response in malignant lymphoma (Cheson, 2014) as assessed by independent central review in each of the following B-NHL disease specific cohorts.

- In patients with follicular lymphoma (FL) grade 1-3a that have relapsed after or are refractory to at least 2 prior lines of systemic therapy, including an anti-CD20 antibody and an alkylating agent.
- In patients with diffuse large B-cell lymphoma (DLBCL) that have relapsed after or are refractory to at least 2 prior lines of systemic therapy, including an anti-CD20 antibody and an alkylating agent.
- In patients with mantle cell lymphoma (MCL) that have relapsed after or are refractory to a BTK inhibitor. This cohort also includes patients who have relapsed or have disease refractory to prior systemic therapy, or patients who have demonstrated intolerance to BTK inhibitor therapy, and who have progressed after other systemic therapy.
- In patients with marginal zone lymphoma (MZL) that have relapsed after or are refractory to at least 2 prior line of systemic therapy.
- In patients with other B-NHL subtypes that have relapsed after or are refractory to at least 2 prior lines of systemic therapy.

### 1.2.2. Secondary Objectives

The secondary objectives of this study are:



VV-RIM-00187995-1.0 Approved - 01 Mar 2022 GMT-5:00

- To assess the anti-tumor activity of single agent odronextamab in each of the above 5 disease specific cohorts, as measured by:
  - ORR according to the Lugano Classification as assessed by local investigator evaluation
  - Complete response (CR) rate according to the Lugano Classification as assessed by independent central review and local investigator evaluation
  - Progression free survival (PFS) according to Lugano Classification as assessed by independent central review and local investigator evaluation
  - Overall survival (OS)
  - Duration of response (DOR) according to the Lugano Classification as assessed by independent central review and local investigator evaluation
  - Disease control rate (DCR) according to the Lugano Classification as assessed by independent central review and local investigator evaluation
- To evaluate the safety and tolerability of odronextamab
- To assess the pharmacokinetics (PK) of odronextamabTo assess the immunogenicity of odronextamab
- To assess the effect of odronextamab on patient reported outcomes, including health-related quality of life (HRQL), as measured by the validated instruments European Organisation for Research and Treatment of Cancer Quality of Life Questionnaire (EORTC QLQ-C30), Functional Assessment of Cancer Therapy-Lymphoma (FACT-Lym), and EuroQoL 5 Dimensions 3 Levels (EQ-5D-3L)

### 1.2.3. Exploratory Objectives

Exploratory objects of this study are:

- To assess changes in serum C-reactive protein (CRP), ferritin, and cytokine levels in patients treated with odronextamab, and to assess the relationship between cytokine increases during study drug treatment, measures of efficacy, the incidence of adverse events (AEs), and relationship to tumor burden and location
- To characterize the rate of undetectable minimal residual disease (MRD) status over the course of the treatment
- To evaluate blood pharmacodynamic biomarkers potentially related to the mechanism of action of odronextamab, including the activation and distribution of peripheral blood mononuclear cell (PBMC) subsets
- To evaluate markers of T-cell activation over time
- To evaluate molecular and cellular profile of malignant lymph node tissue at baseline on treatment and at disease progression (e.g., B cell CD20 expression, T cell subsets and activation markers, DNA and RNA sequence profile)



VV-RIM-00187995-1.0 Approved - 01 Mar 2022 GMT-5:00

- To evaluate the relationship between PK and pharmacodynamic measures for relevant biomarkers and concentration-response (C-R) relationships for efficacy endpoints

### 1.2.4. Modifications from the Statistical Section in the Final Protocol

The sponsor will perform an administrative review for efficacy after at least 70 patients have been treated with the 0.7/4/20 step-up regimen and have cleared a 4-week safety review. At the administrative review, summary of safety for all cohorts and summary of efficacy for the FL grade 1-3a and DLBCL cohorts will be provided.

### 1.2.5. Revision History for SAP Amendments

This is the first version of the SAP.

# 2. INVESTIGATION PLAN

## 2.1. Study Design and Randomization

This is a phase 2, open-label, multi-cohort, multi-center study of odronextamab administered as an IV (Intravenous) infusion to patients with B-NHL that have relapsed or are refractory to prior systemic therapy. The study consists of 5 disease-specific cohorts, each with independent parallel enrollment. Cohort assignments will occur based on the patient's diagnosis and treatment history at the time of enrollment (Figure 1).

**Figure 1: Study Design Schematic**

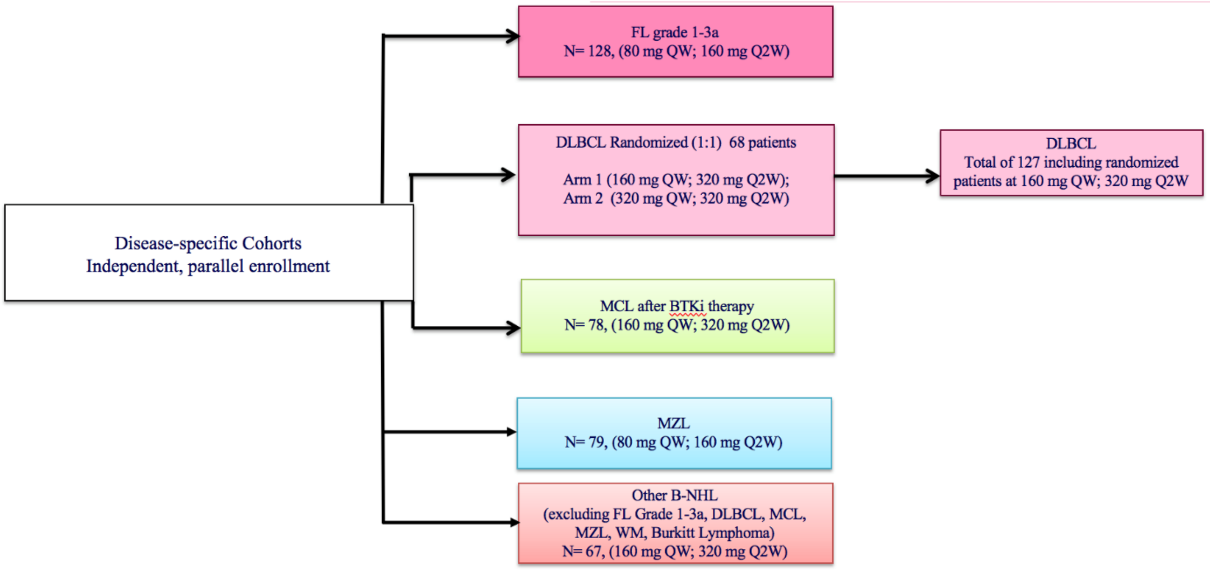


Patients in the DLBCL cohort will be randomized 1:1 to either Arm 1 (160 mg QW dosing followed by 320 mg Q2W dosing) or Arm 2 (320 mg QW dosing followed by 320 mg Q2W dosing) in the initial step of this cohort.

After the initial step, enrollment will only continue in the 160 mg QW/ 320 mg Q2W arm until a total of 112 patients are reached for that dose level (but can be increased up to 127 patients, including the randomized patients in the arm, based on the number of patients required for safety evaluation of revised step-up dose regimen).

New enrollment is paused for the MCL cohort as of global amendment 3 and for the MZL cohort as of global amendment 4. MCL and MZL patients who have received and tolerated the initial dose, intermediate doses, and first QW dose will continue to receive treatment if they are experiencing clinical benefit as per investigator.

Patients under the global protocol amendment 4 will be enrolled with a revised dosing regimen as follows: Each patient will receive odronextamab at an initial dose of 0.7 mg during treatment week 1(administered as a split dose of 0.2 mg and 0.5 mg), followed by an intermediate dose-1 of 4 mg at treatment week 2, intermediate dose-2 of 20 mg at treatment week 3 and then the assigned QW full dose at treatment week 4 through 12 followed by Q2W dose in FL grade 1-3a, DLBCL, and Other B-NHL cohorts simultaneously. The first cytokine release syndrome (CRS) interim safety assessment will be performed in the first 25 patients (FL grade 1-3a and

DLBCL/Other NHL combined) treated with the modified step-up regimen. Further enrollment in the study will be paused until the 25th patient has received the full QW dose or discontinued early.

If safety is cleared in the first CRS interim safety assessment, then parallel enrollment will resume. Enrollment will pause in the FL cohort once the 25th patient with FL grade 1-3a is enrolled and will pause in both the DLBCL and Other B-NHL cohort once the 25th patient with DLBCL or other B-NHL is enrolled. If safety is cleared in the second CRS interim safety assessment, then enrollment will resume until a total of 60 patients are enrolled in each respective cohort. If at any time a second patient in FL grade 1-3a and DLBCL/Other B-NHL cohorts combined experiences a grade ≥3 CRS event, the dose of oral dexamethasone (or equivalent) before and after odronextamab dosing days will be increased to 20 mg, and earlier tocilizumab intervention will be implemented (i.e., tocilizumab use for patients with grade 1 CRS of fever lasting >24 hours and for all patients with grade ≥2 CRS) for all ongoing and subsequently enrolled patients.

The treatment duration will comprise of 12 QW doses followed by Q2W dosing until the time of disease progression or other protocol-defined reason for treatment discontinuation. In addition, if a patient has demonstrated a CR and has shown a durable response for at least 9 months after the initial determination of CR, based on local investigator evaluation, then the frequency of study drug administration at the assigned dose will be decreased from Q2W to Q4W intervals.

## 2.2. Sample Size and Power Considerations

There is no formal statistical hypothesis in this study. The sample size is determined to preserve adequate probability of observing 95% CI lower bound excluding the minimum clinically meaningful ORR for each disease-specific cohort.

Up to a total of 512 patients are planned for enrollment and treatment across 5 disease-specific cohorts at approximately 130 sites within US, Canada, Europe, and Asia-Pacific regions. The sample size for each disease-specific cohort is:

- 128 patients with FL grade 1-3a
- 161 patients with DLBCL (68 randomized patients in Arm 1 and Arm 2, and a total of 112 patients (up to approximately 127 patients) including the randomized patients in the 160 mg QW/320 mg Q2W dose regimen)
- 78 patients with MCL after failure of BTK inhibitor therapy
- 78 patients with MZL
- 67 patients with other B-NHL subtypes

The details of calculation of sample size are provided in the following sections.

### 2.2.1. Follicular Lymphoma Grade 1-3a Cohort

A single-stage exact binomial design will be adopted for the primary endpoint of ORR. The 2-sided 95% confidence intervals for the observed ORRs were calculated based on a sample size of 112. Assuming that an ORR of greater than 49% is clinically meaningful; with 112 patients, an ORR of at least 59% will have a lower bound of the confidence interval that excludes 49%. In



VV-RIM-00187995-1.0 Approved - 01 Mar 2022 GMT-5:00

addition, if the observed ORR is at least 65%, 70%, and 75%, the lower limit of 95% CI will exclude the ORR of 55%, 60% and 65% respectively; i.e., the ORR is significantly different from 55%, 60%, and 65% (Table 1).

**Table 1: The 2-sided 95% Exact Confidence Intervals for Observed ORR Given a Sample Size of 112 Patients**

| Number of Responders | Observed ORR | 95%CI – lower | 95% CI – upper |
|---|---|---|---|
| 50 | 0.45 | 0.352 | 0.543 |
| 56 | 0.50 | 0.404 | 0.596 |
| 62 | 0.55 | 0.457 | 0.648 |
| 66 | 0.59 | 0.492 | 0.681 |
| 67 | 0.60 | 0.501 | 0.690 |
| 73 | 0.65 | 0.556 | 0.739 |
| 78 | 0.70 | 0.602 | 0.780 |
| 84 | 0.75 | 0.659 | 0.827 |

With a sample size of 112 patients, if the true treatment effect of odronextamab is 64%, 70%, 75%, or 80%, the probability is 89%, 89%, 92%, or 92%, respectively, for observed lower bound of 95% CI to exclude 49%, 55%, 60%, or 65%, respectively.

The enrollment in this cohort will continue beyond 112 patients in order to have at least 60 patients treated with the revised step-up regimen. With the additional patients, the probability stated above will be higher.

### 2.2.2. Diffuse Large B-Cell Lymphoma

This study cohort is designed to further evaluate the efficacy and safety of odronextamab for patients with DLBCL that has relapsed or is refractory to at least 2 prior lines of systemic therapy. As an initial step, 68 patients will be randomized into the two treatment arms with 1:1 ratio (Arm 1: 160 mg QW dosing followed by 320 mg Q2W dosing; Arm 2: 320 mg QW dosing followed by 320 mg Q2W dosing). Data from the initial step will be used for descriptive analyses that support PK/pharmacodynamic and advance our understanding of the exposure-response relationship. After the initial step, enrollment will only continue in the 160 mg QW/ 320 mg Q2W arm until a total of 112 patients (up to approximately 127 patients including the randomized patients in the arm) are reached to further study efficacy at this selected dose regimen.

An exact binomial design will be adopted for the primary endpoint of ORR. The 2-sided 95% confidence intervals for the observed ORR were calculated based on a sample size of 112. Assuming a clinical meaningful ORR is greater than 35%, with 112 patients, an ORR of at least 45% will have a lower bound of the confidence interval that excludes 35%. In addition, if the observed ORR is at least 50%, 55% and 60%, the lower limit of 95% CI will exclude the ORR of 40%, 45% and 50% respectively; i.e., the ORR is significantly different from 40%, 45% and 50% (Table 1).


VV-RIM-00187995-1.0 Approved - 01 Mar 2022 GMT-5:00

With a sample size of 112 patients, if the true treatment effect of odronextamab is 50%, 55%, 60% or 65%, the probability is 89%, 88%, 86% or 89% respectively, for observed lower bound of 95% CI to exclude 35%, 40%, 45% and 50%, respectively.

The enrollment in this cohort will continue beyond 112 patients in order to have at least 60 patients of DLBCL or Other aggressive B-NHL (minimum 45 DLBCL patients) treated with the revised step-up regimen. With the additional patients, the probability stated above will be higher.

### 2.2.3. Mantle Cell Lymphoma after BTK inhibitor therapy Cohort

An exact binomial design will be adopted for the primary endpoint ORR. The 2-sided 95% confidence intervals for the observed ORRs were calculated based on a sample size of 78. Assuming a clinical meaningful ORR is greater than 15%, with 78 patients, an ORR of at least 24% will have a lower bound of the confidence interval that excludes 15%. In addition, if the observed ORR is at least 36%, 41% and 47%, the lower limit of 95% CI will exclude the ORR of 25%, 30% and 36% respectively; i.e., the ORR rate is significantly different from 25%, 30% and 36% (Table 2).

**Table 2: The 2-sided 95% Exact Confidence Intervals for Observed ORR Given a Sample Size of 78 Patients**

| Number of Responders | Observed ORR | 95%CI – lower | 95% CI – upper |
|---|---|---|---|
| 19 | 0.24 | 0.153 | 0.354 |
| 24 | 0.31 | 0.208 | 0.422 |
| 28 | 0.36 | 0.253 | 0.476 |
| 32 | 0.41 | 0.3001 | 0.527 |
| 37 | 0.47 | 0.3601 | 0.591 |
| 41 | 0.53 | 0.409 | 0.640 |

With a sample size of 78 patients, if the true treatment effect of odronextamab is 30%, 40%, 45%, or 51%, the probability is 89%, 80%, 79%, or 77%, respectively, for observed lower bound of 95% CI to exclude 15%, 25%, 30%, or 36%, respectively.

### 2.2.4. Marginal Zone Lymphoma Cohort

An exact binomial design will be adopted for the primary endpoint ORR. The 2-sided 95% confidence intervals for the observed ORRs were calculated based on a sample size of 78. Assuming a clinical meaningful ORR is greater than 20%, with 78 patients, an ORR of at least 31% will have a lower bound of the confidence interval that excludes 20%. In addition, if the observed ORR is at least 41%, 47% and 53%, the lower limit of 95% CI will exclude the ORR of 30%, 36% and 40% respectively; i.e., the ORR rate is significantly different from 30%, 36% and 40% (Table 2).

With a sample size of 78 patients, if the true treatment effect of odronextamab is 35%, 45%, 51%, or 55%, the probability is 82%, 79%, 77%, or 71%, respectively, for observed lower bound of 95% CI to exclude 20%, 30%, 36%, or 40%, respectively.

### 2.2.5. Other B-Cell non-Hodgkin Lymphoma Cohort

An exact binomial design will be adopted for the primary endpoint of ORR. The 2-sided 95% confidence intervals for the observed ORRs were calculated based on a sample size of 67. Assuming a clinical meaningful ORR is greater than 10%, with 67 patients, an ORR of at least 19% will have a lower bound of the confidence interval that excludes 10%. In addition, if the observed ORR is at least 31% and 43%, the lower limit of 95% CI will exclude the ORR of 20% and 31% respectively; i.e., the ORR is significantly different from 20% and 31% (Table 3).

**Table 3: The 2-sided 95% Exact Confidence Intervals for Observed ORR Given a Sample Size of 67 Patients**

| Number of Responders | Observed ORR | 95%CI − lower | 95% CI − upper |
|---|---|---|---|
| 13 | 0.19 | 0.108 | 0.309 |
| 21 | 0.31 | 0.206 | 0.438 |
| 29 | 0.43 | 0.312 | 0.560 |

With a sample size of 67 patients, if the true treatment effect of odronextamab is 20%, 30%, or 41%, the probability is 88%, 77%, or 71% respectively, for observed lower bound of 95% CI to exclude 10%, 20%, or 31%, respectively.

Table 4 summarizes the sample size at the original and modified step-up regimens in each cohort.

**Table 4: Sample Size at Original and Modified Step-up Regimens in Each Cohort**

| | Current enrollment at original step-up regimen as of Protocol Amendment 4 | Additional enrollment planned at modified step-up regimen | Maximum cohort size |
|---|---|---|---|
| FL grade 1-3a | 68 | 60 | 128 |
| DLBCL | 101 (67 in Arm 1, 34 in Arm 2) | 45-60 | 161 (127 in Arm 1, 34 in Arm 2) |
| MCL* | 14 | 64 | 78 |
| MZL* | 19 | 60 | 78 |
| Other B-NHL | 14 | 53 | 67 |

* MCL and MZL cohorts will remain on enrollment pause until the proposed step-up dosing regimen has been tested in other disease-specific cohorts.

In addition, if the modified step-up regimen is not deemed acceptable in a specific cohort (i.e., FL grade 1-3a, DLBCL/Other B-NHL, MZL) per interim assessment on grade ≥3 CRS, a revised regimen will be selected for testing in a new sample of 60 patients in that cohort.

# 3. ANALYSIS POPULATIONS

In accordance with guidance from the International Conference of Harmonization of Technical Requirements for Registration of Pharmaceuticals for Human Use (ICH) guideline ICH E9 Statistical Principles for Clinical Trials (ICH, 1998), the following population of analysis will be used for all statistical analysis:

## 3.1. Full Analysis Set (FAS)

Full analysis set (FAS) includes all enrolled patients who receive any doses of odronextamab. Efficacy and baseline variables will be analyzed using the FAS.

## 3.2. Safety Analysis Set (SAF)

The safety analysis set (SAF) includes all patients who received any dose of odronextamab. Treatment compliance/administration and all clinical safety variables will be analyzed using the SAF.

## 3.3. Pharmacokinetic Analysis Set

The PK analysis set includes all patients who received at least one dose of odronextamab and had at least one non-missing drug concentration result following the first dose of odronextamab.

## 3.4. Immunogenicity Analysis Set

The anti-drug antibody (ADA) analysis set includes all patients who received at least one dose of odronextamab and had at least one non-missing ADA result following the first dose of study drug. Subjects will be analyzed according to the treatment actually received.

The neutralizing antibody (NAb) analysis set includes all subjects who received at least one dose of odronextamab,  have at least one non-missing ADA result following the first dose of study drug, and either tested negative in the ADA assay at all ADA sampling times or tested positive for ADA with at least one non-missing Nab result after the first dose of the study drug. Subjects who are ADA negative are set to negative in the NAb analysis set. Subjects will be analyzed according to the treatment actually received.

# 4. ANALYSIS VARIABLES

## 4.1. Demographic and Baseline Characteristics

Patient demographics information such as age, sex, ethnicity, race, height, weight, and baseline Eastern Cooperative Oncology Group (ECOG) performance status will be collected and summarized by disease-specific cohort, dose and overall total.

Baseline disease characteristics will be summarized by disease-specific cohort, dose, and overall total for histological diagnosis at study entry. Additional disease-specific baseline disease characterisctic for each cohort include:

- Follicular lymphoma grade 1-3a: follicular lymphoma grade per ICD-O classification, and follicular lymphoma international prognostic index 1 (FLIPI1)
- Diffuse large B-cell lymphoma: determination and type of cell of origin, DLBCL subtype, DLBCL triple- or double-hit, stage, and international prognostic index (IPI) for NHL
- Mantle cell lymphoma: mantle cell lymphoma (MIPI) score and stage
- Marginal zone lymphoma: marginal zone lymphoma type, stage, and international prognostic index for NHL
- Other B-NHL: other B-NHL type, stage, and international prognostic index (IPI) for NHL
- Number of prior line of anti-lymphoma therapies
- Prior HSCT (Haematopoietic stem cell transplantation)
    - Autologous stem cell transplantation
    - Related donor allogeneic stem cell transplantation
    - Unrelated donor allogeneic stem cell transplantation
    - Cord blood transplantation

## 4.2. Medical History

Pre-existing medical conditions and adverse events that occur prior to first treatment of odronextamab will be reported as medical history and will be coded to Medical Dictionary for Regulatory Activities (MedDRA®).

## 4.3. Pre-Treatment / Concomitant Medication

Medications/Procedures will be recorded from the day of informed consent until 90 days following the last dose of odronextamab or start of a non-protocol anti-lymphoma therapy, whichever occurs first. After 90 days following the last dose or after the start of non-protocol anti-lymphoma therapy, whichever occurs first, any treatments that are started to treat SAEs deemed related to study drug should also be recorded. Medications will be coded using WHO



VV-RIM-00187995-1.0 Approved - 01 Mar 2022 GMT-5:00

(World Health Organization) Drug Dictionary (WHODD). Patients will be counted once within each ATC.

Pre-treatment medications/procedures are medications taken or procedures performed prior to administration of the study drug.

Concomitant medications/procedures are those other than odronextamab or study procedure with start date on and after the first dose of odronextamab until 90 days following the last dose of odronextamab or start of a non-protocol anti-lymphoma therapy, whichever occurs first.

## 4.4. Prohibited Medication During Study

The details of prohibited medication during study are specified in the protocol.

## 4.5. Efficacy Variable

### 4.5.1. Primary Efficacy Variable (s)

The primary endpoint of this study for each of the 5 disease-specific cohorts is ORR according to the Lugano Classification of response in malignant lymphoma (Cheson, 2014) as assessed by the Independent Central Reviewers (IRC). The final analysis for primary efficacy endpoint for ORR is assessed when patients have completed 52 weeks tumor assessments in FL/MZL or have withdrawn from the study earlier, 36 weeks tumor assessments in DLBCL/MCL/Other B-NHL or have withdrawn from the study earlier.

ORR per IRC is defined as the number of patients who achieve a best response of partial response (PR) or better based on independent central review per Lugano Classification of response in malignant lymphoma (Cheson, 2014) divided by the number of patients for each disease-specific cohort who received odronextamab. Best Overall Response (BOR) is defined as the best response, as determined by the independent central review, recorded between the date of first treatment date of odronextamab and the date of objectively documented progression per Lugano Classification. For subjects without documented progression, all available response designations will contribute to the BOR determination. For MZL, determination of response using CT (Computer tomography) only and combined PET-CT (Positron emission tomography) will be done, as mentioned in the protocol

### 4.5.2. Secondary Efficacy Variable(s)

The secondary efficacy variables include: ORR per local investigator evaluation, CR rate, PFS, OS, DOR, DOCR, DCR, TTR, TTNALT per local investigator and independent central review.

#### 4.5.2.1. Objective Response Rate per local investigator

ORR per local investigator is one of the key secondary endpoints. The definition of ORR and BOR are specified in Section 4.5.1 .

#### 4.5.2.2. Complete Response Rate

Complete response rate per investigator or IRC is defined as the number of patients who achieve a best response of CR based on local investigator or IRC review, respectively, per Lugano Classification divided by the number of all dosed patients for each disease specific cohort.



VV-RIM-00187995-1.0 Approved - 01 Mar 2022 GMT-5:00

#### 4.5.2.3. Progression-Free Survival

Progression-free survival (PFS) per IRC or investigator is defined as the time from the start of study treatment until the first documented disease progression based on IRC or investigator assessment respectively, per Lugano response criteria, or death due to any cause, whichever occurs first. If a patient has not progressed or died by the analysis cutoff date, PFS will be censored at the time of the last adequate tumor assessment on or before the cutoff date.

The following sensitivity analyses of PFS per IRC or investigator will be performed.

A sensitivity analysis of PFS is defined as the time from the start of study treatment until the first documented disease progression per Lugano response criteria, or clinical disease progression, or death due to any cause, or start date of subsequent anti-lymphoma therapy (only if 5 or more responders receive other anti-lymphoma therapy prior to documented disease progression) whichever occurs first.

#### 4.5.2.4. Overall Survival

Overall survival (OS) is defined as the time from the start of study treatment to the date of death from any cause. For subjects who are alive by the analysis cutoff date, their survival time will be censored on the date of last known alive date.

Survival follow-up will be conducted every 12 weeks post subject's end of follow-up period until the time of death, loss to follow-up, patient withdrawal of consent for follow-up, or study termination by the sponsor, whichever occurs first.

A sensitivity analysis of OS will be performed to account for the effect of the subsequent anti-lymphoma therapy. Patients who received subsequent anti-lymphoma therapy prior to death will be censored on the start date of subsequent anti-lymphoma therapy.

#### 4.5.2.5. Duration of Response

DOR will be evaluated for responders (CR or PR) only. Duration of Response (DOR) per IRC or investigator is defined as the time between the date of first documented response (CR or PR) to the date of the first documented disease progression as determined by IRC or investigator, respectively, per Lugano response criteria, or death due to any cause, whichever occurs first. If a patient has not progressed or died by the analysis cutoff date, DOR will be censored at the time of the last adequate tumor assessment on or before the cutoff date.

A sensitivity analysis of DOR is defined between the date of first documented response (CR or PR) to the date of the first documented disease progression as determined by IRC or investigator, respectively, per Lugano response criteria, or clinical disease progression, or death due to any cause, or start date of subsequent anti-lymphoma therapy (only if 5 or more responders receive other anti-lymphoma therapy prior to documented disease progression) whichever occurs first.

#### 4.5.2.6. Duration of Complete Response

DOCR will be evaluated for responders CR only. Duration of Complete Response (DOCR) per IRC or investigator is defined as the time between the date of first documented complete response (CR) to the date of the first documented disease progression as determined by IRC or investigator, respectively, per Lugano response criteria, or death due to any cause, whichever

occurs first. If a patient has not progressed or died by the analysis cutoff date, DOCR will be censored at the time of the last adequate tumor assessment on or before the cutoff date.

A sensitivity analysis of DOCR is defined between the date of first documented complete response (CR) to the date of the first documented disease progression as determined by IRC or investigator, respectively, per Lugano response criteria, or clinical disease progression, or death due to any cause, or start date of subsequent anti-lymphoma therapy (only if 3 or more subjects with CR receive other anti-lymphoma therapy prior to documented disease progression) whichever occurs first.

#### 4.5.2.7. Duration of Partial Response

DOPR will be evaluated for responders whose best overall response was PR only. Duration of Partial Response (DOPR) per IRC or investigator is defined as the time between the date of first documented partial response (PR) to the date of the first documented disease progression as determined by IRC or investigator, respectively, per Lugano response criteria, or death due to any cause, whichever occurs first. If a patient has not progressed or died by the analysis cutoff date, DOPR will be censored at the time of the last adequate tumor assessment on or before the cutoff date.

#### 4.5.2.8. Disease Control Rate

Disease control rate (DCR) per investigator or IRC is defined as the proportion of patients who achieve a best overall response CR, PR, or stable disease (SD). Best overall response of SD must have met the response SD criteria at least once ≥12 weeks after start of study treatment.

#### 4.5.2.9. Time to Response

Time to Response (TTR) per IRC or investigator is defined as the time between the date of first dose to the date of first documented response (CR or PR) as determined by IRC or investigator, respectively, per Lugano response criteria. If a patient has no CR/PR or no progression/death by the analysis cutoff date, TTR will be censored at the time of the last adequate tumor assessment on or before the cutoff date. If a patient has no CR/PR but progressed or died by the analysis cutoff date, TTR will be censored at the longest observed follow-up time (of tumor assessment per Lugano criteria) for any subject on the study. TTR will be evaluated for all patients in FAS.

#### 4.5.2.10. Time to new anti-lymphoma therapy

Time to new anti-lymphoma therapy (TTNALT) will be evaluated for patients who receive the new anti-lymphoma therapy only. Time to new anti-lymphoma therapy is defined as the time between the date of first dose to the date of new anti-lymphoma therapy.

## 4.6. Safety Variables

### 4.6.1. Adverse Events and Serious Adverse Events

Adverse events and serious adverse events will be collected from the time of informed consent signature and then at each visit up to 90 days following the last dose, or until the start of non-protocol anti-lymphoma therapy, whichever is earlier. In addition, any SAE that the investigator

believes may be related to study treatment and that occurs after 90 days following the last dose or after the start of non-protocol anti-lymphoma therapy, whichever is earlier, must be collected.

For safety variables, three observation periods are defined for analysis.

The **pre-treatment period** is defined as the time between the day of informed consent and the time prior to the first dose of study treatment.

The **on-treatment period** is defined as the time from the first dose of study treatment up to 90 days after the last dose day of treatment, or the day prior to the start of non-protocol anti-lymphoma therapy, whichever comes first.

The **post-treatment period** is defined as the time after the on-treatment period.

**Treatment Emergent Adverse Events (TEAEs)** are defined as those AEs that occurred during the on-treatment period and any treatment-related SAEs that occurred during the post-treatment period.

An acute infusion related reaction (IRR) is defined as any AE that occurs less than 6 hours from the start of the infusion, or within 2 hours after completion of the infusion (whichever is later), and is associated with typical signs and symptoms including, but not limited to, flushing, tachycardia, hypotension, dyspnea, bronchospasm, back pain, fever, urticaria, edema, nausea, and rashes. Infusion-related reactions (IRRs) will be reported as AEs and graded according to the NCI-CTCAE version 5.

Cytokine release syndrome is a disorder characterized by fever, tachypnea, headache, tachycardia, hypotension, rash, and/or hypoxia; in this study it is defined as such an event that occurs 6 or more hours from the start of the infusion or more than 2 hours after completion of the infusion (whichever is later). Cytokine release syndrome will be reported as AEs and graded according to the criteria adapted from (Lee, 2019).

Laboratory results, vital signs, or ECG abnormalities are to be recorded as AEs if they meet any of the following criteria:

- the test result is associated with accompanying symptoms, and/or
- the test result requires additional diagnostic testing or medical/surgical intervention,
- and/or
- the test result leads to a change in dosing (outside of protocol-stipulated dose
- adjustments), discontinuation from the study, significant additional concomitant drug treatment, or other therapy

### 4.6.2. Adverse Events of Special Interest

An AESI (serious or non-serious) is one of scientific and medical concern specific to the sponsor’s product or program, for which ongoing monitoring and rapid communication by the investigator to the sponsor can be appropriate.

#### 4.6.3. Laboratory Safety Variables

The clinical laboratory data consists of blood chemistry, hematology, urinalysis and other labs. Clinical laboratory values will be converted to standard international (SI) units and grouped by function in summary tables. Conventional unit may be provided.

#### 4.6.4. Vital Signs

Vital signs including temperature, blood pressure, pulse, pulse oximetry, and respiration rate will be collected at screening, treatment period and safety follow-up periods.

#### 4.6.5. 12-Lead Electrocardiography (ECG)

12-Lead ECG parameters include heart rate (recorded from the ventricular rate), the PR, QRS, QT and QTcF, intervals.

#### 4.6.6. Physical Examination Variables

Complete physical examination including examination of skin, head, eyes, nose, throat, neck, joints, lungs, heart, pulse, abdomen (including liver and spleen), lymph nodes, and extremities, as well as a brief neurologic examination at screening.

Limited physical examination including lungs, heart, abdomen, skin, and a lymphatic examination may be performed at subsequent visits during treatment and safety follow-up periods.

## 4.7. Pharmacokinetic Variables

The PK variables are odronextamab concentrations in serum and time.

## 4.8. Immunogenicity Variables

The immunogenicity variables are ADA status, titer, NAb status, and time-point/visit. Samples positive in the ADA assay will be further characterized for ADA titers and for the presence of NAb against odronextamab.

## 4.9. Biomarker Variables

Biomarker variables include serum cytokine biomarker levels over time. Additional variables include absolute counts of T cells and B cells in blood over time, along with the percentage of proliferating / activated T cells of all T cells. The time points for cytokine and immunophenotyping sampling are described in protocol R1979-ONC-1625.

## 4.10. Patient-Reported Outcomes Variables

Patient reported outcomes regarding symptoms, functioning and health related quality of life (QoL) will be evaluated using validated instruments including the EORTC QLQ-C30, EQ-5D-3L, and FACT-Lym. These instruments will be administered during visits at screening and prior to study drug administration at weeks 1, 2, 3, 4 and 10, followed by Q8W in the first year, Q12W in the second year, Q24W in the third and fourth years.



VV-RIM-00187995-1.0 Approved - 01 Mar 2022 GMT-5:00

### 4.10.1. EORTC QLQ-C30

Health-related quality of life (HRQoL) will be assessed using the European Organization for Research and Treatment of Cancer (EORTC) QLQ-C30 questionnaire Version 3. This is a 30-item instrument that has gained wide acceptance in oncology clinical studies. The EORTC QLQ-C30 is composed of multi-item scales and single item measures. These include a global health status/quality of life and five functional scales (physical, role, emotional, social, and cognitive), three symptom scales (fatigue, nausea/vomiting, and pain) and six single item measures assessing additional symptoms commonly reported by cancer patients (dyspnea, insomnia, appetite loss, constipation, and diarrhea) and perceived financial impact of disease. Except for the global health status/ quality of life scale, responses for all items use a 4 point Likert scale ranging from 1 (Not at all) to 4 (Very much). Responses for individual items of the global health status/quality of life scale use a 7-point Likert scale. Scores for all scales and single item measures are standardized to a 0 to 100 range using linear transformation and missing data is addressed based on recommendation in the EORTC QLQ-C30 scoring manual. For the global health status / QOL and functioning scales, higher scores indicate better functioning and health; for the symptom scales/items, higher scores indicate higher symptom burden.

### 4.10.2. FACT-Lym

The FACT-Lym is composed of 27-item from the Functional Assessment of Cancer Therapy-General (FACT-G) plus the 15-item lymphoma subscale (LymS). The FACT-G is the generic core of the Functional Assessment of Chronic Illness Therapy Measurement System (FACIT) and can be used with patient of any tumor type. A 5-point Likert scale ranging from 0 (Not at all) to 4 (Very much) is used in FACT-Lym. The instrument measures the respondent’s health state over the past 7 days in four domains, including physical well-being (PWB, 7 items), social/family well-being (SWB, 7 items), emotional well-being (EWB, 6 items), and functional well-being (FWB, 7 items), and additional concerns (15 items).

### 4.10.3. EQ-5D-3L

The EQ-5D instrument’s descriptive system comprises 5 dimensions: mobility, self-care, usual activities, pain/discomfort and anxiety/depression. Each dimension has 3 levels: no health problems, moderate health problems, and extreme health problems. A dimension for which there are no problems is said to be at level 1, while a dimension for which there are extreme problems is said to be at level 3. Thus, the vectors 11111 and 33333 represent the best health state and the worst health state, respectively, as described by the EQ-5D-3L. These numerals do not have arithmetic properties and cannot be used as a cardinal scale. Nevertheless, empirically derived weights can be applied to an individual’s response to the EQ-5D-3L descriptive system to generate a single summary index score (Euroqol, 2018).

In addition, the EQ-5D-3L includes a visual analog scale (VAS) score. The VAS score represents the respondent’s self-rated health on a vertical, visual analogue scale with two endpoints labeled as 0=’worst imaginable’ and 100=’best imaginable’ health state.

# 5. STATISTICAL METHODS

For continuous variables, descriptive statistics will include the following: the number of patients reflected in the calculation (n), mean, median, standard deviation, minimum, and maximum.

For categorical or ordinal variables; frequencies and percentages will be displayed for each category.

For time-to-event data; Kaplan-Meier curves and estimates, median survival, survival rate at the key landmark time point along with 95% confidence interval will be provided, if applicable.

In general, the analysis will be performed by disease-specific cohort, dose/regimen, and overall total. The details are provided as follow:

- All patients by disease-specific cohort, full dose
- Patients with 1/20 original step-up dosing regimen by disease-specific cohort, full dose
- Patients with 0.7/4/20 revised step-up dosing regimen disease-specific cohort, full dose

## 5.1. Demographics and Baseline Characteristics

Demographic and baseline disease characteristics will be summarized by disease-specific cohort, dose and overall total and listed for the following.

- Age (Summary statistics; <65, ≥65)
- Sex (Male vs. Female)
- Race (White, Black, or African American, Asian, American Indian or Alaska Native, Native Hawaiian or Other Pacific Islander, Not reported, Unknown, Other)
- Ethnicity (Hispanic/Latino, not Hispanic/Latino)
- Baseline ECOG performance status
- Histological diagnosis at study entry (Follicular lymphoma, Diffuse large B-cell lymphoma, Marginal zone lymphoma, Mantle cell lymphoma, Other B-cell Hodgkin Lymphomas)
- Ann Arbor stage at study entry (I, II, III, IV)
- Presence of bulky disease (Yes, No)

Other disease-specific baseline characteristics at study entry will be summarized:

- Follicular lymphoma grade 1-3a
  - Follicular lymphoma grade per ICD-O classification
  - Risk factors for Follicular lymphoma international prognostic index (FLIPI-1) and individual risk factor per investigator
  - POD24 (Progression of disease within 2 years) (Yes, No)

- Diffuse large B-cell lymphoma
  - Cell of origin (Germinal Center B-Cell-like DLBCL, Activated B-Cell-like DLBCL/Non-GCB, Unclassified DLBCL)
  - DLBCL subtype (De novo, Transformed [Richter], Transformed [non-Richter])
  - DLBCL triple- or double-hit
  - International prognostic index score (IPI)
- Mantel cell lymphoma
  - Mantle cell lymphoma (MIPI) score
- Marginal zone lymphoma
  - Marginal zone lymphoma type (Extranodal marginal zone B-cell lymphoma [MALT], Nodal marginal zone B-cell lymphoma, splenic marginal zone lymphoma, Gastric MALT lymphoma, Nongastric MALT lymphoma, unknown)
  - International prognostic index score (IPI)
- Other B-NHL
  - Subtype (Follicular lymphoma grade 3b, Burkitt's lymphoma, B-cell lymphblastic lymphoma, PMBCL, Grey zone lymphoma, High grade B-cell lymphoma NOS, Other)

## 5.2. Medical History

Frequency and incidence of medical history and pre-treatment AE will be summarized by SOC and preferred terms.

## 5.3. Prior/concomitant Illnesses and Medications/Procedures

### 5.3.1. Prior Lymphoma-related cancer systemic therapies

Prior lymphoma-related therapy will be summarized for

- Prior systemic therapy
  - Number of prior lines of systemic therapy
  - Relapse/refractory status to prior therapy
  - Prior exposure to anti-CD20 antibody
  - Prior exposure to alkylating agent
- Prior exposure to BTK inhibitor
- Prior exposure to PI3K (Phosphatidylinositol 3-kinase) inhibitor
- Primary refractory disease to first line treatment
- Refractory to last line of therapy

- Refractory to anti-CD20 in last line therapy
- Refractory to anti-CD20 in any line of therapy
- Double refractory (to both anti-CD20 and alkylator)
- Prior exposure to $R^2$ (Rituximab and Lenalidomide)

Refractory is defined as disease that progressed during previous therapy or relapsed within 6 months from completion of the last dose of any line of therapy. Relapse is defined as disease that recurs following a response lasting > 6 months from completion of the last dose of any line of therapy.

### 5.3.2. Prior Lymphoma-related cancer radiotherapy

- Site of radiation
- Intent of radiation treatment (Curative, Palliative, Prophylaxis)

### 5.3.3. Prior Lymphoma-related cancer surgery

- Surgery location

### 5.3.4. Prior Hematopoietic stem cell transplants

- Autologous stem cell transplantation
- Related donor allogeneic stem cell transplantation
- Unrelated donor allogeneic stem cell transplantation
- Cord blood transplantation

Frequency and proportion of subjects taking concomitant mediation other than antineoplastic agents or other systemic anti-lymphoma therapies will be summarized. Similar summaries will be provided for dexamethasone premedication and subsequent anti-lymphoma therapy.

## 5.4. Prohibited Medications

Frequency and proportion of subjects taking prohibited medication specified in the protocol will be summarized and listed.

## 5.5. Subject Disposition

Subject disposition will be summarized based on FAS. Summary of subject enrollment by region, country and site will be provided. Number and percentage of patients who are treated, completed or discontinued treatment, discontinuation of study as well the reason for discontinuation will be summarized.

## 5.6. Extent of Study Treatment Exposure and Compliance

Summary of exposure will be based on all treated subjects (SAF) by treatment, unless otherwise specified. Treatment duration, dose intensity, and number of doses administered will be summarized.

### 5.6.1. Measurement of Compliance

Treatment compliance will be assessed using number of doses taken compared with number of doses that should have been taken. Specifically, compliance with investigational product will be calculated as follows:

$$\frac{\text{Number of investigational product taken during treatment period}}{\text{Number of investigational product prescribed to be taken during treatment period}} \text{x100\%}$$

where temporary dose discontinuation is ignored.

The number and percentage of subjects who have <60%, 60-80%, 80-100%, and >100% compliance will be summarized.

### 5.6.2. Exposure to Investigational Product

Exposure to investigational product will be summarized for the following:

- Number of study doses administered
- Cumulative dose received (the sum of administered dose)
- Duration of treatment (the interval between date of first dose and date of last dose plus 14 days)
- Actual dose intensity (the ratio of cumulative dose received and treatment duration)
- Relative dose intensity (RDI) (the ratio of actual dose intensity/planned dose intensity)

There are two types of RDI. They will be calculated for before full dose period, after full dose period and all periods, respectively.

Relative dose intensity 1=Actual dose intensity/Planned dose intensity 1 for the step-up dosing period before reaching the full dose, after full dose period, all periods.

Relative dose intensity 2=Actual dose intensity/Planned dose intensity 2 for before full dose period, after full dose period, all periods.

Planned dose intensity 1 uses the original planned dose (e.g., 4 mg for step up regimen at Week 2 regardless whether the patient could tolerate the Week 1 dose and need to retake it at Week 2)

Planned dose intensity 2 uses the modified planned dose (e.g., 0.7 mg for step up regimen at Week 2 since patient could not tolerate 0.7 mg at Week 1 and need to retake 0.7 mg.)

### 5.6.3. Modifications of Study Treatment

#### 5.6.3.1. Dose Delays

Dose delays will be summarized for the following:

- Number and percentage of subjects with at least one dose delay
- Number of dose delays per subject
- Duration of dose delays in days
    - Patients who are taking drug in QW schedule,

      Duration of dose delays = Date of current dose – Date of previous dose - 7 -2


VV-RIM-00187995-1.0 Approved - 01 Mar 2022 GMT-5:00

- Patients who are taking drug in Q2W schedule,

  Duration of dose delays = Date of current dose – Date of previous dose - 14 -2

- Number of Patients who were impacted by COVID-19 and had dose delay will be summarized.

#### 5.6.3.2. Infusion Interruptions

Infusion interruptions will be summarized for the following:

- Number and percentage of subjects with at least one infusion interruptions
- Number of infusion interruptions per subject and the reason for infusion interruption

#### 5.6.3.3. Dose Modifications

Dose modifications will be summarized for the following:

- Number and percentage of subjects with at least one dose modifications
- Number of dose modifications per subject and the reason for dose modification

## 5.7. Analyses of Efficacy Variables

No formal statistical testing is planned for this study. All analyses are descriptive in nature and will be summarized by disease-specific cohort. Confidence intervals will be reported at the two-sided 95% level.

In efficacy analysis of ongoing data, the patients who had opportunity for 12 weeks efficacy assessment will be included.

### 5.7.1. Analysis of Primary Efficacy Variable(s)

Objective response rate (ORR) according to Lugano Classification per IRC is the primary efficacy endpoint. The number and percentage of subjects in each category of BOR (completed response [CR], partial response [PR], stable disease [SD], progressive disease [PD], not evaluable [NE]) per IRC will be presented. Estimates of response rate, along with its exact two-sided 95% CI by Clopper and Pearson will be presented.

The final analysis for primary efficacy endpoint will be performed after the patients have completed 52 weeks tumor assessments in FL/MZL, 36 weeks tumor assessment in DLBCL/MCL/Other B-NHL or have withdrawn from the study earlier.

Descriptive analysis for patients in the two regimen arms (160 mg QW/320 mg Q2W, 320 mg QW/320 mg Q2W) in DLBCL cohort will be performed to support PK/pharmacodynamic and advance the understanding of the exposure-response analysis.

An interim efficacy analysis for primary efficacy endpoint will be performed for FL and DLBCL cohorts. An interim futility analysis will be performed for MCL, MZL, and Other B-NHL cohorts separately. The details are described in Section 7 .

**Subgroup analysis:**

The influence of baseline and demographic characteristics on the treatment effect may be explored. ORR per IRC may be summarized within subsets defined by the following baseline factors recorded on the CRF:

*Subgroups common for all histological subtypes:*

- Age (<65 years, ≥65 years),
- Geographic region (Europe, North America, Asia, Rest of World)
- ECOG performance status (0, 1, 2)
- Ann Arbor Stage (I, II, III, IV)
- Bulky Disease (Yes, No)
- Patients received at least one full dose (Yes, No)
- COVID-19 (AE related with COVID Y/N, Patient impact by COVID Y/N)

*Subgroups specific for histological subtypes:*

**DLBCL**

- Cell of origin (Germinal Center B-Cell-like DLBCL, Activated B-Cell-like DLBCL/Non-GCB, Unclassified DLBCL
- DLBCL subtype (De novo, Transformed [Richter], Transformed [non-Richter])
- DLBCL (Triple-hit, double-hit)
- International prognostic index score (IPI) for DLBCL (low, intermediate, high)

**FL grade 1-3a**

- Risk factors for Follicular lymphoma international prognostic index (FLIPI) and individual risk factor per investigator (low, intermediate, high)
- POD24 for FL patients (Yes, No)

**MCL**

- Mantle cell lymphoma (MIPI) score (low, intermediate, high)

**MZL**

- Marginal zone lymphoma type (Extranodal marginal zone B-cell lymphoma [MALT], Nodal marginal zone B-cell lymphoma, splenic marginal zone lymphoma, Gastric MALT lymphoma, Nongastric MALT lymphoma, unknown)
- International prognostic index score (IPI) for MZL (low, intermediate, high)

**Other B-NHL**

- Other B-NHL subtype (Follicular Lymphoma grade 3b, Burkitt's lymphoma, B-cell lymphoblastic lymphoma, PMBCL, Grey zone lymphoma, High grade B-cell lymphoma NOS, Other)



VV-RIM-00187995-1.0 Approved - 01 Mar 2022 GMT-5:00

- Other B-NHL disease stage (I, II, III, IV)

In case a subgroup includes less than 10 subjects, the analysis for the given subgroup will not be performed or may be combined with other subgroup.

### 5.7.2. Analysis of Secondary Efficacy Variables

The secondary efficacy variables include: ORR per investigator evaluation, CR rate, PFS, OS, DOR, DOCR, DCR, TTR, TTNALT, and per investigator and independent central review will be summarized.

#### 5.7.2.1. Analysis of ORR per Investigator

Objective response rate (ORR) according to Lugano Classification per investigator will be analyzed in a similar fashion as the primary analysis for ORR per IRC.

#### 5.7.2.2. Analysis of Complete Response Rate

Complete response (CR) rate per IRC or per investigator will be analyzed using similar statistical methods as described above for ORR analysis per IRC.

#### 5.7.2.3. Analysis of Progression-Free Survival

Progression-free survival (PFS) will be estimated using the Kaplan Meier (KM) product limit method and will be presented graphically. A two-sided 95% CI for median PFS will be calculated. PFS rates at fixed time points (6 months, 12 months, 18 months, etc.) will be presented along with their corresponding 95% CIs.

Analyses of PFS will be performed per investigator and per IRC, respectively.

#### 5.7.2.4. Analysis of Overall Survival

Overall survival (OS) will be estimated using the KM product limit method and will be presented graphically. A two-sided 95% CI for median OS will be calculated. Survival rates at fixed time points (6 months, 12 months,18 months, etc.) will be presented along with their corresponding 95% CIs.

#### 5.7.2.5. Analysis of Duration of Response

Duration of response (DOR) will be evaluated for subjects who achieve best response of PR or better. The DOR per IRC will be estimated using KM product limit method and will be displayed graphically. A table presenting number of responders, number of events, median DOR and the corresponding 95% CI for the median will be provided. In case there are less than 10 responders in a cohort, the DOR analysis may be provided using descriptive analysis only.

Analysis of DOR per investigator will be performed in a similar method as described above.

#### 5.7.2.6. Analysis of Duration of Complete Response

Duration of complete response (DOCR) will be evaluated for subjects who achieve best response of CR. The DOCR per IRC will be estimated using KM product limit method and will be displayed graphically. A table presenting number of CRs, number of events, median DOCR and

the corresponding 95% CI for the median will be provided. In case there are less than 10 CRs in a cohort, the DOCR analysis may be provided using descriptive analysis only.

Analysis of DOCR per investigator will be performed in a similar method as described above.

#### 5.7.2.7. Analysis of Duration of Partial Response

Duration of partial response (DOPR) will be evaluated for subjects who achieve best response of PR. The DOPR per IRC will be estimated using KM product limit method and will be displayed graphically. A table presenting number of PRs, number of events, median DOPR and the corresponding 95% CI for the median will be provided. In case there are less than 10 PRs in a cohort, the DOPR analysis may be provided using descriptive analysis only.

Analysis of DOPR per investigator will be performed in a similar method as described above.

#### 5.7.2.8. Analysis of Disease Control Rate

Disease control rate (DCR) per IRC will be analyzed using similar statistical methods as described in the above for ORR analysis per IRC. The same analyses will be repeated for DCR per investigator.

#### 5.7.2.9. Analysis of Time to Response

Time to response (TTR) will be evaluated for all patients in FAS. The TTR per IRC will be estimated using KM product limit method and will be displayed graphically. A table presenting number of events, median TTR the corresponding 95% CI for the median will be provided.

Analysis of TTR per investigator will be performed in a similar method as described above.

#### 5.7.2.10. Analysis of Time to Anti-Lymphoma Therapy

Time to anti-lymphoma therapy (TTALT) will be summarized by descriptive statistics for patients who had anti-lymphoma therapy in FAS.

### 5.7.3. Adjustment for Multiple Comparison

Since no formal statistical testing is planned in this study, adjustment for multiplicity is not relevant.

### 5.7.4. Analysis of Patient-Reported Outcomes Data

PRO instruments will be scored according to their respective validation papers, scoring algorithms and user's guides.

PRO analysis will be based on the full analysis set (FAS) as defined in Section 3.1.

#### 5.7.4.1. Patient Disposition

For all PRO scheduled assessment visits, patient disposition will be summarized.



VV-RIM-00187995-1.0 Approved - 01 Mar 2022 GMT-5:00

#### 5.7.4.2. Instrument Completion Rates

For all PRO scheduled assessment visits, the number and percentage of patients who complete at least the minimum requirements for scoring of the instrument will be provided for EORTC QLQ-C30, FACT-Lym and EQ-5D-3L.

#### 5.7.4.3. Descriptive Summary Statistics by Visit

Summary statistics of absolute scores and change from baseline by visit will be produced for each PRO instrument scale. The summary tables will include mean (SD), median, range (min, max) and 95% confidence interval. Data from scheduled visits, including safety and efficacy follow-up visits, will be included in the summary tables. Data from unscheduled visits will not be included-in the summary analysis.

Change from baseline in FACT-LYM item scores (raw scores) will be classified according to the following item response categories:

- Improved ≥ 3 points compared to baseline
- Improved 2 points compared to baseline
- Improved 1 point compared to baseline
- Stable
- Worsened 1 point compared to baseline
- Worsened 2 points compared to baseline
- Worsened ≥ 3 points compared to baseline

Line charts depicting the mean and mean change from baseline along with 95% confidence interval over time will be provided for each scale/subscale.

#### 5.7.4.4. Longitudinal Analysis of Change from Baseline

Change from baseline in EORTC QLQ-C30 (global health status/QoL, physical function and role function, and fatigue) will be analyzed using Mixed-Effects Model Repeated Measure (MMRM) model. Data from scheduled visits while patients are still on study treatment will be included in the analyses. Data after patients off treatment or from unscheduled visits will not be included. LS means of change from baseline at each visit as well as overall average across visits with the 95% confidence intervals will be provided.

#### 5.7.4.5. Time to definitive deterioration (TTDD)

An individual-level analysis will be performed to examine clinically meaningful changes, which is based on the minimally clinically important difference (MCID), the smallest score change that reflects a clinically meaningful treatment effect. The MCID is defined a priori as a ±10-point change from baseline, according to findings of an anchor-based analysis of MCID for EORTC QLQ-C30 included into the EORTC QLQ-C30 Scoring Manual.

**Table 5: Minimal Clinically Important Difference (MCID)**

| Score | Change from baseline | Visit response |
|---|---|---|
| **EORTC QLQ-C30** | | |
| • Fatigue<br>• Functional scales (i.e., physical, role)<br>• Global health status/QoL | ≥ +10 | Worsened |
| | ≤ -10 | Improved |
| | Otherwise | No change |

Change from baseline will be categorized as "improvement" or "deterioration," defined as a 10-point increase or decrease from baseline, respectively, or "no change," defined as a < 10-point increase or decrease from baseline. For each scale, proportions of patients categorized as having improvement, no change, or deterioration will be calculated using the total number of patients who provide answers for ≥50% of the items on the scale.

TTDD will be defined as the time from the date of 1st dose to the date of the first deterioration in PRO scores of at least 10 points as compared to the baseline score, if the deterioration of at least 10 points as compared to the baseline score is also observed at all subsequent time points, or if the patient do not have any subsequent PRO assessments.

For those patients who experienced a definitive meaningful deterioration, TTDD will be computed as follows and then converted to months:

TTDD=Date of definitive clinically meaningful deterioration was observed–Date of 1st dose + 1

Patients who have not experienced a definitive meaningful deterioration and with a non-missing baseline assessment will be censored at the last available PRO assessment. Patients with no baseline PRO assessment, or with a non-missing baseline assessment and without post-baseline PRO assessment, or whose baseline scores do not allow for further deterioration (i.e. The baseline score less than 10) will be censored at the date of 1st dose. Death or progression will not be considered deterioration events.

TTDD of EORTC QLQ-C30 (global health status/QoL, physical and role function, and fatigue) will be analyzed by Kaplan-Meier methodology.

Additional PRO analyses (e.g. details of descriptive analyses of all PRO subscales/domains, imputation methods, handling of missing data, and sensitivity analysis) will be provided in a separated PRO analysis plan (PRO SAP).

## 5.8. Analysis of Safety Data

The analysis of safety and tolerance will be performed on the SAF.

The summary of safety results will be summarized for each disease-specific cohort, dose, and overall total.

### 5.8.1. Adverse Events

Summaries that include frequencies and proportions of patients reporting AEs will include the PTs and the SOCs.

The focus of adverse event analysis in the clinical study report will be on TEAEs. For details on handling missing data and partial dates, see Section 6.

Summaries of TEAEs for each disease cohort will include:

- Overall summary of TEAEs by SOC
- Overall summary of TEAEs by SOC/PT
- Overall summary of TEAEs SOC/PT/CTCAE NCI Grade (all and 3-5)
- Overall summary of TEAEs by PT and NCI Grade (all and 3-5)
- Overall summary of TEAEs resulting in treatment dose delayed, drug interrupted, dose reduced, infusion slowed, drug discontinuation by worst severity grade, NCI Grade (all and 3-5) SOC/PT
- Overall summary of TEAEs resulting in death SOC/PT

The same analyses will be repeated for treatment-related TEAEs, serious TEAEs, serious treatment-related TEAEs, treatment-related TEAE resulting in death, and AEs of special interest.

Number of patients who received/not received the prophylaxes medication and had infection/no infection will be summarized in a 2×2 table. The infection includes PCP (Pneumocystis carinii or Pneumocystis jirovecii pneumonia), CMV (Cytomegalovirus), and HSV (Herpes simplex virus). The corresponding prophylaxis medications contain Bactrim (trimethorprim/sulfamethoxazole); dapsone; pentamidine; atovaquone for PCP, valganciclovir; ganciclovir for CMV, and acyclovir; valacyclovir for HSV.

Summary of TEAEs from initial dosing to intermediate dosing (prior to QW full dose) will also be provided.

Other AE summaries include:

- On-treatment death with primary reason of death
- On-treatment death by COVID (AE related with COVID (Y/N), Patient impact by COVID (Y/N))
- All death with primary reason of death
- Summary of IRR/CRS by worst severity grade (All grades, Grade 3-5)
- Summary of IRR/CRS by worst severity grade (each grade)
- Summary of IRR by worst severity grade (All grades, Grade 3-5)
- Summary of IRR by worst severity grade (each grade)
- Summary of CRS by worst severity grade (All grades, Grade 3-5)
- Summary of CRS by worst severity grade (each grade)
- Summary of signs and symptoms of IRR by worst severity grade (All grades, Grade 3-5) PT
- Summary of signs and symptoms of IRR by worst severity grade (each grade) PT

- Summary of signs and symptoms of CRS by worst severity grade (All grades, Grade 3-5) PT
- Summary of signs and symptoms of CRS by worst severity grade (each grade) PT
- Summary of CRS during initial dose, intermediate dose and full QW dose (All grades, Grade 3-5)
- Summary of CRS during initial dose, intermediate dose and full QW dose (each grade)
- Summary of treatment related ICANS events per sponsor definition (All grades, Grade 3-5)
- Summary of recurrent CRS/IRR episodes

Counts will be provided in each cohort for each PT within each SOC. Percentages will be calculated using the number of patients from the safety population in each disease-specific cohort. For TEAE summary presented by SOC and PT, the summary table will be sorted by descending frequency of primary SOC, and then by descending frequency of PTs within the primary SOC. For TEAE summary presented either by SOC or PT, the summary will be sorted by decreasing frequency of SOC or PT, respectively.

In addition, summary of time to onset of CRS/IRR separately from start of infusion, and duration of CRS will be provided.

Analysis of treatment emergent adverse events of special interest to be presented based on investigator reported-check box. These summaries will be conducted by disease-specific cohort, presented by SOC and PT.

### 5.8.2. Clinical Laboratory Measurements

Baseline clinical laboratory analytes and change from baseline in selected clinical laboratory analytes to each scheduled assessment time will be summarized with descriptive statistics by disease-specific cohort. Summary statistics will include the number of patients, mean, median, standard deviation, quartiles, minimum, and maximum. The graphs of mean (or median) value of some lab parameter vs. visit may be plotted if applicable.

Summary tables for new or worsened laboratory results values by NCI CTCAE version 5 all grade and grade ≥ 3 will be generated.

Shift tables from baseline to post-treatment worst NCI CTCAE version 5 grade will be generated.

### 5.8.3. Analysis of Vital Signs

Vital signs (weight, temperature, pulse, pulse oximetry, blood pressure, and respiration rate) will be summarized by baseline and change from baseline to each scheduled assessment time with descriptive statistics. The graphs of mean (or median) value of some vital sign parameter vs. visit may be plotted if applicable.


VV-RIM-00187995-1.0 Approved - 01 Mar 2022 GMT-5:00

### 5.8.4. Analysis of 12-Lead ECG

ECG parameters will be summarized by baseline and change from baseline to each scheduled and collected assessment time.

ECG status (i.e. normal, abnormal) will be reported. Shift tables will be provided to present the post-baseline status according to the baseline status (normal or missing / abnormal) by disease specific cohort.

### 5.8.5. Physical Exams

Physical exam parameters will be summarized by disease specific cohort.

### 5.8.6. Analysis of Drug Concentration Data

Odronextamab concentrations in serum over time will be measured.

Pharmacokinetic parameters will be generated and summarized with descriptive statistics. Odronextamab PK parameters may include but are not limited to:

- Cmax: maximal concentration in a dosing interval
- Cmin: pre-dose concentration in a dosing interval

The summary of odronextamab concentrations over time will be grouped by regimen received and by disease-specific cohorts.

Data collected in this study may be used for a population PK analysis in conjunction with data from other odronextamab studies. The results will be described in a separate report.

### 5.8.7. Analysis of Immunogenicity Data

#### 5.8.7.1. Analysis of ADA Data

The immunogenicity variables will be summarized using descriptive statistics.

Immunogenicity will be characterized by the ADA response and titers observed in subjects in the ADA analysis set. ADA response categories and titer categories are defined as follows:

ADA response categories:

- ADA Negative, defined as ADA negative in the ADA assay at all time points, regardless of any missing samples.
- Pre-existing immunoreactivity, defined as either an ADA positive response at baseline with all post first dose ADA results negative, or a positive response at baseline, with all post first dose ADA assay responses less than 9-fold over baseline titer levels.



VV-RIM-00187995-1.0 Approved - 01 Mar 2022 GMT-5:00

- Treatment-emergent ADA response, defined as an ADA positive response in the ADA assay post first dose when the baseline results are negative or missing. The treatment-emergent responses will be further characterized as Persistent, Indeterminate or Transient.
  - Persistent Response – Treatment-emergent ADA positive response with two or more consecutive ADA positive sampling time points, separated by at least 16-week period (based on nominal sampling time), with no ADA negative samples in between, regardless of any missing samples.
  - Indeterminate Response – Treatment-emergent ADA positive response with only the last collected sample positive in the ADA assay, regardless of any missing samples.
  - Transient Response – Treatment-emergent ADA positive response that is not considered persistent or indeterminate, regardless of any missing samples.
- Treatment boosted ADA response, defined as any positive response in the ADA assay post first dose that is greater than or equal to 9-fold over baseline titer levels when baseline results are positive in the ADA assay.
- Maximum ADA Titer Category
  - Low (titer <1,000)
  - Moderate (1,000≤ titer ≤10,000)
  - High (titer >10,000)

The following analysis will be provided:

- Number (n) and percentage (%) of ADA-negative subjects (pre-existing immunoreactivity or negative in the ADA assay at all time points) by treatment groups
- Number (n) and percent (%) of treatment-emergent ADA positive subjects by treatment groups and ADA titer categories and at the
  - Number (n) and percent (%) of persistent treatment-emergent ADA positive subjects/patients
  - Number (n) and percent (%) of indeterminate treatment-emergent ADA positive subjects/patients
  - Number (n) and percent (%) of transient treatment-emergent ADA positive subjects/patients
- Number (n) and percent (%) of treatment-boosted ADA positive subjects by treatment groups and ADA titer categories

#### 5.8.7.2. Analysis of Neutralizing Antibody (NAb) Data

The absolute occurrence (n) and percent of subjects (%) with NAb status in the Nab analysis set will be provided by treatment groups.

### 5.8.8. Analysis of Biomarkers

The following biomarker analyses will be performed.

To determine the effect of odronextamab step-up dosing regimen on the patterns and peak levels of cytokines, descriptive statistics for IL-6 values (pre-dose and peak) will be produced at each time point for patients receiving 1/20 mg vs. 0.7/4/20 mg step-up regimen in FL and DLBCL respectively. In addition, box plots for IL-6 values (pre-dose and peak) will be generated over time for patients receiving 1/20 vs. 0.7/4/20 step-up regimens in FL and DLBCL respectively.

To determine the effects of odronextamab on T-cell counts/proliferation status and B-cell counts, descriptive statistics for the following immune cell markers will be produced over time in FL and DLBCL respectively. In addition, box plots for these immune cell markers will be generated over time in FL and DLBCL respectively.

- CD3+ ABS
- CD3+CD4+ ABS
- CD3+CD8+ ABS
- CD3-CD19+ ABS
- CD3+CD8+CD4-DR+ (%CD8)
- CD3+CD8+CD4-Ki67+ (%CD8)
- CD3+CD4+CD8-DR+ (%CD4)
- CD3+CD4+CD8-Ki67+ (%CD4)

To evaluate the association of baseline B-cell and T-cell counts with clinical CRS on odronextamab treatment, descriptive statistics for the following immune cell markers at baseline will be presented for patients receiving 1/20 mg vs. 0.7/4/20 mg step-up regimen by CRS grade in FL and DLBCL respectively. In addition, scatter plots for these immune cell markers at baseline will be generated for patients receiving 1/20 mg vs. 0.7/4/20 mg step-up regimen by CRS grade in FL and DLBCL respectively.

- CD3+ ABS
- CD3+CD4+ ABS
- CD3+CD8+ ABS
- CD3-CD19+ ABS

### 5.8.9. Pharmacokinetic/Pharmacodynamic Analyses

Pharmacokinetic/pharmacodynamic analyses may be conducted on exploratory biomarkers, as appropriate.

### 5.8.10. Exposure-Response Analysis

Exposure-Response (E-R) analyses may be conducted on efficacy endpoints, safety, and relevant exploratory biomarkers, as appropriate.


VV-RIM-00187995-1.0 Approved - 01 Mar 2022 GMT-5:00

## 5.9. Association of Immunogenicity with Exposure, Safety, and Efficacy

### 5.9.1. Immunogenicity and Exposure

Potential association between immunogenicity variables and systemic exposure will be explored by treatment groups. Plots of drug concentration time profiles may be provided to examine the potential impact of ADA response status, titer and NAb on these profiles.

### 5.9.2. Immunogenicity and Safety and Efficacy

Potential association between immunogenicity variables and safety may be explored with a primary focus on the following safety events during the TEAE period:

- Infusion reactions
- Hypersensitivity (SMQ: Hypersensitivity [Narrow])
- Anaphylaxis (SMQ: Anaphylaxis [Narrow])

Potential association between immunogenicity variables and efficacy endpoints may be explored (e.g. scatter plot or spaghetti plot).

The safety and efficacy analyses mentioned above will be conducted using the following categories:

- ADA Positive subjects:
    - Treatment-emergent
    - Treatment-boosted
- Maximum post-baseline titer category
- NAb positive

# 6. DATA CONVENTIONS

The following analysis conventions will be used in the statistical analysis.

## 6.1. Definition of Study Periods for Efficacy/Safety Variables

Baseline: Unless otherwise specified, the baseline assessment for all measurements will be the latest available valid measurement taken prior to the first administration of study drug. If multiple assessments (scheduled and unscheduled) exist with the same collection date (and time if collected), the scheduled assessment will be used as baseline.

Day 1 is the first administration day of study drug , Day -1 is the day before, and there is no Day 0.

Clinical assessments (ECOG, quality of life assessment, laboratory tests, and vital signs) scheduled to be done on the same date of the first dose of the study drug but obtained prior to the first study drug administration, will be considered as baseline evaluations. If the scheduled baseline Day 1 measurements are not available, screening assessments most recent prior to first study drug administration will be used. If multiple assessment records occur prior to the first study dosing, the scheduled record will be used for baseline.

## 6.2. Data Handling Convention for Efficacy Variables

Patients who are deemed NE according to Lugano classification will be considered as not reaching CR/PR in calculating ORR, i.e. they are not considered as responders in the numerator of ORR, but they are counted in the denominator of ORR.

## 6.3. Data Handling Convention for Missing Data

Medication missing/partial dates

- Medication start date
  - If only the day is missing: the first study treatment dose date will be used if the medication start year and month are the same as the first study treatment dose year and month. Otherwise, the first day of the month will replace the missing information. If this leads to a date after the medication end date, the medication end date will be used.
  - If both the day and the moth are missing but not the year: the first study treatment dose date will be used to replace the missing information if the medication start year is the same as the study treatment start year. Otherwise, the first day of the year will be used. If this leads to a date after the medication end date, the medication end date will be used.
  - If a date is completely missing, it will be considered as missing and no imputation will be done.
- Medication end date
  - If end day is missing: the last day of the month will be used


VV-RIM-00187995-1.0 Approved - 01 Mar 2022 GMT-5:00

- If both the day and the month are missing but not the year: the last day of the year will be used
- If a date is completely missing, it will be considered as missing and no imputation will be done.

If the medication start date is missing, the onset day will not be imputed in medication listings.

**Adverse event**

- Adverse event intensity and causality

  If the intensity of an adverse event is missing, it will be classified as “severe (Grade 3)” in the AE summary. If the assessment of relationship of an adverse event to the study treatment is missing, it will be classified as treatment-related.

- Adverse event start date
  - If only the day is missing: the first study treatment dose date will be used if the adverse event start year and month are the same as the first study treatment dose year and month. Otherwise, the first day of the month will replace the missing information. If this leads to a date after the adverse end date, the adverse end date will be used.
  - If both the day and the month are missing but not the year: the first study treatment dose date will be used to replace the missing information if the adverse event start year is the same as the study treatment start year. Otherwise, the first day of the year will be used. If this leads to a date after the adverse event end date, the adverse event end date will be used.
  - If a date is completely missing, it will be considered as missing and no imputation will be done.
- Adverse event end date.
  - If end day is missing: the last day of the month will be used
  - If both the day and the month are missing but not the year: the last day of the year will be used
  - If a date is completely missing, it will be considered as missing and no imputation will be done.

**Date of first / last infusion**

Date of first injection is the first non-missing start date of dosing filled in the CRF “Investigational Product” module.

No imputation will be done for missing data for laboratory, ECG, vital sign, or physical examination.



VV-RIM-00187995-1.0 Approved - 01 Mar 2022 GMT-5:00

## 6.4. Visit Windows

Assessments taken outside of protocol allowable windows will be displayed according to the case report form (CRF) assessment recorded by the investigator.

## 6.5. Unscheduled Assessments

The determination of baselines and values at the end of treatment for both efficacy and safety variables will be based on scheduled available assessments and unscheduled available assessments.

Extra assessments (laboratory data or vital signs associated with non-protocol clinical visits or obtained in the course of investigating or managing adverse events) will be included in listings, but not summaries except for the endpoint determination. If more than one laboratory value is available for a given visit, the first observation will be used in summaries and all observations will be presented in listings.

## 6.6. Pooling of Centers for Statistical Analyses

All participating centers in the study will be pooled together for analyses.

## 6.7. Statistical Technical Issues



VV-RIM-00187995-1.0 Approved - 01 Mar 2022 GMT-5:00

# 7. INTERIM ANALYSIS

An interim efficacy analysis for primary efficacy endpoint will be performed when 80 patients with FL grade 1-3a have completed 52-week assessment and all patients in the DLBCL cohort have completed 36-week assessment or have withdrawn from the study, whichever is later.

The ORR and associated 95% confidence interval will be summarized for FL and DLBCL cohorts. As the primary objective of this interim efficacy analysis is point estimation on ORR and characterization of the precision of point estimation, the study will not be stopped for perceived efficacy. For other efficacy endpoints, 2-sided 95% confidence interval will also be presented.

An interim futility analysis will be performed for each of the MCL, MZL, and other B-NHL disease-specific cohorts after the pre-specified number of evaluable patients in the cohort have completed tumor assessments at 28 weeks or have withdrawn from the study earlier (see Table 6. The futility boundary is determined based on lack of clinically meaningful activity below the threshold in each disease-specific cohort. The enrollment for each disease-specific cohort will continue unless its futility boundary is crossed.

- MCL after BTK inhibitor therapy cohort: The futility boundary of 15% ORR is determined due to the lack of standard of care options. Therefore, if fewer than 5 responders are observed among the first 27 patients, this cohort will be stopped for futility.
- MZL cohort: The futility boundary of 20% ORR is determined due to enrollment of patients with relapsed/refractory disease following failure of standard of care therapies. Therefore, if fewer than 6 responders are observed among the first 27 patients, this cohort will be stopped for futility.
- Other B-NHL cohort: The futility boundary of 10% ORR is determined for the entire cohort due to the enrollment of heterogenous subtypes with aggressive lymphomas that have poor outcomes in relapsed/refractory setting.  Due to low incidence, it is anticipated that there will be very few patients of each subtype in this cohort limiting formal statistical analysis of any subtype. Therefore, if fewer than 4 responders are observed among the first 34 patients, this cohort will be stopped for futility.

**Table 6: Interim Futility Analysis**

| **Study Cohort** | **Total Sample Size** | **Sample Size at Interim Futility** | **Interim futility stopping boundary** |
|---|---|---|---|
| FL grade 1-3a | 128 | NA | NA |
| DLBCL | 112 to 127 | NA | NA |
| MZL | 78 | 27 | <6 responders |
| MCL | 78 | 27 | <5 responders |
| Other B-NHL | 67 | 34 | <4 responders |

# 8. SOFTWARE

All analyses will be done using SAS Version 9.4 or above.

# 9. REFERENCES

1. ICH. (1998, February 5). ICH Harmonized tripartite guideline: Statistical principles for clinical trials (E9). International Conference on Harmonization of Technical Requirements for Registration of Pharmaceuticals for Human Use.
2. Cheson BD, Fisher RI, Barrington SF, et al. Recommendations for initial evaluation, staging, and response assessment of Hodgkin and non-Hodgkin lymphoma: the Lugano classification. J Clin Oncol. 2014;32(27):3059-3068. doi:10.1200/JCO.2013.54.8800
3. Lee DW, Santomasso BD, Locke FL, Ghobadi A, Turtle CJ, Brudno JN, et al. ASTCT Consensus Grading for Cytokine Release Syndrime and Neurologic Toxicity Associated with Immune Effector Cells. Biol Blood Marrow Transplant, 2019; 25 (4): 625-638.
4. European Organisation for Research and Treatment of Cancer. EORTC QLQ-C30 Scoring Manual. Brussels, Belgium: EORTC Data Center; 2001.
5. FACT. Functional Assessment of Cancer Therapy. https://www.facit.org/LiteratureRetrieve.aspx?ID=46138 Published in Febuary 2005.
6. Euroqol. EQ-5D-3L User Guide Version 6, https://euroqol.org/wp-content/uploads/2019/10/EQ-5D-3L-User-Guide_version-6.0.pdf; 2018



VV-RIM-00187995-1.0 Approved - 01 Mar 2022 GMT-5:00

# 10. APPENDIX

## 10.1. Summary of Statistical Analyses

### Efficacy Analysis:

| Endpoint | Analysis Populations | Primary Analysis | Statistical Method | Supportive Analysis | Subgroup Analysis | Other Analyses |
|---|---|---|---|---|---|---|
| **Primary Endpoint** | | | | | | |
| ORR per IRC | *FAS* | *ORR* | *Estimates of response rate, along with its exact two-sided 95% CI by Clopper and Pearson* | | *Yes Subgroups* | |
| **Secondary Endpoints** | | | | | | |
| ORR per Investigator | *FAS* | *ORR* | *Estimates of response rate, along with its exact two-sided 95% CI by Clopper and Pearson* | | *Yes Subgroups* | |
| CR rate per IRC | *FAS* | *CR Rate* | *Estimates of CR rate, along with its exact two-sided 95% CI by Clopper and Pearson* | CR rate per Investigat-or | | |
| DCR rate per IRC | *FAS* | *DCR* | *Estimates of disease control rate, along with its exact two-sided 95% CI by Clopper and Pearson* | DCR rate per Investigat-or | | |
| DOR per IRC | *FAS* | *Time from the date of the first documented CR or PR until the date of the first date of progressive disease, or death due to any cause, whichever occurs first* | *Estimated, median DOR, survival rate at the key landmark time point along with 95% confidence* | DOR per Investigat-or | | *Sensitivity* |

| **Endpoint** | **Analysis Populations** | **Primary Analysis** | **Statistical Method** | **Supportive Analysis** | **Subgroup Analysis** | **Other Analyses** |
|---|---|---|---|---|---|---|
| DOCR per IRC | *FAS* | *Time from the date of the first documented CR until the date of the first date of progressive disease, or death due to any cause, whichever occurs first* | *Estimated, median DOCR, survival rate at the key landmark time point along with 95% confidence* | DOCR per Investigat-or | | *Sensitivity* |
| TOR per IRC | *FAS* | *Time from the date of the first dose to the date of first documented CR/PR* | *Estimated, median TOR, survival rate at the key landmark time point along with 95% confidence* | TOR per Investigat-or | | |
| PFS per IRC | *FAS* | *Time from the start of study treatment until the first date of progressive disease, or death due to any cause, whichever occurs first* | *Estimated, median PFS, survival rate at the key landmark time point along with 95% confidence* | PFS rate per Investigat-or | | *Sensitivity analysis* |
| OS | *FAS* | *Time from the start of study treatment until death due to any cause* | *Estimated, median survival, survival rate at the key landmark time point along with 95% confidence* | | | |

## Safety Analyses:

| **Endpoint** | **Analysis Populations** | **Primary Analysis** | **Statistical Method** | **Supportive Analysis** | **Subgroup Analysis** | **Other Analyses** |
|---|---|---|---|---|---|---|
| Adverse Events | *SAF* | *Percent of patients with TEAEs by system organ class and/or preferred terms* | *Descriptive Statistics* | *No* | | |

| Endpoint | Analysis Populations | Primary Analysis | Statistical Method | Supportive Analysis | Subgroup Analysis | Other Analyses |
|---|---|---|---|---|---|---|
| Study Drug Administration | *SAF* | *Treatment duration, dose intensity, and number of doses administered* | *Descriptive Statistics* | Treatment compliance | | |
| Laboratory | *SAF* | *Shift tables* | *Descriptive Statistics* | *Change from baseline* | | |

## 10.2. Schedule of Time and Events

Details in the study protocol.

Signature Page for VV-RIM-00187995 v1.0

| | |
|---|---|
| Approval/eSignature | PPD<br>PPD<br>22-Feb-2022 14:17:34 GMT+0000 |

| | |
|---|---|
| Approval/eSignature | PPD<br>PPD<br>22-Feb-2022 14:22:14 GMT+0000 |

| | |
|---|---|
| Approval/eSignature | PPD<br>PPD<br>22-Feb-2022 14:44:04 GMT+0000 |

| | |
|---|---|
| Approval/eSignature | PPD<br>PPD<br>22-Feb-2022 14:54:14 GMT+0000 |

| | |
|---|---|
| Approval/eSignature | PPD<br>PPD<br>22-Feb-2022 15:53:55 GMT+0000 |

| | |
|---|---|
| Approval/eSignature | PPD<br>PPD<br>01-Mar-2022 15:03:01 GMT+0000 |

Signature Page for VV-RIM-00187995 v1.0 Approved
