# Supplementary material for: Odronextamab monotherapy in patients with relapsed/refractory diffuse large B cell lymphoma: primary efficacy and safety analysis in phase 2 ELM-2 trial
Source: Nat Cancer. 2025 Mar 17;6(3):528–39. doi: 10.1038/s43018-025-00921-6 (PMC12003196; doi:10.1038/s43018-025-00921-6)
Supplement: Supplementary file 2 — Reporting Summary [file 43018_2025_921_MOESM2_ESM.pdf]

Corresponding author(s): Won Seog Kim

Last updated by author(s): Dec 20, 2024

Reporting Summary

Nature Portfolio wishes to improve the reproducibility of the work that we publish. This form provides structure for consistency and transparency in reporting. For further information on Nature Portfolio policies, see our Editorial Policies and the Editorial Policy Checklist.

Statistics

For all statistical analyses, confirm that the following items are present in the figure legend, table legend, main text, or Methods section.

n/a	Confirmed	
☐	☒	The exact sample size (n) for each experimental group/condition, given as a discrete number and unit of measurement
☒	☐	A statement on whether measurements were taken from distinct samples or whether the same sample was measured repeatedly
☐	☒	The statistical test(s) used AND whether they are one- or two-sided<br>*Only common tests should be described solely by name; describe more complex techniques in the Methods section.*
☒	☐	A description of all covariates tested
☐	☒	A description of any assumptions or corrections, such as tests of normality and adjustment for multiple comparisons
☐	☒	A full description of the statistical parameters including central tendency (e.g. means) or other basic estimates (e.g. regression coefficient) AND variation (e.g. standard deviation) or associated estimates of uncertainty (e.g. confidence intervals)
☒	☐	For null hypothesis testing, the test statistic (e.g. F, t, r) with confidence intervals, effect sizes, degrees of freedom and P value noted<br>*Give P values as exact values whenever suitable.*
☒	☐	For Bayesian analysis, information on the choice of priors and Markov chain Monte Carlo settings
☒	☐	For hierarchical and complex designs, identification of the appropriate level for tests and full reporting of outcomes
☒	☐	Estimates of effect sizes (e.g. Cohen's d, Pearson's r), indicating how they were calculated

Our web collection on statistics for biologists contains articles on many of the points above.

Software and code

Policy information about availability of computer code

Data collection	No software was used
Data analysis	All analyses performed using SAS Version 9.4 or above.

For manuscripts utilizing custom algorithms or software that are central to the research but not yet described in published literature, software must be made available to editors and reviewers. We strongly encourage code deposition in a community repository (e.g. GitHub). See the Nature Portfolio guidelines for submitting code & software for further information.

Data

Policy information about availability of data

All manuscripts must include a data availability statement. This statement should provide the following information, where applicable:

- Accession codes, unique identifiers, or web links for publicly available datasets
- A description of any restrictions on data availability
- For clinical datasets or third party data, please ensure that the statement adheres to our policy

Patient personal data will be treated in compliance with all applicable laws and regulations. The sponsor shall take all appropriate measures to safeguard and prevent access to this data by any unauthorized third party. Qualified researchers can request access to study documents (including the clinical study report, study protocol with any amendments, blank case report form, and statistical analysis plan) that support the methods and findings in this manuscript. Individual anonymized participant data will be considered for sharing 1) once odronextamab and its indication are approved by major health authorities (for example, the U.S.

Food and Drug Administration, European Medicines Agency, Pharmaceuticals and Medical Devices Agency), or development of odronextamab has been discontinued on or after April 2020 and there are no plans for future development 2) if there is legal authority to share the data and 3) there is not a reasonable likelihood of participant reidentification. Requests should be submitted to https://vivli.org/.

Research involving human participants, their data, or biological material

Policy information about studies with human participants or human data. See also policy information about sex, gender (identity/presentation), and sexual orientation and race, ethnicity and racism.

Reporting on sex and gender	Findings apply to both sexes. At baseline 76/127 (59.8%) patients were male and 51 (40.2%) were female (see Table 1). Sex was self-reported by patients.<br><br>The analysis of study outcomes by gender was not predetermined, as gender is not recognized as a prognostic factor and there is no justification for expecting differences based on gender for this indication.
Reporting on race, ethnicity, or other socially relevant groupings	At baseline, 61/127 (48%) of patients were White, 53/127 (41.7%) were Asian, and 13/127 (10.2%) were not reported (see Table 1). Findings apply to all races.
Population characteristics	See Table 1 in manuscript, p 24-26
Recruitment	Measures to ensure diverse and inclusive enrollment: diverse trial sites; translated consent forms for under-represented populations; extended screening windows for patients with access constraints; broad eligibility criteria to include patients with controlled HIV infection, hepatitis B, and hepatitis C; and lower thresholds for those with compromised organ function due to lymphoma.<br><br>The participants for the study were recruited through a competitive enrollment process globally using the Interactive Response Technology (IRT) until the pre-specified sample size was reached. This approach allowed for a diverse range of participants from various backgrounds and locations, therefore mitigating selection bias.
Ethics oversight	The protocol and amendments were approved by the relevant institutional review boards and ethics committees, which are listed in Supplemental Table 6 of the manuscript (see "Supplemental Tables.xls file). The study was conducted in accordance with applicable regulatory requirements, guidelines of Good Clinical Practice as specified by the International Conference on Harmonization, and principles originating from the Declaration of Helsinki. All patients provided informed written consent before enrollment.

Note that full information on the approval of the study protocol must also be provided in the manuscript.

Field-specific reporting

Please select the one below that is the best fit for your research. If you are not sure, read the appropriate sections before making your selection.

☒ Life sciences ☐ Behavioural & social sciences ☐ Ecological, evolutionary & environmental sciences

For a reference copy of the document with all sections, see nature.com/documents/nr-reporting-summary-flat.pdf

Life sciences study design

All studies must disclose on these points even when the disclosure is negative.

Sample size	Assuming that a clinically meaningful ORR is greater than 35%, a sample size of 112 was considered adequate to observe an ORR of ≥45% with a CI lower bound that excludes 35%. Enrollment was increased to include at least 60 patients treated with 0.7/4/20 step-up dosing and up to 127 patients with 160 mg weekly dosing.
Data exclusions	No data were excluded. All patients treated in the global cohort were included
Replication	This is a single-arm multicohort study in different subtypes of lymphoma. The design of the study did not include multiple global cohorts for the same indication, therefore replication was not in-scope in this clinical study. However, we believe the findings could be reproduced, as similar results were obtained with other CD20 bispecific antibodies.
Randomization	Not applicable to this study (one treatment arm only)
Blinding	Not applicable to this study (open label)

Reporting for specific materials, systems and methods

We require information from authors about some types of materials, experimental systems and methods used in many studies. Here, indicate whether each material, system or method listed is relevant to your study. If you are not sure if a list item applies to your research, read the appropriate section before selecting a response.

Materials & experimental systems

n/a	Involved in the study
☐	☒ Antibodies
☒	☐ Eukaryotic cell lines
☒	☐ Palaeontology and archaeology
☒	☐ Animals and other organisms
☐	☒ Clinical data
☒	☐ Dual use research of concern
☒	☐ Plants

Methods

n/a	Involved in the study
☒	☐ ChIP-seq
☒	☐ Flow cytometry
☒	☐ MRI-based neuroimaging

Antibodies

Antibodies used

Odronextamab (an Fc-silenced, human CD20×CD3 bispecific antibody) was the treatment under investigation and was developed by Regeneron Pharmaceuticals, Inc. As this is an investigational product (IMP) studied in the trial, there is no catalog number. There were different dilutions for various step-up doses and different formulations of IMP used in the clinical trial, which were described in the respective pharmacy manual versions that were applicable at the time of treatment.

Validation

Odronextamab was validated by Smith EJ, et al. (Sci Rep 2015) in mice, where the antibody prevented growth of B cell tumors and also caused regression of large established tumors, and in cynomolgus monkeys, where low doses of the drug caused prolonged depletion of B cells in peripheral blood with a serum half-life of approximately 14 days.

Clinical data

Policy information about clinical studies

All manuscripts should comply with the ICMJE guidelines for publication of clinical research and a completed CONSORT checklist must be included with all submissions.

Clinical trial registration

NCT03888105

Study protocol

Full protocol not yet publicly available. Redacted protocol has been provided with this submission.

Data collection

Patients in the DLBCL global cohort (N=127) were enrolled from March 24, 2020, to May 18, 2022, at 54 sites. Patients were recruited from multiple clinical trial sites that included academic centers and community practices with both inpatient and outpatient facilties, across multiple countries including the USA, Australia, Canada, China, France, Germany, Italy, Japan, the Republic of Korea, Poland, Singapore, Spain, Taiwan, and the United Kingdom. Sites are listed on the clinicaltrials.gov page: https://clinicaltrials.gov/study/NCT03888105?term=elm-2&intr=odronextamab&rank=1#contacts-and-locations

Outcomes

The primary endpoint was objective response rate (ORR) assessed by independent central review according to Lugano criteria.
Secondary endpoints were:
- ORR according to Lugano Classification, assessed by local investigator
- Complete response rate, duration of response, progression-free survival, and disease control rate, each according to Lugano Classification and assessed by local investigator and independent central review
- Incidence and severity of TEAEs from the time of first patient dose until end of study
- Pharmacokinetics (concentration of odronextamab)
- Immunogenecity (anti-odronextamab antibodies)
- Changes in scores of patient-reported outcomes, as measured by the validated instruments EORTC QLQ-C30, FACT-Lym, and EQ-5D-3L

Plants

Seed stocks

N/A

Novel plant genotypes

N/A

Authentication

N/A
