# Supplementary material for: Odronextamab monotherapy in patients with relapsed/refractory diffuse large B cell lymphoma: primary efficacy and safety analysis in phase 2 ELM-2 trial
Source: Nat Cancer. 2025 Mar 17;6(3):528–39. doi: 10.1038/s43018-025-00921-6 (PMC12003196; doi:10.1038/s43018-025-00921-6)
Supplement: Supplementary file 4 — Completed CONSORT 2010 abstract checklist. [file 43018_2025_921_MOESM4_ESM.pdf]

Items to include when reporting a randomized trial in a journal or conference abstract

Item	Description	Reported on line number
Title	Identification of the study as randomized	NA – not randomized (single arm)
Authors *	Contact details for the corresponding author	Y – not in abstract due to word limit but on page 2 beneath affiliations
Trial design	Description of the trial design (e.g. parallel, cluster, non-inferiority)	Y – phase 2, multicohort included on line 1 of abstract (p3)
Methods		
Participants	Eligibility criteria for participants and the settings where the data were collected	Y – eligibility info on lines 2-3 of abstract. Further details on eligibility and data collection settings provided on p14-15 of manuscript.
Interventions	Interventions intended for each group	Y – lines 4-7 of abstract
Objective	Specific objective or hypothesis	Y – primary and secondary endpoints on lines 7-9 of abstract
Outcome	Clearly defined primary outcome for this report	Y - primary endpoint on line 7 of abstract
Randomization	How participants were allocated to interventions	NA
Blinding (masking)	Whether or not participants, care givers, and those assessing the outcomes were blinded to group assignment	Y – not in abstract due to word limit but blinding information provided on p9 of manuscript
Results		
Numbers randomized	Number of participants randomized to each group	NA
Recruitment	Trial status	Y – "ongoing" in line 1 of abstract
Numbers analysed	Number of participants analysed in each group	Y – N numbers provided on lines 9-16 of abstract
Outcome	For the primary outcome, a result for each group and the estimated effect size and its precision	Y – ORR result provided on line 10 of abstract. Effect size and precision details not included in the abstract due to word limit but

		can be found on p19 of the manuscript.
Harms	Important adverse events or side effects	Y – CRS, ICANS, and infections data provided on lines 13-17 of abstract
Conclusions	General interpretation of the results	Y – lines 17-18 of abstract
Trial registration	Registration number and name of trial register	Y – line 19 of abstract
Funding	Source of funding	Y – not included in the abstract due to word limit but included in the acknowledgements section on p21 of manuscript

**this item is specific to conference abstracts*
